# Six Highly Conserved Targets of RNAi Revealed in HIV-1-Infected Patients from Russia Are Also Present in Many HIV-1 Strains Worldwide

**DOI:** 10.1016/j.omtn.2017.07.010

**Published:** 2017-07-13

**Authors:** Olga V. Kretova, Daria M. Fedoseeva, Maria A. Gorbacheva, Natalya M. Gashnikova, Maria P. Gashnikova, Nataliya V. Melnikova, Vladimir R. Chechetkin, Yuri V. Kravatsky, Nickolai A. Tchurikov

**Affiliations:** 1 Department of Epigenetic Mechanisms of Gene Expression Regulation, Engelhardt Institute of Molecular Biology, 119334 Moscow, Russia; 2 Department of Retroviruses, State Research Center of Virology and Biotechnology Vector, 630559 Koltsovo, Russia; 3 Laboratory of Post-Genomic Research, Engelhardt Institute of Molecular Biology, 119334 Moscow, Russia

**Keywords:** HIV-1, RNAi targets, gene therapy, ultra-deep sequencing, conserved HIV-1 sequences

## Abstract

RNAi has been suggested for use in gene therapy of HIV/AIDS, but the main problem is that HIV-1 is highly variable and could escape attack from the small interfering RNAs (siRNAs) due to even single nucleotide substitutions in the potential targets. To exhaustively check the variability in selected RNA targets of HIV-1, we used ultra-deep sequencing of six regions of HIV-1 from the plasma of two independent cohorts of patients from Russia. Six RNAi targets were found that are invariable in 82%–97% of viruses in both cohorts and are located inside the domains specifying reverse transcriptase (RT), integrase, vpu, gp120, and p17. The analysis of mutation frequencies and their characteristics inside the targets suggests a likely role for APOBEC3G (apolipoprotein B mRNA editing enzyme, catalytic polypeptide-like 3G, A3G) in G-to-A mutations and a predominant effect of RT biases in the detected variability of the virus. The lowest frequency of mutations was detected in the central part of all six targets. We also discovered that the identical RNAi targets are present in many HIV-1 strains from many countries and from all continents. The data are important for both the understanding of the patterns of HIV-1 mutability and properties of RT and for the development of gene therapy approaches using RNAi for the treatment of HIV/AIDS.

## Introduction

The current highly active antiretroviral therapy has greatly improved the morbidity and mortality of AIDS. However, many of the antiretroviral drugs exert high toxicity upon long-term usage, and all currently used anti-HIV agents generate the appearance of drug-resistant mutants. Therefore, there is a great need for the development of new approaches to HIV/AIDS therapy.

RNAi is a powerful tool to inhibit HIV-1 production in human cells. RNAi was suggested for silencing HIV-1 genes shortly after the discovery of RNAi.^1^, ^2^, ^3^, ^4^, ^5^, ^6^, ^7^, ^8^, ^9^, ^10^ Virus-specific RNAs transcribed from proviral DNA can be attacked in CD4^+^ T cells and macrophages, which are the natural targets of HIV-1, and, as a result, the replication of the virus can be inhibited. The main problem in the development of an approach using RNAi for the treatment of HIV/AIDS is the high variability of the virus. This natural feature of the virus, which is important for its fitness, is mainly due to (1) the viral error-prone reverse transcriptase (RT), (2) recombination during DNA synthesis in the co-packaged genomes leading to an increase in the genetic diversity, and (3) the extremely high level of HIV-1 amplification, which leads to a large population of variants. Additionally, the host protein APOBEC3G (apolipoprotein B mRNA editing enzyme, catalytic polypeptide-like 3G, A3G) produces cytidine deamination (cytosine to uracil) during reverse transcription of the single-stranded newly synthesized minus-strand cDNA, leading to extensive G-to-A mutations in the viral plus strand inside the double-stranded DNA genome; this phenomenon is called hypermutation.^11^, ^12^, ^13^, ^14^, ^15^, ^16^, ^17^, ^18^ Together, these mechanisms lead to high variability in the HIV-1 genome and hamper the development of an RNAi approach for the treatment of HIV/AIDS. In fact, even a single point mutation in a target sequence can allow the virus to escape RNAi.^19^, ^20^

Nevertheless, attempts to find targets that are compatible with a majority of the current HIV-1 variants have proceeded using two main strategies: (1) multiplexing, which is the use of several siRNAs against different targets in HIV-1 transcripts,^21^, ^22^ or (2) targeting of RNAs corresponding to highly conserved regions of the viral genome.^23^, ^24^, ^25^ For example, using dual-siRNA hairpin constructs that simultaneously target two viral transcripts helps to avoid the formation of RNAi-escape mutants.^21^ The accessible conserved isles in the coding regions of the viral mRNAs are good targets for RNAi because of a constraint in the number of variations tolerated in these critical coding regions. Currently, studies to improve RNA stability and delivery to the target cells are underway, and preclinical studies examining the efficiency of RNAi for viral suppression and gene therapy clinical trials for HIV-1-infected patients are ongoing (see Bobbin et al.^26^ and Klemm et al.^27^ for a review); however, RNAi-based therapy has not yet reached the clinic.

Here, we describe the results of ultra-deep sequencing of six conserved HIV-1 regions from two independent cohorts of patients in Russia. Our data indicate that up to 92%–97% of viruses possess six identical RNAi targets. The corresponding Dicer substrates efficiently attacked the targets in a non-viral system using the *luc* reporter gene. The data suggest that the detected targets could be used for the development of a gene therapy approach to treat HIV/AIDS.

## Results

### Alignment of Ultra-Deep Sequencing Reads Reveals Conserved Sequences in the RNAi Targets

**Figure 1:**
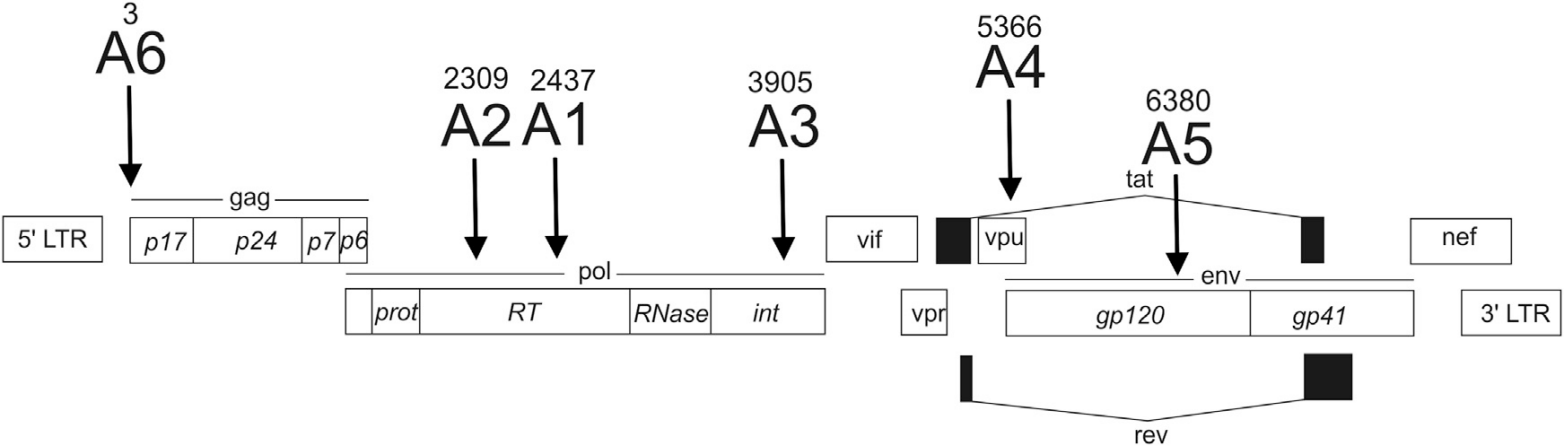
Schematic Presentation of RNAi Targets A1–A6 within the HIV-1 Gene Map The positions of 19-bp targets are indicated. The values shown above the targets indicate the 5′ numbering of a target in the reference sequence (GenBank: AF316544).

Selected siRNA targets in different conserved functionally important coding regions in the HIV-1 subtype A genome are shown in Figure 1. They are indicated as A1–A6 (A denotes the subtype of the virus). A1 and A2 are located inside the RT domain and A3 is inside the integrase domain (int), whereas A4, A5, and A6 are inside the domains specifying vpu, gp120, and p17, respectively.

Target A1 was selected using the Tuschl algorithm, whereas the remaining (A2, A3, A4, A5, and A6) targets were selected using the Dharmacon (http://dharmacon.gelifesciences.com/design-center/). All six RNAi targets are located in the fully spliced HIV-1 mRNA species and correspond to the protein-coding regions (Figure S13).^28^ The reads of the ultra-deep sequencing were aligned using the procedure described in the Materials and Methods section, which allows the alignment and analysis up to about eight million reads. The data on the deep sequencing of the viruses from both cohorts were deposited in NCBI (Bioproject: PRJNA344431).

**Figure 2:**
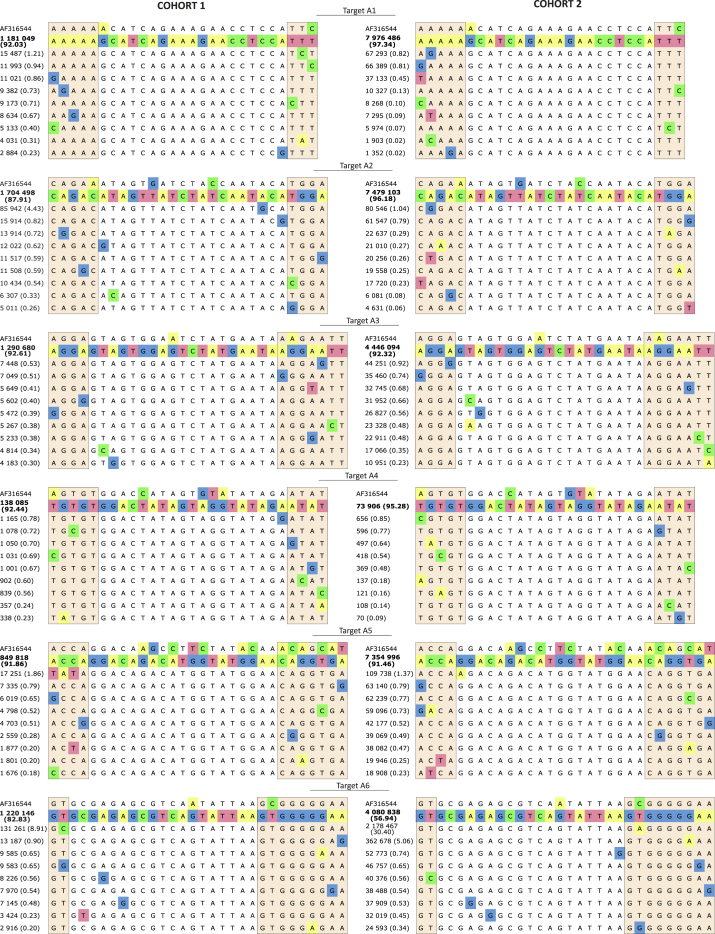
Alignments of Deep Sequencing Reads from Both Cohorts The top ten alignments are shown. The complete alignments are shown in Figures S1–S12. The reference sequence is shown on the top (GenBank: AF316544). The second line represents the sequences observed in the majority of reads. The number and percentage of reads are indicated. The 19-nt core RNAi sequences are not shaded.

In our preliminary studies, we analyzed the same genomic regions using cloning and capillary sequencing. In total, 42 clones that included about 300-bp fragments of DNA and corresponded to the mixture of the viruses of cohort 1 were analyzed (GenBank: KC681847–KC681888). We deduced the consensus sequences of the targets based on the analysis of a limited number of clones.^29^, ^30^, ^31^ Surprisingly, very similar target sequences were found in the mixtures of viruses from both cohorts, now based on analysis of thousands or millions of reads (Figures 2 and S1–S12; Table 1). It is of note that practically the same characteristic changes with respect to the reference sequence (isolate from the Republic of the Congo, GenBank: AF316544, 2001) were detected in all targets from both cohorts.

**Table 1:** Sequences of the 27- to 30-nt Regions Possessing RNAi Targets that Are Shared by the Majority of Viruses in Both Cohorts

Percentage of viruses possessing particular 27- to 30-nt regions in the deep sequencing data is shown after the corresponding sequence. The 19-nt core sequences corresponding to overlapping regions in double-stranded siRNAs are underlined. Percentage of viruses containing identical core sequence is shown in parentheses. The changes in nucleotide sequences from the reference sequence (isolate 97CDKP58e, 2001, from the Republic of the Congo, GenBank: AF316544) are highlighted in yellow. The changes between sequences in the same cohort are highlighted in green. The number of high-quality reads possessing the indicated target is shown in the rightmost column. C1, cohort 1; C2, cohort 2. The data on cloned sequences were deposited in GenBank: KC681847–KC681888. The deep sequencing data for the two cohorts were deposited in NCBI (Bioproject: PRJNA344431). seq., sequence.

Table 1 shows the 27- to 30-bp regions that are found in up to 97% of viruses. For target A6 in cohort 2, we observed that only 56% of viruses possessed the identical target, whereas about 30% contained one nucleotide substitution (T to A) outside of the core sequence if compared with the result for cohort 1. The values for the 19-bp core sequences corresponding to the overlapping plus and minus strands in double-stranded siRNAs (ds-siRNAs) are even higher for all targets (up to 99%; Table S1). A core sequence does not include the dinucleotide 3′ overhangs of ds-siRNA and is critical for the efficiency of siRNA in the initiation of RNAi.^32^ These A1–A6 regions correspond to the conserved coding regions in the HIV-1 genome. Figure S13 shows the amino acid sequences of the conserved domains from the Conserved Domains Database (CDD) and Resources (NCBI) (https://www.ncbi.nlm.nih.gov/Structure/cdd/cdd.shtml) and indicates the stretches corresponding to the conserved RNAi targets.

### Lower Frequencies of Mutations Were Observed in the Middle Part of the RNAi Targets

**Figure 3:**
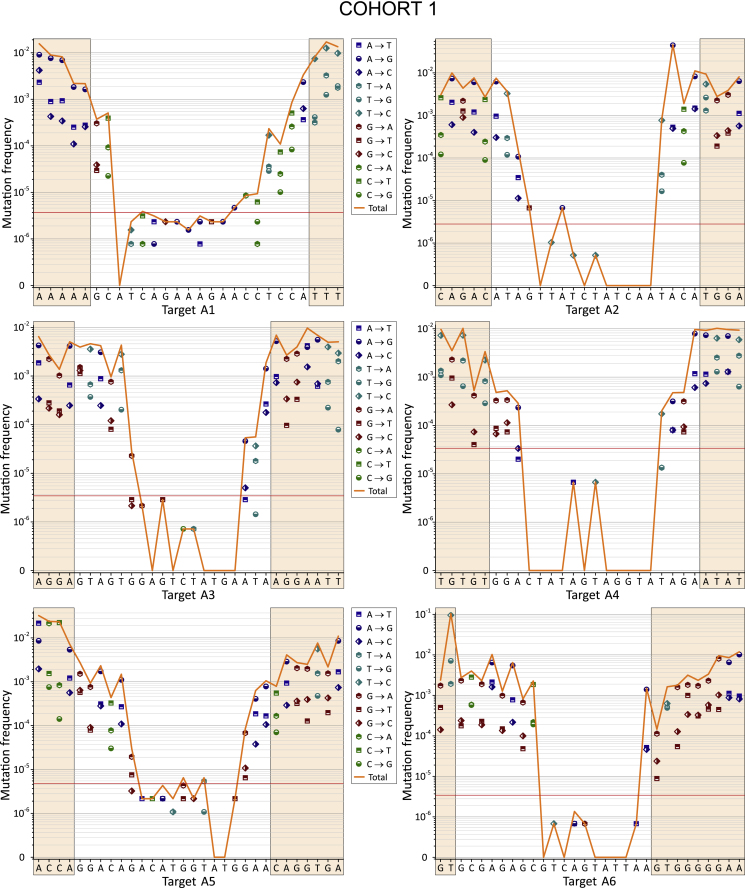
Curve Showing the Frequencies of Nucleotide Substitutions along the 27- to 30-nt Targets for Cohort 1 The horizontal red line corresponds to the threshold of reliable mutation detection (Equation 5 in Materials and Methods). The frequencies were determined against the most invariable RNAi target and were calculated by Equation 1 (Materials and Methods).

**Figure 4:**
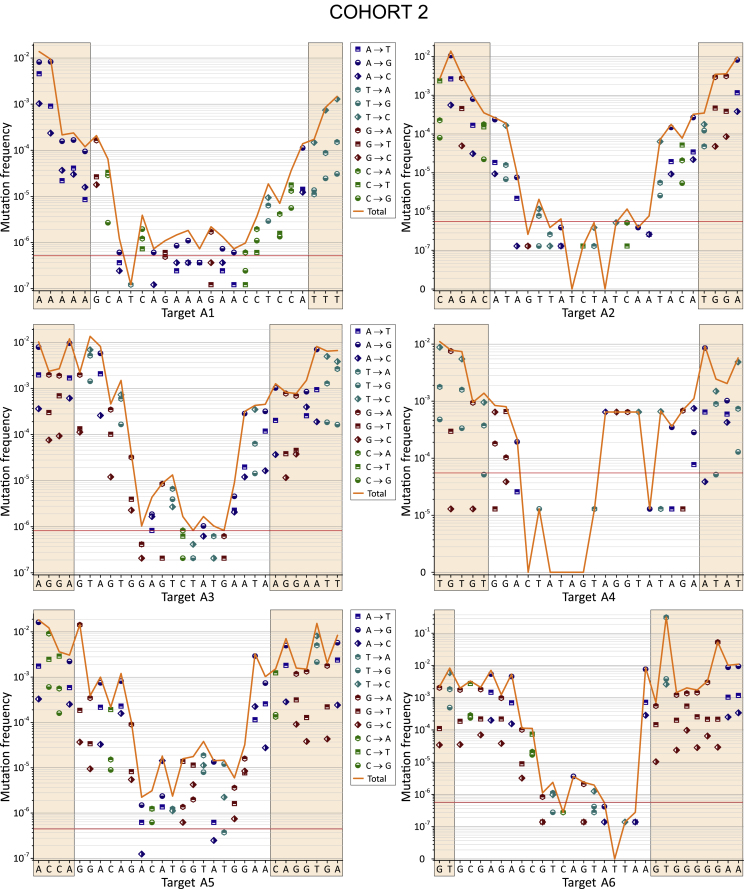
Curve Showing the Frequencies of Nucleotide Substitutions along the 27- to 30-nt Targets for Cohort 2 The horizontal red line corresponds to the threshold of reliable mutation detection (Equation 5 in Materials and Methods). The frequencies were determined against the most invariable RNAi target and were calculated by Equation 1 (Materials and Methods).

Next, we used the deep sequencing data for the analysis of the mutation profiles and mutation characteristics along the targets. Figures 3 and 4 show the profiles of all 12 possible nucleotide substitutions (four transitions and eight transversions) along the 27- to 30-bp sequences in the plus strand of the HIV-1 DNA. In this study, the frequencies of nucleotide substitutions were determined against the sets of aligned sequences (Equations 1 and 2 in Materials and Methods), which is more convenient and unambiguous in the case of deep sequencing data. All 12 profiles (six regions from two cohorts) demonstrate mutation frequencies in a wide range from 10^−7^ to 10^−1^.

The interesting common feature of all profiles is that they exhibit a U-shape and demonstrate the lowest mutation frequencies in the middle part of the sequences that correspond to the core siRNA stretches. The data may indicate that the corresponding conserved regions are important for the function of HIV-1 proteins. One example that supports this conclusion is the target A2, which spans the region specifying the IVIYQYMD stretch in the RT enzyme (Figure S13). Residue M184 in this stretch lies close to the primer terminus and near to the binding site for the incoming nucleoside triphosphate (dNTP). M184 is a part of the dNTP-binding site of HIV-1 RT (dNTP-binding pocket) and is located in the highly conserved YMDD motif, which is found in all retroviruses.^33^ Y183 in this motif contributes to both dNTP affinity and processivity of RT.^34^

**Figure 5:**
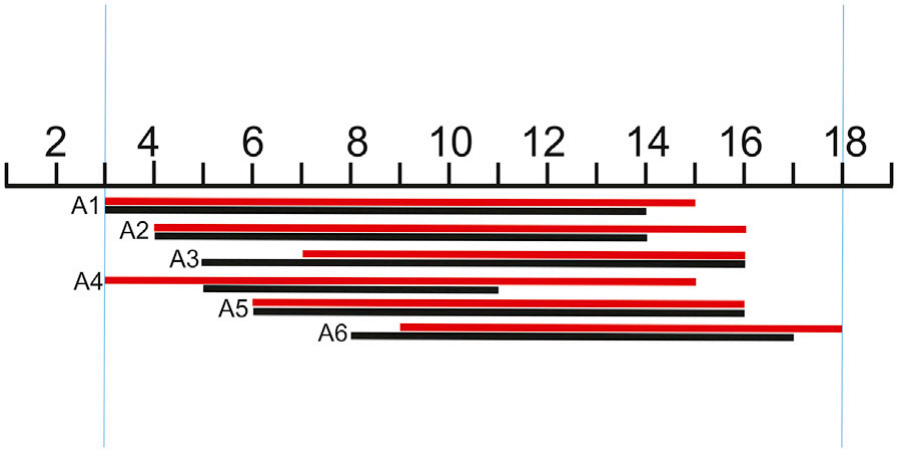
The Most Conserved Positions inside 19-nt RNAi Core Sequences The regions possessing the mutation frequencies below 10^−4^ (see Figures 3 and 4) are shown between the blue lines. The values indicate the number of the nucleotide in a target. Cohort 1 is shown in red. Cohort 2 is shown in black.

The second reason for the collection of sequences possessing a much lower mutation rate in the middle part is the selection procedure used for the design of active siRNAs. It is known that the requirements for the core 19-nt sequence includes a number of characteristics associated with siRNA functionality: low G/C content, a bias toward low internal stability at the sense-strand 3′ terminus, lack of inverted repeats, and sense-strand base preferences (positions 3, 10, 13, and 19).^32^ The regions in the range from 3 to 18 nt in the core 19-nt sequences from the 12 profiles are the most conserved (mutation frequencies below 10^−4^; Figure 5). Therefore, we conclude that both the limitations due to the biological functions of HIV-1 proteins and the rules for the design of efficient siRNAs shaped the selected RNAi targets.

### Characteristics of the Mutations inside the 27- to 30-bp DNA Sequences that Include the RNAi Targets

Mutations in HIV-1 have been studied for many years, and during the last decade, a novel possibility for the analysis of such mutations was provided by deep sequencing. Although there are some problems in using this approach for the complete genome assembly of the virus based on short read lengths,^35^ the method is suitable for the detailed analysis of mutations in short genomic regions from viral populations. The main source of the high variability of HIV-1 is the error-prone nature of RT, although recently, it was discussed whether APOBECK3 proteins could also significantly contribute to the genetic diversity of HIV-1 inside human cells.^15^, ^16^

**Figure 6:**
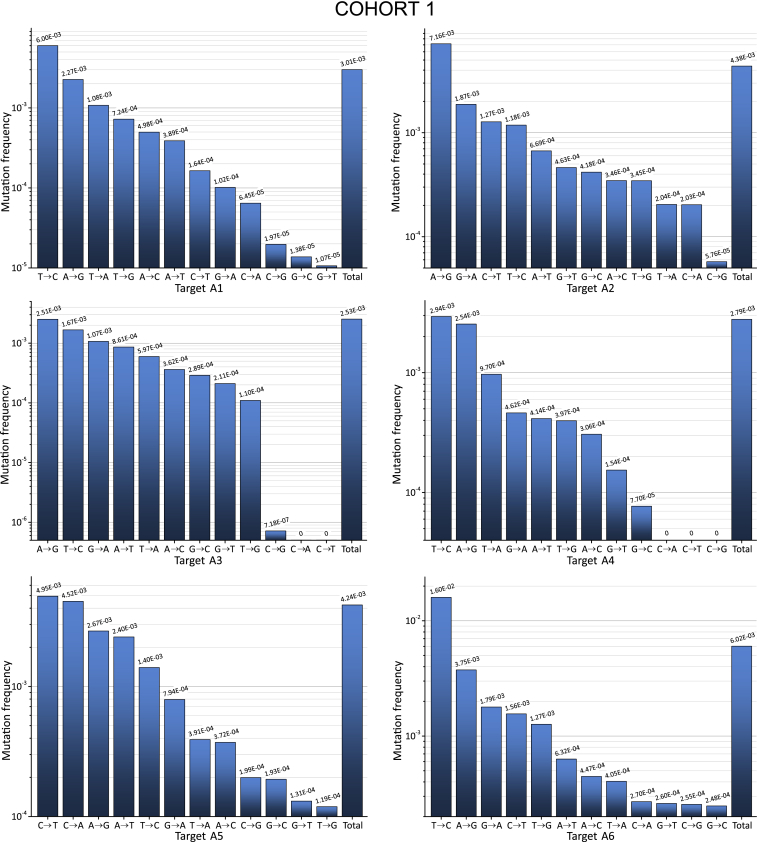
Frequencies of All Possible Nucleotide Substitutions along the 27- to 30-nt Targets for Cohort 1 The frequencies were determined against the most invariable RNAi target and were calculated by Equation 1 (Materials and Methods).

**Figure 7:**
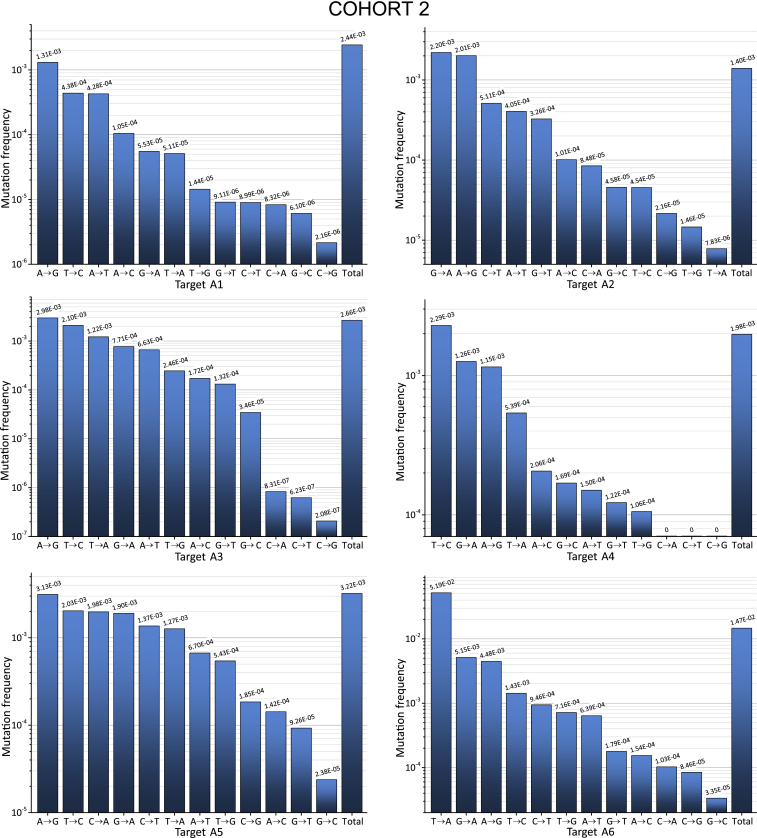
Frequencies of All Possible Nucleotide Substitutions along the 27- to 30-nt Targets for Cohort 2 The frequencies were determined against the most invariable RNAi target and were calculated by Equation 1 (Materials and Methods).

We attempted to answer this question using deep sequencing reads for analysis of mutations. It is known that changes either in the HIV-1 RT or in the sequence of the nucleic acid template can affect the spectrum of mutations produced during viral replication.^36^ From this point of view, the spectrum of mutations in our study, which is based on the wild-type RT in a population of viruses, could vary, depending only from sequences of different targets. As expected, we observed that the frequencies of particular mutations varied for different targets (Figures 6 and 7).

We calculated the frequencies in all nucleotide substitutions observed in the regions possessing the RNAi targets, as described in the Materials and Methods section. A3G, a single-stranded DNA cytidine deaminase, could generate G-to-A mutations in the plus strand of HIV-1 DNA. The data shown in Figures 6 and 7 indicate that the G-to-A mutations are frequent but were dominant only inside target A2 in cohort 2. Therefore, we conclude that A3G is not the main source of the observed variability in the viruses isolated from plasma.

The main nucleotide substitutions observed were transitions T to C and A to G, which are the most frequent in all targets. The reverse transitions C to T or G to A occurred less frequently. We conclude that these mutations reflect the nature of the RT, which is prone to low fidelity in the discrimination between a pair of purines or pyrimidines. Some of the nucleotide substitutions, such as C to G and G to C, for all targets occur at a low rate. These bases are likely the most clearly discriminated by the enzyme. At the same time, we observed a strong sequence dependence of errors made by the RT. For example, targets A1 and A5 both possess six and five C bases, respectively, but the C-to-T transitions in them differ by 30- or 152-fold in cohorts 1 and 2, respectively. A more detailed analysis of mutations that reflects the nature of the RT will be published separately.

### Y183 and M184 Variants of Mutations in the 3′ Flank of Target A2 Occur at High Frequencies

Of particular interest are the mutations in the target A2, which possesses the sequence that specifies the highly conserved YMDD motif that is found in all retroviruses (Figure S13). We selected patients that had been receiving antiretroviral therapy for up to 5 years but still possessed high-titer viremia (cohort 2) in order to check the putative changes inside the RNAi targets induced by the drugs. It is known that M184 is mutated to a valine (M184V) in response to lamivudine or emtricitabine treatment^37^. However, in the population of viruses from cohort 2, we observed only the M184I mutation at a high frequency (up to 2.3 × 10^−3^; Table 2). In contrast, in cohort 1, we detected M184V at high frequencies (9 × 10^−2^) as well as a number mutations due to substitutions in the second and third bases of the ATG codon: M184T (6 × 10^−3^), M184R (1.7 × 10^−3^), M184K (1.5 × 10^−3^), and M184I (2.5 × 10^−3^). M184 variants display a reduced replication capacity and transmission efficacy and tend to revert over time in the new host.^38^

**Table 2:** Characteristics of the Mutations in Target A2 that Affect Y183 and M184 in RT

Amino Acid, Codon	First Base Mutations, Codons, Amino Acids/Frequencies	Second Base Mutations, Codons, Amino Acids/Frequencies	Third Base Mutations, Codons, Amino Acids/Frequencies
**Cohort 1**			

Y183, TAC	T to C, CAC, H/7·10^−4^	A to G, TGC, C/5·10^−2^	C to T, TAT, Y/1.5·10^−3^
	T to A, AAC, N/4.5·10^−5^	A to T, TTC, F/6·10^−4^	C to A, TAA, Stop/4.5·10^−4^
	T to G, GAC, D/1.8·10^−5^	A to C, TCC, S/15.5·10^−5^	C to G, TAG, Stop/8.5·10^−5^
M184, ATG	A to G, GTG, V/9·10^−2^	T to C, ACG, T/6·10^−3^	G to A, ATA, I/2.5·10^−3^
	A to T, TTG, L/1.8·10^−3^	T to G, AGG, R/1.7·10^−3^	G to C, ATC, I/3.7·10^−4^
	A to C, CTG, L/1.7·10^−3^	T to A, AAG, K/1.5·10^−3^	G to T, ATT, I/2·10^−4^

**Cohort 2**			

Y183, TAC	T to C, CAC, H/7·10^−5^	A to G, TGC, C/1.5·10^−4^	C to T, TAT, Y/6·10^−5^
	T to A, AAC, N/6·10^−5^	A to T, TTC, F/2·10^−5^	C to A, TAA, Stop/2·10^−5^
	T to G, GAC, D/3·10^−6^	A to C, TCC, S/9·10^−6^	C to G, TAG, Stop/5.5·10^−6^
M184, ATG	A to G, GTG, V/3·10^−4^	T to C, ACG, T/1.0·10^−4^	G to A, ATA, I/2.3·10^−3^
	A to T, TTG, L/3·10^−5^	T to G, AGG, R/3·10^−5^	G to C, ATC, I/5.2·10^−4^
	A to C, CTG, L/2·10^−5^	T to A, AAG, K/5·10^−5^	G to T, ATT, I/5·10^−5^

Among Y183 variants, only one was detected at a high frequency: Y183C (5 × 10^−3^) from cohort 1 (Table 2). This mutant has only 5% of the wild-type RT activity,^39^ and the mutation likely spread such a high frequency with the aid of the wild-type RT during multiple infections of human cells.^40^

### Dicer Substrates Corresponding to the Detected Targets Induce Efficient RNAi In Vitro

Deep sequencing data allowed us to select six conserved regions that were detected in the majority (from 92.39% to 99.58% in the 19-nt core sequences) of the HIV-1 subtype A viruses in both cohorts. The data led us to test the efficiency of RNAi triggered by 27-bp Dicer substrates corresponding to the detected targets. Dicer substrates demonstrate the same or better efficacy of RNAi compared with double-stranded siRNA formed by 21-nt RNAs.^41^, ^42^ Table 3 shows the sequences of chemically synthesized RNAs that were annealed to form 27-bp double-stranded molecules with blunt ends.

**Table 3:** RNA Sequences that Were Used for the Preparation of Dicer Substrates and the DNA Sequence Containing Six RNAi Targets

Target	Domain	Target Sequence, 5′-3′	RNAs, 5′-3′
A1	RT	AAAAAGCATCAGAAAGAACCTCCATTT	AAAAAGCAUCAGAAAGAACCUCCAUUU
			AAAUGGAGGUUCUUUCUGAUGCUUUUU
A2	RT	AGACATAGTTATCTATCAATACATGGA	AGACAUAGUUAUCUAUCAAUACAUGGA
			UCCAUGUAUUGAUAGAUAACUAUGUCU
A3	Int	AGGAGTAGTGGAGTCTATGAATAAGGA	AGGAGUAGUGGAGUCUAUGAAUAAGGA
			UCCUUAUUCAUAGACUCCACUACUCCU
A4	vpu	GTGTGGACTATAGTAGGTATAGAATAT	GUGUGGACUAUAGUAGGUAUAGAAUAU
			AUAUUCUAUACCUACUAUAGUCCACAC
A5	gp120	ACCAGGACAGACATGGTATGGAACAGG	ACCAGGACAGACAUGGUAUGGAACAGG
			CCUGUUCCAUACCAUGUCUGUCCUGGU
A6	p17	GTGCGAGAGCGTCAGTATTAAGTGGGG	GUGCGAGAGCGUCAGUAUUAAGUGGGG
			CCCCACUUAAUACUGACGCUCUCGCAC

The 19-nt core sequences are underlined. The pairs of RNA molecules are shown in the 5′-3′ direction. These pairs could form 27-bp double-stranded RNAs (Dicer substrates).

**Figure 8:**
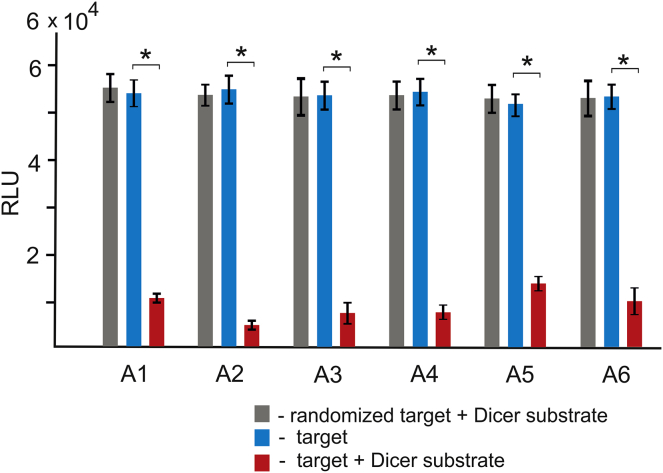
Efficiency of RNAi Initiated by the Dicer Substrates Results of co-transfection experiments are shown (see Materials and Methods). Luminescence of the *Renilla luc* gene (RLU) was normalized to that of the firefly *luc* gene. *p < 0.005.

We used co-transfection of a plasmid-containing DNA fragment containing the 27-nt RNAi target, which was cloned into the psiCHECK-2 vector (Promega) and the corresponding Dicer substrate. The vector was specially designed for quantitative testing of RNAi efficacy. It possesses two *luc* reporter genes (one from firefly and one from *Renilla*). The DNA fragment containing the targets was cloned into the 3′ UTR of the *Renilla* gene, which is important for expression. Both reporter genes are expressed after transfection, but only the *Renilla* mRNA can be attacked by the RNAi. Figure 8 shows the results of co-transfection experiments, in which expression of the firefly gene was used for normalization of the *Renilla* luminescence. Consistent RNAi-mediated silencing was observed in the range 81%–91% for the different targets. The randomized sequences of the targets were not attacked by RNAi. The data strongly indicate that the Dicer substrates corresponding to the six RNAi targets are processed into efficient siRNAs.

### Identical RNAi Targets Are Present in Many HIV-1 Isolates from Different Countries

The data on the existence of conserved HIV-1 sequences that are the same in two independent cohorts of HIV-infected patients from Russia and could serve as RNAi targets encouraged us to determine whether the identical targets are present in different isolates from around the world. It was of interest to estimate the percentage of HIV-1 sequences possessing these targets, similar to the deep sequencing reads that we performed in the course of this study. However, such a search is hampered because we cannot analyze all the HIV-1 databases by the same way. Therefore, we used a simple approach and exploited the regular BLAST search tool (https://blast.ncbi.nlm.nih.gov/Blast.cgi?PAGE=MegaBlast&PROGRAM=blastn&BLAST_PROGRAMS=megaBlast&PAGE_TYPE=BlastSearch&DBSEARCH=true&QUERY=&SUBJECTS =). We selected the Nucleotide collection (nr/nt) and Max target sequences as 5,000, assuming that this would permit the approximate estimation of the spread of the corresponding targets in different isolates. For the targets A1–A3, A4, and A6, we obtained only HIV-1 sequences among the 5,000 BLAST hits. In the case of A5, only sequences from Russia were observed among non-HIV-1 hits. Surprisingly, we found that targets identical to the 19-bp core sequences of A1–A3, A4, and A6 were detected in different HIV-1 isolates from different countries on all continents (Table 4). Of course, the data strongly depend upon the number of isolates that were sequenced from a particular country. Nevertheless, the data strongly suggest that the identical targets A1, A2, and A6 are widely dispersed. We detected 100% identical sequences in the majority of the BLAST hits: in 4,956 HIV-1 sequences (99%) for A6, 4,837 sequences (96.7%) for A2, and 4,651 sequences (93%) for A1. The A3 target was not so widespread and was detected in 1,395 sequences (27.9%). The A2 target is very abundant in isolates from USA, UK, Switzerland, and Germany. The target A6 is characteristic of isolates from Kenya, USA, UK, Switzerland, and Thailand.

**Table 4:** Spread of the Identical Targets in HIV-1 Isolates from Different Countries

Target/Domain	19-nt Core Sequence	Number of Identical BLAST Hits	Country
A1/RT	GCATCAGAAAGAACCTCCA	4,651	Russia, USA, Uzbekistan, Spain, Malaysia, Kenya, Rwanda, Ukraine, China, Uganda, Cameroon, Nigeria, South Africa, South Korea, Hungary, Iran, Brazil, UK, Germany, Morocco, Ethiopia, Senegal, Mexico, Togo, Panama, French Guiana, Belgium, Angola, Sierra Leone, Ghana, Thailand, D.R. Congo, Burundi, Puerto Rico, Australia, Poland, Algeria, Israel, Cuba, India, Botswana, Jamaica, Turkey, Tanzania, Bulgaria, Cyprus, Portugal
A2/RT	ATAGTTATCTATCAATACA	4,837	Russia, USA, South Africa, France, Uzbekistan, Spain, Malaysia, Kenya, South Korea, UK, Peru, China, Switzerland, Germany, Uganda, Cameroon, Surinam, Brazil, Mexico, Kuwait
A3/Int	GTAGTGGAGTCTATGAATA	1,395	Russia, Uzbekistan, Rwanda, Ukraine, Kenya, Spain, Zambia, Armenia, Kirgizstan, Kazakhstan, France, Uganda, Canada, South Africa, UK, Cyprus, Cameroon, Germany, Belgium, Cuba, Georgia
A4/Vpu	GGACTATAGTAGGTATAGA	768	Russia, Rwanda, Ukraine, Iran, Nigeria, Uganda, D.R. Congo, Kenya, Spain, Cameroon, India, China, Myanmar, Thailand, Cyprus, Afghanistan, USA, Viet Nam, South Africa, Denmark, Tanzania, Kazakhstan, Uzbekistan, Georgia, Botswana
A5/Gp120	GGACAGACATGGTATGGAA	–	Russia
A6/P17	GCGAGAGCGTCAGTATTAA	4,956	USA, Thailand, Russia, Kenya, Rwanda, Cameroon, Pakistan, Ukraine, Brazil, China, India, Nigeria, Cameroon, Greece, Tanzania, Spain, Uganda, Senegal, UK, South Africa, Germany, Botswana, Portugal, D.R. Congo, Burkina Faso, France, Zambia, Cuba, India, Guinea-Bissau, Sweden, Estonia, Malaysia, Iran, Canada, Myanmar, Switzerland, Netherlands, South Korea, Malawi, Poland

The search using nucleotide BLAST (Nucleotide collection [nr/nt]) was performed using the 19-nt core sequences. The number of identical hits is indicated. The list of countries is shown in the order of the number of corresponding hits that appeared in the list. The search was performed for the maximum of 5,000 target sequences with automatically adjusted parameters for short input sequences. The target A5 was detected only in Russia.

Next, we decided to use the Los Alamos database, which includes all HIV sequences available worldwide (http://www.hiv.lanl.gov). Tables S3–S7 shows the results of the HIV BLAST search using a specialized HIV sequence database (https://www.hiv.lanl.gov/content/sequence/BASIC_BLAST/basic_blast.html). The tool allows the search of up to only 200 HIV-1 sequences. The data indicate that the identical targets are present in all 200 tested sequences. Interestingly, we observed that these targets were found in HIV-1 strains from different countries and corresponded to different subtypes, including 11 subtypes for A4 and 20 subtypes for A6. For example, the list for A6 includes subtypes A1, A1C, A1D, A1CD, B, 01B, 01BC, C, G, 01_AE, F1, 69_01B, 70_BF1, 58_01B, 56_cpx, 35_AD, 65_cpx, BF1, 28_BF, and 29_BF. The Los Alamos HIV database includes all available HIV sequences worldwide, including the full-length HIV-1 genomic sequences. This comprehensive collection of HIV sequences is important for both the choice of the most conserved protein sequences for vaccine design and selection of siRNA sequences.^26^, ^43^ The deep sequencing approach for selecting the conserved RNAi targets that we used is limited by the use of viruses from a small number of patients and thus does not provide a global representation; however, our method reveals the diversity of millions of HIV variants that are present in every patient of our cohorts. Deep sequencing is also suitable for the detailed analysis of RT biases. Both approaches could be combined. The data shown in Tables S3–S7 indicate that the HIV samples of different subtypes from the Los Alamos database possess sequences that are identical to the RNAi targets detected by deep sequencing. Therefore, we conclude that the targets detected by deep sequencing in about 90% of HIV-1 isolates from Russia are also present in many HIV-1 isolates worldwide.

## Discussion

The data based on the analysis of about 7 × 10^6^ aligned reads representing two independent cohorts of HIV-infected patients strongly support the conclusion that the studied isolates possess six conserved regions that could serve as RNAi targets. Surprisingly, in such a highly mutable virus, we detected the identical targets in both cohorts. Up to 99.5% of the viruses analyzed possessed the same six 19-bp RNAi targets. Moreover, many HIV-1 isolates corresponding to diverse subtypes from different countries contain the identical sequences in the A1, A2, and A6 regions, so these could potentially be used as the targets for RNAi-based gene therapy. These particular sequences have great potential for the development of an artificial RNAi approach because they were found in 93%–99% of 5,000 BLAST hits. It has been described that using two targets that are conserved in up to 80% of viruses could provide efficient silencing in up to 95% of viruses.^31^ Of course, for the development of this approach, one should solve the very important problem of the delivery of Dicer substrates, siRNA, or genetic constructs expressing the corresponding small hairpin RNAs. Nevertheless, these results suggest a way of monitoring current HIV-1 strains, even by low-scale deep sequencing of the six RNAi targets described in the patients from different countries, and to select the detected predominant variants of sequences for the targeted RNAi. In our preliminary experiments, we used the analysis of a limited number of cloned sequences and found targets that are present in the majority of viruses in cohort 1.^29^, ^30^, ^31^

The analysis of ultra-deep sequencing data of HIV-1 variants provides important information regarding the nature of RT. The nucleotide biases of this error-prone enzyme could be uncovered and may lead to a better understanding of the mechanisms responsible for the main source of HIV-1 variability. The HIV-1 genome is hypermutated by APOBEC3G in blood cells, but only a very small fraction of these mutations reach the plasma, indicating that many viruses are defective as a result of the extremely high mutation load and are incompatible with the release of viral particles.^15^ In our experiments, we used the HIV-1 variants that were released into the plasma. Although G-to-A transitions, which are characteristic consequences of APOBEC3G action, were observed in the range between 5.53 × 10^−5^ and 5.15 × 10^−3^, they were not predominant compared with other mutations that happened at much higher frequencies. For example, the most frequent transversion T to A reached 5.19 × 10^−2^ in A6 from cohort 2 (Figure 7), and the most frequent transition T to C was 1.67 × 10^−2^ in A3 from cohort 1 (Figure 6). The result is consistent with the recent conclusion that APOBEC3-induced G-to-A hypermutation provides only a small contribution to HIV-1 genetic variation in the viruses isolated from plasma.^16^ It cannot be excluded that RT also contributes to lethal mutations and the corresponding genomes cannot reach the plasma. Currently, we are performing a detailed analysis of the lethal mutations in the deep sequencing reads and mutations that are compatible with the release of viral particles (data to be published separately).

It is known that M184V and M184I mutations lead to drug resistance and, at the same time, dramatically decrease the processivity of RT.^38^ The patients from cohort 2 were treated with lamivudine for between 3 and 5 years. Surprisingly, we observed a very high level of the M184V mutation in cohort 1 (up to 9 × 10^−2^), which was not expected to contain a lot of drug-resistant HIV-1 variants because the corresponding patients were not receiving antiretroviral therapy. On the other hand, in cohort 2, which was supposed to contain drug-resistant variants of HIV-1, the M184V and M184I mutations were detected at frequencies of 3 × 10^−4^ and up to 2.3 × 10^−3^, respectively. Previously, it was shown that due to the mutational bias of RT, the M184I (ATA) variant in lamivudine-treated patients is usually observed first because there is a higher frequency of G-to-A substitutions toward the ATA codon than the A-to-G substitutions toward GTG (184V).^44^ However, later, due to the higher processivity of the M184V enzyme,^45^ the virus possessing this mutation outgrew. This is a possible explanation of the data observed in cohort 2. Nevertheless, the data strongly suggest that these mutations can emerge without antiretroviral therapy.

We observed that the most frequent nucleotide substitutions were transitions and that RT rather poorly discriminates between two variants of pyrimidines (T or C) or purines (A or G), which may explain the rapid reversions of M184V in the new host.^38^

The frequencies of nucleotide substitutions that were observed in the range of up to 10^−2^ inside 27- to 30-bp sequences still remain the mutation rate inside the core 19-nt sequences below 10^−5^. This fact strongly suggests that these regions have biological constraints for mutations, which is why we conclude that the selected regions could be used as the targets of RNAi.

HIV-1 mRNA may have secondary structures and this could restrict RNAi action. RNA-protein complexes could also mask a target sequence and prevent recognition by the corresponding siRNA. To address these questions, we will analyze in future studies the selected RNA targets further using a viral system, in which the virus will be isolated from the patients described in the current study.

## Materials and Methods

### RT-PCR and Preparation of Libraries

The RNA preparations were provided by the State Research Center of Virology and Biotechnology Vector (Russia) from their collection of isolates. Cohort 1 included five isolates of HIV-1 subtype A (10RU6587, 11RU6933, 11RU6949, 10RU6483, and 11RU1996) from patients who were not receiving antiretroviral therapy. Cohort 2 included four isolates (45788, 11; 2229, 13; MZ; and QN). The cohort was assumed to contain drug-resistant viruses. The patients from cohort 2 received antiretroviral therapy for 5 years and still possessed high-titer viremia and therefore the cohort was considered to include drug-resistant HIV-1 variants. Written informed consent was obtained from each participant. Blood samples were linked with demographic and clinical data via coded ID numbers according to the requirements of medical ethics in Russia. The study of clinical blood samples was in compliance with the ethical standards of the Helsinki Declaration of 1975 and the requirements of Model Law no. 26-10 “On the protection of human rights and dignity in biomedical research in the member states of the Commonwealth of Independent States” (October 18, 2005).

The RNA preparations were extracted individually from 500 μL of plasma samples using ViroSeq reagents (Celera Diagnostics) and then treated with DNase using a DNA-free kit (Ambion) as described previously.^46^, ^47^ The concentration of RNA preparations was measured using a NanoDrop 2000.

Five (cohort 1) or four (cohort 2) RNA preparations were pooled (6 ng of each individual RNA preparation) and used for RT-PCR. Approximately 15 ng of total RNA and M-MLV RT were used to synthesize cDNAs by use of a DNA-free kit (Ambion) according to the manufacturer’s instructions. cDNAs corresponding to two regions in the RT domain and to one region in the integrase domain from the *pol* gene as well as the conserved region from the *vpu* gene, the conserved region from the gp120 domain of the *env* gene, and the p17 domain from the *gag* gene were synthesized using the primers indicated in Table S2. Nested PCR was used for amplification of regions of approximately 300 bp that contained the six selected RNAi targets located at a distance of 20–50 bp from the 5′ end of the amplified DNA fragment. Primers were selected using the Primer Selection Tool (http://biotools.umassmed.edu/). The conditions for PCR for each set of primers were determined in preliminary experiments using a Mastercycler personal PCR instrument (Eppendorf). The identity of the amplified DNA fragments was confirmed by cloning and capillary sequencing.

Amplified DNA was used for the preparation of the library. Libraries were prepared according to Illumina’s instructions that accompany the DNA Sample Kit or the NEBNext Ultra DNA Library Prep Kit for Illumina. Briefly, DNA was end repaired using a combination of T4 DNA polymerase, *E. coli* DNA Pol I large fragment (Klenow polymerase), and T4 polynucleotide kinase. The blunt, phosphorylated ends were treated with Klenow fragment and dATP to yield a protruding 3′ A base for ligation of Illumina adapters, which have a single T base overhang at the 3′ end. After adaptor ligation, DNA was PCR amplified with Illumina primers for 15 cycles. Deep sequencing was performed using an Illumina Genome Analyzer IIx (cohort 1) or paired-end reads (2 × 150) produced by an Illumina HiSeq 1500 (cohort 2). The data were deposited in NCBI (Bioproject: PRJNA344431).

### Data Processing

The analysis of multiple related variants of rapidly evolving HIV-1 viruses with ultra-deep sequencing comprising more than 1 million reads can be performed with a bioinformatic pipeline (for a recent review, see Leipizig^48^ and Posada-Cespedesa et al.^49^). In a bioinformatic pipeline, the data are consecutively processed by an organized package of programs, such that the data at the output of one program are transferred to the input of another program. We tried several variants and finally created the following pipeline. The raw sequenced reads were first evaluated by their quality using FastQC.^50^ Then, the reads of too-short length (<20 nt) and low quality (q < 26) were filtered out from the initial set using Cutadapt.^51^ The invariable elements that could subsequently be used as a natural reference for the evaluation of mutation and microindel rates were searched for via a two-step iterative procedure. In the first step, the sequences for HIV-1 isolate 97CDKP58e from the Republic of the Congo (GenBank: AF316544) was used as a generic reference for HIV-1 subtype A sequences and aligned against a sampling set of 1,000 reads. The results of the alignment were analyzed by our own ad hoc Perl script to detect the most invariable sequence. In the second step, this sequence was used as the reference against the complete set of reads using Bowtie 2.^52^ All non-aligned reads were filtered out by SAMtools.^53^ The resulting alignment data were converted to Fasta format by UCSC Genome tools.^54^ All mutants detected by pairwise alignment of each read from the Fasta file against the reference target sequence were collected into an array suitable for further multiple alignments. The multiple alignments were performed using MAFFT software.^55^ To analyze the results of the multiple alignment and calculate the corresponding mutation rates and related statistics, we developed a Perl script in-house. This script has output options to visualize the results in terms of multiple alignments or phylogenetic trees using the Ugene toolkit.^56^

In our analysis, RNAi targets were appended by short fragments from both sides of each target, so the total length of the analyzed fragments was about 30 nt. For brevity, these longer fragments will also be termed RNAi targets. In this paper, the frequencies of nucleotide substitutions were determined against the sets of aligned sequences, which is reasonable in the case of deep sequencing data. The frequency of mutations in a particular site of the reference target (corresponding to the most invariable fragment as described above), $f_{N\to N^{\prime };\text{site}}$, was assessed against the set obtained by multiple alignment. The frequency of particular replacements, $N\to N^{\prime }$, N = (A, C, G, T), across the RNAi target was calculated as:

(Equation 1) $$f_{N\to N^{\prime }}=\frac{1}{n_{N}}\sum_{\mathrm{sites}}f_{N\to N^{\prime };\text{site}},$$

where *n*_*N*_ is the total number of nucleotides of type *N* within the RNAi target, and the summation in Equation 1 is performed across the sites occupied by the nucleotides of type *N*. The total frequency of mutations across the RNAi target was determined as:

(Equation 2) $$f_{m}=\sum_{N}\sum_{N^{\prime }\neq N}f_{N}f_{N\to N^{\prime }},$$

where $f_{N}=n_{N}/L_{\mathrm{target}}$ is the frequency of nucleotides of type *N* within the RNAi target, *L*_target_ is the target length, and $f_{N\to N^{\prime }}$ is defined by Equation 1.

The expected dispersion of replacements in a particular site may be assessed by binomial distribution^57^, ^58^

(Equation 3) $$\sigma_{\text{site}}^{2}=f_{N\to N^{\prime };\text{site}}\left(1-f_{N\to N^{\prime };\mathrm{site}}\right)/n_{seq},$$

where *n*_*seq*_ is the total number of the sequences in the multiple alignment set. For sensitivity of mutation detection, using the criterion

(Equation 4) $$1.96\sigma \left(f_{thr}\right)=f_{thr},$$

(Pr = 0.05) yields at the large *n*_*seq*_ the threshold detection rate

(Equation 5) $$f_{thr}\approx 3.84/n_{seq}.$$

This threshold determines the rate of reliable detection for replacement $N\to N^{\prime }$ at a particular site at the given number of aligned reads *n*_*seq*_. This estimate yields $f_{thr}\approx 3.8\times 10^{-6}$ for *n*_*seq*_ = 10^6^. The statistical significance between the counterpart replacements at a particular site for two different datasets (in our work, these are the sets for the cohorts of non-resistant [cohort 1] and drug-resistant patients [cohort 2]) can be assessed by the Gaussian *z*-criterion for the fractions.^57^ The similar criterion can also be applied to the statistical assessment of the difference between frequencies of replacements at different sites. As seen from Equation 2, at $f_{N\to N^{\prime };\text{site}}=10^{-4}$ and *n*_*seq*_ = 10^6^, the difference of approximately $\Delta f_{N\to N^{\prime };\text{site}}\approx 10^{-5}$ can be resolved between the counterpart replacements $N\to N^{\prime }$ at a particular site.

### Transfection Assays

The 27-bp targets shown in Table 3 were cloned into *Xho* I and *Not* I cloning sites inside the 3′ UTR of the *Renilla* gene of the psiCHECK-2 vector. Cultured HEK293T cells were plated 1 day prior to transfection (5 × 10^4^ cells per 24-well culture dish). For co-transfection experiments, Lipofectamine 3000 Transfection Reagent (ThermoFisher Scientific) was used according to the manufacturer’s instructions. Per well, we used 15 ng of the experimental DNA constructs containing a target of RNAi cloned into psiCHECK-2 mixed with 12.5 pmol of a Dicer substrate in 25 μL of serum-free medium (SFM). Lipofectamine reagent was mixed with 25 μL of SFM and mixed by vortex for 2–3 s. Both solutions were mixed and then 0.2 μL of P3000 reagent was added, and the final solution was mixed by vortex for 2–3 s and incubated for 5 min at room temperature. After the incubation, the solution was added to the cells in 500 μL of DMEM. The cells were incubated for 48 hr. Firefly and *Renilla* luciferase activities were measured using the Dual-Luciferase Reporter Assay System (Promega) and a Reporter Microplate Luminometer (Turner BioSystems). The *Renilla* luciferase data were normalized to the firefly luciferase data. Excel and Origin software were used for data analysis.

## Author Contributions

O.V.K. and N.A.T. conceived and supervised the study; D.M.F., O.V.K., N.A.T., M.A.G., N.M.G., and M.P.G. performed the experiments; V.R.C. and Y.V.K. analyzed data; and N.A.T. wrote the manuscript.

## Supplemental Information

Document S1. Figures S1–S13 and Table S1–S7

Document S2. Article plus Supplemental Information
