# Supplementary material for: Six Highly Conserved Targets of RNAi Revealed in HIV-1-Infected Patients from Russia Are Also Present in Many HIV-1 Strains Worldwide
Source: Mol Ther Nucleic Acids. 2017 Jul 13;8:330–44. doi: 10.1016/j.omtn.2017.07.010 (PMC5537207; doi:10.1016/j.omtn.2017.07.010)
Supplement: Document S1. Figures S1–S13 and Table S1–S7 [file mmc1.pdf]

OMTN, Volume *8*

Supplemental Information

Six Highly Conserved Targets of RNAi Revealed in HIV-1-Infected Patients from Russia Are Also Present in Many HIV-1 Strains Worldwide

Olga V. Kretova, Daria M. Fedoseeva, Maria A. Gorbacheva, Natalya M. Gashnikova, Maria P. Gashnikova, Nataliya V. Melnikova, Vladimir R. Chechetkin, Yuri V. Kravatsky, and Nickolai A. Tchurikov

Table S1. Percentage of 19-bp target invariability for different targets and cohorts.

	Cohort 1		Cohort 2	
**Target**	Aligned reads	Invariability, percent	Aligned reads	Invariability, percent
**A1**	1 291 242	99.17 ± 0.0080	8 390 187	97.71 ± 0.0052
**A2**	1 956 875	92.40 ± 0.0189	7 816 744	99.58 ± 0.0023
**A3**	1 418 446	96.84 ± 0.0147	4 862 688	96.68 ± 0.0081
**A4**	152 194	97.36 ± 0.0411	77 584	99.70 ± 0.0197
**A5**	951 233	97.14 ± 0.0171	8 181 119	96.57 ± 0.0064
**A6**	1 529 377	94.12 ± 0.0190	7 188 344	96.94 ± 0.0064

The invariability of 19-bp RNAi targets was assessed against the sets of aligned reads. The expected standard deviation was calculated according to Eq. (3) in the Materials and Methods section.

Table S2. Primers used for RT-PCR.

№	Text of primer (5'-3')	Used for:	Domain
**1**	TTTGATATGTCCATTGGTCTAGCCCTTGTT	Primer extension, (-) primer	RT-A1
**2**	CATCTATTGAGATGGGGATTTACCA	PCR-2, (+) primer	RT-A1
**3**	TGTTCTCTGCCAATTCCAATTCTG	PCR-2, (-) primer	RT-A1
**4**	CTCTGTTAGTGCTTTGGTCCCCCTAAGGAG	Primer extension, (-) primer	RT-A2
**5**	GACAAAGATCTTAGAGCCCTTTAGA	PCR-2, (+) primer	RT-A2
**6**	TATATCATTGACAGTCCAGCTTTCC	PCR-2, (-) primer	RT-A2
**7**	TTCTTGGTACTACCTTTATTTCGTTATTGTCTTG	Primer extension, (-) primer	Int-A3
**8**	AGAATTTGGAATTCCCTACAATCCC	PCR-2, (+) primer	Int-A3
**9**	TCTGCTGTCCCTGTAATAAACCC	PCR-2, (-) primer	Int-A3
**10**	CTAGAATCATTTCTTGTGGGTTGGGGTCTGTG	Primer extension, (-) primer	Vpu-A4
**11**	AGTAGGACTAATAGTAGCATTCATAG	PCR-2, (+) primer	Vpu-A4
**12**	TAATAGACTGTGACCCACAAGTTATTT	PCR-2, (-) primer	Vpu-A4
**13**	ACTACTGGCCTAATTCCATGTGTACATTGT	Primer extension, (-) primer	Gp120-A5
**14**	GACCCAACAACAATACAAGAAAAAGT	PCR-2, (+) primer	Gp120-A5
**15**	GTTGTATTGCAATAGAAAAATTCTCCT	PCR-2, (-) primer	Gp120-A5
**16**	TCCTAGGTGATATGGCCTGGTGTACCATTTG	Primer extension, (-) primer	P17-A6
**17**	TTGACTAGCGGAGGCTAGAAGG	PCR-2, (+) primer	P17-A6
**18**	ATTATGTAATGATTTAAGTTCTTCTGTT	PCR-2, (-) primer	P17-A6

Sequences were selected using the corresponding regions in AF316544 sequence. PCR primers are shown only for PCR-2.

Table S3. HIV BLAST results for target A1.

https://www.hiv.lanl.gov/content/sequence/BASIC_BLAST/basic_blast.html

HIV sequence database

HIV BLAST Results

BLAST Summary

200 sequences –13 different subtypes: A1, B, D, 02_AG, 02G, BF1, 01_AE, 08_BC, A1D, 35_AD, BC, 10_CD, C.

Query= seq1=A1 19 bp core sequence

Query	Acc	Description	Identity(%)
seq1	KY035111	HIV-1|name|seid 743558|-|US|2001|def	100
seq1	KY035251	HIV-1|name|seid 743418|-|US|2000|def	100
seq1	KY035957	HIV-1|name|seid 742712|-|US|2006|def	100
seq1	KY036939	HIV-1|name|seid 741730|-|US|2011|def	100
seq1	KY037212	HIV-1|name|seid 741457|-|US|2010|def	100
seq1	KY037546	HIV-1|name|seid 741123|-|US|2008|def	100
seq1	KY037705	HIV-1|name|seid 740964|-|US|2001|def	100
seq1	KX139396	HIV-1|name|seid 739853|-|NG|2012|def	100
seq1	KX944684	HIV-1|name|seid 739044|D|UG|2013|def	100
seq1	KX926891	HIV-1|name|seid 738677|B|US|2003|def	100
seq1	KX926901	HIV-1|name|seid 738667|B|US|2007|def	100
seq1	KX927025	HIV-1|name|seid 738543|B|US|2002|def	100
seq1	KY235817	HIV-1|name|seid 736976|-|UZ|2015|def	100
seq1	KY235854	HIV-1|name|seid 736939|A1|UZ|2015|def	100
seq1	KX790972	HIV-1|name|seid 728827|A1|KE|2012|def	100
seq1	KX791026	HIV-1|name|seid 728773|A1|KE|2012|def	100
seq1	KX661499	HIV-1|name|seid 728656|B|GB|2012|def	100
seq1	KX661727	HIV-1|name|seid 728428|B|GB|2010|def	100
seq1	KX662059	HIV-1|name|seid 728096|B|GB|2011|def	100

**seq1**	KX662388	HIV-1|name|seid 727767|A1|GB|2010|def	100
**seq1**	KX662716	HIV-1|name|seid 727439|B|GB|2008|def	100
**seq1**	KX887886	HIV-1|name|seid 725293|B|BR|2015|def	100
**seq1**	KX888007	HIV-1|name|seid 725172|B|BR|2015|def	100
**seq1**	KX888678	HIV-1|name|seid 724501|BF1|BR|2015|def	100
**seq1**	KX888825	HIV-1|name|seid 724354|B|BR|2015|def	100
**seq1**	KX782011	HIV-1|name|seid 721841|B|KR|2011|def	100
**seq1**	KU954716	HIV-1|name|seid 720993|BC|CN|2012|def	100
**seq1**	KX302419	HIV-1|name|seid 719272|A1|KE|2000|def	100
**seq1**	KX302561	HIV-1|name|seid 719236|A1|KE|2002|def	100
**seq1**	KU678028	HIV-1|name|seid 720075|B|US|2014|def	100
**seq1**	KU678034	HIV-1|name|seid 720069|B|US|2014|def	100
**seq1**	KT379823	HIV-1|name|seid 715951|B|CN|2008|def	100
**seq1**	KX018204	HIV-1|name|seid 714260|B|US|-|def	100
**seq1**	KX465662	HIV-1|name|seid 709717|B|DE|2007|def	100
**seq1**	KX466879	HIV-1|name|seid 708500|B|DE|2010|def	100
**seq1**	KX467092	HIV-1|name|seid 708287|02_AG|DE|2011|def	100
**seq1**	KU645853	HIV-1|name|seid 706221|A1|RU|2013|def	100
**seq1**	KU645876	HIV-1|name|seid 706198|A1|RU|2013|def	100
**seq1**	KU645878	HIV-1|name|seid 706196|A1|RU|2013|def	100
**seq1**	KU670329	HIV-1|name|seid 706182|A1|RU|2013|def	100
**seq1**	KX306421	HIV-1|name|seid 705386|A1|KE|2010|def	100
**seq1**	KX306437	HIV-1|name|seid 705370|A1|KE|2010|def	100
**seq1**	KU684966	HIV-1|name|seid 705032|02G|CM|2010|def	100
**seq1**	KJ474071	HIV-1|name|seid 701536|B|PA|2009|def	100
**seq1**	KJ474082	HIV-1|name|seid 701525|B|PA|2009|def	100
**seq1**	KJ474638	HIV-1|name|seid 700969|B|PA|2013|def	100
**seq1**	KT998288	HIV-1|name|seid	100

		700679|B|GF|2006|def	
**seq1**	KX128987	HIV-1|name|seid 698006|A1|-|-|def	100
**seq1**	KT713247	HIV-1|name|seid 693755|B|ES|1999|def	100
**seq1**	KR677204	HIV-1|name|seid 692773|02G|TG|2012|def	100
**seq1**	KU574443	HIV-1|name|seid 692197|B|AT|-|def	100
**seq1**	KU248505	HIV-1|name|seid 691230|A1|KE|2004|def	100
**seq1**	KU248535	HIV-1|name|seid 691200|A1|KE|2004|def	100
**seq1**	KU248537	HIV-1|name|seid 691198|A1|KE|2004|def	100
**seq1**	KU248573	HIV-1|name|seid 691162|A1|KE|2004|def	100
**seq1**	KU248656	HIV-1|name|seid 691079|A1|KE|2005|def	100
**seq1**	KU248678	HIV-1|name|seid 691057|D|KE|2005|def	100
**seq1**	KU248685	HIV-1|name|seid 691050|A1|KE|2005|def	100
**seq1**	KU248714	HIV-1|name|seid 691021|A1|KE|2005|def	100
**seq1**	KU644957	HIV-1|name|seid 690152|B|US|2000|def	100
**seq1**	KT863987	HIV-1|name|seid 688930|A1|BE|-|def	100
**seq1**	KT864319	HIV-1|name|seid 688598|02_AG|BE|-|def	100
**seq1**	KT869041	HIV-1|name|seid 688542|B|MX|2013|def	100
**seq1**	KT869070	HIV-1|name|seid 688513|B|MX|2014|def	100
**seq1**	KP877908	HIV-1|name|seid 683521|A1|KE|2011|def	100
**seq1**	KP877945	HIV-1|name|seid 683484|A1|KE|2011|def	100
**seq1**	KT347996	HIV-1|name|seid 685469|A1|UG|2010|def	100
**seq1**	KT348010	HIV-1|name|seid 685455|A1|UG|2010|def	100
**seq1**	KT348112	HIV-1|name|seid 685353|D|UG|2010|def	100
**seq1**	KT348170	HIV-1|name|seid 685295|D|UG|2010|def	100
**seq1**	KT348172	HIV-1|name|seid 685293|A1D|UG|2010|def	100
**seq1**	KU498429	HIV-1|name|seid 685128|A1|GB|2008|def	100
**seq1**	KU499327	HIV-1|name|seid 684230|D|GB|2008|def	100
**seq1**	KT427720	HIV-1|name|seid 676707|B|BR|2010|def	100

**seq1**	KJ396414	HIV-1|name|seid 673997|B|FR|2006|def	100
**seq1**	KT315997	HIV-1|name|seid 672788|A1|CD|2012|def	100
**seq1**	KT121452	HIV-1|name|seid 672226|A1|RU|2015|def	100
**seq1**	KT741420	HIV-1|name|seid 671822|B|BR|2009|def	100
**seq1**	KT746713	HIV-1|name|seid 666529|BF1|BR|2008|def	100
**seq1**	KT747846	HIV-1|name|seid 665396|BF1|BR|2009|def	100
**seq1**	KT748494	HIV-1|name|seid 664748|B|BR|2007|def	100
**seq1**	KT228902	HIV-1|name|seid 662873|A1|AU|2012|def	100
**seq1**	KT229271	HIV-1|name|seid 662504|B|AU|2004|def	100
**seq1**	KT370918	HIV-1|name|seid 660500|A1|KE|2009|def	100
**seq1**	KM985254	HIV-1|name|seid 658669|B|IL|2011|def	100
**seq1**	KR233334	HIV-1|name|seid 658545|A1|RU|2008|def	100
**seq1**	KR233337	HIV-1|name|seid 658542|A1|RU|2008|def	100
**seq1**	KT340116	HIV-1|name|seid 658309|B|PL|2014|def	100
**seq1**	KT340203	HIV-1|name|seid 658222|B|PL|2014|def	100
**seq1**	KM358093	HIV-1|name|seid 657667|B|US|2010|def	100
**seq1**	KT022364	HIV-1|name|seid 657563|A1|KE|2004|def	100
**seq1**	KF267601	HIV-1|name|seid 653027|01_AE|CN|2010|def	100
**seq1**	HG421598	HIV-1|name|seid 643451|08_BC|CN|2010|def	100
**seq1**	KP688157	HIV-1|name|seid 642702|B|CU|2008|def	100
**seq1**	KR188035	HIV-1|name|seid 641704|01_AE|CN|2013|def	100
**seq1**	KP738951	HIV-1|name|seid 640665|D|UG|2005|def	100
**seq1**	KP739008	HIV-1|name|seid 640608|D|UG|2005|def	100
**seq1**	KP739041	HIV-1|name|seid 640575|D|UG|2006|def	100
**seq1**	KP739061	HIV-1|name|seid 640555|D|UG|2006|def	100
**seq1**	KP739064	HIV-1|name|seid 640552|D|UG|2006|def	100
**seq1**	KP739159	HIV-1|name|seid 640457|D|UG|2005|def	100
**seq1**	KP739163	HIV-1|name|seid	100

		640453|D|UG|2005|def	
**seq1**	KP121155	HIV-1|name|seid 634664|BF1|TR|2014|def	100
**seq1**	KP121156	HIV-1|name|seid 634663|BF1|TR|2014|def	100
**seq1**	KP065817	HIV-1|name|seid 634520|B|US|2011|def	100
**seq1**	KP066341	HIV-1|name|seid 633996|B|US|2011|def	100
**seq1**	KP066658	HIV-1|name|seid 633679|B|US|2011|def	100
**seq1**	KP066826	HIV-1|name|seid 633511|B|US|2011|def	100
**seq1**	KP066871	HIV-1|name|seid 633466|B|US|2011|def	100
**seq1**	KP066888	HIV-1|name|seid 633449|B|US|2011|def	100
**seq1**	KP066892	HIV-1|name|seid 633445|B|US|2011|def	100
**seq1**	KP067002	HIV-1|name|seid 633335|B|US|2011|def	100
**seq1**	KP067016	HIV-1|name|seid 633321|B|US|2011|def	100
**seq1**	KP067017	HIV-1|name|seid 633320|B|US|2011|def	100
**seq1**	KP113072	HIV-1|name|seid 633238|B|US|2002|def	100
**seq1**	KP113084	HIV-1|name|seid 633226|B|US|2002|def	100
**seq1**	KP113095	HIV-1|name|seid 633215|B|US|2002|def	100
**seq1**	KP113101	HIV-1|name|seid 633209|B|US|2002|def	100
**seq1**	KP113362	HIV-1|name|seid 632948|B|US|2002|def	100
**seq1**	KP113371	HIV-1|name|seid 632939|B|US|2002|def	100
**seq1**	KP067025	HIV-1|name|seid 633312|B|US|2011|def	100
**seq1**	KP223814	HIV-1|name|seid 626395|A1|RW|2007|def	100
**seq1**	KP681733	HIV-1|name|seid 624614|A1D|TZ|-|def	100
**seq1**	KM851060	HIV-1|name|seid 621802|B|BR|2012|def	100
**seq1**	KF984060	HIV-1|name|seid 621504|C|IN|2012|def	100
**seq1**	KF544044	HIV-1|name|seid 621391|35_AD|IR|2011|def	100
**seq1**	KF544054	HIV-1|name|seid 621381|35_AD|IR|2011|def	100
**seq1**	KP235159	HIV-1|name|seid 620638|BC|CN|2009|def	100
**seq1**	KJ636003	HIV-1|name|seid 620524|A1|CY|2012|def	100

**seq1**	EU342764	HIV-1|name|seid 27683|10_CD|ES|2003|def	100
**seq1**	KF444838	HIV-1|name|seid 564447|-|GR|-|def	100
**seq1**	JX300509	HIV-1|name|seid 502888|B|HR|2006|def	100
**seq1**	JQ259171	HIV-1|name|seid 533260|01_AE|BG|2009|def	100
**seq1**	GQ268493	HIV-1|name|seid 370115|A1|GE|2007|def	100
**seq1**	JF929071	HIV-1|name|seid 443581|A1|ES|2007|def	100
**seq1**	GQ268440	HIV-1|name|seid 370168|A1|GE|2006|def	100
**seq1**	KC184386	HIV-1|name|seid 517927|A1|IL|2001|def	100
**seq1**	KP013647	HIV-1|name|seid 619050|B|SI|2013|def	100
**seq1**	KP013743	HIV-1|name|seid 618954|A1|SI|2000|def	100
**seq1**	KM395730	HIV-1|name|seid 617219|C|CN|2010|def	100
**seq1**	KM438266	HIV-1|name|seid 616652|A1D|TZ|2008|def	100
**seq1**	KM588946	HIV-1|name|seid 613836|01_AE|KW|2013|def	100
**seq1**	KM247296	HIV-1|name|seid 613632|A1|RU|1999|def	100
**seq1**	KM247299	HIV-1|name|seid 613629|A1|RU|1999|def	100
**seq1**	KJ870273	HIV-1|name|seid 613208|A1|RU|2010|def	100
**seq1**	KJ870346	HIV-1|name|seid 613135|A1|RU|2010|def	100
**seq1**	KJ870365	HIV-1|name|seid 613116|A1|RU|2011|def	100
**seq1**	KJ870435	HIV-1|name|seid 613046|A1|RU|2011|def	100
**seq1**	KJ870580	HIV-1|name|seid 612901|A1|RU|2012|def	100
**seq1**	KJ870603	HIV-1|name|seid 612878|A1|RU|2012|def	100
**seq1**	KJ870632	HIV-1|name|seid 612849|A1|RU|2013|def	100
**seq1**	KJ870677	HIV-1|name|seid 612804|A1|RU|2012|def	100
**seq1**	KJ870682	HIV-1|name|seid 612799|A1|RU|2013|def	100
**seq1**	KJ769488	HIV-1|name|seid 609954|B|US|2008|def	100
**seq1**	KF544096	HIV-1|name|seid 607114|A1|KE|2007|def	100
**seq1**	KF544137	HIV-1|name|seid 607073|A1|KE|2006|def	100
**seq1**	KF544200	HIV-1|name|seid	100

		607010|A1|KE|2006|def	
**seq1**	KJ769707	HIV-1|name|seid 606811|B|DE|2001|def	100
**seq1**	KJ769933	HIV-1|name|seid 606585|B|DE|2004|def	100
**seq1**	KJ770061	HIV-1|name|seid 606457|B|DE|2005|def	100
**seq1**	KJ770081	HIV-1|name|seid 606437|B|DE|2005|def	100
**seq1**	KJ770176	HIV-1|name|seid 606342|B|DE|2006|def	100
**seq1**	KJ770841	HIV-1|name|seid 605677|B|DE|2008|def	100
**seq1**	KJ771004	HIV-1|name|seid 605514|B|DE|2009|def	100
**seq1**	KJ771214	HIV-1|name|seid 605304|B|DE|2010|def	100
**seq1**	KJ771247	HIV-1|name|seid 605271|02_AG|DE|2010|def	100
**seq1**	KJ722815	HIV-1|name|seid 604627|B|US|2006|def	100
**seq1**	KJ722844	HIV-1|name|seid 604598|B|US|2001|def	100
**seq1**	KJ723386	HIV-1|name|seid 604056|B|US|2005|def	100
**seq1**	KJ722071	HIV-1|name|seid 603103|A1|RU|2011|def	100
**seq1**	KJ722084	HIV-1|name|seid 603090|A1|RU|2012|def	100
**seq1**	KJ906694	HIV-1|name|seid 601271|D|UG|2002|def	100
**seq1**	KJ906797	HIV-1|name|seid 601168|A1|UG|2006|def	100
**seq1**	KJ906853	HIV-1|name|seid 601112|A1|UG|2006|def	100
**seq1**	KJ906907	HIV-1|name|seid 601058|A1|UG|2007|def	100
**seq1**	KJ906911	HIV-1|name|seid 601054|A1|UG|2007|def	100
**seq1**	KJ907072	HIV-1|name|seid 600893|A1C|UG|2008|def	100
**seq1**	KJ907123	HIV-1|name|seid 600842|A1D|UG|2008|def	100
**seq1**	KJ907142	HIV-1|name|seid 600823|D|UG|2008|def	100
**seq1**	KJ502161	HIV-1|name|seid 597707|A1|KE|2013|def	100
**seq1**	KJ185225	HIV-1|name|seid 596817|C|IN|2012|def	100
**seq1**	KJ778897	HIV-1|name|seid 596143|0107|CN|2012|def	100
**seq1**	KC900666	HIV-1|name|seid 593064|A1|UG|2010|def	100
**seq1**	KC900683	HIV-1|name|seid 593047|A1|KE|2010|def	100

**seq1**	KC900692	HIV-1|name|seid 593038|A1|UG|2010|def	100
**seq1**	KC900699	HIV-1|name|seid 593031|A1C|KE|2009|def	100
**seq1**	KC900707	HIV-1|name|seid 593023|A1|UG|2010|def	100
**seq1**	KJ499570	HIV-1|name|seid 592525|A1|RU|2012|def	100
**seq1**	KF531125	HIV-1|name|seid 589772|A1|TZ|2005|def	100
**seq1**	KF531248	HIV-1|name|seid 589649|A1|TZ|2005|def	100
**seq1**	KF531275	HIV-1|name|seid 589622|A1|TZ|2005|def	100
**seq1**	KC681848	HIV-1|name|seid 592265|A1|RU|-|def	100
**seq1**	KF531361	HIV-1|name|seid 589536|A1|TZ|2005|def	100
**seq1**	KF531448	HIV-1|name|seid 589449|A1|TZ|2005|def	100
**seq1**	KF531484	HIV-1|name|seid 589413|A1|TZ|2005|def	100
**seq1**	KC560241	HIV-1|name|seid 587354|01_AE|TW|2012|def	100
**seq1**	KF135126	HIV-1|name|seid 587142|A1|IL|2009|def	100
**seq1**	KF720945	HIV-1|name|seid 583855|A1|AM|2009|def	100
**seq1**	KF576525	HIV-1|name|seid 582335|F2|CM|-|def	100
**seq1**	KF576588	HIV-1|name|seid 582272|A1G|CM|-|def	100

Table S4. HIV BLAST results for target A2.

https://www.hiv.lanl.gov/content/sequence/BASIC_BLAST/basic_blast.html

HIV sequence database

HIV BLAST Results

BLAST Summary

200 sequences – 16 different subtypes – D, B, A1, BF1, BC, 02_AG, 02G, A1D, 01AE, 35_AD, 10_CD, 01_AE , C, 0107, F2, A1G.

Query= seq1=A2 19 bp core sequence

Query	Acc	Description	Identity(%)
**seq1**	KY035111	HIV-1|name|seid 743558|-|US|2001|def	100

**seq1**	KY035251	HIV-1|name|seid 743418|-|US|2000|def	100
**seq1**	KY035957	HIV-1|name|seid 742712|-|US|2006|def	100
**seq1**	KY036939	HIV-1|name|seid 741730|-|US|2011|def	100
**seq1**	KY037212	HIV-1|name|seid 741457|-|US|2010|def	100
**seq1**	KY037546	HIV-1|name|seid 741123|-|US|2008|def	100
**seq1**	KY037705	HIV-1|name|seid 740964|-|US|2001|def	100
**seq1**	KX139396	HIV-1|name|seid 739853|-|NG|2012|def	100
**seq1**	KX944684	HIV-1|name|seid 739044|D|UG|2013|def	100
**seq1**	KX926891	HIV-1|name|seid 738677|B|US|2003|def	100
**seq1**	KX926901	HIV-1|name|seid 738667|B|US|2007|def	100
**seq1**	KX927025	HIV-1|name|seid 738543|B|US|2002|def	100
**seq1**	KY235817	HIV-1|name|seid 736976|-|UZ|2015|def	100
**seq1**	KY235854	HIV-1|name|seid 736939|A1|UZ|2015|def	100
**seq1**	KX790972	HIV-1|name|seid 728827|A1|KE|2012|def	100
**seq1**	KX791026	HIV-1|name|seid 728773|A1|KE|2012|def	100
**seq1**	KX661499	HIV-1|name|seid 728656|B|GB|2012|def	100
**seq1**	KX661727	HIV-1|name|seid 728428|B|GB|2010|def	100
**seq1**	KX662059	HIV-1|name|seid 728096|B|GB|2011|def	100
**seq1**	KX662388	HIV-1|name|seid 727767|A1|GB|2010|def	100
**seq1**	KX662716	HIV-1|name|seid 727439|B|GB|2008|def	100
**seq1**	KX887886	HIV-1|name|seid 725293|B|BR|2015|def	100
**seq1**	KX888007	HIV-1|name|seid 725172|B|BR|2015|def	100
**seq1**	KX888678	HIV-1|name|seid 724501|BF1|BR|2015|def	100
**seq1**	KX888825	HIV-1|name|seid 724354|B|BR|2015|def	100
**seq1**	KX782011	HIV-1|name|seid 721841|B|KR|2011|def	100
**seq1**	KU954716	HIV-1|name|seid 720993|BC|CN|2012|def	100
**seq1**	KX302419	HIV-1|name|seid 719272|A1|KE|2000|def	100
**seq1**	KX302561	HIV-1|name|seid	100

		719236|A1|KE|2002|def	
**seq1**	KU678028	HIV-1|name|seid 720075|B|US|2014|def	100
**seq1**	KU678034	HIV-1|name|seid 720069|B|US|2014|def	100
**seq1**	KT379823	HIV-1|name|seid 715951|B|CN|2008|def	100
**seq1**	KX018204	HIV-1|name|seid 714260|B|US|-|def	100
**seq1**	KX465662	HIV-1|name|seid 709717|B|DE|2007|def	100
**seq1**	KX466879	HIV-1|name|seid 708500|B|DE|2010|def	100
**seq1**	KX467092	HIV-1|name|seid 708287|02_AG|DE|2011|def	100
**seq1**	KU645853	HIV-1|name|seid 706221|A1|RU|2013|def	100
**seq1**	KU645876	HIV-1|name|seid 706198|A1|RU|2013|def	100
**seq1**	KU645878	HIV-1|name|seid 706196|A1|RU|2013|def	100
**seq1**	KU670329	HIV-1|name|seid 706182|A1|RU|2013|def	100
**seq1**	KX306421	HIV-1|name|seid 705386|A1|KE|2010|def	100
**seq1**	KX306437	HIV-1|name|seid 705370|A1|KE|2010|def	100
**seq1**	KU684966	HIV-1|name|seid 705032|02G|CM|2010|def	100
**seq1**	KJ474071	HIV-1|name|seid 701536|B|PA|2009|def	100
**seq1**	KJ474082	HIV-1|name|seid 701525|B|PA|2009|def	100
**seq1**	KJ474638	HIV-1|name|seid 700969|B|PA|2013|def	100
**seq1**	KT998288	HIV-1|name|seid 700679|B|GF|2006|def	100
**seq1**	KX128987	HIV-1|name|seid 698006|A1|-|-|def	100
**seq1**	KT713247	HIV-1|name|seid 693755|B|ES|1999|def	100
**seq1**	KR677204	HIV-1|name|seid 692773|02G|TG|2012|def	100
**seq1**	KU574443	HIV-1|name|seid 692197|B|AT|-|def	100
**seq1**	KU248505	HIV-1|name|seid 691230|A1|KE|2004|def	100
**seq1**	KU248535	HIV-1|name|seid 691200|A1|KE|2004|def	100
**seq1**	KU248537	HIV-1|name|seid 691198|A1|KE|2004|def	100
**seq1**	KU248573	HIV-1|name|seid 691162|A1|KE|2004|def	100
**seq1**	KU248656	HIV-1|name|seid 691079|A1|KE|2005|def	100

**seq1**	KU248678	HIV-1|name|seid 691057|D|KE|2005|def	100
**seq1**	KU248685	HIV-1|name|seid 691050|A1|KE|2005|def	100
**seq1**	KU248714	HIV-1|name|seid 691021|A1|KE|2005|def	100
**seq1**	KU644957	HIV-1|name|seid 690152|B|US|2000|def	100
**seq1**	KT863987	HIV-1|name|seid 688930|A1|BE|-|def	100
**seq1**	KT864319	HIV-1|name|seid 688598|02_AG|BE|-|def	100
**seq1**	KT869041	HIV-1|name|seid 688542|B|MX|2013|def	100
**seq1**	KT869070	HIV-1|name|seid 688513|B|MX|2014|def	100
**seq1**	KP877908	HIV-1|name|seid 683521|A1|KE|2011|def	100
**seq1**	KP877945	HIV-1|name|seid 683484|A1|KE|2011|def	100
**seq1**	KT347996	HIV-1|name|seid 685469|A1|UG|2010|def	100
**seq1**	KT348010	HIV-1|name|seid 685455|A1|UG|2010|def	100
**seq1**	KT348112	HIV-1|name|seid 685353|D|UG|2010|def	100
**seq1**	KT348170	HIV-1|name|seid 685295|D|UG|2010|def	100
**seq1**	KT348172	HIV-1|name|seid 685293|A1D|UG|2010|def	100
**seq1**	KU498429	HIV-1|name|seid 685128|A1|GB|2008|def	100
**seq1**	KU499327	HIV-1|name|seid 684230|D|GB|2008|def	100
**seq1**	KT427720	HIV-1|name|seid 676707|B|BR|2010|def	100
**seq1**	KJ396414	HIV-1|name|seid 673997|B|FR|2006|def	100
**seq1**	KT315997	HIV-1|name|seid 672788|A1|CD|2012|def	100
**seq1**	KT121452	HIV-1|name|seid 672226|A1|RU|2015|def	100
**seq1**	KT741420	HIV-1|name|seid 671822|B|BR|2009|def	100
**seq1**	KT746713	HIV-1|name|seid 666529|BF1|BR|2008|def	100
**seq1**	KT747846	HIV-1|name|seid 665396|BF1|BR|2009|def	100
**seq1**	KT748494	HIV-1|name|seid 664748|B|BR|2007|def	100
**seq1**	KT228902	HIV-1|name|seid 662873|A1|AU|2012|def	100
**seq1**	KT229271	HIV-1|name|seid 662504|B|AU|2004|def	100
**seq1**	KT370918	HIV-1|name|seid	100

		660500|A1|KE|2009|def	
**seq1**	KM985254	HIV-1|name|seid 658669|B|IL|2011|def	100
**seq1**	KR233334	HIV-1|name|seid 658545|A1|RU|2008|def	100
**seq1**	KR233337	HIV-1|name|seid 658542|A1|RU|2008|def	100
**seq1**	KT340116	HIV-1|name|seid 658309|B|PL|2014|def	100
**seq1**	KT340203	HIV-1|name|seid 658222|B|PL|2014|def	100
**seq1**	KM358093	HIV-1|name|seid 657667|B|US|2010|def	100
**seq1**	KT022364	HIV-1|name|seid 657563|A1|KE|2004|def	100
**seq1**	KF267601	HIV-1|name|seid 653027|01_AE|CN|2010|def	100
**seq1**	HG421598	HIV-1|name|seid 643451|08_BC|CN|2010|def	100
**seq1**	KP688157	HIV-1|name|seid 642702|B|CU|2008|def	100
**seq1**	KR188035	HIV-1|name|seid 641704|01_AE|CN|2013|def	100
**seq1**	KP738951	HIV-1|name|seid 640665|D|UG|2005|def	100
**seq1**	KP739008	HIV-1|name|seid 640608|D|UG|2005|def	100
**seq1**	KP739041	HIV-1|name|seid 640575|D|UG|2006|def	100
**seq1**	KP739061	HIV-1|name|seid 640555|D|UG|2006|def	100
**seq1**	KP739064	HIV-1|name|seid 640552|D|UG|2006|def	100
**seq1**	KP739159	HIV-1|name|seid 640457|D|UG|2005|def	100
**seq1**	KP739163	HIV-1|name|seid 640453|D|UG|2005|def	100
**seq1**	KP121155	HIV-1|name|seid 634664|BF1|TR|2014|def	100
**seq1**	KP121156	HIV-1|name|seid 634663|BF1|TR|2014|def	100
**seq1**	KP065817	HIV-1|name|seid 634520|B|US|2011|def	100
**seq1**	KP066341	HIV-1|name|seid 633996|B|US|2011|def	100
**seq1**	KP066658	HIV-1|name|seid 633679|B|US|2011|def	100
**seq1**	KP066826	HIV-1|name|seid 633511|B|US|2011|def	100
**seq1**	KP066871	HIV-1|name|seid 633466|B|US|2011|def	100
**seq1**	KP066888	HIV-1|name|seid 633449|B|US|2011|def	100
**seq1**	KP066892	HIV-1|name|seid 633445|B|US|2011|def	100

**seq1**	KP067002	HIV-1|name|seid 633335|B|US|2011|def	100
**seq1**	KP067016	HIV-1|name|seid 633321|B|US|2011|def	100
**seq1**	KP067017	HIV-1|name|seid 633320|B|US|2011|def	100
**seq1**	KP113072	HIV-1|name|seid 633238|B|US|2002|def	100
**seq1**	KP113084	HIV-1|name|seid 633226|B|US|2002|def	100
**seq1**	KP113095	HIV-1|name|seid 633215|B|US|2002|def	100
**seq1**	KP113101	HIV-1|name|seid 633209|B|US|2002|def	100
**seq1**	KP113362	HIV-1|name|seid 632948|B|US|2002|def	100
**seq1**	KP113371	HIV-1|name|seid 632939|B|US|2002|def	100
**seq1**	KP067025	HIV-1|name|seid 633312|B|US|2011|def	100
**seq1**	KP223814	HIV-1|name|seid 626395|A1|RW|2007|def	100
**seq1**	KP681733	HIV-1|name|seid 624614|A1D|TZ|-|def	100
**seq1**	KM851060	HIV-1|name|seid 621802|B|BR|2012|def	100
**seq1**	KF984060	HIV-1|name|seid 621504|C|IN|2012|def	100
**seq1**	KF544044	HIV-1|name|seid 621391|35_AD|IR|2011|def	100
**seq1**	KF544054	HIV-1|name|seid 621381|35_AD|IR|2011|def	100
**seq1**	KP235159	HIV-1|name|seid 620638|BC|CN|2009|def	100
**seq1**	KJ636003	HIV-1|name|seid 620524|A1|CY|2012|def	100
**seq1**	EU342764	HIV-1|name|seid 27683|10_CD|ES|2003|def	100
**seq1**	KF444838	HIV-1|name|seid 564447|-|GR|-|def	100
**seq1**	JX300509	HIV-1|name|seid 502888|B|HR|2006|def	100
**seq1**	JQ259171	HIV-1|name|seid 533260|01_AE|BG|2009|def	100
**seq1**	GQ268493	HIV-1|name|seid 370115|A1|GE|2007|def	100
**seq1**	JF929071	HIV-1|name|seid 443581|A1|ES|2007|def	100
**seq1**	GQ268440	HIV-1|name|seid 370168|A1|GE|2006|def	100
**seq1**	KC184386	HIV-1|name|seid 517927|A1|IL|2001|def	100
**seq1**	KP013647	HIV-1|name|seid 619050|B|SI|2013|def	100
**seq1**	KP013743	HIV-1|name|seid	100

		618954|A1|SI|2000|def	
**seq1**	KM395730	HIV-1|name|seid 617219|C|CN|2010|def	100
**seq1**	KM438266	HIV-1|name|seid 616652|A1D|TZ|2008|def	100
**seq1**	KM588946	HIV-1|name|seid 613836|01_AE|KW|2013|def	100
**seq1**	KM247296	HIV-1|name|seid 613632|A1|RU|1999|def	100
**seq1**	KM247299	HIV-1|name|seid 613629|A1|RU|1999|def	100
**seq1**	KJ870273	HIV-1|name|seid 613208|A1|RU|2010|def	100
**seq1**	KJ870346	HIV-1|name|seid 613135|A1|RU|2010|def	100
**seq1**	KJ870365	HIV-1|name|seid 613116|A1|RU|2011|def	100
**seq1**	KJ870435	HIV-1|name|seid 613046|A1|RU|2011|def	100
**seq1**	KJ870580	HIV-1|name|seid 612901|A1|RU|2012|def	100
**seq1**	KJ870603	HIV-1|name|seid 612878|A1|RU|2012|def	100
**seq1**	KJ870632	HIV-1|name|seid 612849|A1|RU|2013|def	100
**seq1**	KJ870677	HIV-1|name|seid 612804|A1|RU|2012|def	100
**seq1**	KJ870682	HIV-1|name|seid 612799|A1|RU|2013|def	100
**seq1**	KJ769488	HIV-1|name|seid 609954|B|US|2008|def	100
**seq1**	KF544096	HIV-1|name|seid 607114|A1|KE|2007|def	100
**seq1**	KF544137	HIV-1|name|seid 607073|A1|KE|2006|def	100
**seq1**	KF544200	HIV-1|name|seid 607010|A1|KE|2006|def	100
**seq1**	KJ769707	HIV-1|name|seid 606811|B|DE|2001|def	100
**seq1**	KJ769933	HIV-1|name|seid 606585|B|DE|2004|def	100
**seq1**	KJ770061	HIV-1|name|seid 606457|B|DE|2005|def	100
**seq1**	KJ770081	HIV-1|name|seid 606437|B|DE|2005|def	100
**seq1**	KJ770176	HIV-1|name|seid 606342|B|DE|2006|def	100
**seq1**	KJ770841	HIV-1|name|seid 605677|B|DE|2008|def	100
**seq1**	KJ771004	HIV-1|name|seid 605514|B|DE|2009|def	100
**seq1**	KJ771214	HIV-1|name|seid 605304|B|DE|2010|def	100
**seq1**	KJ771247	HIV-1|name|seid 605271|02_AG|DE|2010|def	100

**seq1**	KJ722815	HIV-1|name|seid 604627|B|US|2006|def	100
**seq1**	KJ722844	HIV-1|name|seid 604598|B|US|2001|def	100
**seq1**	KJ723386	HIV-1|name|seid 604056|B|US|2005|def	100
**seq1**	KJ722071	HIV-1|name|seid 603103|A1|RU|2011|def	100
**seq1**	KJ722084	HIV-1|name|seid 603090|A1|RU|2012|def	100
**seq1**	KJ906694	HIV-1|name|seid 601271|D|UG|2002|def	100
**seq1**	KJ906797	HIV-1|name|seid 601168|A1|UG|2006|def	100
**seq1**	KJ906853	HIV-1|name|seid 601112|A1|UG|2006|def	100
**seq1**	KJ906907	HIV-1|name|seid 601058|A1|UG|2007|def	100
**seq1**	KJ906911	HIV-1|name|seid 601054|A1|UG|2007|def	100
**seq1**	KJ907072	HIV-1|name|seid 600893|A1C|UG|2008|def	100
**seq1**	KJ907123	HIV-1|name|seid 600842|A1D|UG|2008|def	100
**seq1**	KJ907142	HIV-1|name|seid 600823|D|UG|2008|def	100
**seq1**	KJ502161	HIV-1|name|seid 597707|A1|KE|2013|def	100
**seq1**	KJ185225	HIV-1|name|seid 596817|C|IN|2012|def	100
**seq1**	KJ778897	HIV-1|name|seid 596143|0107|CN|2012|def	100
**seq1**	KC900666	HIV-1|name|seid 593064|A1|UG|2010|def	100
**seq1**	KC900683	HIV-1|name|seid 593047|A1|KE|2010|def	100
**seq1**	KC900692	HIV-1|name|seid 593038|A1|UG|2010|def	100
**seq1**	KC900699	HIV-1|name|seid 593031|A1C|KE|2009|def	100
**seq1**	KC900707	HIV-1|name|seid 593023|A1|UG|2010|def	100
**seq1**	KJ499570	HIV-1|name|seid 592525|A1|RU|2012|def	100
**seq1**	KF531125	HIV-1|name|seid 589772|A1|TZ|2005|def	100
**seq1**	KF531248	HIV-1|name|seid 589649|A1|TZ|2005|def	100
**seq1**	KF531275	HIV-1|name|seid 589622|A1|TZ|2005|def	100
**seq1**	KC681848	HIV-1|name|seid 592265|A1|RU|-|def	100
**seq1**	KF531361	HIV-1|name|seid 589536|A1|TZ|2005|def	100
**seq1**	KF531448	HIV-1|name|seid	100

		589449|A1|TZ|2005|def	
**seq1**	KF531484	HIV-1|name|seid 589413|A1|TZ|2005|def	100
**seq1**	KC560241	HIV-1|name|seid 587354|01_AE|TW|2012|def	100
**seq1**	KF135126	HIV-1|name|seid 587142|A1|IL|2009|def	100
**seq1**	KF720945	HIV-1|name|seid 583855|A1|AM|2009|def	100
**seq1**	KF576525	HIV-1|name|seid 582335|F2|CM|-|def	100
**seq1**	KF576588	HIV-1|name|seid 582272|A1G|CM|-|def	100

Table S5. HIV BLAST results for target A3.

https://www.hiv.lanl.gov/content/sequence/BASIC_BLAST/basic_blast.html

HIV sequence database

HIV BLAST Results

BLAST Summary

200 sequences – 12 different subtypes A1, 63_02A1, 63A1, D, 01_AE, BG, 02_AG, 14_BG, G, U, 37_cpx, A1BC, and SIV.

Query= seq1=A3 19 bp core sequence

Query	Acc	Description	Identity(%)
**seq1**	KU749400	HIV-1|name|seid 739023|A1|UA|2011|def	100
**seq1**	KX574417	HIV-1|name|seid 723997|63_02A1|RU|2015|def	100
**seq1**	KX574448	HIV-1|name|seid 723966|63A1|RU|2015|def	100
**seq1**	KX640210	HIV-1|name|seid 722783|A1|RU|2015|def	100
**seq1**	KX640233	HIV-1|name|seid 722760|A1|RU|2015|def	100
**seq1**	KF716491	HIV-1|name|seid 561754|A1|RU|2008|def	100
**seq1**	KJ415771	HIV-1|name|seid 593225|A1|AM|2009|def	100
**seq1**	AB870417	HIV-1|name|seid 571650|A1|JP|2012|def	100
**seq1**	HQ709849	HIV-1|name|seid 411685|A1|UG|2009|def	100
**seq1**	HQ709981	HIV-1|name|seid 411553|A1|UG|2009|def	100
**seq1**	HQ709994	HIV-1|name|seid 411540|A1|UG|2009|def	100
**seq1**	HQ710071	HIV-1|name|seid	100

		411463|A1|UG|2008|def	
**seq1**	HQ710187	HIV-1|name|seid 411347|D|UG|2008|def	100
**seq1**	AB253680	HIV-1|name|seid 102579|01_AE|JP|-|def	100
**seq1**	U57602	SIV|name|seid 238125|SAB|SN|1991|def	100
**seq1**	AB253651	HIV-1|name|seid 102608|01_AE|JP|-|def	100
**seq1**	AB253647	HIV-1|name|seid 102612|01_AE|JP|-|def	100
**seq1**	EF589042	HIV-1|name|seid 65712|A1|KZ|2002|def	100
**seq1**	EF589043	HIV-1|name|seid 65711|A1|KZ|2002|def	100
**seq1**	EU074774	HIV-1|name|seid 42123|BG|ES|2002|def	100
**seq1**	KX574407	HIV-1|name|seid 724007|63_02A1|RU|2015|def	100
**seq1**	KX574445	HIV-1|name|seid 723969|63_02A1|RU|2015|def	100
**seq1**	KC215175	HIV-1|name|seid 544754|A1|KZ|2009|def	100
**seq1**	JX202786	HIV-1|name|seid 522750|A1|UG|2007|def	100
**seq1**	HQ709985	HIV-1|name|seid 411549|A1|UG|2009|def	100
**seq1**	HQ710182	HIV-1|name|seid 411352|D|UG|2008|def	100
**seq1**	HM466988	HIV-1|name|seid 368941|A1|RU|2008|def	100
**seq1**	AF423755	HIV-1|name|seid 89445|BG|ES|1999|def	100
**seq1**	AF458242	HIV-1|name|seid 149881|A1|RW|1992|def	100
**seq1**	EU618551	HIV-1|name|seid 263944|02_AG|CM|1996|def	100
**seq1**	EU618518	HIV-1|name|seid 263977|02_AG|CM|1996|def	100
**seq1**	FJ670517	HIV-1|name|seid 333749|14_BG|ES|2004|def	100
**seq1**	GU217258	HIV-1|name|seid 336815|A1|GB|-|def	100
**seq1**	GU217004	HIV-1|name|seid 337069|A1|ES|2006|def	100
**seq1**	KY235915	HIV-1|name|seid 736878|A1|UZ|2013|def	100
**seq1**	KX574426	HIV-1|name|seid 723988|63_02A1|RU|2015|def	100
**seq1**	KX640200	HIV-1|name|seid 722793|A1|RU|2015|def	100
**seq1**	KX640217	HIV-1|name|seid 722776|A1|RU|2015|def	100
**seq1**	KC312920	HIV-1|name|seid 531946|A1|RU|2012|def	100

**seq1**	JX236669	HIV-1|name|seid 494843|A1|UG|2007|def	100
**seq1**	AF423759	HIV-1|name|seid 89441|14_BG|ES|2000|def	100
**seq1**	AY612637	HIV-1|name|seid 88145|G|PT|-|def	100
**seq1**	AB253421	HIV-1|name|seid 102981|A1|RW|1992|def	100
**seq1**	AB253673	HIV-1|name|seid 102586|01_AE|JP|-|def	100
**seq1**	AB253657	HIV-1|name|seid 102602|01_AE|JP|-|def	100
**seq1**	FJ465107	HIV-1|name|seid 258252|G|RU|2005|def	100
**seq1**	FJ388932	HIV-1|name|seid 283159|A1|CY|2005|def	100
**seq1**	GU362884	HIV-1|name|seid 368148|G|ES|2009|def	100
**seq1**	FJ670518	HIV-1|name|seid 333761|14_BG|ES|2004|def	100
**seq1**	FJ670527	HIV-1|name|seid 333754|DF1G|ES|2004|def	100
**seq1**	GU216925	HIV-1|name|seid 337148|G|ES|2005|def	100
**seq1**	DQ823356	HIV-1|name|seid 104698|A1|UA|2001|def	100
**seq1**	KU749402	HIV-1|name|seid 739021|A1|UA|2012|def	100
**seq1**	KX640229	HIV-1|name|seid 722764|A1|RU|2015|def	100
**seq1**	KX640235	HIV-1|name|seid 722758|A1|RU|2015|def	100
**seq1**	KJ415752	HIV-1|name|seid 593244|A1|AM|2009|def	100
**seq1**	KJ415754	HIV-1|name|seid 593242|A1|AM|2009|def	100
**seq1**	KF612672	HIV-1|name|seid 578014|A1|RU|2012|def	100
**seq1**	AB870695	HIV-1|name|seid 571368|B|JP|2011|def	100
**seq1**	KF716500	HIV-1|name|seid 561745|A1|UG|2003|def	100
**seq1**	JX202790	HIV-1|name|seid 522748|A1|UG|2007|def	100
**seq1**	JQ821358	HIV-1|name|seid 484432|A1|KG|2009|def	100
**seq1**	JQ292900	HIV-1|name|seid 462829|A1|RU|2006|def	100
**seq1**	HM466994	HIV-1|name|seid 368935|A1|UA|2008|def	100
**seq1**	AF413970	HIV-1|name|seid 142101|A1|UA|2000|def	100
**seq1**	AB253674	HIV-1|name|seid 102585|01_AE|JP|-|def	100
**seq1**	FJ465122	HIV-1|name|seid	100

		258237|G|RU|2005|def	
**seq1**	FJ388950	HIV-1|name|seid 283141|A1|CY|2006|def	100
**seq1**	FJ388951	HIV-1|name|seid 283140|A1|CY|2006|def	100
**seq1**	AY829203	HIV-1|name|seid 114155|A1|UZ|2002|def	100
**seq1**	DQ823358	HIV-1|name|seid 104696|A1|UA|2001|def	100
**seq1**	EU074780	HIV-1|name|seid 42117|BG|ES|2005|def	100
**seq1**	KY235909	HIV-1|name|seid 736884|A1|UZ|2013|def	100
**seq1**	KX574449	HIV-1|name|seid 723965|63A1|RU|2015|def	100
**seq1**	KJ197201	HIV-1|name|seid 610471|63_02A1|RU|2013|def	100
**seq1**	KJ415770	HIV-1|name|seid 593226|A1|AM|2009|def	100
**seq1**	KF612693	HIV-1|name|seid 577993|A1|RU|2011|def	100
**seq1**	KF648734	HIV-1|name|seid 564908|A1|KG|2010|def	100
**seq1**	KC215172	HIV-1|name|seid 544757|02_AG|KZ|2009|def	100
**seq1**	HQ710075	HIV-1|name|seid 411459|A1|UG|2008|def	100
**seq1**	HM215250	HIV-1|name|seid 398693|U|CA|2001|def	100
**seq1**	HM466985	HIV-1|name|seid 368944|A1|RU|2008|def	100
**seq1**	AF413972	HIV-1|name|seid 142099|A1|UA|2000|def	100
**seq1**	AB253677	HIV-1|name|seid 102582|01_AE|JP|-|def	100
**seq1**	AF450098	HIV-1|name|seid 89446|G|ES|1999|def	100
**seq1**	FJ465099	HIV-1|name|seid 258260|G|RU|2005|def	100
**seq1**	FJ670522	HIV-1|name|seid 333758|14_BG|ES|2005|def	100
**seq1**	GU217042	HIV-1|name|seid 337031|G|ES|2007|def	100
**seq1**	FJ183543	HIV-1|name|seid 2785|A1|DE|2007|def	100
**seq1**	KX574421	HIV-1|name|seid 723993|63_02A1|RU|2015|def	100
**seq1**	KX574440	HIV-1|name|seid 723974|63_02A1|RU|2015|def	100
**seq1**	KX574452	HIV-1|name|seid 723962|63A1|RU|2015|def	100
**seq1**	KX640198	HIV-1|name|seid 722795|A1|RU|2015|def	100
**seq1**	KX228801	HIV-1|name|seid 713516|G|ES|2009|def	100

**seq1**	KJ415756	HIV-1|name|seid 593240|A1|AM|2009|def	100
**seq1**	KJ415761	HIV-1|name|seid 593235|A1|AM|2009|def	100
**seq1**	KJ415767	HIV-1|name|seid 593229|A1|AM|2009|def	100
**seq1**	KJ415777	HIV-1|name|seid 593219|A1|AM|2009|def	100
**seq1**	KC681871	HIV-1|name|seid 592242|A1|RU|-|def	100
**seq1**	KF612661	HIV-1|name|seid 578025|A1|RU|2012|def	100
**seq1**	JX425832	HIV-1|name|seid 500733|A1G|FR|2008|def	100
**seq1**	JQ846266	HIV-1|name|seid 486199|A1|KG|2009|def	100
**seq1**	HQ714910	HIV-1|name|seid 415467|02_AG|CM|-|def	100
**seq1**	AY829211	HIV-1|name|seid 114147|A1|UZ|2002|def	100
**seq1**	FJ388892	HIV-1|name|seid 283199|A1|CY|2005|def	100
**seq1**	FJ465126	HIV-1|name|seid 258233|G|RU|2005|def	100
**seq1**	KU749399	HIV-1|name|seid 739024|A1|UA|2011|def	100
**seq1**	KU749404	HIV-1|name|seid 739019|A1|UA|2012|def	100
**seq1**	KX574406	HIV-1|name|seid 724008|63_02A1|RU|2015|def	100
**seq1**	KX640236	HIV-1|name|seid 722757|A1|RU|2015|def	100
**seq1**	AB870674	HIV-1|name|seid 571389|B|JP|2011|def	100
**seq1**	HM467005	HIV-1|name|seid 368924|A1|RU|2009|def	100
**seq1**	DQ823366	HIV-1|name|seid 104691|A1|UA|2001|def	100
**seq1**	FJ465119	HIV-1|name|seid 258240|G|RU|2005|def	100
**seq1**	FJ465105	HIV-1|name|seid 258254|G|RU|2005|def	100
**seq1**	EU618547	HIV-1|name|seid 263948|G|CM|1996|def	100
**seq1**	KY235916	HIV-1|name|seid 736877|A1|UZ|2013|def	100
**seq1**	KF747732	HIV-1|name|seid 614004|A1|RU|-|def	100
**seq1**	KF612690	HIV-1|name|seid 577996|A1|RU|2011|def	100
**seq1**	KC473837	HIV-1|name|seid 527351|14_BG|ES|2005|def	100
**seq1**	JX202785	HIV-1|name|seid 523017|A1|UG|2007|def	100
**seq1**	JX202795	HIV-1|name|seid	100

		523012|A1|UG|2007|def	
**seq1**	JX451923	HIV-1|name|seid 510265|14_BG|CH|2008|def	100
**seq1**	HQ709996	HIV-1|name|seid 411538|A1|UG|2009|def	100
**seq1**	HQ710069	HIV-1|name|seid 411465|A1|UG|2008|def	100
**seq1**	DQ460696	HIV-1|name|seid 116033|A2|KE|-|def	100
**seq1**	AB253422	HIV-1|name|seid 102980|A1|RW|1992|def	100
**seq1**	AB253671	HIV-1|name|seid 102588|01_AE|JP|-|def	100
**seq1**	AF457080	HIV-1|name|seid 189746|A1|KE|2000|def	100
**seq1**	FJ473401	HIV-1|name|seid 258430|G|RU|2005|def	100
**seq1**	FJ388903	HIV-1|name|seid 283188|A1|CY|2005|def	100
**seq1**	GU358383	HIV-1|name|seid 333723|G|FR|2009|def	100
**seq1**	DQ823360	HIV-1|name|seid 104694|A1|UA|2001|def	100
**seq1**	KY235920	HIV-1|name|seid 736873|-|UZ|2013|def	100
**seq1**	KX574418	HIV-1|name|seid 723996|63_02A1|RU|2015|def	100
**seq1**	KX574422	HIV-1|name|seid 723992|63_02A1|RU|2015|def	100
**seq1**	KX574430	HIV-1|name|seid 723984|63_02A1|RU|2015|def	100
**seq1**	KX640239	HIV-1|name|seid 722754|A1|RU|2015|def	100
**seq1**	KX640249	HIV-1|name|seid 722744|A1|RU|2015|def	100
**seq1**	KJ415732	HIV-1|name|seid 593264|A1|AM|2009|def	100
**seq1**	KJ415742	HIV-1|name|seid 593254|A1|AM|2009|def	100
**seq1**	KC681877	HIV-1|name|seid 592236|A1|RU|-|def	100
**seq1**	KF612669	HIV-1|name|seid 578017|A1|RU|2012|def	100
**seq1**	FJ183641	HIV-1|name|seid 2687|A1|DE|2007|def	100
**seq1**	HQ714911	HIV-1|name|seid 415466|02_AG|CM|-|def	100
**seq1**	HQ709993	HIV-1|name|seid 411541|A1|UG|2009|def	100
**seq1**	HM467012	HIV-1|name|seid 368917|A1|RU|2009|def	100
**seq1**	GU230137	HIV-1|name|seid 371432|14_BG|PT|2000|def	100
**seq1**	AF450096	HIV-1|name|seid 89448|14_BG|ES|2000|def	100

**seq1**	AB253676	HIV-1|name|seid 102583|01_AE|JP|-|def	100
**seq1**	AB253655	HIV-1|name|seid 102604|01_AE|JP|-|def	100
**seq1**	FV536612	synthetic DNA|name|seid 343931|-|-|-|def	100
**seq1**	FJ480285	HIV-1|name|seid 281559|G|CM|1996|def	100
**seq1**	GU216919	HIV-1|name|seid 337154|G|ES|2005|def	100
**seq1**	EU074775	HIV-1|name|seid 42122|BG|ES|2002|def	100
**seq1**	FJ388907	HIV-1|name|seid 283184|A1|CY|2005|def	100
**seq1**	EF589040	HIV-1|name|seid 65714|A1|KZ|2002|def	100
**seq1**	KX574432	HIV-1|name|seid 723982|63_02A1|RU|2015|def	100
**seq1**	KT022379	HIV-1|name|seid 657548|G|KE|2006|def	100
**seq1**	KJ415736	HIV-1|name|seid 593260|A1|AM|2009|def	100
**seq1**	KC681878	HIV-1|name|seid 592235|A1|RU|-|def	100
**seq1**	KC681865	HIV-1|name|seid 592248|A1|RU|-|def	100
**seq1**	AB869898	HIV-1|name|seid 572172|02_AG|JP|2012|def	100
**seq1**	KC215174	HIV-1|name|seid 544755|A1|KZ|2009|def	100
**seq1**	KC312894	HIV-1|name|seid 531959|A1|RU|2012|def	100
**seq1**	JX500699	HIV-1|name|seid 510190|63_02A1|RU|2011|def	100
**seq1**	JX425446	HIV-1|name|seid 500926|14_BG|FR|2008|def	100
**seq1**	JQ250602	HIV-1|name|seid 462122|02_AG|FR|2007|def	100
**seq1**	HQ714921	HIV-1|name|seid 415456|37_cpx|CM|-|def	100
**seq1**	HQ710073	HIV-1|name|seid 411461|A1|UG|2008|def	100
**seq1**	JF683780	HIV-1|name|seid 400209|A1|CY|2008|def	100
**seq1**	HM466979	HIV-1|name|seid 368950|A1|RU|2008|def	100
**seq1**	FJ465104	HIV-1|name|seid 258255|G|RU|2005|def	100
**seq1**	KU749405	HIV-1|name|seid 739018|A1|UA|2012|def	100
**seq1**	KX574415	HIV-1|name|seid 723999|63_02A1|RU|2015|def	100
**seq1**	KX574442	HIV-1|name|seid 723972|63_02A1|RU|2015|def	100
**seq1**	KF747731	HIV-1|name|seid	100

		614005|A1|RU|-|def	
**seq1**	KF747733	HIV-1|name|seid 614003|A1|RU|-|def	100
**seq1**	KJ415731	HIV-1|name|seid 593265|A1|AM|2009|def	100
**seq1**	KJ415764	HIV-1|name|seid 593232|A1|AM|2009|def	100
**seq1**	JQ292892	HIV-1|name|seid 462833|A1|RU|2002|def	100
**seq1**	HQ710078	HIV-1|name|seid 411456|A1|UG|2008|def	100
**seq1**	HQ710184	HIV-1|name|seid 411350|D|UG|2008|def	100
**seq1**	AB253678	HIV-1|name|seid 102581|01_AE|JP|-|def	100
**seq1**	DQ460695	HIV-1|name|seid 116034|A2|KE|-|def	100
**seq1**	AY829208	HIV-1|name|seid 114150|A1|UZ|2002|def	100
**seq1**	AF457077	HIV-1|name|seid 189749|A1|KE|2000|def	100
**seq1**	EU618229	HIV-1|name|seid 264266|G|CM|2002|def	100
**seq1**	EU618079	HIV-1|name|seid 264416|02_AG|CM|2003|def	100
**seq1**	KX640192	HIV-1|name|seid 722801|A1|RU|2015|def	100
**seq1**	KJ415768	HIV-1|name|seid 593228|A1|AM|2009|def	100
**seq1**	KC681875	HIV-1|name|seid 592238|A1|RU|-|def	100
**seq1**	AB869602	HIV-1|name|seid 572468|A1|JP|2010|def	100
**seq1**	KF716477	HIV-1|name|seid 561768|G|KE|2009|def	100
**seq1**	FR846409	HIV-1|name|seid 501646|G|PT|-|def	100
**seq1**	FJ183545	HIV-1|name|seid 2783|A1|DE|2006|def	100
**seq1**	HQ710183	HIV-1|name|seid 411351|D|UG|2008|def	100
**seq1**	HQ710189	HIV-1|name|seid 411345|D|UG|2008|def	100
**seq1**	AY857145	HIV-1|name|seid 79539|A1BC|AU|-|def	100
**seq1**	AF423756	HIV-1|name|seid 89444|14_BG|ES|1999|def	100

Table S6. HIV BLAST results for target A4.

https://www.hiv.lanl.gov/content/sequence/BASIC_BLAST/basic_blast.html

HIV sequence database

HIV BLAST Results

BLAST Summary

200 sequences – 11 different subtypes – A1, A1D, 01_AE, 35_AD, C, A1CD, 01A1, B, 11_cpx, 67_01B , 01B.

Query= seq1=A4 19 bp core sequence

Query	Acc	Description	Identity(%)
**seq1**	**KU749400**	**HIV-1|name|seid 739023|A1|UA|2011|def**	**100**
**seq1**	KT022364	HIV-1|name|seid 657563|A1|KE|2004|def	100
**seq1**	KT022399	HIV-1|name|seid 657528|A1D|KE|2005|def	100
**seq1**	KP223805	HIV-1|name|seid 626404|A1|RW|2007|def	100
**seq1**	JX203053	HIV-1|name|seid 522883|A1|UG|2007|def	100
**seq1**	JX203060	HIV-1|name|seid 522613|A1|UG|2007|def	100
**seq1**	JX447239	HIV-1|name|seid 498578|01_AE|TH|2009|def	100
**seq1**	AB703608	HIV-1|name|seid 496762|35_AD|IR|2010|def	100
**seq1**	HM027823	HIV-1|name|seid 363845|A1|UG|2007|def	100
**seq1**	GQ477442	HIV-1|name|seid 350590|35_AD|AF|2006|def	100
**seq1**	FJ623482	HIV-1|name|seid 336170|A1|KE|2006|def	100
**seq1**	FJ185243	HIV-1|name|seid 297884|01_AE|VN|1997|def	100
**seq1**	EU875277	HIV-1|name|seid 7122|A1|KE|1995|def	100
**seq1**	EU875216	HIV-1|name|seid 7183|A1|KE|2002|def	100
**seq1**	EU836515	HIV-1|name|seid 8283|A1|KE|2002|def	100
**seq1**	EU836462	HIV-1|name|seid 8336|A1|KE|1999|def	100
**seq1**	EU836448	HIV-1|name|seid 8350|A1|KE|1995|def	100
**seq1**	EU836335	HIV-1|name|seid 8463|A1|KE|1987|def	100
**seq1**	EU836339	HIV-1|name|seid 8459|A1|KE|1987|def	100
**seq1**	EU875237	HIV-1|name|seid 7162|A1|KE|2002|def	100
**seq1**	EF589042	HIV-1|name|seid 65712|A1|KZ|2002|def	100
**seq1**	KT012691	HIV-1|name|seid 653643|A1|IN|2012|def	100

**seq1**	KT152841	HIV-1|name|seid 643407|A1|IN|1999|def	100
**seq1**	KP223802	HIV-1|name|seid 626407|A1|RW|2007|def	100
**seq1**	KC681881	HIV-1|name|seid 592232|A1|RU|-|def	100
**seq1**	JX203056	HIV-1|name|seid 522615|A1|UG|2007|def	100
**seq1**	JX447073	HIV-1|name|seid 498661|01_AE|TH|2008|def	100
**seq1**	JN860764	HIV-1|name|seid 481249|01_AE|TH|2007|def	100
**seq1**	HM027871	HIV-1|name|seid 363797|A1D|UG|2003|def	100
**seq1**	AF069671	HIV-1|name|seid 223867|A1|SE|1994|def	100
**seq1**	FJ623483	HIV-1|name|seid 336169|A1|KE|2006|def	100
**seq1**	FJ185235	HIV-1|name|seid 297892|01_AE|VN|1998|def	100
**seq1**	AY358044	HIV-1|name|seid 163486|01_AE|TH|1999|def	100
**seq1**	EU875346	HIV-1|name|seid 7053|A1|KE|2002|def	100
**seq1**	EU875276	HIV-1|name|seid 7123|A1|KE|1995|def	100
**seq1**	EU875186	HIV-1|name|seid 7213|C|KE|1997|def	100
**seq1**	EU875108	HIV-1|name|seid 7252|A1|KE|1988|def	100
**seq1**	EU836329	HIV-1|name|seid 8469|A1|KE|1990|def	100
**seq1**	EU836357	HIV-1|name|seid 8441|A1|KE|1987|def	100
**seq1**	EF158040	HIV-1|name|seid 66098|35_AD|AF|2005|def	100
**seq1**	KJ190269	HIV-1|name|seid 585838|A1|RW|2007|def	100
**seq1**	KJ190273	HIV-1|name|seid 585834|A1|RW|2007|def	100
**seq1**	KF716470	HIV-1|name|seid 561775|A1CD|KE|2010|def	100
**seq1**	JX112800	HIV-1|name|seid 553671|01_AE|CN|2010|def	100
**seq1**	JX203210	HIV-1|name|seid 522754|A1|UG|2007|def	100
**seq1**	JX203052	HIV-1|name|seid 522617|A1|UG|2007|def	100
**seq1**	JX447056	HIV-1|name|seid 499595|01_AE|TH|2007|def	100
**seq1**	JX447076	HIV-1|name|seid 499585|01_AE|TH|2008|def	100
**seq1**	JX447311	HIV-1|name|seid 498542|01_AE|TH|2006|def	100
**seq1**	JX447285	HIV-1|name|seid	100

		498555|01_AE|TH|2007|def	
**seq1**	JN248355	HIV-1|name|seid 484374|01_AE|TH|2005|def	100
**seq1**	JF430899	HIV-1|name|seid 461794|A1|RU|2008|def	100
**seq1**	HQ616083	HIV-1|name|seid 456299|A1|RU|2008|def	100
**seq1**	HM027853	HIV-1|name|seid 363815|A1D|UG|2000|def	100
**seq1**	HM027877	HIV-1|name|seid 363791|A1D|UG|2003|def	100
**seq1**	AB253421	HIV-1|name|seid 102981|A1|RW|1992|def	100
**seq1**	AY905588	HIV-1|name|seid 122494|A1|KE|1987|def	100
**seq1**	AF457073	HIV-1|name|seid 189753|A1D|KE|1999|def	100
**seq1**	AF143901	HIV-1|name|seid 224230|A|TW|-|def	100
**seq1**	AF457070	HIV-1|name|seid 189756|A1|KE|2000|def	100
**seq1**	FJ623484	HIV-1|name|seid 336168|A1|KE|2006|def	100
**seq1**	FJ388953	HIV-1|name|seid 283138|01A1|CY|2006|def	100
**seq1**	FJ388932	HIV-1|name|seid 283159|A1|CY|2005|def	100
**seq1**	FJ866115	HIV-1|name|seid 300537|A1|KE|2005|def	100
**seq1**	FV536588	synthetic DNA|name|seid 343955|-|-|-|def	100
**seq1**	EU875305	HIV-1|name|seid 7094|A1D|KE|1996|def	100
**seq1**	EU875218	HIV-1|name|seid 7181|A1|KE|2002|def	100
**seq1**	EU836479	HIV-1|name|seid 8319|A1|KE|1997|def	100
**seq1**	EU836411	HIV-1|name|seid 8387|A1|KE|1996|def	100
**seq1**	DQ823356	HIV-1|name|seid 104698|A1|UA|2001|def	100
**seq1**	KM217988	HIV-1|name|seid 635602|01_AE|CN|2007|def	100
**seq1**	KP223849	HIV-1|name|seid 626360|A1|RW|2007|def	100
**seq1**	KC913687	HIV-1|name|seid 625159|01C|MM|2009|def	100
**seq1**	KJ190274	HIV-1|name|seid 585833|A1|RW|2007|def	100
**seq1**	KF716499	HIV-1|name|seid 561746|A1|RW|2003|def	100
**seq1**	JX203059	HIV-1|name|seid 522880|A1|UG|2007|def	100
**seq1**	JX203216	HIV-1|name|seid 522751|A1|UG|2007|def	100

**seq1**	JX203050	HIV-1|name|seid 522618|A1|UG|2007|def	100
**seq1**	JX203054	HIV-1|name|seid 522616|A1|UG|2007|def	100
**seq1**	JX203142	HIV-1|name|seid 522572|A1|UG|2007|def	100
**seq1**	JX447082	HIV-1|name|seid 499582|01_AE|TH|2005|def	100
**seq1**	JX447728	HIV-1|name|seid 499259|01_AE|TH|2009|def	100
**seq1**	JF430896	HIV-1|name|seid 461791|A1|RU|2008|def	100
**seq1**	HM027872	HIV-1|name|seid 363796|A1D|UG|2003|def	100
**seq1**	AF457086	HIV-1|name|seid 189740|A1|KE|2000|def	100
**seq1**	FJ388951	HIV-1|name|seid 283140|A1|CY|2006|def	100
**seq1**	FJ185250	HIV-1|name|seid 297877|01_AE|VN|1997|def	100
**seq1**	FJ866123	HIV-1|name|seid 300529|A1|KE|2005|def	100
**seq1**	AB485632	HIV-1|name|seid 312775|A1|UG|-|def	100
**seq1**	GU367592	HIV-1|name|seid 342455|B|US|-|def	100
**seq1**	EU875353	HIV-1|name|seid 7046|A1|KE|2002|def	100
**seq1**	EU875211	HIV-1|name|seid 7188|A1|KE|2002|def	100
**seq1**	EU836521	HIV-1|name|seid 8277|A1|KE|2002|def	100
**seq1**	EU836483	HIV-1|name|seid 8315|A1|KE|1995|def	100
**seq1**	EU836422	HIV-1|name|seid 8376|A1|KE|2000|def	100
**seq1**	EU836570	HIV-1|name|seid 9310|A1D|KE|1996|def	100
**seq1**	AY829203	HIV-1|name|seid 114155|A1|UZ|2002|def	100
**seq1**	DQ823358	HIV-1|name|seid 104696|A1|UA|2001|def	100
**seq1**	KP223801	HIV-1|name|seid 626408|A1|RW|2007|def	100
**seq1**	KP223822	HIV-1|name|seid 626387|A1|RW|2007|def	100
**seq1**	KP223840	HIV-1|name|seid 626369|A1|RW|2007|def	100
**seq1**	KP223848	HIV-1|name|seid 626361|A1|RW|2007|def	100
**seq1**	KC913647	HIV-1|name|seid 625199|01_AE|MM|2008|def	100
**seq1**	JX203057	HIV-1|name|seid 522881|A1|UG|2007|def	100
**seq1**	AF069670	HIV-1|name|seid	100

		223868|A1|SE|1994|def	
**seq1**	FJ623476	HIV-1|name|seid 336176|A1|KE|2006|def	100
**seq1**	EU875345	HIV-1|name|seid 7054|A1|KE|2002|def	100
**seq1**	EU875297	HIV-1|name|seid 7102|A1D|KE|1996|def	100
**seq1**	EU875298	HIV-1|name|seid 7101|A1D|KE|1996|def	100
**seq1**	EU836480	HIV-1|name|seid 8318|A1|KE|1994|def	100
**seq1**	EU836453	HIV-1|name|seid 8345|A1|KE|2002|def	100
**seq1**	EU836376	HIV-1|name|seid 8422|A1|KE|1995|def	100
**seq1**	EU110085	HIV-1|name|seid 14870|A1|KE|2001|def	100
**seq1**	KY042007	HIV-1|name|seid 720849|35_AD|IR|2012|def	100
**seq1**	KP109490	HIV-1|name|seid 626344|A1|UG|2009|def	100
**seq1**	JX447061	HIV-1|name|seid 498667|01_AE|TH|2007|def	100
**seq1**	HQ616082	HIV-1|name|seid 456290|A1|RU|-|def	100
**seq1**	AF413973	HIV-1|name|seid 142098|A1|UA|2000|def	100
**seq1**	AY829211	HIV-1|name|seid 114147|A1|UZ|2002|def	100
**seq1**	FJ388892	HIV-1|name|seid 283199|A1|CY|2005|def	100
**seq1**	AF127555	HIV-1|name|seid 211622|11_cpx|CM|1995|def	100
**seq1**	M62320	HIV-1|name|seid 252812|A1|UG|1985|def	100
**seq1**	FJ388925	HIV-1|name|seid 283166|A1|CY|2005|def	100
**seq1**	DQ083238	HIV-1|name|seid 119770|A1C|IN|2001|def	100
**seq1**	EU875352	HIV-1|name|seid 7047|A1|KE|2002|def	100
**seq1**	EU875300	HIV-1|name|seid 7099|A1D|KE|1996|def	100
**seq1**	EU836539	HIV-1|name|seid 8259|A1|KE|2004|def	100
**seq1**	EU836519	HIV-1|name|seid 8279|A1|KE|2002|def	100
**seq1**	EU836451	HIV-1|name|seid 8347|A1|KE|1995|def	100
**seq1**	EU836555	HIV-1|name|seid 8243|A1|KE|1989|def	100
**seq1**	KU749399	HIV-1|name|seid 739024|A1|UA|2011|def	100
**seq1**	KU749404	HIV-1|name|seid 739019|A1|UA|2012|def	100

**seq1**	KT022383	HIV-1|name|seid 657544|A1|KE|2006|def	100
**seq1**	KT152839	HIV-1|name|seid 643409|A1|IN|2009|def	100
**seq1**	KM217993	HIV-1|name|seid 635597|01_AE|CN|2007|def	100
**seq1**	KP223777	HIV-1|name|seid 626417|A1|RW|2007|def	100
**seq1**	KP223804	HIV-1|name|seid 626405|A1|RW|2007|def	100
**seq1**	KP223842	HIV-1|name|seid 626367|A1|RW|2007|def	100
**seq1**	KC913646	HIV-1|name|seid 625200|01_AE|MM|2008|def	100
**seq1**	JX203058	HIV-1|name|seid 522614|A1|UG|2007|def	100
**seq1**	JX203145	HIV-1|name|seid 522571|A1|UG|2007|def	100
**seq1**	JX447756	HIV-1|name|seid 499245|01_AE|TH|2007|def	100
**seq1**	JX448028	HIV-1|name|seid 499109|01_AE|TH|2008|def	100
**seq1**	JX447133	HIV-1|name|seid 498631|01_AE|TH|2006|def	100
**seq1**	HQ616093	HIV-1|name|seid 456295|A1|RU|2008|def	100
**seq1**	JF683779	HIV-1|name|seid 400210|A1|CY|2008|def	100
**seq1**	AB253428	HIV-1|name|seid 102974|A1|UG|1992|def	100
**seq1**	DQ823366	HIV-1|name|seid 104691|A1|UA|2001|def	100
**seq1**	AY945731	HIV-1|name|seid 106213|01_AE|TH|1999|def	100
**seq1**	AY284977	HIV-1|name|seid 110961|A|AR|1998|def	100
**seq1**	AY734554	HIV-1|name|seid 118541|A1C|TZ|2002|def	100
**seq1**	AF457087	HIV-1|name|seid 189739|A1C|KE|2000|def	100
**seq1**	AB098333	HIV-1|name|seid 176667|A1|UG|1992|def	100
**seq1**	FJ623485	HIV-1|name|seid 336167|A1|KE|2006|def	100
**seq1**	FJ623486	HIV-1|name|seid 336166|A1|KE|2006|def	100
**seq1**	FJ623477	HIV-1|name|seid 336175|A1|KE|2006|def	100
**seq1**	FJ185238	HIV-1|name|seid 297889|01_AE|VN|1997|def	100
**seq1**	EU875363	HIV-1|name|seid 7036|A1|KE|2002|def	100
**seq1**	EU875354	HIV-1|name|seid 7045|A1|KE|2002|def	100
**seq1**	EU875293	HIV-1|name|seid	100

		7106|A1D|KE|1996|def	
**seq1**	EU875240	HIV-1|name|seid 7159|A1|KE|2002|def	100
**seq1**	EU875235	HIV-1|name|seid 7164|A1|KE|2002|def	100
**seq1**	EU875212	HIV-1|name|seid 7187|A1|KE|2002|def	100
**seq1**	EU836527	HIV-1|name|seid 8271|A1|KE|2002|def	100
**seq1**	EU836345	HIV-1|name|seid 8453|A1D|KE|1987|def	100
**seq1**	EU875306	HIV-1|name|seid 7093|A1D|KE|1996|def	100
**seq1**	AF484512	HIV-1|name|seid 151375|A1|UG|1998|def	100
**seq1**	KU958484	SHIV|name|seid 697863|A1|-|-|def	100
**seq1**	KJ190263	HIV-1|name|seid 585844|A1|RW|2007|def	100
**seq1**	KJ190268	HIV-1|name|seid 585839|A1|RW|2007|def	100
**seq1**	KC183780	HIV-1|name|seid 527328|67_01B|CN|2011|def	100
**seq1**	JX203204	HIV-1|name|seid 522757|A1|UG|2007|def	100
**seq1**	JX203214	HIV-1|name|seid 522752|A1|UG|2007|def	100
**seq1**	JX447754	HIV-1|name|seid 499246|01_AE|TH|2007|def	100
**seq1**	JX448018	HIV-1|name|seid 499114|01B|TH|2005|def	100
**seq1**	JX447055	HIV-1|name|seid 498670|01_AE|TH|2007|def	100
**seq1**	JF683761	HIV-1|name|seid 400228|A1|CY|2007|def	100
**seq1**	HQ691074	HIV-1|name|seid 376832|01_AE|TH|2007|def	100
**seq1**	HQ691078	HIV-1|name|seid 376828|01_AE|TH|2007|def	100
**seq1**	HQ691079	HIV-1|name|seid 376827|01_AE|TH|2007|def	100
**seq1**	AF457052	HIV-1|name|seid 189774|A1|KE|2000|def	100
**seq1**	AB253422	HIV-1|name|seid 102980|A1|RW|1992|def	100
**seq1**	AY905603	HIV-1|name|seid 122479|A1D|KE|1987|def	100
**seq1**	AF457080	HIV-1|name|seid 189746|A1|KE|2000|def	100
**seq1**	FJ388903	HIV-1|name|seid 283188|A1|CY|2005|def	100
**seq1**	FJ647148	HIV-1|name|seid 282689|A1|ZA|2001|def	100
**seq1**	EU875349	HIV-1|name|seid 7050|A1|KE|2002|def	100

**seq1**	EU875351	HIV-1|name|seid 7048|A1|KE|2002|def	100
**seq1**	EU836424	HIV-1|name|seid 8374|A1|KE|1996|def	100
**seq1**	EU836338	HIV-1|name|seid 8460|A1|KE|1987|def	100
**seq1**	DQ823360	HIV-1|name|seid 104694|A1|UA|2001|def	100
**seq1**	EU836394	HIV-1|name|seid 8404|A1|KE|1996|def	100
**seq1**	EU875109	HIV-1|name|seid 7251|A1|KE|1988|def	100
**seq1**	DQ912822	HIV-1|name|seid 39195|A1D|DK|1996|def	100
**seq1**	KP223800	HIV-1|name|seid 626409|A1|RW|2007|def	100
**seq1**	KP223836	HIV-1|name|seid 626373|A1|RW|2007|def	100
**seq1**	KC913633	HIV-1|name|seid 625213|01_AE|MM|2008|def	100
**seq1**	JX203209	HIV-1|name|seid 523021|A1|UG|2007|def	100
**seq1**	JX203206	HIV-1|name|seid 522756|A1|UG|2007|def	100
**seq1**	JX203134	HIV-1|name|seid 522576|A1|UG|2007|def	100

Table S7. HIV BLAST results for target A6.

https://www.hiv.lanl.gov/content/sequence/BASIC_BLAST/basic_blast.html

HIV sequence database

HIV BLAST Results

BLAST Summary

200 sequences – 20 different subtypes - A1, A1D, C, B, G, 01_AE, F1, A1CD, A1C, 69_01B, 70_BF1, 58_01B, 56_cpx, 35_AD, 65_cpx, 01B, 01BC, BF1, 28_BF, 29_BF.

Query= seq1=A6 19 bp core sequence

Query	Acc	Description	Identity(%)
**seq1**	KU749400	HIV-1|name|seid 739023|A1|UA|2011|def	100
**seq1**	KU749413	HIV-1|name|seid 739010|-|PK|2014|def	100
**seq1**	KU921867	HIV-1|name|seid 721374|A1|KE|-|def	100
**seq1**	KU921873	HIV-1|name|seid	100

		721368|A1D|KE|-|def	
**seq1**	KU921923	HIV-1|name|seid 721318|A1CD|KE|-|def	100
**seq1**	KU921958	HIV-1|name|seid 721283|A1|KE|-|def	100
**seq1**	KU921991	HIV-1|name|seid 721250|A1|KE|-|def	100
**seq1**	KU922009	HIV-1|name|seid 721232|A1|KE|-|def	100
**seq1**	KU922059	HIV-1|name|seid 721182|C|KE|-|def	100
**seq1**	KU678028	HIV-1|name|seid 720075|B|US|2014|def	100
**seq1**	KU678034	HIV-1|name|seid 720069|B|US|2014|def	100
**seq1**	KX505399	HIV-1|name|seid 715720|B|US|2014|def	100
**seq1**	KX505580	HIV-1|name|seid 715539|B|US|2015|def	100
**seq1**	KU142283	HIV-1|name|seid 707613|B|GB|2007|def	100
**seq1**	KU142287	HIV-1|name|seid 707609|B|GB|2006|def	100
**seq1**	KU142301	HIV-1|name|seid 707595|B|GB|2005|def	100
**seq1**	KU142371	HIV-1|name|seid 707525|B|GB|2007|def	100
**seq1**	KU142448	HIV-1|name|seid 707448|B|GB|2006|def	100
**seq1**	KU142520	HIV-1|name|seid 707376|B|GB|2006|def	100
**seq1**	KU501257	HIV-1|name|seid 703551|01BC|CN|2013|def	100
**seq1**	KT124748	HIV-1|name|seid 703412|B|US|2001|def	100
**seq1**	KT124780	HIV-1|name|seid 703380|B|DE|2010|def	100
**seq1**	KT124787	HIV-1|name|seid 703373|B|DE|2012|def	100
**seq1**	KU685583	HIV-1|name|seid 693262|B|ES|2014|def	100
**seq1**	KT736703	HIV-1|name|seid 688254|C|ZA|2006|def	100
**seq1**	KT123093	HIV-1|name|seid 686827|G|PT|2007|def	100
**seq1**	KR811243	HIV-1|name|seid 684134|01_AE|CN|-|def	100
**seq1**	KR811265	HIV-1|name|seid 684112|01_AE|CN|-|def	100
**seq1**	KR811279	HIV-1|name|seid 684098|01_AE|CN|-|def	100
**seq1**	KU141042	HIV-1|name|seid 683733|B|US|-|def	100
**seq1**	KU168264	HIV-1|name|seid 685623|01_AE|-|2001|def	100

**seq1**	KT427659	HIV-1|name|seid 676768|B|BR|2010|def	100
**seq1**	KT427663	HIV-1|name|seid 676764|F1|BR|2010|def	100
**seq1**	KT427713	HIV-1|name|seid 676714|B|BR|2010|def	100
**seq1**	KT427720	HIV-1|name|seid 676707|B|BR|2010|def	100
**seq1**	KT427735	HIV-1|name|seid 676692|B|BR|2010|def	100
**seq1**	KT427760	HIV-1|name|seid 676667|BC|BR|2010|def	100
**seq1**	KT427789	HIV-1|name|seid 676638|BF1|BR|2010|def	100
**seq1**	KT339951	HIV-1|name|seid 674377|B|US|2008|def	100
**seq1**	KT339954	HIV-1|name|seid 674374|B|US|2005|def	100
**seq1**	LC100171	HIV-1|name|seid 662405|01_AE|VN|-|def	100
**seq1**	LC100194	HIV-1|name|seid 662382|01_AE|VN|-|def	100
**seq1**	LC100370	HIV-1|name|seid 662206|01_AE|VN|-|def	100
**seq1**	LC100442	HIV-1|name|seid 662134|01_AE|VN|-|def	100
**seq1**	LC100497	HIV-1|name|seid 662079|01_AE|VN|-|def	100
**seq1**	KT022364	HIV-1|name|seid 657563|A1|KE|2004|def	100
**seq1**	KT022399	HIV-1|name|seid 657528|A1D|KE|2005|def	100
**seq1**	KR020074	HIV-1|name|seid 656623|A1|KE|2008|def	100
**seq1**	KT191818	HIV-1|name|seid 656234|B|CN|2009|def	100
**seq1**	KT183246	HIV-1|name|seid 650893|C|ZA|2008|def	100
**seq1**	KT183260	HIV-1|name|seid 650879|C|ZA|2008|def	100
**seq1**	KT200355	HIV-1|name|seid 650790|B|ES|2010|def	100
**seq1**	KT200349	HIV-1|name|seid 650796|B|ES|2008|def	100
**seq1**	KR781619	HIV-1|name|seid 645081|A1|KE|-|def	100
**seq1**	KR781652	HIV-1|name|seid 645048|D|KE|1995|def	100
**seq1**	KR781722	HIV-1|name|seid 644978|A1|KE|1995|def	100
**seq1**	KR781738	HIV-1|name|seid 644962|A1|KE|1997|def	100
**seq1**	KR781778	HIV-1|name|seid 644922|D|KE|1994|def	100
**seq1**	KR781900	HIV-1|name|seid	100

		644800|A1|KE|2002|def	
**seq1**	KR781901	HIV-1|name|seid 644799|A1|KE|1995|def	100
**seq1**	KR781938	HIV-1|name|seid 644762|A1C|KE|1996|def	100
**seq1**	KR781973	HIV-1|name|seid 644727|A1D|KE|1997|def	100
**seq1**	KR782137	HIV-1|name|seid 644563|A1|KE|1995|def	100
**seq1**	KP223729	HIV-1|name|seid 626465|A1|RW|2007|def	100
**seq1**	KP223742	HIV-1|name|seid 626452|A1|RW|2007|def	100
**seq1**	KP223814	HIV-1|name|seid 626395|A1|RW|2007|def	100
**seq1**	KM192420	HIV-1|name|seid 626251|C|ZA|2008|def	100
**seq1**	KM192485	HIV-1|name|seid 626186|C|ZA|2008|def	100
**seq1**	KM192488	HIV-1|name|seid 626183|C|ZA|2008|def	100
**seq1**	KM192611	HIV-1|name|seid 626060|C|ZA|2009|def	100
**seq1**	KM192792	HIV-1|name|seid 625879|A1C|ZA|2010|def	100
**seq1**	KM192815	HIV-1|name|seid 625856|C|ZA|2010|def	100
**seq1**	KP056202	HIV-1|name|seid 625341|C|ZA|2008|def	100
**seq1**	LC027100	HIV-1|name|seid 625223|69_01B|JP|2005|def	100
**seq1**	KP208184	HIV-1|name|seid 624183|C|ZA|2012|def	100
**seq1**	KM484714	HIV-1|name|seid 617237|01_AE|CN|2013|def	100
**seq1**	KJ869481	HIV-1|name|seid 615984|B|DE|2004|def	100
**seq1**	KM243681	HIV-1|name|seid 615367|A1|IR|2012|def	100
**seq1**	KF818791	HIV-1|name|seid 613346|01_AE|CN|2010|def	100
**seq1**	KC913948	HIV-1|name|seid 611552|01_AE|MM|2008|def	100
**seq1**	KF701649	HIV-1|name|seid 608047|B|US|1985|def	100
**seq1**	KF701661	HIV-1|name|seid 608035|B|US|1985|def	100
**seq1**	KF701686	HIV-1|name|seid 608010|B|US|1985|def	100
**seq1**	KF701742	HIV-1|name|seid 607954|B|US|1987|def	100
**seq1**	KF701803	HIV-1|name|seid 607893|B|US|1988|def	100
**seq1**	KF701810	HIV-1|name|seid 607886|B|US|1989|def	100

**seq1**	KF701843	HIV-1|name|seid 607853|B|US|1988|def	100
**seq1**	KF701891	HIV-1|name|seid 607805|B|US|1984|def	100
**seq1**	KJ849801	HIV-1|name|seid 607375|B|BR|2010|def	100
**seq1**	KJ849809	HIV-1|name|seid 607367|70_BF1|BR|2010|def	100
**seq1**	KJ669905	HIV-1|name|seid 603539|B|NL|1991|def	100
**seq1**	KM048486	HIV-1|name|seid 600518|C|ZM|2008|def	100
**seq1**	KM048555	HIV-1|name|seid 600449|C|ZM|2006|def	100
**seq1**	KJ778897	HIV-1|name|seid 596143|0107|CN|2012|def	100
**seq1**	KF905959	HIV-1|name|seid 589068|C|ZA|2008|def	100
**seq1**	KF905968	HIV-1|name|seid 589059|C|ZA|2008|def	100
**seq1**	KF425293	HIV-1|name|seid 586799|58_01B|MY|2010|def	100
**seq1**	KJ190262	HIV-1|name|seid 585845|A1|RW|2007|def	100
**seq1**	AB873386	HIV-1|name|seid 573976|B|JP|-|def	100
**seq1**	AB873529	HIV-1|name|seid 573836|B|JP|-|def	100
**seq1**	AB873545	HIV-1|name|seid 573820|B|JP|-|def	100
**seq1**	KC852174	HIV-1|name|seid 564448|56_cpx|FR|2010|def	100
**seq1**	KF716480	HIV-1|name|seid 561765|D|UG|2011|def	100
**seq1**	KF526178	HIV-1|name|seid 561588|B|US|2011|def	100
**seq1**	KF208785	HIV-1|name|seid 559393|C|ZA|2010|def	100
**seq1**	JX112796	HIV-1|name|seid 553675|01_AE|CN|2010|def	100
**seq1**	JX112816	HIV-1|name|seid 553655|01_AE|CN|2007|def	100
**seq1**	KC797225	HIV-1|name|seid 552856|B|CH|2008|def	100
**seq1**	JX112856	HIV-1|name|seid 553615|01_AE|CN|2007|def	100
**seq1**	KC899333	HIV-1|name|seid 551375|B|FR|2012|def	100
**seq1**	KC149452	HIV-1|name|seid 543293|C|MW|2007|def	100
**seq1**	JQ302435	HIV-1|name|seid 542833|01_AE|CN|2009|def	100
**seq1**	JQ302372	HIV-1|name|seid 542642|01_AE|CN|2009|def	100
**seq1**	AB716197	HIV-1|name|seid	100

		539509|35_AD|IR|2010|def	
**seq1**	KC312514	HIV-1|name|seid 537345|B|US|2003|def	100
**seq1**	KC312532	HIV-1|name|seid 537336|B|US|2003|def	100
**seq1**	JX973394	HIV-1|name|seid 536646|B|US|2008|def	100
**seq1**	JX973448	HIV-1|name|seid 536619|B|US|2008|def	100
**seq1**	JX973480	HIV-1|name|seid 536603|B|US|2009|def	100
**seq1**	JX973492	HIV-1|name|seid 536597|B|US|2008|def	100
**seq1**	JX974044	HIV-1|name|seid 536321|C|ZA|2007|def	100
**seq1**	JX974114	HIV-1|name|seid 536286|C|ZA|2007|def	100
**seq1**	JX973347	HIV-1|name|seid 535614|B|US|2009|def	100
**seq1**	JX973433	HIV-1|name|seid 535571|B|US|2008|def	100
**seq1**	JX973493	HIV-1|name|seid 535541|B|US|2008|def	100
**seq1**	KC149259	HIV-1|name|seid 533919|C|MW|2008|def	100
**seq1**	HW060663	-|name|seid 533907|-|-|-|def	100
**seq1**	KC596063	HIV-1|name|seid 527345|01_AE|CN|2010|def	100
**seq1**	KC183778	HIV-1|name|seid 527329|65_cpx|CN|2011|def	100
**seq1**	JX203155	HIV-1|name|seid 523048|A1|UG|2007|def	100
**seq1**	JX202859	HIV-1|name|seid 522980|D|UG|2007|def	100
**seq1**	JX203156	HIV-1|name|seid 522781|A1|UG|2007|def	100
**seq1**	JX202904	HIV-1|name|seid 522691|D|UG|2007|def	100
**seq1**	JX202942	HIV-1|name|seid 522672|A1|UG|2007|def	100
**seq1**	JX202944	HIV-1|name|seid 522671|A1|UG|2007|def	100
**seq1**	JX446989	HIV-1|name|seid 498703|01_AE|TH|2007|def	100
**seq1**	JQ900864	HIV-1|name|seid 517273|01_AE|CN|2010|def	100
**seq1**	JQ900871	HIV-1|name|seid 517143|01_AE|CN|2009|def	100
**seq1**	JQ900891	HIV-1|name|seid 517133|B|CN|2008|def	100
**seq1**	JX960631	HIV-1|name|seid 510146|01_AE|CN|2009|def	100
**seq1**	JX960598	HIV-1|name|seid 510184|B|CN|2009|def	100
**seq1**	JX446646	HIV-1|name|seid	100

		499800|01_AE|TH|2008|def	
**seq1**	JX446850	HIV-1|name|seid 499698|01_AE|TH|2008|def	100
**seq1**	JX446852	HIV-1|name|seid 499697|01_AE|TH|2008|def	100
**seq1**	JX446872	HIV-1|name|seid 499687|01_AE|TH|2007|def	100
**seq1**	JX447018	HIV-1|name|seid 499614|01_AE|TH|2004|def	100
**seq1**	JX447170	HIV-1|name|seid 499538|01_AE|TH|2007|def	100
**seq1**	JX447422	HIV-1|name|seid 499412|01_AE|TH|2007|def	100
**seq1**	JX447442	HIV-1|name|seid 499402|01_AE|TH|2005|def	100
**seq1**	JX447352	HIV-1|name|seid 499447|01_AE|TH|2006|def	100
**seq1**	JX447460	HIV-1|name|seid 499393|01_AE|TH|2008|def	100
**seq1**	JX447628	HIV-1|name|seid 499309|01_AE|TH|2005|def	100
**seq1**	JX447676	HIV-1|name|seid 499285|01B|TH|2006|def	100
**seq1**	JX447762	HIV-1|name|seid 499242|01_AE|TH|2007|def	100
**seq1**	JX447768	HIV-1|name|seid 499239|01_AE|TH|2007|def	100
**seq1**	JX447866	HIV-1|name|seid 499190|01_AE|TH|2007|def	100
**seq1**	JX448012	HIV-1|name|seid 499117|01B|TH|2005|def	100
**seq1**	JX448236	HIV-1|name|seid 499005|01_AE|TH|2006|def	100
**seq1**	JX446763	HIV-1|name|seid 498816|01_AE|TH|2008|def	100
**seq1**	JX447573	HIV-1|name|seid 498411|01_AE|TH|2005|def	100
**seq1**	JX447763	HIV-1|name|seid 498316|01_AE|TH|2007|def	100
**seq1**	JX447793	HIV-1|name|seid 498301|B|TH|2006|def	100
**seq1**	JX447351	HIV-1|name|seid 498522|01_AE|TH|2006|def	100
**seq1**	JX448159	HIV-1|name|seid 498118|01_AE|TH|2005|def	100
**seq1**	JX448233	HIV-1|name|seid 498081|01_AE|TH|2006|def	100
**seq1**	JX448269	HIV-1|name|seid 498063|01_AE|TH|2005|def	100
**seq1**	AB703608	HIV-1|name|seid 496762|35_AD|IR|2010|def	100
**seq1**	JF932500	HIV-1|name|seid 496736|B|CN|2007|def	100
**seq1**	JF932477	HIV-1|name|seid 496731|B|CN|2007|def	100

**seq1**	JF932487	HIV-1|name|seid 496726|B|CN|2007|def	100
**seq1**	JX264248	HIV-1|name|seid 495195|01_AE|JP|1992|def	100
**seq1**	JX264372	HIV-1|name|seid 495133|B|JP|2007|def	100
**seq1**	JX264327	HIV-1|name|seid 494997|B|JP|2004|def	100
**seq1**	JX264345	HIV-1|name|seid 494988|B|JP|2005|def	100
**seq1**	JX264353	HIV-1|name|seid 494984|B|JP|2005|def	100
**seq1**	JX264359	HIV-1|name|seid 494981|B|JP|2006|def	100
**seq1**	JX264371	HIV-1|name|seid 494975|B|JP|2007|def	100
**seq1**	JX140678	HIV-1|name|seid 494863|A1B|ES|2009|def	100
**seq1**	JX244929	HIV-1|name|seid 494782|G|CM|2007|def	100
**seq1**	JQ229725	HIV-1|name|seid 494245|B|SE|2009|def	100
**seq1**	JQ229756	HIV-1|name|seid 492190|B|SE|2010|def	100
**seq1**	JN248347	HIV-1|name|seid 484378|B|TH|2005|def	100
**seq1**	JN248349	HIV-1|name|seid 484377|01B|TH|2005|def	100
**seq1**	JN685355	HIV-1|name|seid 479536|B|US|2007|def	100
**seq1**	JN685364	HIV-1|name|seid 479115|B|US|2006|def	100
**seq1**	JQ846108	HIV-1|name|seid 478300|B|ES|2007|def	100
**seq1**	JF905590	HIV-1|name|seid 477581|B|US|2005|def	100
**seq1**	JF905592	HIV-1|name|seid 477580|B|US|2006|def	100
**seq1**	JQ316134	HIV-1|name|seid 477599|B|KR|2005|def	100
**seq1**	JN014135	HIV-1|name|seid 473478|C|ZM|1998|def	100
**seq1**	JN014279	HIV-1|name|seid 473406|C|ZM|2002|def	100
**seq1**	JN014266	HIV-1|name|seid 472922|C|ZM|2002|def	100
**seq1**	JN014350	HIV-1|name|seid 472880|C|ZM|2000|def	100
**seq1**	JN014360	HIV-1|name|seid 472875|C|ZM|2000|def	100
**seq1**	JQ927640	HIV-1|name|seid 472584|B|US|2005|def	100
**seq1**	JF804812	HIV-1|name|seid 476613|28_BF|BR|2005|def	100
**seq1**	JF804807	HIV-1|name|seid	100

		476588|29_BF|BR|2005|def	
**seq1**	GQ430655	HIV-1|name|seid 319506|B|KE|1995|def	100
**seq1**	GQ430668	HIV-1|name|seid 319493|B|KE|1995|def	100
**seq1**	GQ432541	HIV-1|name|seid 317620|B|KE|1996|def	100

Figure S1. Complete alignment of deep sequencing reads for A1 (cohort 1).

The reference sequence is shown at the top (GenBank AF316544). The number and percentage of identical reads are indicated.

Target A1

Targets:

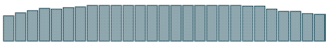

Amount (Percentage)	1	2	3	4	5	6	7	8	9	10	11	12	13	14	15	16	17	18	19	20	21	22	23	24	25	26	27
	a	a	a	a	a	g	c	A	t	c	a	g	a	a	a	g	a	a	c	c	t	c	c	a	t	t	t
AF316544	A	A	A	A	A	A	C	A	T	C	A	G	A	A	A	G	A	A	C	C	T	C	C	A	T	T	C
**1 181 049 (92.03)**	A	A	A	A	A	G	C	A	T	C	A	G	A	A	A	G	A	A	C	C	T	C	C	A	T	T	T
15 487 (1.21)	A	A	A	A	A	G	C	A	T	C	A	G	A	A	A	G	A	A	C	C	T	C	C	A	T	C	T
11 993 (0.94)	A	A	A	A	A	G	C	A	T	C	A	G	A	A	A	G	A	A	C	C	T	C	C	A	T	T	C
11 021 (0.86)	G	A	A	A	A	G	C	A	T	C	A	G	A	A	A	G	A	A	C	C	T	C	C	A	T	T	T
9 382 (0.73)	A	G	A	A	A	G	C	A	T	C	A	G	A	A	A	G	A	A	C	C	T	C	C	A	T	T	T
9 173 (0.71)	A	A	A	A	A	G	C	A	T	C	A	G	A	A	A	G	A	A	C	C	T	C	C	A	C	T	T
8 634 (0.67)	A	A	G	A	A	G	C	A	T	C	A	G	A	A	A	G	A	A	C	C	T	C	C	A	T	T	T
5 133 (0.40)	C	A	A	A	A	G	C	A	T	C	A	G	A	A	A	G	A	A	C	C	T	C	C	A	T	T	T
4 031 (0.31)	A	A	A	A	A	G	C	A	T	C	A	G	A	A	A	G	A	A	C	C	T	C	C	A	T	A	T
2 884 (0.23)	A	A	A	A	A	G	C	A	T	C	A	G	A	A	A	G	A	A	C	C	T	C	C	G	T	T	T
2 852 (0.22)	T	A	A	A	A	G	C	A	T	C	A	G	A	A	A	G	A	A	C	C	T	C	C	A	T	T	T
2 360 (0.18)	A	A	A	A	A	G	C	A	T	C	A	G	A	A	A	G	A	A	C	C	T	C	C	A	T	T	G
2 277 (0.18)	A	A	A	G	A	G	C	A	T	C	A	G	A	A	A	G	A	A	C	C	T	C	C	A	T	T	T
2 150 (0.17)	A	A	A	A	A	G	C	A	T	C	A	G	A	A	A	G	A	A	C	C	T	C	C	A	T	T	A
2 045 (0.16)	A	A	A	A	G	G	C	A	T	C	A	G	A	A	A	G	A	A	C	C	T	C	C	A	T	T	T
1 547 (0.12)	A	A	A	A	A	G	C	A	T	C	A	G	A	A	A	G	A	A	C	C	T	C	C	A	T	G	T
1 162 (0.09)	A	A	T	A	A	G	C	A	T	C	A	G	A	A	A	G	A	A	C	C	T	C	C	A	T	T	T
1 088 (0.09)	A	T	A	A	A	G	C	A	T	C	A	G	A	A	A	G	A	A	C	C	T	C	C	A	T	T	T
778 (0.06)	A	A	A	A	A	G	C	A	T	C	A	G	A	A	A	G	A	A	C	C	T	C	C	C	T	T	T
630 (0.05)	A	A	A	A	A	G	C	A	T	C	A	G	A	A	A	G	A	A	C	C	T	C	T	A	T	T	T
533 (0.04)	A	C	A	A	A	G	C	A	T	C	A	G	A	A	A	G	A	A	C	C	T	C	C	A	T	T	T
504 (0.04)	A	A	A	A	A	G	C	A	T	C	A	G	A	A	A	G	A	A	C	C	T	C	C	A	G	T	T
490 (0.04)	A	A	A	A	A	G	T	A	T	C	A	G	A	A	A	G	A	A	C	C	T	C	C	A	T	T	T
446 (0.04)	A	A	A	A	A	G	C	A	T	C	A	G	A	A	A	G	A	A	C	C	T	C	C	T	T	T	T
426 (0.03)	A	A	C	A	A	G	C	A	T	C	A	G	A	A	A	G	A	A	C	C	T	C	C	A	T	T	T
384 (0.03)	A	A	A	A	A	A	C	A	T	C	A	G	A	A	A	G	A	A	C	C	T	C	C	A	T	T	T
382 (0.03)	A	A	A	A	A	G	C	A	T	C	A	G	A	A	A	G	A	A	C	C	T	C	C	A	A	T	T
352 (0.03)	A	A	A	A	T	G	C	A	T	C	A	G	A	A	A	G	A	A	C	C	T	C	C	A	T	T	T
317 (0.03)	A	A	A	T	A	G	C	A	T	C	A	G	A	A	A	G	A	A	C	C	T	C	C	A	T	T	T
314 (0.02)	A	A	A	A	C	G	C	A	T	C	A	G	A	A	A	G	A	A	C	C	T	C	C	A	T	T	T
307 (0.02)	A	A	A	A	A	G	C	A	T	C	A	G	A	A	A	G	A	A	C	C	T	C	A	A	T	T	T
215 (0.02)	A	A	A	A	A	G	C	A	T	C	A	G	A	A	A	G	A	A	C	C	C	C	C	A	T	T	T
138 (0.01)	A	A	A	A	A	G	C	A	T	C	A	G	A	A	A	G	A	A	C	C	T	C	C	A	T	C	C
138 (0.01)	G	A	A	A	A	G	C	A	T	C	A	G	A	A	A	G	A	A	C	C	T	C	C	A	T	C	T
132 (0.01)	A	A	A	C	A	G	C	A	T	C	A	G	A	A	A	G	A	A	C	C	T	C	C	A	T	T	T
119 (0.01)	G	A	A	A	A	G	C	A	T	C	A	G	A	A	A	G	A	A	C	C	T	C	C	A	T	T	C
114 (0.01)	A	A	A	A	A	G	A	A	T	C	A	G	A	A	A	G	A	A	C	C	T	C	C	A	T	T	T
112 (0.01)	A	A	A	A	A	G	C	A	T	C	A	G	A	A	A	G	A	A	C	C	T	C	C	A	C	C	T
105 (0.01)	A	A	A	A	A	G	C	A	T	C	A	G	A	A	A	G	A	A	C	C	T	C	G	A	T	T	T
91 (0.01)	A	A	A	A	A	G	C	A	T	C	A	G	A	A	A	G	A	A	C	C	T	T	C	A	T	T	T
89 (0.01)	G	A	G	A	A	G	C	A	T	C	A	G	A	A	A	G	A	A	C	C	T	C	C	A	T	T	T
83 (0.01)	A	G	A	A	A	G	C	A	T	C	A	G	A	A	A	G	A	A	C	C	T	C	C	A	T	C	T
73 (0.01)	A	A	A	A	A	G	C	A	T	C	A	G	A	A	A	G	A	A	C	C	T	C	C	A	C	T	C
69 (0.01)	A	G	A	A	A	G	C	A	T	C	A	G	A	A	A	G	A	A	C	C	T	C	C	A	T	T	C
59 (0.01)	A	A	A	A	A	G	C	A	T	C	A	G	A	A	A	G	A	A	C	C	T	C	-	A	T	T	T
55 (0.00)	A	G	G	A	A	G	C	A	T	C	A	G	A	A	A	G	A	A	C	C	T	C	C	A	T	T	T
54 (0.00)	C	A	A	A	A	G	C	A	T	C	A	G	A	A	A	G	A	A	C	C	T	C	C	A	T	T	C
52 (0.00)	G	G	A	A	A	G	C	A	T	C	A	G	A	A	A	G	A	A	C	C	T	C	C	A	T	T	T
49 (0.00)	C	A	A	A	A	G	C	A	T	C	A	G	A	A	A	G	A	A	C	C	T	C	C	A	T	C	T
46 (0.00)	A	A	A	A	A	G	C	A	T	C	A	G	A	A	A	G	A	A	C	C	A	C	C	A	T	T	T
41 (0.00)	A	A	A	A	A	C	C	A	T	C	A	G	A	A	A	G	A	A	C	C	T	C	C	A	T	T	T
37 (0.00)	A	A	A	A	A	T	C	A	T	C	A	G	A	A	A	G	A	A	C	C	T	C	C	A	T	T	T
36 (0.00)	C	A	G	A	A	G	C	A	T	C	A	G	A	A	A	G	A	A	C	C	T	C	C	A	T	T	T
36 (0.00)	A	A	A	A	A	G	C	A	T	C	A	G	A	A	A	G	A	A	C	C	T	C	C	A	T	A	C
35 (0.00)	G	A	A	A	A	G	C	A	T	C	A	G	A	A	A	G	A	A	C	C	T	C	C	A	C	T	T
33 (0.00)	A	A	A	A	A	G	C	A	T	C	A	G	A	A	A	G	A	A	C	C	T	C	C	A	C	A	T
32 (0.00)	A	A	A	A	A	G	C	A	T	C	A	G	A	A	A	G	A	A	C	C	T	C	C	G	T	C	T
32 (0.00)	C	G	A	A	A	G	C	A	T	C	A	G	A	A	A	G	A	A	C	C	T	C	C	A	T	T	T
31 (0.00)	A	A	A	A	A	G	C	A	T	C	A	G	A	A	A	G	A	A	C	C	T	A	C	A	T	T	T
27 (0.00)	A	A	A	A	A	G	G	A	T	C	A	G	A	A	A	G	A	A	C	C	T	C	C	A	T	T	T
26 (0.00)	A	A	A	A	A	G	C	A	T	C	A	G	A	A	A	G	A	A	C	C	G	C	C	A	T	T	T
26 (0.00)	G	A	A	A	A	G	C	A	T	C	A	G	A	A	A	G	A	A	C	C	T	C	C	A	T	T	A
26 (0.00)	G	A	A	A	A	G	C	A	T	C	A	G	A	A	A	G	A	A	C	C	T	C	C	A	T	A	T
25 (0.00)	A	A	A	A	A	G	C	A	T	C	A	G	A	A	A	G	A	A	C	C	T	C	C	A	T	C	G
23 (0.00)	T	A	A	A	A	G	C	A	T	C	A	G	A	A	A	G	A	A	C	C	T	C	C	A	T	C	T
23 (0.00)	T	A	A	A	A	G	C	A	T	C	A	G	A	A	A	G	A	A	C	C	T	C	C	A	T	T	C
22 (0.00)	A	A	A	A	A	G	C	A	T	C	A	G	A	A	A	G	A	A	C	C	T	C	C	A	T	C	A
21 (0.00)	A	A	A	A	A	G	C	A	T	C	A	G	A	A	A	G	A	A	C	C	T	C	C	A	C	T	A
21 (0.00)	C	T	A	A	A	G	C	A	T	C	A	G	A	A	A	G	A	A	C	C	T	C	C	A	T	T	T
21 (0.00)	A	G	A	G	A	G	C	A	T	C	A	G	A	A	A	G	A	A	C	C	T	C	C	A	T	T	T
19 (0.00)	A	G	A	A	A	G	C	A	T	C	A	G	A	A	A	G	A	A	C	C	T	C	C	A	T	T	A
18 (0.00)	A	A	A	A	A	G	C	A	T	C	A	G	A	A	A	G	A	A	C	C	T	C	C	A	C	G	T
18 (0.00)	A	A	A	A	A	G	C	A	T	C	A	G	A	A	A	G	A	A	C	C	T	C	C	A	C	T	G
18 (0.00)	G	A	A	G	A	G	C	A	T	C	A	G	A	A	A	G	A	A	C	C	T	C	C	A	T	T	T
17 (0.00)	A	A	A	A	A	G	C	A	T	C	A	G	A	A	A	G	A	A	C	C	T	C	C	G	C	T	T
17 (0.00)	G	T	A	A	A	G	C	A	T	C	A	G	A	A	A	G	A	A	C	C	T	C	C	A	T	T	T
16 (0.00)	A	A	A	A	A	G	C	A	T	C	A	G	A	A	A	G	A	A	C	C	T	C	C	G	T	T	C
16 (0.00)	T	G	A	A	A	G	C	A	T	C	A	G	A	A	A	G	A	A	C	C	T	C	C	A	T	T	T
16 (0.00)	G	A	A	A	A	G	C	A	T	C	A	G	A	A	A	G	A	A	C	C	T	C	C	A	T	T	G
15 (0.00)	T	A	G	A	A	G	C	A	T	C	A	G	A	A	A	G	A	A	C	C	T	C	C	A	T	T	T
14 (0.00)	C	A	A	A	A	G	C	A	T	C	A	G	A	A	A	G	A	A	C	C	T	C	C	A	T	T	A
13 (0.00)	A	A	A	A	A	G	C	A	T	C	A	G	A	A	A	G	A	A	C	C	T	C	C	A	A	C	T
13 (0.00)	G	A	A	A	G	G	C	A	T	C	A	G	A	A	A	G	A	A	C	C	T	C	C	A	T	T	T
13 (0.00)	A	A	A	A	A	G	C	A	T	C	A	G	A	A	A	G	A	A	C	C	T	C	C	A	T	A	G
12 (0.00)	G	A	A	A	A	G	C	A	T	C	A	G	A	A	A	G	A	A	C	C	T	C	C	A	T	G	T
11 (0.00)	A	A	G	G	A	G	C	A	T	C	A	G	A	A	A	G	A	A	C	C	T	C	C	A	T	T	T
11 (0.00)	C	A	A	A	A	G	C	A	T	C	A	G	A	A	A	G	A	A	C	C	T	C	C	A	C	T	T
11 (0.00)	G	A	A	A	A	G	C	A	T	C	A	G	A	A	A	G	A	A	C	C	T	C	C	G	T	T	T
10 (0.00)	A	G	A	A	A	G	C	A	T	C	A	G	A	A	A	G	A	A	C	C	T	C	C	A	T	A	T
10 (0.00)	A	G	T	A	A	G	C	A	T	C	A	G	A	A	A	G	A	A	C	C	T	C	C	A	T	T	T
10 (0.00)	A	G	A	A	A	G	C	A	T	C	A	G	A	A	A	G	A	A	C	C	T	C	C	A	C	T	T
10 (0.00)	A	A	G	A	A	G	C	A	T	C	A	G	A	A	A	G	A	A	C	C	T	C	C	A	T	T	C
9 (0.00)	A	G	A	A	A	G	C	A	T	C	A	G	A	A	A	G	A	A	C	C	T	C	C	A	T	T	G
8 (0.00)	A	A	A	A	A	G	C	A	T	C	A	G	A	A	A	G	A	A	C	C	T	C	C	A	T	G	C
8 (0.00)	A	A	A	G	G	G	C	A	T	C	A	G	A	A	A	G	A	A	C	C	T	C	C	A	T	T	T
8 (0.00)	C	A	A	A	A	G	C	A	T	C	A	G	A	A	A	G	A	A	C	C	T	C	C	A	T	A	T
7 (0.00)	T	A	A	A	G	G	C	A	T	C	A	G	A	A	A	G	A	A	C	C	T	C	C	A	T	T	T
7 (0.00)	A	A	A	A	A	G	C	A	T	C	A	G	A	A	A	G	A	A	C	C	T	C	C	A	G	C	T
7 (0.00)	T	A	A	A	A	G	C	A	T	C	A	G	A	A	A	G	A	A	C	C	T	C	C	A	T	T	G
7 (0.00)	A	A	A	A	A	G	C	A	T	C	A	G	A	A	A	G	A	A	A	T	G	G	A	G	G	T	T
6 (0.00)	T	A	A	A	A	G	C	A	T	C	A	G	A	A	A	G	A	A	C	C	T	C	C	A	T	A	T
6 (0.00)	T	A	A	G	A	G	C	A	T	C	A	G	A	A	A	G	A	A	C	C	T	C	C	A	T	T	T
6 (0.00)	T	A	T	A	A	G	C	A	T	C	A	G	A	A	A	G	A	A	C	C	T	C	C	A	T	T	T
6 (0.00)	G	A	T	A	A	G	C	A	T	C	A	G	A	A	A	G	A	A	C	C	T	C	C	A	T	T	T
6 (0.00)	A	T	G	A	A	G	C	A	T	C	A	G	A	A	A	G	A	A	C	C	T	C	C	A	T	T	T
6 (0.00)	A	A	A	A	A	G	C	A	T	C	A	G	A	A	A	G	A	A	C	C	T	C	C	T	T	C	T
5 (0.00)	A	G	A	A	G	G	C	A	T	C	A	G	A	A	A	G	A	A	C	C	T	C	C	A	T	T	T
5 (0.00)	A	C	A	A	A	G	C	A	T	C	A	G	A	A	A	G	A	A	C	C	T	C	C	A	T	T	C
5 (0.00)	T	A	A	A	A	G	C	A	T	C	A	G	A	A	A	G	A	A	C	C	T	C	C	A	C	T	T
5 (0.00)	A	A	A	A	A	C	A	A	T	C	A	G	A	A	A	G	A	A	C	C	T	C	C	A	T	T	T
5 (0.00)	C	A	A	A	A	G	C	A	T	C	A	G	A	A	A	G	A	A	C	C	T	C	C	G	T	T	T
5 (0.00)	T	A	A	A	A	G	C	A	T	C	A	G	A	A	A	G	A	A	C	C	T	C	C	A	T	T	A
5 (0.00)	C	A	A	A	A	G	C	A	T	C	A	G	A	A	A	G	A	A	C	C	T	C	C	A	T	T	G
5 (0.00)	C	A	A	A	G	G	C	A	T	C	A	G	A	A	A	G	A	A	C	C	T	C	C	A	T	T	T
5 (0.00)	A	A	A	A	A	G	C	A	T	C	A	G	A	A	A	G	A	A	C	C	T	C	C	C	T	T	C
5 (0.00)	A	A	A	A	A	G	C	A	T	C	A	G	A	A	A	G	A	A	C	C	T	G	C	A	T	T	T
5 (0.00)	A	A	A	A	A	G	C	A	T	C	A	G	A	A	A	G	A	A	C	C	T	C	C	T	C	T	T
5 (0.00)	A	A	A	A	A	G	C	A	T	C	A	G	A	A	A	G	A	A	C	C	T	C	C	G	T	A	T
4 (0.00)	T	A	A	A	A	G	C	A	T	C	A	G	A	A	A	G	A	A	C	C	T	C	C	G	T	T	T
4 (0.00)	A	A	A	A	A	G	C	A	T	C	A	G	A	A	A	G	A	A	C	C	T	C	C	G	T	G	T
4 (0.00)	A	C	G	A	A	G	C	A	T	C	A	G	A	A	A	G	A	A	C	C	T	C	C	A	T	T	T
4 (0.00)	A	G	A	A	A	G	C	A	T	C	A	G	A	A	A	G	A	A	C	C	T	C	C	G	T	T	T
4 (0.00)	A	A	A	G	A	G	C	A	T	C	A	G	A	A	A	G	A	A	C	C	T	C	C	A	T	T	C
4 (0.00)	A	A	G	A	G	G	C	A	T	C	A	G	A	A	A	G	A	A	C	C	T	C	C	A	T	T	T
4 (0.00)	G	A	A	A	A	G	C	A	T	C	A	G	A	A	A	G	A	A	C	C	T	C	C	A	A	T	T
4 (0.00)	T	A	A	A	A	G	C	A	T	C	A	G	A	A	A	G	A	A	C	C	T	C	C	A	T	G	T
4 (0.00)	G	A	A	A	A	G	T	A	T	C	A	G	A	A	A	G	A	A	C	C	T	C	C	A	T	T	T
4 (0.00)	A	A	A	A	A	G	C	A	T	C	A	G	A	A	A	G	A	A	C	C	T	C	C	T	T	T	C
4 (0.00)	C	A	C	A	A	G	C	A	T	C	A	G	A	A	A	G	A	A	C	C	T	C	C	A	T	T	T
4 (0.00)	A	A	A	A	A	G	C	A	T	C	A	G	A	A	A	G	A	A	C	C	T	C	C	C	C	T	T
4 (0.00)	A	A	A	A	A	G	C	A	T	C	A	G	A	A	A	G	A	A	C	C	T	C	C	G	T	T	G
4 (0.00)	A	A	A	A	A	G	C	A	T	C	A	G	A	A	A	G	A	A	C	C	T	C	C	G	T	T	A
4 (0.00)	A	A	A	A	A	G	C	A	T	C	A	G	A	A	A	G	A	A	C	C	T	C	C	A	T	A	A

Figure S2. Complete alignment of deep sequencing reads for A2 (cohort 1).

The reference sequence is shown at the top (GenBank AF316544). The number and percentage of identical reads are indicated.

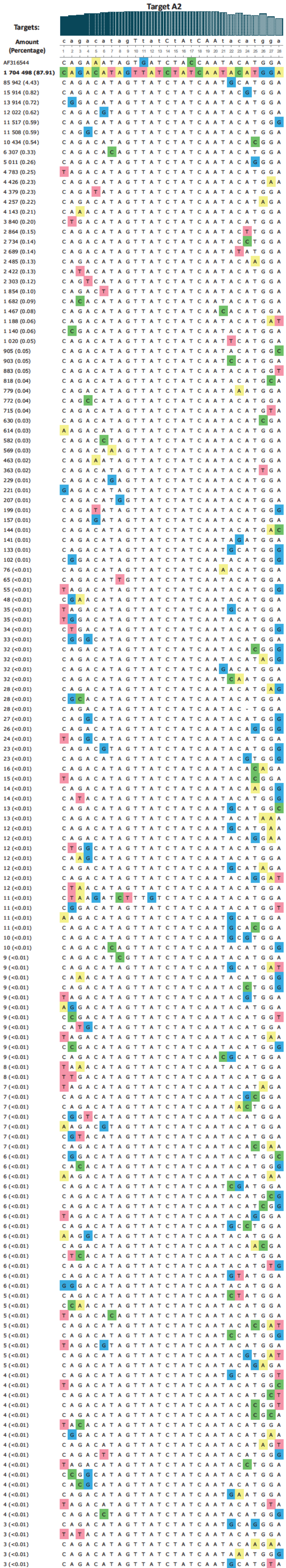
Target A2
Targets:
Amount
(Percentage)
AF316544

Figure S3. Complete alignment of deep sequencing reads for A3 (cohort 1).

The reference sequence is shown at the top (GenBank AF316544). The number and percentage of identical reads are indicated.

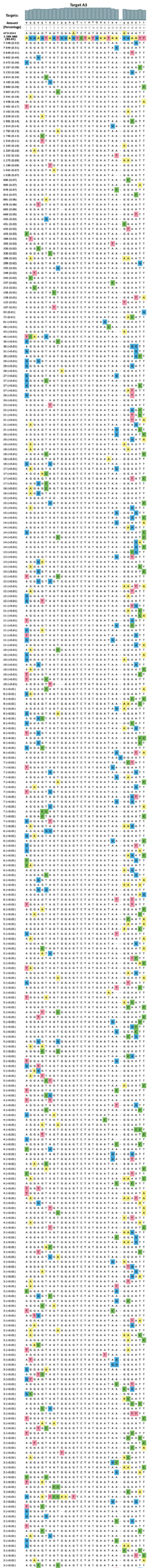
Target A3
Targets:
Amount
(Percentage)
AF316544
1 290 680 (92.61)

Figure S4. Complete alignment of deep sequencing reads for A4 (cohort 1).

The reference sequence is shown at the top (GenBank AF316544). The number and percentage of identical reads are indicated.

Target A4

Targets:

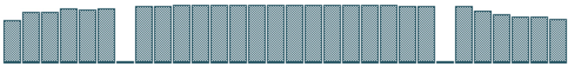

Amount (Percentage)	Sequence (positions 1–30)
	tgtgtg-gaCtATaGtaggTAta-gaatat
AF316544	AGTGTG-GACCATAGTGTATATA-GAATAT
**138 085 (92.44)**	TGTGTG-GACTATAGTAGGTATA-GAATAT
1 165 (0.78)	TGTGTG-GACTATAGTAGGTATA-GGATAT
1 078 (0.72)	TGCGTG-GACTATAGTAGGTATA-GAATAT
1 050 (0.70)	TGTGTG-GACTATAGTAGGTATA-GAGTAT
1 031 (0.69)	CGTGTG-GACTATAGTAGGTATA-GAATAT
1 001 (0.67)	TGTGTG-GACTATAGTAGGTATA-GAATGT
902 (0.60)	TGTGTG-GACTATAGTAGGTATA-GAACAT
839 (0.56)	TGTGTG-GACTATAGTAGGTATA-GAATAC
357 (0.24)	TGTGTG-GACTATAGTAGGTATA-GAATAA
338 (0.23)	TATGTG-GACTATAGTAGGTATA-GAATAT
331 (0.22)	TGTGCG-GACTATAGTAGGTATA-GAATAT
324 (0.22)	TGAGTG-GACTATAGTAGGTATA-GAATAT
304 (0.20)	TGTGTG-GACTATAGTAGGTATA-GAAAAT
190 (0.13)	AGTGTG-GACTATAGTAGGTATA-GAATAT
188 (0.13)	TGTGTG-GACTATAGTAGGTATA-GAAGAT
173 (0.12)	TGTGTG-GACTATAGTAGGTATA-GTATAT
166 (0.11)	TGTGTG-GACTATAGTAGGTATA-GAATCT
151 (0.10)	TGTGTG-GACTATAGTAGGTATA-GATTAT
134 (0.09)	TGTGTG-GACTATAGTAGGTATA-GAATTT
119 (0.08)	TGTGAG-GACTATAGTAGGTATA-GAATAT
115 (0.08)	GTTGTG-GACTATAGTAGGTATA-GAATAT
104 (0.07)	TGTGTG-GACTATAGTAGGTATA-GACTAT
102 (0.07)	TGTGT--GACTATAGTAGGTATA-GAATAT
92 (0.06)	TGGGTG-GACTATAGTAGGTATA-GAATAT
90 (0.06)	TGTGTG-GACTATAGTAGGTATA-GAATAG
84 (0.06)	TGTGTG-GACTATAGTAGGTATA-GCATAT
57 (0.04)	TGTATG-GACTATAGTAGGTATA-GAATAT
48 (0.03)	TGTGTG-AACTATAGTAGGTATA-GAATAT
48 (0.03)	TGTGTA-GACTATAGTAGGTATA-GAATAT
46 (0.03)	TGTGTG-GACTATAGTAGGTATA-AAATAT
46 (0.03)	TGTGTG-GACTATAGTAGGTATG-GAATAT
39 (0.03)	TGTGGG-GACTATAGTAGGTATA-GAATAT
36 (0.02)	GGTGTG-GACTATAGTAGGTATA-GAATAT
35 (0.02)	TCTGTG-GACTATAGTAGGTATA-GAATAT
32 (0.02)	TGTGTG-GGCTATAGTAGGTATA-GAATAT
29 (0.02)	TGTGTG-GACTATAGTAGGTATA-GAAATT
25 (0.02)	TGTGTG-GACTATAGTAGGTACA-GAATAT
18 (0.01)	TGTGTG-GACTATAGTAGGTATA-GAACGT
18 (0.01)	TTTGTG-GACTATAGTAGGTATA-GAATAT
16 (0.01)	TGTGTG-CACTATAGTAGGTATA-GAATAT
14 (0.01)	TGTGTG-GACTATAGTAGGTATA-CAATAT
12 (0.01)	TGTGTG-GACTATAGTAGGTATT-GAATAT
12 (0.01)	TGTGTG-GACTATAGTAGGTATA-GAATCA
12 (0.01)	TGTGTG-GACTATAGTAGGTATA-GATATA
11 (0.01)	TGTGTG-TACTATAGTAGGTATA-GAATAT
11 (0.01)	TGTGTT-GACTATAGTAGGTATA-GAATAT
11 (0.01)	TGTGTG-GACTATAGTAGGTATA-TAATAT
11 (0.01)	TGTGTG-GACTATAGTAGGTATA-GAGTAA
11 (0.01)	TGTGTG-GACTATAGTAGGTATC-GAATAT
10 (0.01)	TGTGTG-GACTATAGTAGGTATA-GAAACT
10 (0.01)	TGTCTG-GACTATAGTAGGTATA-GAATAT
9 (0.01)	TGTGTG-GACTATAGTAGGTATA-GAGTGT
9 (0.01)	CGTGTG-GACTATAGTAGGTATA-GAATAC
9 (0.01)	TGTGTC-GACTATAGTAGGTATA-GAATAT
8 (0.01)	CGTGTG-GACTATAGTAGGTATA-GAGTAT
7 (0.01)	TGTGTG-GACTATAGTAGGTATA-GAGTAC
7 (0.01)	TGTGTG-GACTATAGTAGGTATA-GAGAAT
6 (<0.01)	CGTGTG-GACTATAGTAGGTATA-GAATGT
6 (<0.01)	CGCGTG-GACTATAGTAGGTATA-GAATAT
5 (<0.01)	TGTGTG-GACTATAGTAGGTATA-GAACTA
5 (<0.01)	TGTGTG-GACTATAGTAGGTATA-GGATGT
5 (<0.01)	TGTGTG-GACTATAGTAGGTATA-GAATGC
5 (<0.01)	TGTGTG-GCCTATAGTAGGTATA-GAATAT
5 (<0.01)	TGTGTG-GACTATAGTAGGTATA-GAGCAT
5 (<0.01)	TGTGTGTGACTATAGTAGGTATA-GAATAT
5 (<0.01)	TGTGTG-GACTATAGTAGGTATA-GAACTT
5 (<0.01)	CGTGTG-GACTATAGTAGGTATA-GAACAT
5 (<0.01)	TGTTTG-GACTATAGTAGGTATA-GAATAT
4 (<0.01)	TGCGTG-GACTATAGTAGGTATA-GAATAC
4 (<0.01)	TGTGTG-GACTATAGTAGGTATA-GAACAC
3 (<0.01)	TGTGTG-GACTATAGTAGGTATA-GGACAT
3 (<0.01)	TGTGTG-GTCTATAGTAGGTATA-GAATAT
3 (<0.01)	CGTGTG-GACTATAGTAGGTATA-GAATAA
3 (<0.01)	CGTGTG-GACTATAGTAGGTATA-GGATAT
3 (<0.01)	AGTGTG-GACTATAGTAGGTATA-GAATAC
3 (<0.01)	TGTGG--GACTATAGTAGGTATA-GAATAT
3 (<0.01)	TGTGTG-GACTATAGTAGGTATA-GATCAT
2 (<0.01)	AGCGTG-GACTATAGTAGGTATA-GAATAT
2 (<0.01)	CGTGTG-GACTATAGTAGGTATA-GAAAAT
2 (<0.01)	TGTGTG-GACTATAGTAGGTATA-GGATAC
2 (<0.01)	CATGTG-GACTATAGTAGGTATA-GAATAT
2 (<0.01)	TGTGTG-GACTATAGTAGGTATA-GCATGT

Figure S5. Complete alignment of deep sequencing reads for A5 (cohort 1).

The reference sequence is shown at the top (GenBank AF316544). The number and percentage of identical reads are indicated.

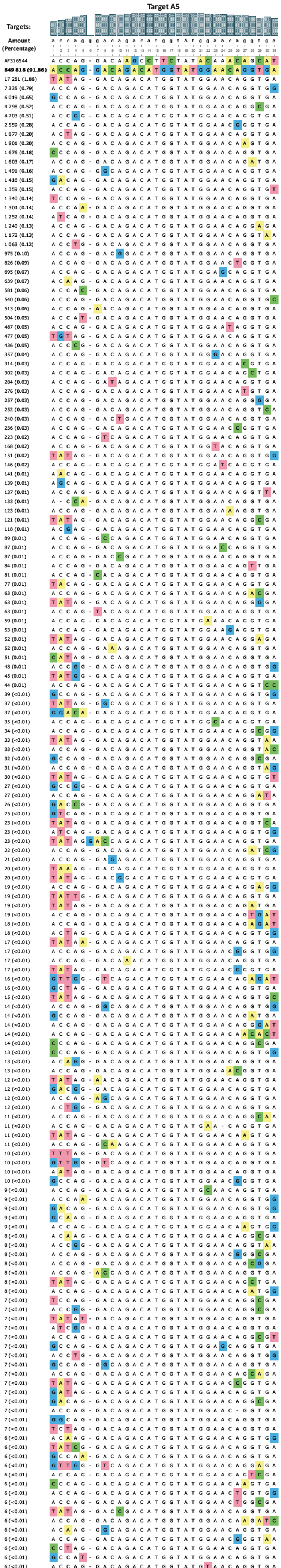

Target A5
Targets:
Amount
(Percentage)
a c c a g g g a c a g a c a t g g t A t g g a a c a g g t g a
1 2 3 4 5 6 7 8 9 10 11 12 13 14 15 16 17 18 19 20 21 22 23 24 25 26 27 28 29 30 31
AF316544 A C C A G - G A C A A G C C T T C T A T A C A A A C A G C A T
849 818 (91.86) A C C A G - G A C A G A C A T G G T A T G G A A C A G G T G A

Figure S6. Complete alignment of deep sequencing reads for A6 (cohort 1).

The reference sequence is shown at the top (GenBank AF316544). The number and percentage of identical reads are indicated.

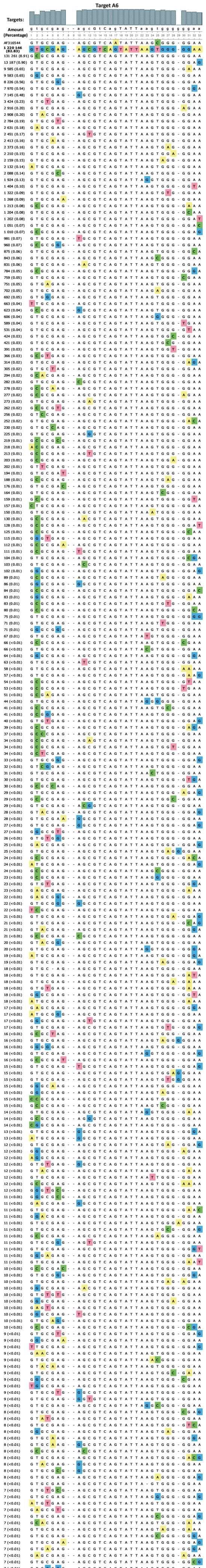
Target A6
Targets:
Amount
(Percentage)

Figure S7. Complete alignment of deep sequencing reads for A1 (cohort 2).

The reference sequence is shown at the top (GenBank AF316544). The number and percentage of identical reads are indicated.

Target A1

Targets:

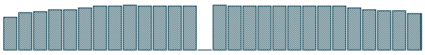

Amount (Percentage)	Sequence (positions 1–28)
Target	aaaaagcatcaga-aagaacctccattt
AF316544	AAAAAACATCAGA-AAGAACCTCCATTC
**7 976 486 (97.34)**	AAAAAGCATCAGA-AAGAACCTCCATTT
67 293 (0.82)	AGAAAGCATCAGA-AAGAACCTCCATTT
66 389 (0.81)	GAAAAGCATCAGA-AAGAACCTCCATTT
37 133 (0.45)	TAAAAGCATCAGA-AAGAACCTCCATTT
10 327 (0.13)	AAAAAGCATCAGA-AAGAACCTCCATTC
8 268 (0.10)	CAAAAGCATCAGA-AAGAACCTCCATTT
7 295 (0.09)	ATAAAGCATCAGA-AAGAACCTCCATTT
5 974 (0.07)	AAAAAGCATCAGA-AAGAACCTCCATCT
1 903 (0.02)	ACAAAGCATCAGA-AAGAACCTCCATTT
1 352 (0.02)	AAAGAGCATCAGA-AAGAACCTCCATTT
1 332 (0.02)	AAAAAACATCAGA-AAGAACCTCCATTT
1 233 (0.01)	AAGAAGCATCAGA-AAGAACCTCCATTT
1 197 (0.01)	AAAAAGCATCAGA-AAGAACCTCCATTA
1 159 (0.01)	AAAAAGCATCAGA-AAGAACCTCCACTT
866 (0.01)	AAAAAGCATCAGA-AAGAACCTCCGTTT
755 (0.01)	AAAAGGCATCAGA-AAGAACCTCCATTT
687 (0.01)	AAAAAGCATCAGA-AAGAACCTCCATAT
472 (0.01)	GGAAAGCATCAGA-AAGAACCTCCATTT
333 (<0.01)	AAATAGCATCAGA-AAGAACCTCCATTT
293 (<0.01)	AACAAGCATCAGA-AAGAACCTCCATTT
269 (<0.01)	AAAAAGTATCAGA-AAGAACCTCCATTT
245 (<0.01)	AAAAAGCATCAGA-AAGAACCTCCATTG
242 (<0.01)	AAACAGCATCAGA-AAGAACCTCCATTT
236 (<0.01)	AAAAAGAATCAGA-AAGAACCTCCATTT
218 (<0.01)	AAAAATCATCAGA-AAGAACCTCCATTT
207 (<0.01)	TGAAAGCATCAGA-AAGAACCTCCATTT
200 (<0.01)	AAAAAGCATCAGA-AAGAACCTCCATGT
170 (<0.01)	AATAAGCATCAGA-AAGAACCTCCATTT
143 (<0.01)	AAAAACCATCAGA-AAGAACCTCCATTT
141 (<0.01)	AAAAAGCATCAGA-AAGAACCTCTATTT
129 (<0.01)	AAAACGCATCAGA-AAGAACCTCCATTT
112 (<0.01)	AAAAAGCATCAGA-AAGAACCTCCTTTT
106 (<0.01)	AAAAAGCATCAGA-AAGAACCTCCAGTT
103 (<0.01)	AAAAAGCATCAGA-AAGAACCTCAATTT
98 (<0.01)	AAAAAGCATCAGA-AAGAACCTCCCTTT
84 (<0.01)	AAAAAGCATCAGA-AAGAACCTCCAATT
76 (<0.01)	AAAAAGCATCAGA-AAGAACCCCCATTT
70 (<0.01)	GAAAAGCATCAGA-AAGAACCTCCATTC
67 (<0.01)	GTAAAGCATCAGA-AAGAACCTCCATTT
64 (<0.01)	AAAATGCATCAGA-AAGAACCTCCATTT
52 (<0.01)	AAAAAGCATCAGA-AAGAACCACCATTT
51 (<0.01)	AAAAAGCATCAGA-AAGAACCTC-ATTT
46 (<0.01)	CGAAAGCATCAGA-AAGAACCTCCATTT
45 (<0.01)	AAAAAGCATCAGA-AAGAACCTCGATTT
42 (<0.01)	AAAAAGCATCAGA-AAGAACCTCCATCC
36 (<0.01)	TAAAAGCATCAGA-AAGAACCTCCATTC
35 (<0.01)	CAAAAGCATCAGA-AAGAACCTCCATTC
34 (<0.01)	AAAAAGCATCAGA-AAGAACCTACATTT
34 (<0.01)	CTAAAGCATCAGA-AAGAACCTCCATTT
23 (<0.01)	TTAAAGCATCAGA-AAGAACCTCCATTT
22 (<0.01)	AAAAAGGATCAGA-AAGAACCTCCATTT
22 (<0.01)	AAAAAGCATCAGA-AAGAACCGCCATTT
16 (<0.01)	AAAAAGCATCAGA-AAGAACATCCATTT
15 (<0.01)	AAAAAGCATCAGA-AAGAACCTCCACTC
14 (<0.01)	AAAAAGCATCAGA-AAGAACCTCCGTTC
14 (<0.01)	GAAAAGCATCAGA-AAGAACCTCCATCT
14 (<0.01)	GCAAAGCATCAGA-AAGAACCTCCATTT
14 (<0.01)	AAAAAGCATGAGA-AAGAACCTCCATTT
13 (<0.01)	AAAAAGCATCAGA-AAAAACCTCCATTT
12 (<0.01)	AGAAAGCATCAGA-AAGAACCTCCATTC
12 (<0.01)	AAAAAGCATCAGA-AAGAACCTTCATTT
11 (<0.01)	CAAAAGCATCAGA-AAGAACCTCCATCT
11 (<0.01)	GAGAAGCATCAGA-AAGAACCTCCATTT
10 (<0.01)	CAGAAGCATCAGA-AAGAACCTCCATTT
10 (<0.01)	AAAAAGCATCAGA-AAGAACCTCCATAA
9 (<0.01)	AAAAAGCATCAGA-GAGAACCTCCATTT
9 (<0.01)	AAAAAGCATAAGA-AAGAACCTCCATTT
9 (<0.01)	AAAAAGCATCAGA-AAGAACCTGCATTT
9 (<0.01)	AGGAAGCATCAGA-AAGAACCTCCATTT
8 (<0.01)	GAAAAGCATCAGA-AAGAACCTCCGTTT
8 (<0.01)	AAAAAGCATCAGA-AAGAACCTCCATAC
8 (<0.01)	AAAAAGCATCAGA-AAGAACCTCCGCTT
7 (<0.01)	GAAAAGCATCAGA-AAGAACCTCCACTT
7 (<0.01)	AAAAAGCATCAGA-AAGAACCTCCACCT
7 (<0.01)	AAAAAGCATCAGA-AAGAACGTCCATTT
6 (<0.01)	TCAAAGCATCAGA-AAGAACCTCCATTT
6 (<0.01)	TAAAAGCATCAGA-AAGAACCTCCATTA
6 (<0.01)	AAGAAGCATCAGA-AAGAACCTCCATTC
5 (<0.01)	AAAAAGCATCAGG-AAGAACCTCCATTT
5 (<0.01)	GAAAAGCATCAGA-AAGAACCTCCATTA
5 (<0.01)	AAAAAGCATCAGA-AAGAACCTCCGTCT
5 (<0.01)	AAAAAGCATTAGA-AAGAACCTCCATTT
5 (<0.01)	AAAAAGCATCAGA-AAGAACCTCCATAG
5 (<0.01)	AGAAAGCATCAGA-AAGAACCTCCATCT
5 (<0.01)	TAAAAGCATCAGA-AAGAACCTCCGTTT
4 (<0.01)	AGAAAGCATCAGA-AAGAACCTCCATTA
4 (<0.01)	GAAGAGCATCAGA-AAGAACCTCCATTT
4 (<0.01)	CAAAAGCATCAGA-AAGAACCTCCACTT
4 (<0.01)	CAAAAGCATCAGA-AAGAACCTCCATTA
4 (<0.01)	AAAAAGCATCAGA-AAGGACCTCCATTT
4 (<0.01)	AGAAGGCATCAGA-AAGAACCTCCATTT
4 (<0.01)	AAAAAGCATCAGA-AAGAACTTCCATTT
4 (<0.01)	AGAAAGCATCAGA-AAGAACCTCCGTTT
4 (<0.01)	AAGAAGCATCAGA-AAGAACCTCCACTT
4 (<0.01)	AAAAAGCGTCAGA-AAGAACCTCCATTT
3 (<0.01)	AAAAAGCATCAGC-AAGAACCTCCATTT
3 (<0.01)	GATAAGCATCAGA-AAGAACCTCCATTT
3 (<0.01)	AAAAAGCATCATA-AAGAACCTCCATTT
3 (<0.01)	AAAAAGCATCAGA-AAGAAACTCCATTT
3 (<0.01)	AAAAAGCATCAGA-TAGAACCTCCATTT
3 (<0.01)	AAAAGGCATCAGA-AAGAACCTCCATTC
3 (<0.01)	GAAAAACATCAGA-AAGAACCTCCATTT
3 (<0.01)	AAAAAGCATCGGA-AAGAACCTCCATTT
3 (<0.01)	AAAAAGCATCAGA-AACAACCTCCATTT
[illegible]	[illegible]

Figure S8. Complete alignment of deep sequencing reads for A2 (cohort 2).

The reference sequence is shown at the top (GenBank AF316544). The number and percentage of identical reads are indicated.

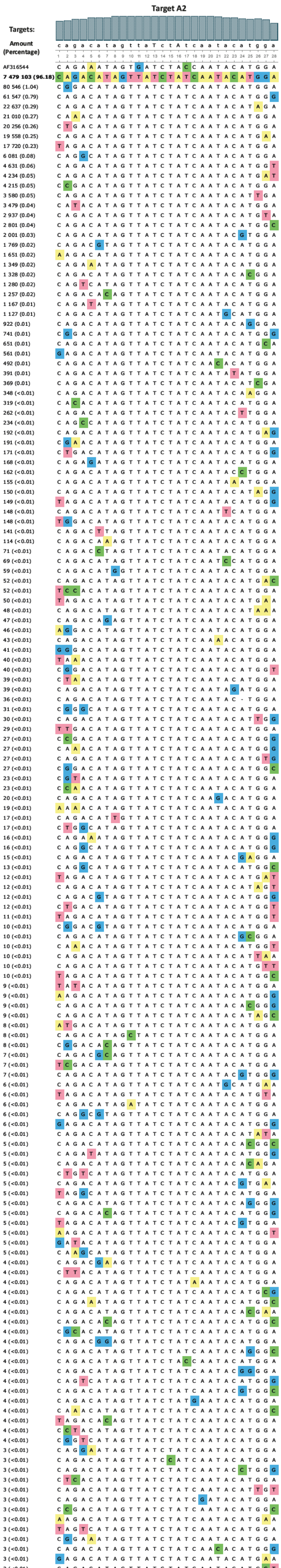

Target A2
Targets:
Amount (Percentage)

Figure S9. Complete alignment of deep sequencing reads for A3 (cohort 2).

The reference sequence is shown at the top (GenBank AF316544). The number and percentage of identical reads are indicated.

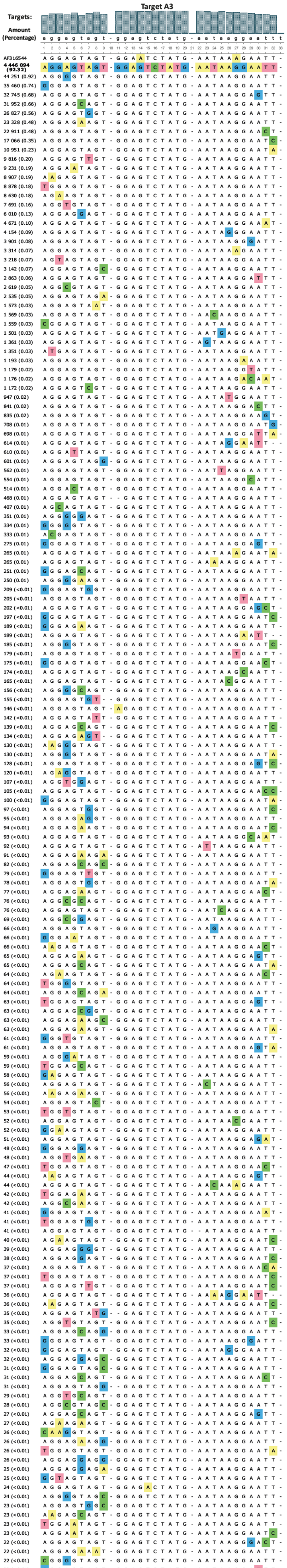
Target A3
Targets:
Amount
(Percentage)
AF316544
4 446 094
(92.32)

Figure S10. Complete alignment of deep sequencing reads for A4 (cohort 2).

The reference sequence is shown at the top (GenBank AF316544). The number and percentage of identical reads are indicated.

Target A4

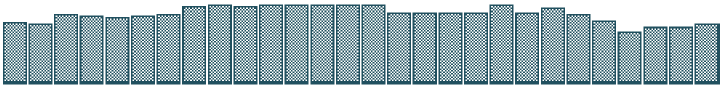

Targets:	t g t g t g g a C t A T A G t a g g t a t a g a a t a t
Amount (Percentage)	1 2 3 4 5 6 7 8 9 10 11 12 13 14 15 16 17 18 19 20 21 22 23 24 25 26 27 28
AF316544	A G T G T G G A C C A T A G T G T A T A T A G A A T A T
**73 906 (95.28)**	T G T G T G G A C T A T A G T A G G T A T A G A A T A T
656 (0.85)	C G T G T G G A C T A T A G T A G G T A T A G A A T A T
596 (0.77)	T G T G T G G A C T A T A G T A G G T A T A G A G T A T
497 (0.64)	T A T G T G G A C T A T A G T A G G T A T A G A A T A T
418 (0.54)	T G C G T G G A C T A T A G T A G G T A T A G A A T A T
369 (0.48)	T G T G T G G A C T A T A G T A G G T A T A G A A T A C
137 (0.18)	A G T G T G G A C T A T A G T A G G T A T A G A A T A T
121 (0.16)	T G A G T G G A C T A T A G T A G G T A T A G A A T A T
108 (0.14)	T G T G T G G A C T A T A G T A G G T A T A G A A C A T
70 (0.09)	T G T G T G G A C T A T A G T A G G T A T A G A A T G T
49 (0.06)	T G T G T G G A C T A T A G T A G G T A T A G A A T A A
43 (0.06)	- G T G T G G A C T A T A G T A G G T A T A G A A T A T
29 (0.04)	T G T G T G G A C T A T A G T A G G T A T A G - - - - -
27 (0.04)	T A T G T G G A C T A T A G T A G G T A T A G A G T A T
27 (0.04)	- - T G T G G A C T A T A G T A G G T A T A G A A T A T
26 (0.03)	G G T G T G G A C T A T A G T A G G T A T A G A A T A T
26 (0.03)	T G G G T G G A C T A T A G T A G G T A T A G A A T A T
26 (0.03)	T G T G T G G A C T A T A G T A G G T A T G G A A T A T
25 (0.03)	T A T A C C T A C T A T A G T C C A C A C A A C T A C T
25 (0.03)	T G T G A G G A C T A T A G T A G G T A T A G A A T A T
24 (0.03)	T G T G C G G A C T A T A G T A G G T A T A G A A T A T
23 (0.03)	T G T A T G G A C T A T A G T A G G T A T A G A A T A T
21 (0.03)	T G T G T G G A C T A T A G T A G G T A T A G G A T A T
19 (0.02)	T G T G T G G A C T A T A G T A G G T A T A G A A T T T
15 (0.02)	- - - G T G G A C T A T A G T A G G T A T A G A A T A T
15 (0.02)	T G T G T G G G C T A T A G T A G G T A T A G A A T A T
15 (0.02)	T A T A C C T A C T A T A G T C C A C A C A A C T A T T
14 (0.02)	T G T G T A G A C T A T A G T A G G T A T A G A A T A T
12 (0.01)	T G T G T G G A C T A T A G T A G G T A T A G A A T - -
11 (0.01)	T G T G T G G A C T A T A G T A G G T A T A G A - - - -
11 (0.01)	G T T G T G G A C T A T A G T A G G T A T A G A A T A T
11 (0.01)	T T T G T G G A C T A T A G T A G G T A T A G A A T A T
10 (0.01)	T G T G T G G A C T A T A G T A G G T A T A G A A A A T
9 (0.01)	T G T G T G G A C T A T A G T A G G T A T A G A A T A -
9 (0.01)	T G T G T G G A C T A T A G T A G G T A T A G A A T A G
8 (0.01)	C G T G T G G A C T A T A G T A G G T A T A G A A T G T
8 (0.01)	T G T G T G A A C T A T A G T A G G T A T A G A A T A T
7 (0.01)	T G T G T G G A C T A T A G T A G G T A T A G A A A T A
6 (0.01)	T G T G T G G A C T A T A G T A G G T A T A G A G - - -
6 (0.01)	T G T G T G G A C T A T A G T A G G T A T A G A G C A T
6 (0.01)	T G T G T G G A C T A T A G T A G G T A T A A A A T A T
6 (0.01)	T G T G T G G A C T A T A G T A G G T A T A G C A T A T
5 (0.01)	T G T G T G G A C T A T A G T A G G T A T A G T A T A T
5 (0.01)	T G T G T G G A C T A T A G T A G G T A T A G A G A A T
5 (0.01)	T G T G T G G A C T A T A G T A G G T A T A - - - - - -
5 (0.01)	T G T G T G G A C T A T A G T A G G T A T A G A A T C T
5 (0.01)	T G T G T G G A C T A T A G T A G G T A T A G A A - - -
4 (0.01)	T G T G G G G A C T A T A G T A G G T A T A G A A T A T
4 (0.01)	C G T G A G G A C T A T A G T A G G T A T A G A A T A T
3 (0.00)	T G T G T G G A C T A T A G T A G G T A T A G C G T A T
3 (0.00)	C G C G T G G A C T A T A G T A G G T A T A G A A T A T
3 (0.00)	C G T G T G G A C T A T A G T A G G T A T A G A A T A C
3 (0.00)	T G T G T G G A C T A T A G T A G G T A T A G A C T A T
3 (0.00)	T A T G T G G A C T A T A G T A G G T A T A G A G - - -
3 (0.00)	T G T G T G C A C T A T A G T A G G T A T A G A A T A T
2 (0.00)	T G T G T G G A C T A T A G T A G G T A T A G A T T A T
2 (0.00)	T G T G T G G A C T A T A G T A G G T A T A G A G T - -
2 (0.00)	T G T G T G G T C T A T A G T A G G T A T A G A A T A T

Figure S11. Complete alignment of deep sequencing reads for A5 (cohort 2).

The reference sequence is shown at the top (GenBank AF316544). The number and percentage of identical reads are indicated.

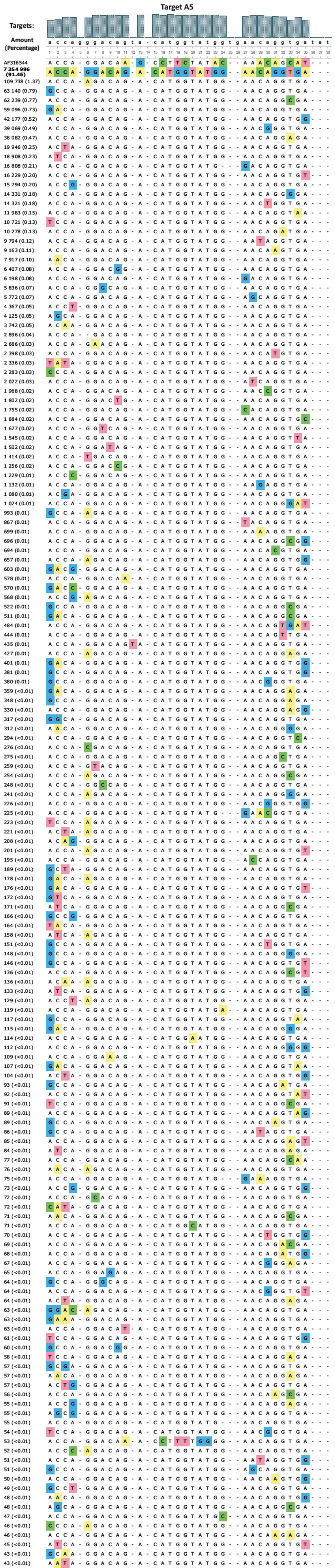
Target A5
Targets:
Amount
(Percentage)
AF316544

Figure S12. Complete alignment of deep sequencing reads for A6 (cohort 2).

The reference sequence is shown at the top (GenBank AF316544). The number and percentage of identical reads are indicated.

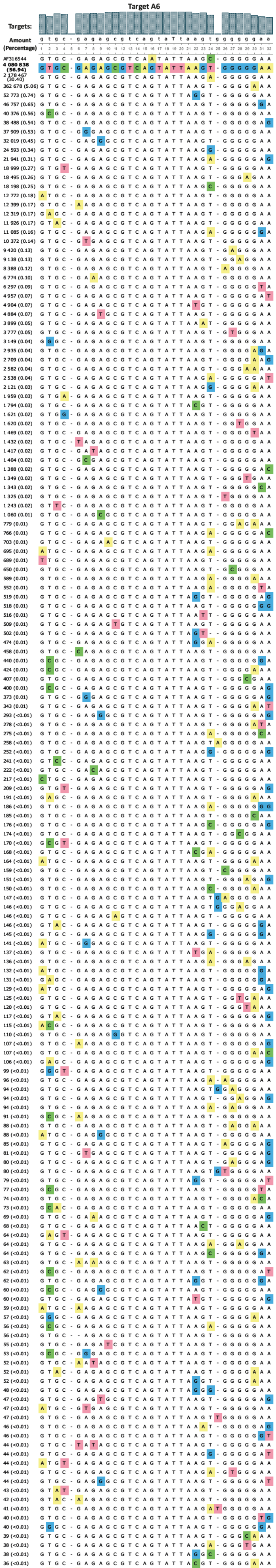
Target A6
Targets:
Amount
(Percentage)
AF316544

Figure S13. Conserved Domains Database (CDD) and Resources – NCBI – for the targets A1–A6.

The regions corresponding to the targets are highlighted in yellow.

[Conserved Domains Database (CDD) and Resources - NCBI]

A1, A2

RT_Rtv: Reverse transcriptases (RTs) from retroviruses (Rtvs). RTs catalyze the conversion of single-stranded RNA into double-stranded viral DNA for integration into host chromosomes.

Pssm-ID: 238823 [Multi-domain] **Cd Length:** 213 **Bit Score:** 350.43 **E-value:** 3.09e-113

```
                         10        20        30        40        50        60        70        80
                 ....*....|....*....|....*....|....*....|....*....|....*....|....*....|....*....|
gi 14530264  173 GPKVKQWPLTEEKIKALTEICKEMEKEGKISKIGpeNPYNTPIFAIKKKDStKWRKLVDFRELNKRTQDFWEVQLGIPHP 252
Cdd:cd01645    1 PVWIKQWPLTEEKLEALTELVTEQLKEGHIEPST--SPWNTPVFVIKKKSG-KWRLLHDLRAVNAQTQDMGALQPGLPHP 77
                         90       100       110       120       130       140       150       160
                 ....*....|....*....|....*....|....*....|....*....|....*....|....*....|....*....|
gi 14530264  253 AGLKKKKSVTVLDVGDAYFSVPLDEGFRKYTAFTIPSINNETPGVRYQYNVLPQGWKGSPAIFQSSMTKILEPFRADNPE 332
Cdd:cd01645   78 AALPKGWPLIVLDLKDCFFSIPLHPDDRERFAFTVPSINNKGPAKRYQWKVLPQGMKNSPTICQSFVAQALEPFRKQYPD 157
                        170       180       190       200       210
                 ....*....|....*....|....*....|....*....|....*....|....*..
gi 14530264  333 IVIYQYMDDLYVGSDLEiGQHRAKIEELREHLLRWGFTTPDKKHQKEPPFLWMGYEL 389
Cdd:cd01645  158 IVIYHYMDDILIASDLE-GQLREIYEELRQTLLRWGLTIPPEKVQKEPPFQYLGYEL 213
                    A2                                      A1
```

A3

Integrase core domain

Integrase mediates integration of a DNA copy of the viral genome into the host chromosome. Integrase is composed of three domains. The amino-terminal domain is a zinc binding domain pfam02022. This domain is the central catalytic domain. The carboxyl terminal domain that is a non-specific DNA binding domain pfam00552. The catalytic domain acts as an endonuclease when two nucleotides are removed from the 3' ends of the blunt-ended viral DNA made by reverse transcription. This domain also catalyzes the DNA strand transfer reaction of the 3' ends of the viral DNA to the 5' ends of the integration site.

```
                          10        20        30        40        50        60        70        80
                  ....*....|....*....|....*....|....*....|....*....|....*....|....*....|....*....|
gi 14530264   773 PGVWQLDCTHLEGKI------ILVAVHVASGYIEAEVIPAETGQETAYFILKLA----GGWPVKVIHTDNGSNFTSAAVK 842
Cdd:pfam00665   2 NELWQTDFTYVRVPGgggklyLAVAVDDFSREIVAWALSSEMDAELVIDALKRAiefrGPPGPKIIHSDNGSEYTSKAFQ 81
                          90       100       110
                  ....*....|....*....|....*....|..
gi 14530264   843 AACWWANVTQEFGIPYNPQSQGVVESMNKELK 874
Cdd:pfam00665  82 EFLAHYGITHSFSRPGNPQDNGKVERFNGTLK 113
```

A4

Vpu protein; The Vpu protein contains an N-terminal transmembrane spanning region and a C-terminal cytoplasmic region. The HIV-1 Vpu protein stimulates virus production by enhancing the release of viral particles from infected cells. The VPU protein binds specifically to CD4.

```
                         10        20        30        40        50        60        70
                 ....*....|....*....|....*....|....*....|....*....|....*....|....*....|....*
gi 14530268    1 MQALEISAIVGLIVAFIAATVVWTIVYIEYRKIRKQKRIERLLDRIGERAEDSGNESEGDAEE-LAKLVEMGGFD 74
Cdd:pfam00558  1 MLLLEIIGLIALIVALIINIVVWTIVYREYRKIKKQREILRLIKRIRERAEDSGNESNGDEEEeLADLVHSHGFD 75
```

A5

Envelope glycoprotein GP120; The entry of HIV requires interaction of viral GP120 with CD4 and a chemokine receptor on the cell surface.

Pssm-ID: 278917 **Cd Length:** 525 **Bit Score:** 36.68 **E-value:** 2.62e-05

```
                          10        20        30
                  ....*....|....*....|....*....|..
Query_22997     7 VEITCIRPNNNTRKSIRF--GPGQAFYTNSII 36
Cdd:pfam00516 295 LEINCKRPGNKTRKPIRImrGPGRAFVFHGKI 326
```

A6

gag gene protein p17 (matrix protein); The matrix protein forms an icosahedral shell associated with the inner membrane of the mature immunodeficiency virus.

Pssm-ID: 249943 **Cd Length:** 140 **Bit Score:** 195.93 **E-value:** 5.21e-60

```
                          10        20        30        40        50        60        70        80
                  ....*....|....*....|....*....|....*....|....*....|....*....|....*....|....*....|
gi_14530263     1 ARASILSGGKLEAWEKIRLRPGGKKKYRLKHLVWASRELEKFSINPGLLETAAGCRQILGQLQPALQTGTEELRSLYNTV 80
Cdd:pfam00540   2 ARASVLSGGELDKWEKIRLRPGGKKKYKLKHLVWASRELERFAVNPGLLETSEGCRKILGQLQPSLQTGSEGLRSLYNTV 81
                          90       100       110       120       130       140
                  ....*....|....*....|....*....|....*....|....*....|....*....|.
gi_14530263    81 AVLYCVHQKIEVKDTKEALDKIEEEQNTCKQRTQhaaADTGSSRSQDYRGSSSQNYPIVQN 141
Cdd:pfam00540  82 AVLYCVHQRIDVKDTKEALEKIEEEQNKSKKKKT---AVPPGAQQAANTGGTGNSSGVSQN 139
```
