# Supplementary material for: Six Highly Conserved Targets of RNAi Revealed in HIV-1-Infected Patients from Russia Are Also Present in Many HIV-1 Strains Worldwide
Source: Mol Ther Nucleic Acids. 2017 Jul 13;8:330–44. doi: 10.1016/j.omtn.2017.07.010 (PMC5537207; doi:10.1016/j.omtn.2017.07.010)
Supplement: Document S2. Article plus Supplemental Information [file mmc2.pdf]

Molecular Therapy
Nucleic Acids
Original Article

Six Highly Conserved Targets of RNAi Revealed in HIV-1-Infected Patients from Russia Are Also Present in Many HIV-1 Strains Worldwide

Olga V. Kretova,[1] Daria M. Fedoseeva,[1] Maria A. Gorbacheva,[1] Natalya M. Gashnikova,[2] Maria P. Gashnikova,[2] Nataliya V. Melnikova,[3] Vladimir R. Chechetkin,[1] Yuri V. Kravatsky,[1] and Nickolai A. Tchurikov[1]

[1]Department of Epigenetic Mechanisms of Gene Expression Regulation, Engelhardt Institute of Molecular Biology, 119334 Moscow, Russia; [2]Department of Retroviruses, State Research Center of Virology and Biotechnology Vector, 630559 Koltsovo, Russia; [3]Laboratory of Post-Genomic Research, Engelhardt Institute of Molecular Biology, 119334 Moscow, Russia

RNAi has been suggested for use in gene therapy of HIV/AIDS, but the main problem is that HIV-1 is highly variable and could escape attack from the small interfering RNAs (siRNAs) due to even single nucleotide substitutions in the potential targets. To exhaustively check the variability in selected RNA targets of HIV-1, we used ultra-deep sequencing of six regions of HIV-1 from the plasma of two independent cohorts of patients from Russia. Six RNAi targets were found that are invariable in 82%–97% of viruses in both cohorts and are located inside the domains specifying reverse transcriptase (RT), integrase, vpu, gp120, and p17. The analysis of mutation frequencies and their characteristics inside the targets suggests a likely role for APOBEC3G (apolipoprotein B mRNA editing enzyme, catalytic polypeptide-like 3G, A3G) in G-to-A mutations and a predominant effect of RT biases in the detected variability of the virus. The lowest frequency of mutations was detected in the central part of all six targets. We also discovered that the identical RNAi targets are present in many HIV-1 strains from many countries and from all continents. The data are important for both the understanding of the patterns of HIV-1 mutability and properties of RT and for the development of gene therapy approaches using RNAi for the treatment of HIV/AIDS.

INTRODUCTION

The current highly active antiretroviral therapy has greatly improved the morbidity and mortality of AIDS. However, many of the antiretroviral drugs exert high toxicity upon long-term usage, and all currently used anti-HIV agents generate the appearance of drug-resistant mutants. Therefore, there is a great need for the development of new approaches to HIV/AIDS therapy.

RNAi is a powerful tool to inhibit HIV-1 production in human cells. RNAi was suggested for silencing HIV-1 genes shortly after the discovery of RNAi.[1–10] Virus-specific RNAs transcribed from proviral DNA can be attacked in $CD4^+$ T cells and macrophages, which are the natural targets of HIV-1, and, as a result, the replication of the virus can be inhibited. The main problem in the development of an approach using RNAi for the treatment of HIV/AIDS is the high variability of the virus. This natural feature of the virus, which is important for its fitness, is mainly due to (1) the viral error-prone reverse transcriptase (RT), (2) recombination during DNA synthesis in the co-packaged genomes leading to an increase in the genetic diversity, and (3) the extremely high level of HIV-1 amplification, which leads to a large population of variants. Additionally, the host protein APOBEC3G (apolipoprotein B mRNA editing enzyme, catalytic polypeptide-like 3G, A3G) produces cytidine deamination (cytosine to uracil) during reverse transcription of the single-stranded newly synthesized minus-strand cDNA, leading to extensive G-to-A mutations in the viral plus strand inside the double-stranded DNA genome; this phenomenon is called hypermutation.[11–18] Together, these mechanisms lead to high variability in the HIV-1 genome and hamper the development of an RNAi approach for the treatment of HIV/AIDS. In fact, even a single point mutation in a target sequence can allow the virus to escape RNAi.[19,20]

Nevertheless, attempts to find targets that are compatible with a majority of the current HIV-1 variants have proceeded using two main strategies: (1) multiplexing, which is the use of several siRNAs against different targets in HIV-1 transcripts,[21,22] or (2) targeting of RNAs corresponding to highly conserved regions of the viral genome.[23–25] For example, using dual-siRNA hairpin constructs that simultaneously target two viral transcripts helps to avoid the formation of RNAi-escape mutants.[21] The accessible conserved isles in the coding regions of the viral mRNAs are good targets for RNAi because of a constraint in the number of variations tolerated in these critical coding regions. Currently, studies to improve RNA stability and delivery to the target cells are underway, and preclinical studies examining the efficiency of RNAi for viral suppression and gene therapy clinical

Received 3 March 2017; accepted 10 July 2017;
http://dx.doi.org/10.1016/j.omtn.2017.07.010.

Correspondence: Nickolai A. Tchurikov, Department of Epigenetic Mechanisms of Gene Expression Regulation, Engelhardt Institute of Molecular Biology, 119334 Moscow, Russia.
E-mail: tchurikov@eimb.ru

 © 2017 The Authors.
This is an open access article under the CC BY license (http://creativecommons.org/licenses/by/4.0/).

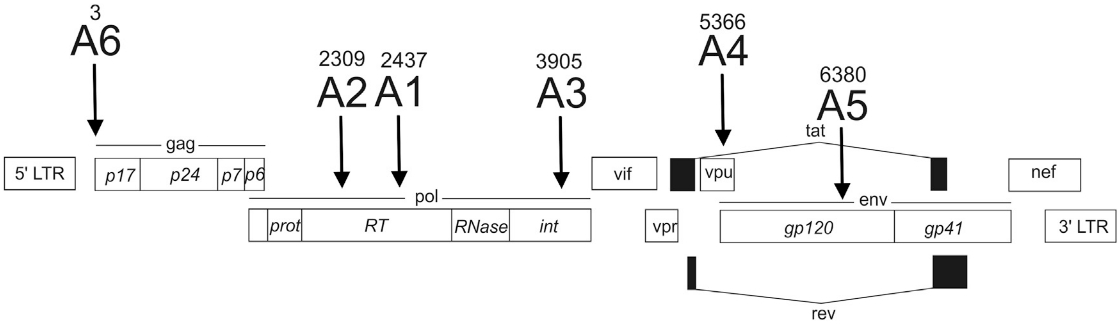

Figure 1. Schematic Presentation of RNAi Targets A1–A6 within the HIV-1 Gene Map

The positions of 19-bp targets are indicated. The values shown above the targets indicate the 5′ numbering of a target in the reference sequence (GenBank: AF316544).

trials for HIV-1-infected patients are ongoing (see Bobbin et al.[26] and Klemm et al.[27] for a review); however, RNAi-based therapy has not yet reached the clinic.

Here, we describe the results of ultra-deep sequencing of six conserved HIV-1 regions from two independent cohorts of patients in Russia. Our data indicate that up to 92%–97% of viruses possess six identical RNAi targets. The corresponding Dicer substrates efficiently attacked the targets in a non-viral system using the *luc* reporter gene. The data suggest that the detected targets could be used for the development of a gene therapy approach to treat HIV/AIDS.

RESULTS

Alignment of Ultra-Deep Sequencing Reads Reveals Conserved Sequences in the RNAi Targets

Selected siRNA targets in different conserved functionally important coding regions in the HIV-1 subtype A genome are shown in Figure 1. They are indicated as A1–A6 (A denotes the subtype of the virus). A1 and A2 are located inside the RT domain and A3 is inside the integrase domain (int), whereas A4, A5, and A6 are inside the domains specifying vpu, gp120, and p17, respectively.

Target A1 was selected using the Tuschl algorithm, whereas the remaining (A2, A3, A4, A5, and A6) targets were selected using the Dharmacon (http://dharmacon.gelifesciences.com/design-center/). All six RNAi targets are located in the fully spliced HIV-1 mRNA species and correspond to the protein-coding regions (Figure S13).[28] The reads of the ultra-deep sequencing were aligned using the procedure described in the Materials and Methods section, which allows the alignment and analysis up to about eight million reads. The data on the deep sequencing of the viruses from both cohorts were deposited in NCBI (Bioproject: PRJNA344431).

In our preliminary studies, we analyzed the same genomic regions using cloning and capillary sequencing. In total, 42 clones that included about 300-bp fragments of DNA and corresponded to the mixture of the viruses of cohort 1 were analyzed (GenBank: KC681847–KC681888). We deduced the consensus sequences of the targets based on the analysis of a limited number of clones.[29–31] Surprisingly, very similar target sequences were found in the mixtures of viruses from both cohorts, now based on analysis of thousands or millions of reads (Figures 2 and S1–S12; Table 1). It is of note that practically the same characteristic changes with respect to the reference sequence (isolate from the Republic of the Congo, GenBank: AF316544, 2001) were detected in all targets from both cohorts.

Table 1 shows the 27- to 30-bp regions that are found in up to 97% of viruses. For target A6 in cohort 2, we observed that only 56% of viruses possessed the identical target, whereas about 30% contained one nucleotide substitution (T to A) outside of the core sequence if compared with the result for cohort 1. The values for the 19-bp core sequences corresponding to the overlapping plus and minus strands in double-stranded siRNAs (ds-siRNAs) are even higher for all targets (up to 99%; Table S1). A core sequence does not include the dinucleotide 3′ overhangs of ds-siRNA and is critical for the efficiency of siRNA in the initiation of RNAi.[32] These A1–A6 regions correspond to the conserved coding regions in the HIV-1 genome. Figure S13 shows the amino acid sequences of the conserved domains from the Conserved Domains Database (CDD) and Resources (NCBI) (https://www.ncbi.nlm.nih.gov/Structure/cdd/cdd.shtml) and indicates the stretches corresponding to the conserved RNAi targets.

Lower Frequencies of Mutations Were Observed in the Middle Part of the RNAi Targets

Next, we used the deep sequencing data for the analysis of the mutation profiles and mutation characteristics along the targets. Figures 3 and 4 show the profiles of all 12 possible nucleotide substitutions (four transitions and eight transversions) along the 27- to 30-bp sequences in the plus strand of the HIV-1 DNA. In this study, the frequencies of nucleotide substitutions were determined against the sets of aligned sequences (Equations 1 and 2 in Materials and Methods), which is more convenient and unambiguous in the case of deep sequencing data. All 12 profiles (six regions from two cohorts) demonstrate mutation frequencies in a wide range from 10^{-7} to 10^{-1}.

The interesting common feature of all profiles is that they exhibit a U-shape and demonstrate the lowest mutation frequencies in the

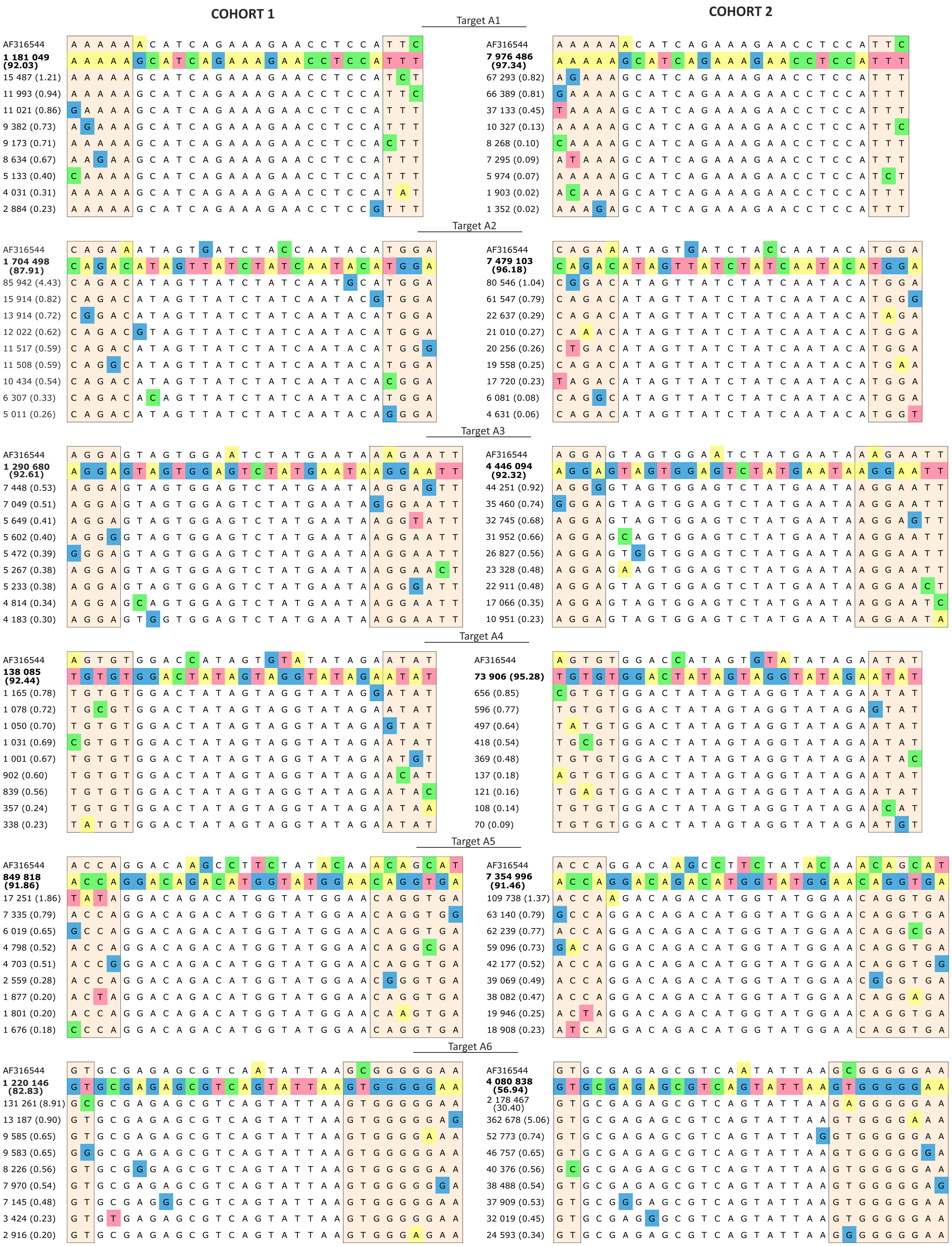

(legend on next page)

Table 1. Sequences of the 27- to 30-nt Regions Possessing RNAi Targets that Are Shared by the Majority of Viruses in Both Cohorts

Target	Domain	Sequence, 5′-3′	% of Reads	Source	Number of Reads
A1	RT	AAAAAACATCAGAAAGAACCTC CATTC	–	AF316544	–
		AAAAAGCATCAGAAAGAACCTC CATTT	–	C1, clones	–
		AAAAAGCATCAGAAAGAACCTC CATTT	92.3 (99,174)	C1, deep seq.	1,181,049
		AAAAAGCATCAGAAAGAACCTC CATTT	97.34 (97.71)	C2, deep seq.	7,976,486
A2	RT	CAGAAATAGTGATCTACCAATA CATGGA	–	AF316544	–
		CAGACATAGTTATCTATCAATA CATGGA	–	C1, clones	–
		CAGACATAGTTATCTATCAATA CATGGA	87.91 (92.39)	C1, deep seq.	1,704,498
		CAGACATAGTTATCTATCAATA CATGGA	96.18 (99.58)	C2, deep seq.	7,479,103
A3	Int	AGGAGTAGTGGAATCTATGAAT AAAGAATT	–	AF316544	–
		AGGAGTAGTGGAGTCTATGAAT AAGGAATT	–	C1, clones	–
		AGGAGTAGTGGAGTCTATGAAT AAGGAATT	92.61(96.84)	C1, deep seq.	1,290,680
		AGGAGTAGTGGAGTCTATGAAT AAGGAATT	92.32(96.68)	C2, deep seq.	4,446,094
A4	vpu	AGTGTGGACCATAGTGTATATA GAATAT	–	AF316544	–
		TGTGTGGACTATAGTAGGTATA GAATGT	–	C1, clones	–
		TGTGTGGACTATAGTAGGTATA GAATAT	92.44 (97.36)	C1, deep seq.	138,085
		TGTGTGGACTATAGTAGGTATA GAATAT	95.28 (99.7)	C2, deep seq.	73,906
A5	gp120	ACCAGGACAAGCCTTCTATACA AACAGCAT	–	AF316544	–
		ACCAGGACAGACATGGTATGGA ACAGGTGA	–	C1, clones	–
		ACCAGGACAGACATGGTATGGA ACAGGTGA	91.86 (97.14)	C1, deep seq.	849,818
		ACCAGGACAGACATGGTATGGA ACAGGTGA	91.46 (96.58)	C2, deep seq.	7,354,996
A6	p17	GTGCGAGAGCGTCAATATTAAG CGGGGGAA	–	AF316544	–
		GTGCGAGAGCGTCAGTATTAAG TGGGGGAA	–	C1, clones	–
		GTGCGAGAGCGTCAGTATTAAG TGGGGGAA	82.83 (94,12)	C1, deep seq.	1,220,146
		GTGCGAGAGCGTCAGTATTAAG TGGGGGAA	56.94 (96.94)	C2, deep seq.	4,080,838
		GTGCGAGAGCGTCAGTATTAAG AGGGGGAA	30.397	C2, deep seq.	2,178,467
		GTGCGAGAGCGTCAGTATTAAG TGGGGAAA	5.061	C2, deep seq.	362,678

Percentage of viruses possessing particular 27- to 30-nt regions in the deep sequencing data is shown after the corresponding sequence. The 19-nt core sequences corresponding to overlapping regions in double-stranded siRNAs are underlined. Percentage of viruses containing identical core sequence is shown in parentheses. The changes in nucleotide sequences from the reference sequence (isolate 97CDKP58e, 2001, from the Republic of the Congo, GenBank: AF316544) are highlighted in yellow. The changes between sequences in the same cohort are highlighted in green. The number of high-quality reads possessing the indicated target is shown in the rightmost column. C1, cohort 1; C2, cohort 2. The data on cloned sequences were deposited in GenBank: KC681847–KC681888. The deep sequencing data for the two cohorts were deposited in NCBI (Bioproject: PRJNA344431). seq., sequence.

middle part of the sequences that correspond to the core siRNA stretches. The data may indicate that the corresponding conserved regions are important for the function of HIV-1 proteins. One example that supports this conclusion is the target A2, which spans the region specifying the IVIYQYMD stretch in the RT enzyme (Figure S13). Residue M184 in this stretch lies close to the primer terminus and near to the binding site for the incoming nucleoside triphosphate (dNTP). M184 is a part of the dNTP-binding site of HIV-1 RT (dNTP-binding pocket) and is located in the highly conserved YMDD motif, which is found in all retroviruses.[33] Y183 in this motif contributes to both dNTP affinity and processivity of RT.[34]

The second reason for the collection of sequences possessing a much lower mutation rate in the middle part is the selection procedure used for the design of active siRNAs. It is known that the requirements for the core 19-nt sequence includes a number of characteristics associated with siRNA functionality: low G/C content, a bias toward low internal stability at the sense-strand 3′ terminus, lack of inverted repeats, and sense-strand base preferences (positions 3, 10, 13, and 19).[32] The regions in the range from 3 to 18 nt in the core 19-nt sequences from the 12 profiles are the most conserved (mutation frequencies below 10^{-4}; Figure 5). Therefore, we conclude that both the limitations due to the biological functions of HIV-1 proteins and the rules for the design of efficient siRNAs shaped the selected RNAi targets.

Characteristics of the Mutations inside the 27- to 30-bp DNA Sequences that Include the RNAi Targets

Mutations in HIV-1 have been studied for many years, and during the last decade, a novel possibility for the analysis of such mutations was provided by deep sequencing. Although there are some problems in using this approach for the complete genome assembly of the virus based on short read lengths,[35] the method is suitable for the detailed analysis of mutations in short genomic regions from viral populations. The main source of the high variability of HIV-1 is the error-prone nature of RT, although recently, it was discussed whether APOBECK3 proteins could also significantly contribute to the genetic diversity of HIV-1 inside human cells.[15,16]

We attempted to answer this question using deep sequencing reads for analysis of mutations. It is known that changes either in the HIV-1 RT or in the sequence of the nucleic acid template can affect the spectrum of mutations produced during viral replication.[36] From this point of view, the spectrum of mutations in our study, which is based on the wild-type RT in a population of viruses, could vary, depending only from sequences of different targets. As expected, we observed that the frequencies of particular mutations varied for different targets (Figures 6 and 7).

We calculated the frequencies in all nucleotide substitutions observed in the regions possessing the RNAi targets, as described in the Materials and Methods section. A3G, a single-stranded DNA cytidine deaminase, could generate G-to-A mutations in the plus strand of HIV-1 DNA. The data shown in Figures 6 and 7 indicate that the G-to-A mutations are frequent but were dominant only inside target A2 in cohort 2. Therefore, we conclude that A3G is not

Figure 2. Alignments of Deep Sequencing Reads from Both Cohorts

The top ten alignments are shown. The complete alignments are shown in Figures S1–S12. The reference sequence is shown on the top (GenBank: AF316544). The second line represents the sequences observed in the majority of reads. The number and percentage of reads are indicated. The 19-nt core RNAi sequences are not shaded.

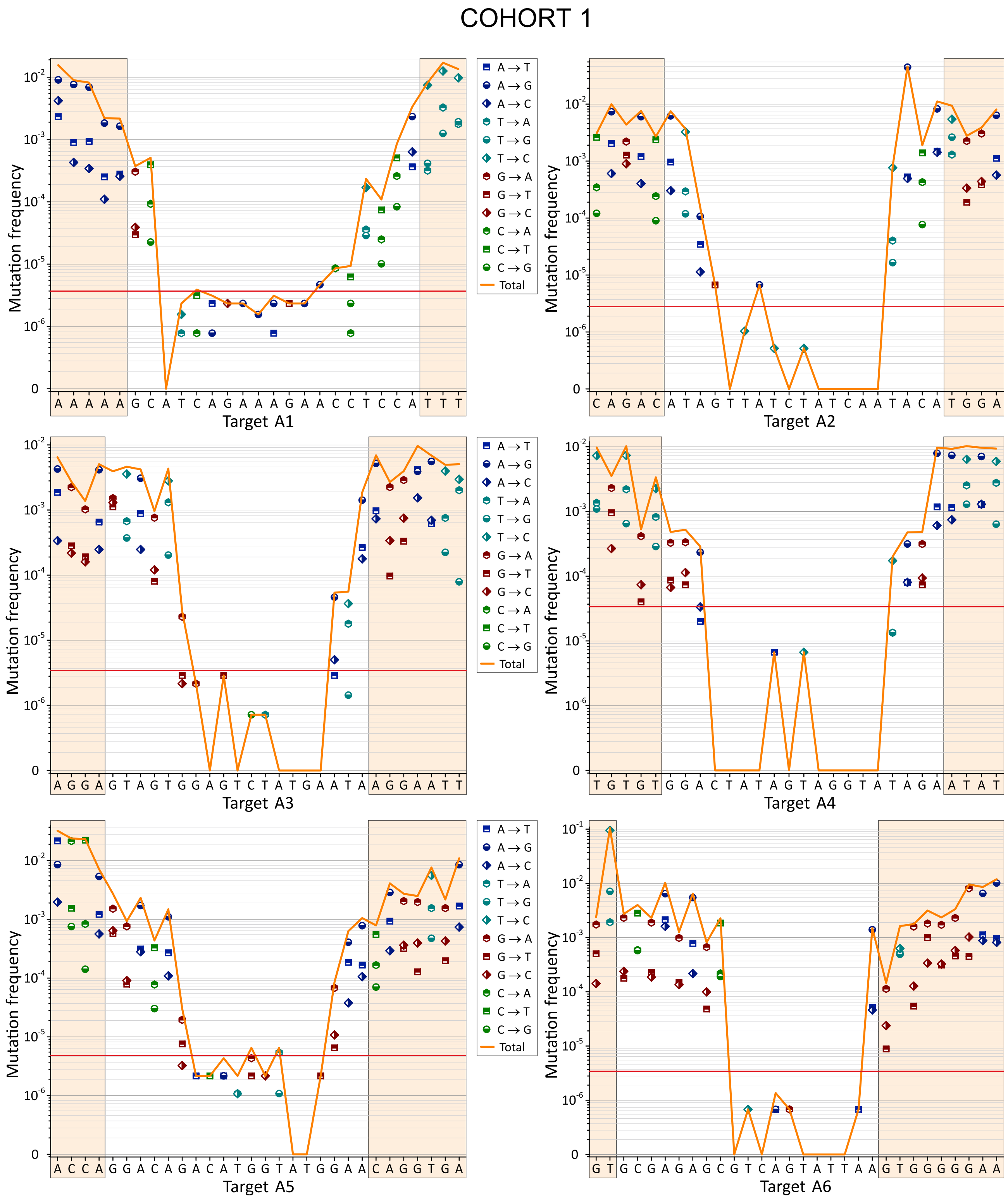

Figure 3. Curve Showing the Frequencies of Nucleotide Substitutions along the 27- to 30-nt Targets for Cohort 1

The horizontal red line corresponds to the threshold of reliable mutation detection (Equation 5 in Materials and Methods). The frequencies were determined against the most invariable RNAi target and were calculated by Equation 1 (Materials and Methods).

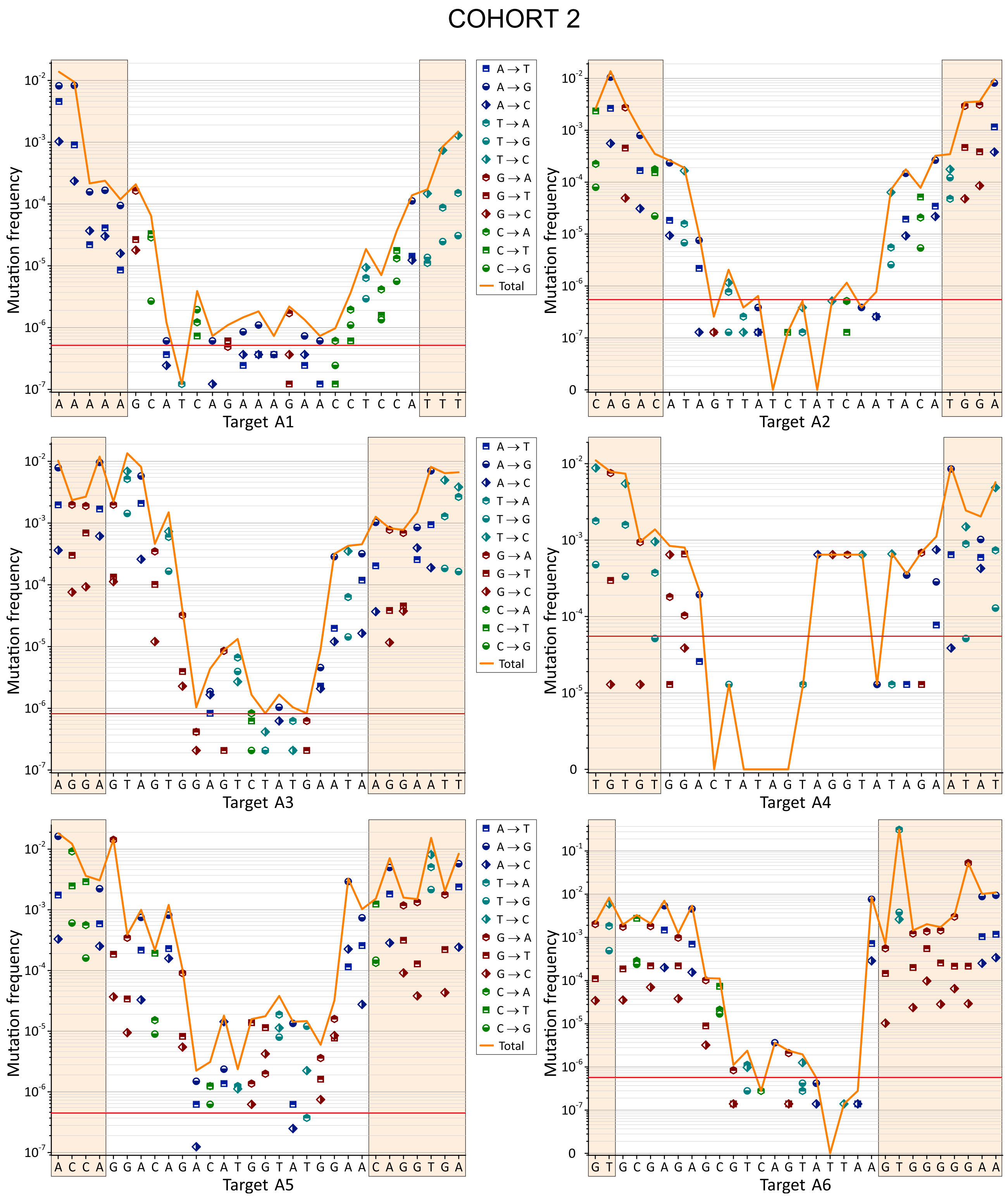

Figure 4. Curve Showing the Frequencies of Nucleotide Substitutions along the 27- to 30-nt Targets for Cohort 2

The horizontal red line corresponds to the threshold of reliable mutation detection (Equation 5 in Materials and Methods). The frequencies were determined against the most invariable RNAi target and were calculated by Equation 1 (Materials and Methods).

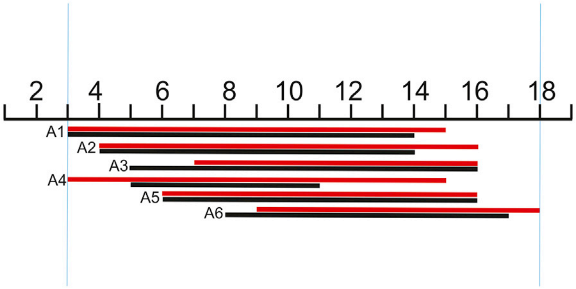

Figure 5. The Most Conserved Positions inside 19-nt RNAi Core Sequences
The regions possessing the mutation frequencies below 10^{-4} (see Figures 3 and 4) are shown between the blue lines. The values indicate the number of the nucleotide in a target. Cohort 1 is shown in red. Cohort 2 is shown in black.

the main source of the observed variability in the viruses isolated from plasma.

The main nucleotide substitutions observed were transitions T to C and A to G, which are the most frequent in all targets. The reverse transitions C to T or G to A occurred less frequently. We conclude that these mutations reflect the nature of the RT, which is prone to low fidelity in the discrimination between a pair of purines or pyrimidines. Some of the nucleotide substitutions, such as C to G and G to C, for all targets occur at a low rate. These bases are likely the most clearly discriminated by the enzyme. At the same time, we observed a strong sequence dependence of errors made by the RT. For example, targets A1 and A5 both possess six and five C bases, respectively, but the C-to-T transitions in them differ by 30- or 152-fold in cohorts 1 and 2, respectively. A more detailed analysis of mutations that reflects the nature of the RT will be published separately.

Y183 and M184 Variants of Mutations in the 3′ Flank of Target A2 Occur at High Frequencies

Of particular interest are the mutations in the target A2, which possesses the sequence that specifies the highly conserved YMDD motif that is found in all retroviruses (Figure S13). We selected patients that had been receiving antiretroviral therapy for up to 5 years but still possessed high-titer viremia (cohort 2) in order to check the putative changes inside the RNAi targets induced by the drugs. It is known that M184 is mutated to a valine (M184V) in response to lamivudine or emtricitabine treatment[37]. However, in the population of viruses from cohort 2, we observed only the M184I mutation at a high frequency (up to 2.3×10^{-3}; Table 2). In contrast, in cohort 1, we detected M184V at high frequencies (9×10^{-2}) as well as a number mutations due to substitutions in the second and third bases of the ATG codon: M184T (6×10^{-3}), M184R (1.7×10^{-3}), M184K (1.5×10^{-3}), and M184I (2.5×10^{-3}). M184 variants display a reduced replication capacity and transmission efficacy and tend to revert over time in the new host.[38]

Among Y183 variants, only one was detected at a high frequency: Y183C (5×10^{-3}) from cohort 1 (Table 2). This mutant has only 5% of the wild-type RT activity,[39] and the mutation likely spread such a high frequency with the aid of the wild-type RT during multiple infections of human cells.[40]

Dicer Substrates Corresponding to the Detected Targets Induce Efficient RNAi In Vitro

Deep sequencing data allowed us to select six conserved regions that were detected in the majority (from 92.39% to 99.58% in the 19-nt core sequences) of the HIV-1 subtype A viruses in both cohorts. The data led us to test the efficiency of RNAi triggered by 27-bp Dicer substrates corresponding to the detected targets. Dicer substrates demonstrate the same or better efficacy of RNAi compared with double-stranded siRNA formed by 21-nt RNAs.[41,42] Table 3 shows the sequences of chemically synthesized RNAs that were annealed to form 27-bp double-stranded molecules with blunt ends.

We used co-transfection of a plasmid-containing DNA fragment containing the 27-nt RNAi target, which was cloned into the psiCHECK-2 vector (Promega) and the corresponding Dicer substrate. The vector was specially designed for quantitative testing of RNAi efficacy. It possesses two *luc* reporter genes (one from firefly and one from *Renilla*). The DNA fragment containing the targets was cloned into the 3′ UTR of the *Renilla* gene, which is important for expression. Both reporter genes are expressed after transfection, but only the *Renilla* mRNA can be attacked by the RNAi. Figure 8 shows the results of co-transfection experiments, in which expression of the firefly gene was used for normalization of the *Renilla* luminescence. Consistent RNAi-mediated silencing was observed in the range 81%–91% for the different targets. The randomized sequences of the targets were not attacked by RNAi. The data strongly indicate that the Dicer substrates corresponding to the six RNAi targets are processed into efficient siRNAs.

Identical RNAi Targets Are Present in Many HIV-1 Isolates from Different Countries

The data on the existence of conserved HIV-1 sequences that are the same in two independent cohorts of HIV-infected patients from Russia and could serve as RNAi targets encouraged us to determine whether the identical targets are present in different isolates from around the world. It was of interest to estimate the percentage of HIV-1 sequences possessing these targets, similar to the deep sequencing reads that we performed in the course of this study. However, such a search is hampered because we cannot analyze all the HIV-1 databases by the same way. Therefore, we used a simple approach and exploited the regular BLAST search tool (https://blast.ncbi.nlm.nih.gov/Blast.cgi?PAGE=MegaBlast&PROGRAM=blastn&BLAST_PROGRAMS=megaBlast&PAGE_TYPE=BlastSearch&DBSEARCH=true&QUERY=&SUBJECTS =). We selected the Nucleotide collection (nr/nt) and Max target sequences as 5,000, assuming that this would permit the approximate estimation of the spread of the corresponding targets in different isolates. For the targets A1–A3, A4, and A6, we obtained only HIV-1 sequences among the 5,000 BLAST hits. In the case of A5, only sequences from Russia were observed among non-HIV-1 hits. Surprisingly, we

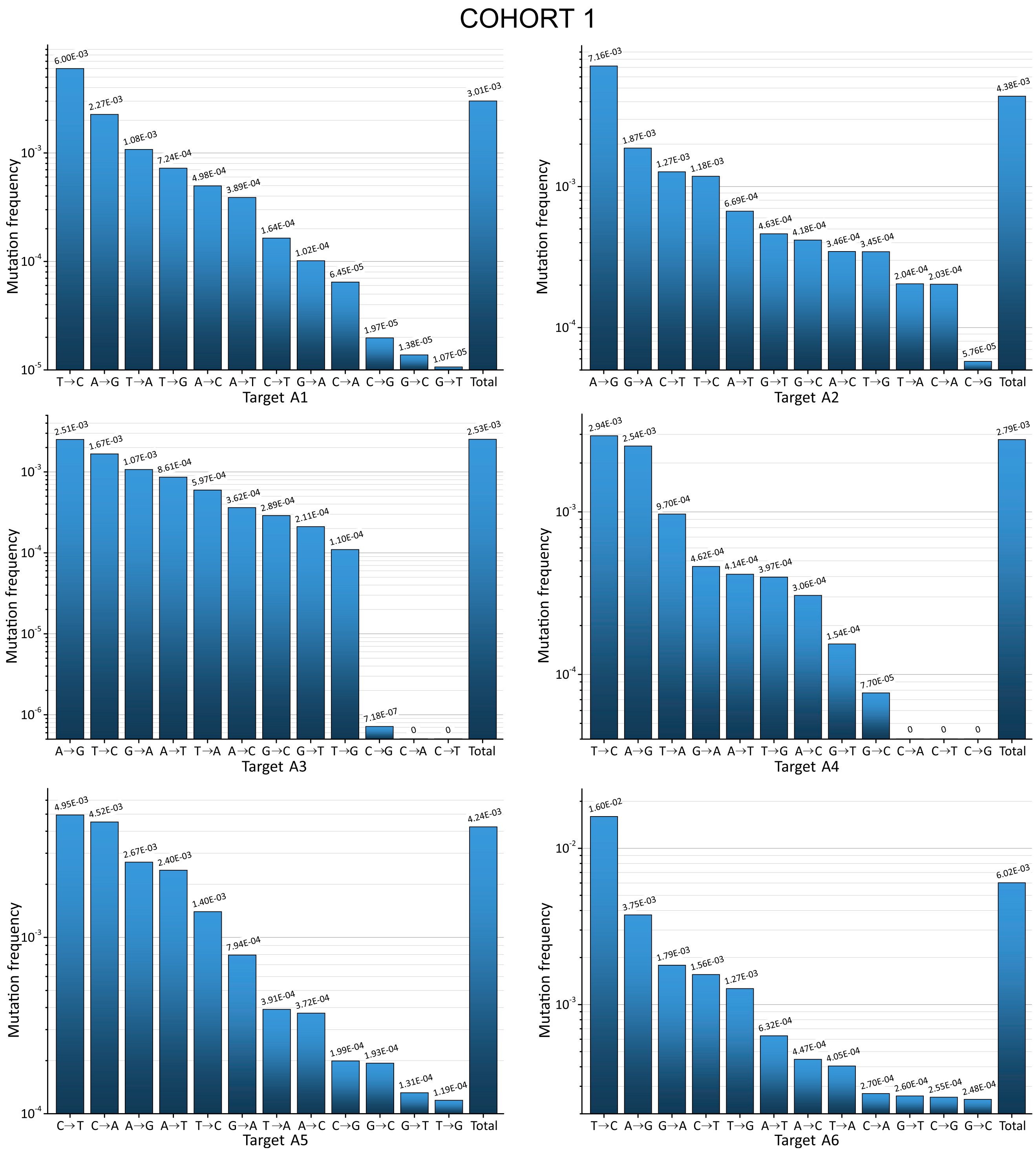

Figure 6. Frequencies of All Possible Nucleotide Substitutions along the 27- to 30-nt Targets for Cohort 1

The frequencies were determined against the most invariable RNAi target and were calculated by Equation 1 (Materials and Methods).

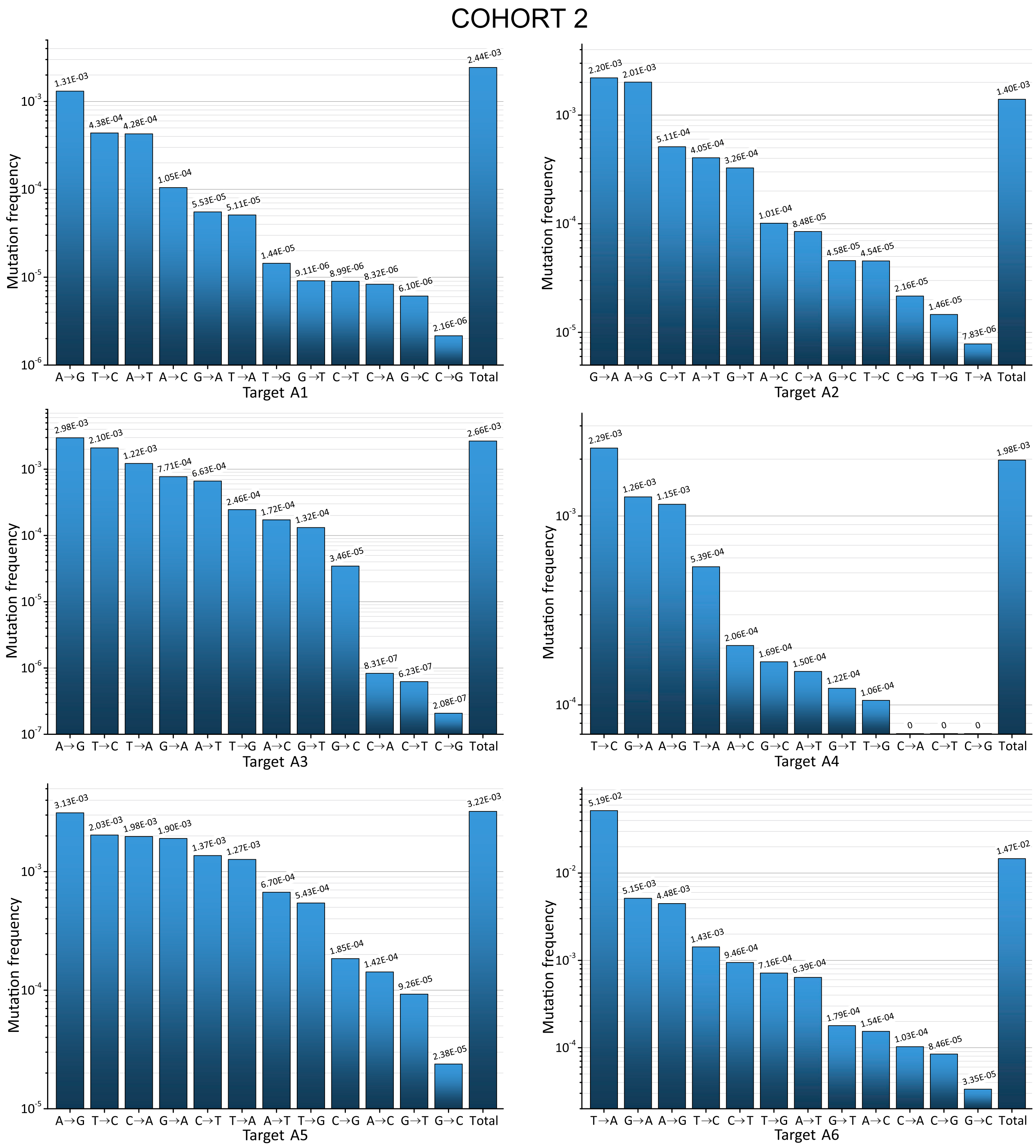

Figure 7. Frequencies of All Possible Nucleotide Substitutions along the 27- to 30-nt Targets for Cohort 2

The frequencies were determined against the most invariable RNAi target and were calculated by Equation 1 (Materials and Methods).

Table 2. Characteristics of the Mutations in Target A2 that Affect Y183 and M184 in RT

Amino Acid, Codon	First Base Mutations, Codons, Amino Acids/Frequencies	Second Base Mutations, Codons, Amino Acids/Frequencies	Third Base Mutations, Codons, Amino Acids/Frequencies
Cohort 1			
Y183, TAC	T to C, CAC, H/7·10^{-4}	A to G, TGC, C/5·10^{-2}	C to T, TAT, Y/1.5·10^{-3}
	T to A, AAC, N/4.5·10^{-5}	A to T, TTC, F/6·10^{-4}	C to A, TAA, Stop/4.5·10^{-4}
	T to G, GAC, D/1.8·10^{-5}	A to C, TCC, S/15.5·10^{-5}	C to G, TAG, Stop/8.5·10^{-5}
M184, ATG	A to G, GTG, V/9·10^{-2}	T to C, ACG, T/6·10^{-3}	G to A, ATA, I/2.5·10^{-3}
	A to T, TTG, L/1.8·10^{-3}	T to G, AGG, R/1.7·10^{-3}	G to C, ATC, I/3.7·10^{-4}
	A to C, CTG, L/1.7·10^{-3}	T to A, AAG, K/1.5·10^{-3}	G to T, ATT, I/2·10^{-4}
Cohort 2			
Y183, TAC	T to C, CAC, H/7·10^{-5}	A to G, TGC, C/1.5·10^{-4}	C to T, TAT, Y/6·10^{-5}
	T to A, AAC, N/6·10^{-5}	A to T, TTC, F/2·10^{-5}	C to A, TAA, Stop/2·10^{-5}
	T to G, GAC, D/3·10^{-6}	A to C, TCC, S/9·10^{-6}	C to G, TAG, Stop/5.5·10^{-6}
M184, ATG	A to G, GTG, V/3·10^{-4}	T to C, ACG, T/1.0·10^{-4}	G to A, ATA, I/2.3·10^{-3}
	A to T, TTG, L/3·10^{-5}	T to G, AGG, R/3·10^{-5}	G to C, ATC, I/5.2·10^{-4}
	A to C, CTG, L/2·10^{-5}	T to A, AAG, K/5·10^{-5}	G to T, ATT, I/5·10^{-5}

found that targets identical to the 19-bp core sequences of A1–A3, A4, and A6 were detected in different HIV-1 isolates from different countries on all continents (Table 4). Of course, the data strongly depend upon the number of isolates that were sequenced from a particular country. Nevertheless, the data strongly suggest that the identical targets A1, A2, and A6 are widely dispersed. We detected 100% identical sequences in the majority of the BLAST hits: in 4,956 HIV-1 sequences (99%) for A6, 4,837 sequences (96.7%) for A2, and 4,651 sequences (93%) for A1. The A3 target was not so widespread and was detected in 1,395 sequences (27.9%). The A2 target is very abundant in isolates from USA, UK, Switzerland, and Germany. The target A6 is characteristic of isolates from Kenya, USA, UK, Switzerland, and Thailand.

Next, we decided to use the Los Alamos database, which includes all HIV sequences available worldwide (http://www.hiv.lanl.gov). Tables S3–S7 shows the results of the HIV BLAST search using a specialized HIV sequence database (https://www.hiv.lanl.gov/content/sequence/BASIC_BLAST/basic_blast.html). The tool allows the search of up to only 200 HIV-1 sequences. The data indicate that the identical targets are present in all 200 tested sequences. Interestingly, we observed that these targets were found in HIV-1 strains from different countries and corresponded to different subtypes, including 11 subtypes for A4 and 20 subtypes for A6. For example, the list for A6 includes subtypes A1, A1C, A1D, A1CD, B, 01B, 01BC, C, G, 01_AE, F1, 69_01B, 70_BF1, 58_01B, 56_cpx, 35_AD, 65_cpx, BF1, 28_BF, and 29_BF. The Los Alamos HIV database includes all available HIV sequences worldwide, including the full-length HIV-1 genomic sequences. This comprehensive collection of HIV sequences is important for both the choice of the most conserved protein sequences for vaccine design and selection of siRNA sequences.[26,43] The deep sequencing approach for selecting the conserved RNAi targets that we used is limited by the use of viruses from a small number of patients and thus does not provide a global representation; however, our method reveals the diversity of millions of HIV variants that are present in every patient of our cohorts. Deep sequencing is also suitable for the detailed analysis of RT biases. Both approaches could be combined. The data shown in Tables S3–S7 indicate that the HIV samples of different subtypes from the Los Alamos database possess sequences that are identical to the RNAi targets detected by deep sequencing. Therefore, we conclude that the targets detected by deep sequencing in about 90% of HIV-1 isolates from Russia are also present in many HIV-1 isolates worldwide.

DISCUSSION

The data based on the analysis of about 7×10^6 aligned reads representing two independent cohorts of HIV-infected patients strongly support the conclusion that the studied isolates possess six conserved regions that could serve as RNAi targets. Surprisingly, in such a highly mutable virus, we detected the identical targets in both cohorts. Up to 99.5% of the viruses analyzed possessed the same six 19-bp RNAi targets. Moreover, many HIV-1 isolates corresponding to diverse subtypes from different countries contain the identical sequences in the A1, A2, and A6 regions, so these could potentially be used as the targets for RNAi-based gene therapy. These particular sequences have great potential for the development of an artificial RNAi approach because they were found in 93%–99% of 5,000 BLAST hits. It has been described that using two targets that are conserved in up to 80% of viruses could provide efficient silencing in up to 95% of viruses.[31] Of course, for the development of this approach, one should solve the very important problem of the delivery of Dicer substrates, siRNA, or genetic constructs expressing the corresponding small hairpin RNAs. Nevertheless, these results suggest a way of monitoring current HIV-1 strains, even by low-scale deep sequencing of the six RNAi targets described in the patients from different countries, and to select the detected

Table 3. RNA Sequences that Were Used for the Preparation of Dicer Substrates and the DNA Sequence Containing Six RNAi Targets

Target	Domain	Target Sequence, 5′-3′	RNAs, 5′-3′
A1	RT	AAAAAGCATCAGAAAGAACCTCCATTT	AAAAAGCAUCAGAAAGAACCUCCAUUU
			AAAUGGAGGUUCUUUCUGAUGCUUUUU
A2	RT	AGACATAGTTATCTATCAATACATGGA	AGACAUAGUUAUCUAUCAAUACAUGGA
			UCCAUGUAUUGAUAGAUAACUAUGUCU
A3	Int	AGGAGTAGTGGAGTCTATGAATAAGGA	AGGAGUAGUGGAGUCUAUGAAUAAGGA
			UCCUUAUUCAUAGACUCCACUACUCCU
A4	vpu	GTGTGGACTATAGTAGGTATAGAATAT	GUGUGGACUAUAGUAGGUAUAGAAUAU
			AUAUUCUAUACCUACUAUAGUCCACAC
A5	gp120	ACCAGGACAGACATGGTATGGAACAGG	ACCAGGACAGACAUGGUAUGGAACAGG
			CCUGUUCCAUACCAUGUCUGUCCUGGU
A6	p17	GTGCGAGAGCGTCAGTATTAAGTGGGG	GUGCGAGAGCGUCAGUAUUAAGUGGGG
			CCCCACUUAAUACUGACGCUCUCGCAC

The 19-nt core sequences are underlined. The pairs of RNA molecules are shown in the 5′-3′ direction. These pairs could form 27-bp double-stranded RNAs (Dicer substrates).

predominant variants of sequences for the targeted RNAi. In our preliminary experiments, we used the analysis of a limited number of cloned sequences and found targets that are present in the majority of viruses in cohort 1.[29–31]

The analysis of ultra-deep sequencing data of HIV-1 variants provides important information regarding the nature of RT. The nucleotide biases of this error-prone enzyme could be uncovered and may lead to a better understanding of the mechanisms responsible for the main source of HIV-1 variability. The HIV-1 genome is hypermutated by APOBEC3G in blood cells, but only a very small fraction of these mutations reach the plasma, indicating that many viruses are defective as a result of the extremely high mutation load and are incompatible with the release of viral particles.[15] In our experiments, we used the HIV-1 variants that were released into the plasma. Although G-to-A transitions, which are characteristic consequences of APOBEC3G action, were observed in the range between 5.53×10^{-5} and 5.15×10^{-3}, they were not predominant compared with other mutations that happened at much higher frequencies. For example, the most frequent transversion T to A reached 5.19×10^{-2} in A6 from cohort 2 (Figure 7), and the most frequent transition T to C was 1.67×10^{-2} in A3 from cohort 1 (Figure 6). The result is consistent with the recent conclusion that APOBEC3-induced G-to-A hypermutation provides only a small contribution to HIV-1 genetic variation in the viruses isolated from plasma.[16] It cannot be excluded that RT also contributes to lethal mutations and the corresponding genomes cannot reach the plasma. Currently, we are performing a detailed analysis of the lethal mutations in the deep sequencing reads and mutations that are compatible with the release of viral particles (data to be published separately).

It is known that M184V and M184I mutations lead to drug resistance and, at the same time, dramatically decrease the processivity of RT.[38] The patients from cohort 2 were treated with lamivudine for between 3 and 5 years. Surprisingly, we observed a very high level of the M184V mutation in cohort 1 (up to 9×10^{-2}), which was not expected to contain a lot of drug-resistant HIV-1 variants because the corresponding patients were not receiving antiretroviral therapy. On the other hand, in cohort 2, which was supposed to contain drug-resistant variants of HIV-1, the M184V and M184I mutations were detected at frequencies of 3×10^{-4} and up to 2.3×10^{-3}, respectively. Previously, it was shown that due to the mutational bias of RT, the M184I (ATA) variant in lamivudine-treated patients is usually observed first because there is a higher frequency of G-to-A substitutions toward the ATA codon than the A-to-G substitutions toward GTG (184V).[44] However, later, due to the higher processivity of the M184V enzyme,[45] the virus possessing this mutation outgrew. This is a possible explanation of the data observed in cohort 2. Nevertheless, the data strongly suggest that these mutations can emerge without antiretroviral therapy.

We observed that the most frequent nucleotide substitutions were transitions and that RT rather poorly discriminates between two variants of pyrimidines (T or C) or purines (A or G), which may explain the rapid reversions of M184V in the new host.[38]

The frequencies of nucleotide substitutions that were observed in the range of up to 10^{-2} inside 27- to 30-bp sequences still remain the mutation rate inside the core 19-nt sequences below 10^{-5}. This fact strongly suggests that these regions have biological constraints for mutations, which is why we conclude that the selected regions could be used as the targets of RNAi.

HIV-1 mRNA may have secondary structures and this could restrict RNAi action. RNA-protein complexes could also mask a target sequence and prevent recognition by the corresponding siRNA. To address these questions, we will analyze in future studies the selected

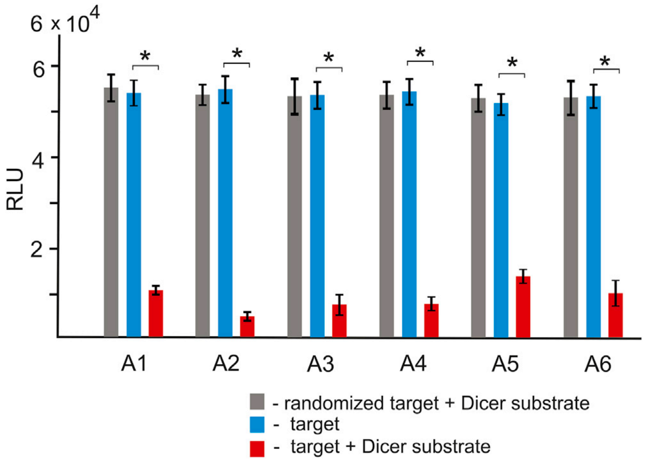

Figure 8. Efficiency of RNAi Initiated by the Dicer Substrates

Results of co-transfection experiments are shown (see Materials and Methods). Luminescence of the *Renilla luc* gene (RLU) was normalized to that of the firefly *luc* gene. *p < 0.005.

RNA targets further using a viral system, in which the virus will be isolated from the patients described in the current study.

MATERIALS AND METHODS

RT-PCR and Preparation of Libraries

The RNA preparations were provided by the State Research Center of Virology and Biotechnology Vector (Russia) from their collection of isolates. Cohort 1 included five isolates of HIV-1 subtype A (10RU6587, 11RU6933, 11RU6949, 10RU6483, and 11RU1996) from patients who were not receiving antiretroviral therapy. Cohort 2 included four isolates (45788, 11; 2229, 13; MZ; and QN). The cohort was assumed to contain drug-resistant viruses. The patients from cohort 2 received antiretroviral therapy for 5 years and still possessed high-titer viremia and therefore the cohort was considered to include drug-resistant HIV-1 variants. Written informed consent was obtained from each participant. Blood samples were linked with demographic and clinical data via coded ID numbers according to the requirements of medical ethics in Russia. The study of clinical blood samples was in compliance with the ethical standards of the Helsinki Declaration of 1975 and the requirements of Model Law no. 26-10 "On the protection of human rights and dignity in biomedical research in the member states of the Commonwealth of Independent States" (October 18, 2005).

The RNA preparations were extracted individually from 500 μL of plasma samples using ViroSeq reagents (Celera Diagnostics) and then treated with DNase using a DNA-free kit (Ambion) as described previously.[46,47] The concentration of RNA preparations was measured using a NanoDrop 2000.

Five (cohort 1) or four (cohort 2) RNA preparations were pooled (6 ng of each individual RNA preparation) and used for RT-PCR. Approximately 15 ng of total RNA and M-MLV RT were used to synthesize cDNAs by use of a DNA-free kit (Ambion) according to the manufacturer's instructions. cDNAs corresponding to two regions in the RT domain and to one region in the integrase domain from the *pol* gene as well as the conserved region from the *vpu* gene, the conserved region from the gp120 domain of the *env* gene, and the p17 domain from the *gag* gene were synthesized using the primers indicated in Table S2. Nested PCR was used for amplification of regions of approximately 300 bp that contained the six selected RNAi targets located at a distance of 20–50 bp from the 5′ end of the amplified DNA fragment. Primers were selected using the Primer Selection Tool (http://biotools.umassmed.edu/). The conditions for PCR for each set of primers were determined in preliminary experiments using a Mastercycler personal PCR instrument (Eppendorf). The identity of the amplified DNA fragments was confirmed by cloning and capillary sequencing.

Amplified DNA was used for the preparation of the library. Libraries were prepared according to Illumina's instructions that accompany the DNA Sample Kit or the NEBNext Ultra DNA Library Prep Kit for Illumina. Briefly, DNA was end repaired using a combination of T4 DNA polymerase, *E. coli* DNA Pol I large fragment (Klenow polymerase), and T4 polynucleotide kinase. The blunt, phosphorylated ends were treated with Klenow fragment and dATP to yield a protruding 3′ A base for ligation of Illumina adapters, which have a single T base overhang at the 3′ end. After adaptor ligation, DNA was PCR amplified with Illumina primers for 15 cycles. Deep sequencing was performed using an Illumina Genome Analyzer IIx (cohort 1) or paired-end reads (2 × 150) produced by an Illumina HiSeq 1500 (cohort 2). The data were deposited in NCBI (Bioproject: PRJNA344431).

Data Processing

The analysis of multiple related variants of rapidly evolving HIV-1 viruses with ultra-deep sequencing comprising more than 1 million reads can be performed with a bioinformatic pipeline (for a recent review, see Leipizig[48] and Posada-Cespedesa et al.[49]). In a bioinformatic pipeline, the data are consecutively processed by an organized package of programs, such that the data at the output of one program are transferred to the input of another program. We tried several variants and finally created the following pipeline. The raw sequenced reads were first evaluated by their quality using FastQC.[50] Then, the reads of too-short length (<20 nt) and low quality (q < 26) were filtered out from the initial set using Cutadapt.[51] The invariable elements that could subsequently be used as a natural reference for the evaluation of mutation and microindel rates were searched for via a two-step iterative procedure. In the first step, the sequences for HIV-1 isolate 97CDKP58e from the Republic of the Congo (GenBank: AF316544) was used as a generic reference for HIV-1 subtype A sequences and aligned against a sampling set of 1,000 reads. The results of the alignment were analyzed by our own ad hoc Perl script to detect the most invariable sequence. In the second step, this sequence was used as the reference against the complete set of reads using Bowtie 2.[52] All non-aligned reads were filtered out by SAMtools.[53] The resulting alignment data

Table 4. Spread of the Identical Targets in HIV-1 Isolates from Different Countries

Target/Domain	19-nt Core Sequence	Number of Identical BLAST Hits	Country
A1/RT	GCATCAGAAAGAACCTCCA	4,651	Russia, USA, Uzbekistan, Spain, Malaysia, Kenya, Rwanda, Ukraine, China, Uganda, Cameroon, Nigeria, South Africa, South Korea, Hungary, Iran, Brazil, UK, Germany, Morocco, Ethiopia, Senegal, Mexico, Togo, Panama, French Guiana, Belgium, Angola, Sierra Leone, Ghana, Thailand, D.R. Congo, Burundi, Puerto Rico, Australia, Poland, Algeria, Israel, Cuba, India, Botswana, Jamaica, Turkey, Tanzania, Bulgaria, Cyprus, Portugal
A2/RT	ATAGTTATCTATCAATACA	4,837	Russia, USA, South Africa, France, Uzbekistan, Spain, Malaysia, Kenya, South Korea, UK, Peru, China, Switzerland, Germany, Uganda, Cameroon, Surinam, Brazil, Mexico, Kuwait
A3/Int	GTAGTGGAGTCTATGAATA	1,395	Russia, Uzbekistan, Rwanda, Ukraine, Kenya, Spain, Zambia, Armenia, Kirgizstan, Kazakhstan, France, Uganda, Canada, South Africa, UK, Cyprus, Cameroon, Germany, Belgium, Cuba, Georgia
A4/Vpu	GGACTATAGTAGGTATAGA	768	Russia, Rwanda, Ukraine, Iran, Nigeria, Uganda, D.R. Congo, Kenya, Spain, Cameroon, India, China, Myanmar, Thailand, Cyprus, Afghanistan, USA, Viet Nam, South Africa, Denmark, Tanzania, Kazakhstan, Uzbekistan, Georgia, Botswana
A5/Gp120	GGACAGACATGGTATGGAA	–	Russia
A6/P17	GCGAGAGCGTCAGTATTAA	4,956	USA, Thailand, Russia, Kenya, Rwanda, Cameroon, Pakistan, Ukraine, Brazil, China, India, Nigeria, Cameroon, Greece, Tanzania, Spain, Uganda, Senegal, UK, South Africa, Germany, Botswana, Portugal, D.R. Congo, Burkina Faso, France, Zambia, Cuba, India, Guinea-Bissau, Sweden, Estonia, Malaysia, Iran, Canada, Myanmar, Switzerland, Netherlands, South Korea, Malawi, Poland

The search using nucleotide BLAST (Nucleotide collection [nr/nt]) was performed using the 19-nt core sequences. The number of identical hits is indicated. The list of countries is shown in the order of the number of corresponding hits that appeared in the list. The search was performed for the maximum of 5,000 target sequences with automatically adjusted parameters for short input sequences. The target A5 was detected only in Russia.

were converted to Fasta format by UCSC Genome tools.[54] All mutants detected by pairwise alignment of each read from the Fasta file against the reference target sequence were collected into an array suitable for further multiple alignments. The multiple alignments were performed using MAFFT software.[55] To analyze the results of the multiple alignment and calculate the corresponding mutation rates and related statistics, we developed a Perl script in-house. This script has output options to visualize the results in terms of multiple alignments or phylogenetic trees using the Ugene toolkit.[56]

In our analysis, RNAi targets were appended by short fragments from both sides of each target, so the total length of the analyzed fragments was about 30 nt. For brevity, these longer fragments will also be termed RNAi targets. In this paper, the frequencies of nucleotide substitutions were determined against the sets of aligned sequences, which is reasonable in the case of deep sequencing data. The frequency of mutations in a particular site of the reference target (corresponding to the most invariable fragment as described above), $f_{N\to N';\mathrm{site}}$, was assessed against the set obtained by multiple alignment. The frequency of particular replacements, $N\to N'$, N = (A, C, G, T), across the RNAi target was calculated as:

$$f_{N\to N'}=\frac{1}{n_N}\sum_{\mathrm{sites}}f_{N\to N';\mathrm{site}}, \quad \text{(Equation 1)}$$

where n_N is the total number of nucleotides of type N within the RNAi target, and the summation in Equation 1 is performed across the sites occupied by the nucleotides of type N. The total frequency of mutations across the RNAi target was determined as:

$$f_m=\sum_N\sum_{N'\neq N}f_Nf_{N\to N'}, \quad \text{(Equation 2)}$$

where $f_N=n_N/L_{\mathrm{target}}$ is the frequency of nucleotides of type N within the RNAi target, L_{target} is the target length, and $f_{N\to N'}$ is defined by Equation 1.

The expected dispersion of replacements in a particular site may be assessed by binomial distribution[57,58]

$$\sigma^2_{\mathrm{site}}=f_{N\to N';\mathrm{site}}\left(1-f_{N\to N';\mathrm{site}}\right)/n_{seq}, \quad \text{(Equation 3)}$$

where n_{seq} is the total number of the sequences in the multiple alignment set. For sensitivity of mutation detection, using the criterion

$$1.96\sigma\left(f_{thr}\right)=f_{thr}, \quad \text{(Equation 4)}$$

(Pr = 0.05) yields at the large n_{seq} the threshold detection rate

$$f_{thr}\approx 3.84/n_{seq}. \quad \text{(Equation 5)}$$

This threshold determines the rate of reliable detection for replacement $N\to N'$ at a particular site at the given number of aligned reads n_{seq}. This estimate yields $f_{thr}\approx 3.8\times 10^{-6}$ for $n_{seq}=10^6$. The statistical significance between the counterpart replacements at a particular site for two different datasets (in our work, these are the sets for the cohorts

of non-resistant [cohort 1] and drug-resistant patients [cohort 2]) can be assessed by the Gaussian z-criterion for the fractions.[57] The similar criterion can also be applied to the statistical assessment of the difference between frequencies of replacements at different sites. As seen from Equation 2, at $f_{N \to N';\text{site}} = 10^{-4}$ and $n_{seq} = 10^6$, the difference of approximately $\Delta f_{N \to N';\text{site}} \approx 10^{-5}$ can be resolved between the counterpart replacements $N \to N'$ at a particular site.

Transfection Assays

The 27-bp targets shown in Table 3 were cloned into *Xho* I and *Not* I cloning sites inside the 3′ UTR of the *Renilla* gene of the psiCHECK-2 vector. Cultured HEK293T cells were plated 1 day prior to transfection (5×10^4 cells per 24-well culture dish). For co-transfection experiments, Lipofectamine 3000 Transfection Reagent (ThermoFisher Scientific) was used according to the manufacturer's instructions. Per well, we used 15 ng of the experimental DNA constructs containing a target of RNAi cloned into psiCHECK-2 mixed with 12.5 pmol of a Dicer substrate in 25 μL of serum-free medium (SFM). Lipofectamine reagent was mixed with 25 μL of SFM and mixed by vortex for 2–3 s. Both solutions were mixed and then 0.2 μL of P3000 reagent was added, and the final solution was mixed by vortex for 2–3 s and incubated for 5 min at room temperature. After the incubation, the solution was added to the cells in 500 μL of DMEM. The cells were incubated for 48 hr. Firefly and *Renilla* luciferase activities were measured using the Dual-Luciferase Reporter Assay System (Promega) and a Reporter Microplate Luminometer (Turner BioSystems). The *Renilla* luciferase data were normalized to the firefly luciferase data. Excel and Origin software were used for data analysis.

SUPPLEMENTAL INFORMATION

Supplemental Information includes thirteen figures and seven tables and can be found with this article online at http://dx.doi.org/10.1016/j.omtn.2017.07.010.

AUTHOR CONTRIBUTIONS

O.V.K. and N.A.T. conceived and supervised the study; D.M.F., O.V.K., N.A.T., M.A.G., N.M.G., and M.P.G. performed the experiments; V.R.C. and Y.V.K. analyzed data; and N.A.T. wrote the manuscript.

ACKNOWLEDGMENTS

We thank Dr. A.V. Kudryavtseva and Dr. A.V. Snezhkina for help with sequencing and Y.N. Toropchina and D.S. Vinogradov for technical assistance. This work was supported by a grant from the Russian Science Foundation (Project No. 15-14-00005).

REFERENCES

1. Lee, N.S., Dohjima, T., Bauer, G., Li, H., Li, M.J., Ehsani, A., Salvaterra, P., and Rossi, J. (2002). Expression of small interfering RNAs targeted against HIV-1 rev transcripts in human cells. Nat. Biotechnol. *20*, 500–505.

2. Novina, C.D., Murray, M.F., Dykxhoorn, D.M., Beresford, P.J., Riess, J., Lee, S.K., Collman, R.G., Lieberman, J., Shankar, P., and Sharp, P.A. (2002). siRNA-directed inhibition of HIV-1 infection. Nat. Med. *8*, 681–686.

3. Jacque, J.M., Triques, K., and Stevenson, M. (2002). Modulation of HIV-1 replication by RNA interference. Nature *418*, 435–438.

4. Coburn, G.A., and Cullen, B.R. (2002). Potent and specific inhibition of human immunodeficiency virus type 1 replication by RNA interference. J. Virol. *76*, 9225–9231.

5. Capodici, J., Karikó, K., and Weissman, D. (2002). Inhibition of HIV-1 infection by small interfering RNA-mediated RNA interference. J. Immunol. *169*, 5196–5201.

6. Park, W.S., Miyano-Kurosaki, N., Hayafune, M., Nakajima, E., Matsuzaki, T., Shimada, F., and Takaku, H. (2002). Prevention of HIV-1 infection in human peripheral blood mononuclear cells by specific RNA interference. Nucleic Acids Res. *30*, 4830–4835.

7. Surabhi, R.M., and Gaynor, R.B. (2002). RNA interference directed against viral and cellular targets inhibits human immunodeficiency virus type 1 replication. J. Virol. *76*, 12963–12973.

8. Yamamoto, T., Omoto, S., Mizuguchi, M., Mizukami, H., Okuyama, H., Okada, N., Saksena, N.K., Brisibe, E.A., Otake, K., and Fuji, Y.R. (2002). Double-stranded nef RNA interferes with human immunodeficiency virus type 1 replication. Microbiol. Immunol. *46*, 809–817.

9. Hu, W.Y., Myers, C.P., Kilzer, J.M., Pfaff, S.L., and Bushman, F.D. (2002). Inhibition of retroviral pathogenesis by RNA interference. Curr. Biol. *12*, 1301–1311.

10. Stevenson, M. (2003). Dissecting HIV-1 through RNA interference. Nat. Rev. Immunol. *3*, 851–858.

11. Pathak, V.K., and Temin, H.M. (1990). Broad spectrum of in vivo forward mutations, hypermutations, and mutational hotspots in a retroviral shuttle vector after a single replication cycle: substitutions, frameshifts, and hypermutations. Proc. Natl. Acad. Sci. USA *87*, 6019–6023.

12. Lecossier, D., Bouchonnet, F., Clavel, F., and Hance, A.J. (2003). Hypermutation of HIV-1 DNA in the absence of the Vif protein. Science *300*, 1112.

13. Harris, R.S., Sheehy, A.M., Craig, H.M., Malim, M.H., and Neuberger, M.S. (2003). DNA deamination: not just a trigger for antibody diversification but also a mechanism for defense against retroviruses. Nat. Immunol. *4*, 641–643.

14. Jern, P., Russell, R.A., Pathak, V.K., and Coffin, J.M. (2009). Likely role of APOBEC3G-mediated G-to-A mutations in HIV-1 evolution and drug resistance. PLoS Pathog. *5*, e1000367.

15. Cuevas, J.M., Geller, R., Garijo, R., López-Aldeguer, J., and Sanjuán, R. (2015). Extremely high mutation rate of HIV-1 *in vivo*. PLoS Biol. *13*, e1002251.

16. Delviks-Frankenberry, K.A., Nikolaitchik, O.A., Burdick, R.C., Gorelick, R.J., Keele, B.F., Hu, W.S., and Pathak, V.K. (2016). Minimal contribution of APOBEC3-induced G-to-A hypermutation to HIV-1 recombination and genetic variation. PLoS Pathog. *12*, e1005646.

17. Berkhout, B., and de Ronde, A. (2004). APOBEC3G versus reverse transcriptase in the generation of HIV-1 drug-resistance mutations. AIDS *18*, 1861–1863.

18. Kim, E.Y., Bhattacharya, T., Kunstman, K., Swantek, P., Koning, F.A., Malim, M.H., and Wolinsky, S.M. (2010). Human APOBEC3G-mediated editing can promote HIV-1 sequence diversification and accelerate adaptation to selective pressure. J. Virol. *84*, 10402–10405.

19. Pusch, O., Boden, D., Silbermann, R., Lee, F., Tucker, L., and Ramratnam, B. (2003). Nucleotide sequence homology requirements of HIV-1-specific short hairpin RNA. Nucleic Acids Res. *31*, 6444–6449.

20. Senserrich, J., Pauls, E., Armand-Ugón, M., Clotet-Codina, I., Moncunill, G., Clotet, B., and Esté, J.A. (2008). HIV-1 resistance to the anti-HIV activity of a shRNA targeting a dual-coding region. Virology *372*, 421–429.

21. Liu, Y.P., Haasnoot, J., ter Brake, O., Berkhout, B., and Konstantinova, P. (2008). Inhibition of HIV-1 by multiple siRNAs expressed from a single microRNA polycistron. Nucleic Acids Res. *36*, 2811–2824.

22. ter Brake, O., 't Hooft, K., Liu, Y.P., Centlivre, M., von Eije, K.J., and Berkhout, B. (2008). Lentiviral vector design for multiple shRNA expression and durable HIV-1 inhibition. Mol. Ther. *16*, 557–564.

23. Churikov, N.A., Gashnikova, N.M., Kretova, O.V., and Pokrovskiĭ, A.G. (2006). [Gene silencing of HIV-1 using genetic constructs expressing siRNAS]. Mol. Biol. (Mosk.) *40*, 784–787.

24. Naito, Y., Nohtomi, K., Onogi, T., Uenishi, R., Ui-Tei, K., Saigo, K., and Takebe, Y. (2007). Optimal design and validation of antiviral siRNA for targeting HIV-1. Retrovirology *4*, 80.

25. von Eije, K.J., ter Brake, O., and Berkhout, B. (2008). Human immunodeficiency virus type 1 escape is restricted when conserved genome sequences are targeted by RNA interference. J. Virol. *82*, 2895–2903.

26. Bobbin, M.L., Burnett, J.C., and Rossi, J.J. (2015). RNA interference approaches for treatment of HIV-1 infection. Genome Med. *7*, 50.

27. Klemm, V., Mitchell, J., Cortez-Jugo, C., Cavalieri, F., Symonds, G., Caruso, F., Kelleher, A.D., and Ahlenstiel, C. (2016). Achieving HIV-1 control through RNA-directed gene regulation. Genes (Basel) *7*, E119.

28. Karn, J., and Stoltzfus, C.M. (2012). Transcriptional and posttranscriptional regulation of HIV-1 gene expression. Cold Spring Harb. Perspect. Med. *2*, a006916.

29. Tchurikov, N.A., Fedoseeva, D.M., Gashnikova, N.M., Sosin, D.V., Gorbacheva, M.A., Alembekov, I.R., Chechetkin, V.R., Kravatsky, Y.V., and Kretova, O.V. (2016). Conserved sequences in the current strains of HIV-1 subtype A in Russia are effectively targeted by artificial RNAi in vitro. Gene *583*, 78–83.

30. Kravatsky, Y.V., Chechetkin, V.R., Fedoseeva, D.M., Gorbacheva, M.A., Kretova, O.V., and Tchurikov, N.A. (2016). [Mutation frequencies in HIV-1 subtype-A genome in regions containing efficient RNAi targets]. Mol. Biol. (Mosk.) *50*, 480–485.

31. Kretova, O.V., Chechetkin, V.R., Fedoseeva, D.M., Kravatsky, Y.V., Sosin, D.V., Alembekov, I.R., Gorbacheva, M.A., Gashnikova, N.M., and Tchurikov, N.A. (2016). Analysis of variability in HIV-1 subtype A strains in Russia suggests a combination of deep sequencing and multitarget RNA interference for silencing of the virus. AIDS Res. Hum. Retroviruses *33*, 194–201.

32. Reynolds, A., Leake, D., Boese, Q., Scaringe, S., Marshall, W.S., and Khvorova, A. (2004). Rational siRNA design for RNA interference. Nat. Biotechnol. *22*, 326–330.

33. Garforth, S.J., Lwatula, C., and Prasad, V.R. (2014). The lysine 65 residue in HIV-1 reverse transcriptase function and in nucleoside analog drug resistance. Viruses *6*, 4080–4094.

34. Harris, D., Yadav, P.N., and Pandey, V.N. (1998). Loss of polymerase activity due to Tyr to Phe substitution in the YMDD motif of human immunodeficiency virus type-1 reverse transcriptase is compensated by Met to Val substitution within the same motif. Biochemistry *37*, 9630–9640.

35. Mangul, S., Wu, N.C., Mancuso, N., Zelikovsky, A., Sun, R., and Eskin, E. (2014). Accurate viral population assembly from ultra-deep sequencing data. Bioinformatics *30*, i329–i337.

36. Abram, M.E., Ferris, A.L., Das, K., Quiñones, O., Shao, W., Tuske, S., Alvord, W.G., Arnold, E., and Hughes, S.H. (2014). Mutations in HIV-1 reverse transcriptase affect the errors made in a single cycle of viral replication. J. Virol. *88*, 7589–7601.

37. Gu, Z., Gao, Q., Li, X., Parniak, M.A., and Wainberg, M.A. (1992). Novel mutation in the human immunodeficiency virus type 1 reverse transcriptase gene that encodes cross-resistance to 2′,3′-dideoxyinosine and 2′,3′-dideoxycytidine. J. Virol. *66*, 7128–7135.

38. Pingen, M., Sarrami-Forooshani, R., Wensing, A.M., van Ham, P., Drewniak, A., Boucher, C.A., Geijtenbeek, T.B., and Nijhuis, M. (2014). Diminished transmission of drug resistant HIV-1 variants with reduced replication capacity in a human transmission model. Retrovirology *11*, 113.

39. Wrobel, J.A., Chao, S.F., Conrad, M.J., Merker, J.D., Swanstrom, R., Pielak, G.J., and Hutchison, C.A., 3rd (1998). A genetic approach for identifying critical residues in the fingers and palm subdomains of HIV-1 reverse transcriptase. Proc. Natl. Acad. Sci. USA *95*, 638–645.

40. Ssemwanga, D., Lyagoba, F., Ndembi, N., Mayanja, B.N., Larke, N., Wang, S., Baalwa, J., Williamson, C., Grosskurth, H., and Kaleebu, P. (2011). Multiple HIV-1 infections with evidence of recombination in heterosexual partnerships in a low risk rural clinical cohort in Uganda. Virology *411*, 113–131.

41. Kim, D.H., Behlke, M.A., Rose, S.D., Chang, M.S., Choi, S., and Rossi, J.J. (2005). Synthetic dsRNA Dicer substrates enhance RNAi potency and efficacy. Nat. Biotechnol. *23*, 222–226.

42. Carneiro, B., Braga, A.C.S., Batista, M.N., Harris, M., and Rahal, P. (2015). Evaluation of canonical siRNA and Dicer substrate RNA for inhibition of hepatitis C virus genome replication–a comparative study. PLoS ONE *10*, e0117742.

43. Wee, E.G., Ondondo, B., Berglund, P., Archer, J., McMichael, A.J., Baltimore, D., Ter Meulen, J.H., and Hanke, T. (2017). HIV-1 conserved mosaics delivered by regimens with integration-deficient DC-targeting lentiviral vector induce robust T cells. Mol. Ther. *25*, 494–503.

44. Keulen, W., Back, N.K., van Wijk, A., Boucher, C.A., and Berkhout, B. (1997). Initial appearance of the 184Ile variant in lamivudine-treated patients is caused by the mutational bias of human immunodeficiency virus type 1 reverse transcriptase. J. Virol. *71*, 3346–3350.

45. Back, N.K., Nijhuis, M., Keulen, W., Boucher, C.A., Oude Essink, B.O., van Kuilenburg, A.B., van Gennip, A.H., and Berkhout, B. (1996). Reduced replication of 3TC-resistant HIV-1 variants in primary cells due to a processivity defect of the reverse transcriptase enzyme. EMBO J. *15*, 4040–4049.

46. Tchurikov, N.A., and Kretova, O.V. (2007). *Suffix*-specific RNAi leads to silencing of F element in *Drosophila melanogaster*. PLoS ONE *2*, e476.

47. Tchurikov, N.A., and Kretova, O.V. (2011). Both piRNA and siRNA pathways are silencing transcripts of the *suffix* element in the *Drosophila melanogaster* germline and somatic cells. PLoS ONE *6*, e21882.

48. Leipzig, J. (2017). A review of bioinformatic pipeline frameworks. Brief. Bioinform. *18*, 530–536.

49. Posada-Cespedesa, S., Seifert, D., and Beerenwinkel, N. (2016). Recent advances in inferring viral diversity from high-throughput sequencing data. Virus Res., Published online September 28, 2016. http://dx.doi.org/10.1016/j.virusres.2016.09.016.

50. Andrews, S. (2010). FastQC: a quality control tool for high throughput sequence data. http://www.bioinformatics.babraham.ac.uk/projects/fastqc.

51. Martin, M. (2011). Cutadapt removes adapter sequences from high-throughput sequencing reads. EMBnet.journal. *17*, 10–12.

52. Langmead, B., and Salzberg, S.L. (2012). Fast gapped-read alignment with Bowtie 2. Nat. Methods *9*, 357–359.

53. Li, H., Handsaker, B., Wysoker, A., Fennell, T., Ruan, J., Homer, N., Marth, G., Abecasis, G., and Durbin, R.; 1000 Genome Project Data Processing Subgroup (2009). The sequence alignment/map (SAM) format and SAMtools. Bioinformatics *25*, 2078–2079.

54. Kent, W.J., Zweig, A.S., Barber, G., Hinrichs, A.S., and Karolchik, D. (2010). BigWig and BigBed: enabling browsing of large distributed datasets. Bioinformatics *26*, 2204–2207.

55. Katoh, K., and Standley, D.M. (2013). MAFFT multiple sequence alignment software version 7: improvements in performance and usability. Mol. Biol. Evol. *30*, 772–780.

56. Okonechnikov, K., Golosova, O., and Fursov, M.; UGENE team (2012). Unipro UGENE: a unified bioinformatics toolkit. Bioinformatics *28*, 1166–1167.

57. Johnson, N.L., and Lion, F.C. (1977). Statistics and Experimental Design in Engineering and the Physical Sciences, *Volume 1*, Second Edition (Wiley).

58. Weir, B.S. (1996). Genetic Data Analysis II: Methods for Discrete Population Genetic Data (Sinauer Associates).

OMTN, Volume *8*

Supplemental Information

Six Highly Conserved Targets of RNAi Revealed in HIV-1-Infected Patients from Russia Are Also Present in Many HIV-1 Strains Worldwide

Olga V. Kretova, Daria M. Fedoseeva, Maria A. Gorbacheva, Natalya M. Gashnikova, Maria P. Gashnikova, Nataliya V. Melnikova, Vladimir R. Chechetkin, Yuri V. Kravatsky, and Nickolai A. Tchurikov

Table S1. Percentage of 19-bp target invariability for different targets and cohorts.

	Cohort 1		Cohort 2	
**Target**	Aligned reads	Invariability, percent	Aligned reads	Invariability, percent
**A1**	1 291 242	99.17 ± 0.0080	8 390 187	97.71 ± 0.0052
**A2**	1 956 875	92.40 ± 0.0189	7 816 744	99.58 ± 0.0023
**A3**	1 418 446	96.84 ± 0.0147	4 862 688	96.68 ± 0.0081
**A4**	152 194	97.36 ± 0.0411	77 584	99.70 ± 0.0197
**A5**	951 233	97.14 ± 0.0171	8 181 119	96.57 ± 0.0064
**A6**	1 529 377	94.12 ± 0.0190	7 188 344	96.94 ± 0.0064

The invariability of 19-bp RNAi targets was assessed against the sets of aligned reads. The expected standard deviation was calculated according to Eq. (3) in the Materials and Methods section.

Table S2. Primers used for RT-PCR.

**№**	**Text of primer (5'-3')**	**Used for:**	**Domain**
**1**	TTTGATATGTCCATTGGTCTAGCCCTTGTT	Primer extension, (-) primer	RT-A1
**2**	CATCTATTGAGATGGGGATTTACCA	PCR-2, (+) primer	RT-A1
**3**	TGTTCTCTGCCAATTCCAATTCTG	PCR-2, (-) primer	RT-A1
**4**	CTCTGTTAGTGCTTTGGTCCCCCTAAGGAG	Primer extension, (-) primer	RT-A2
**5**	GACAAAGATCTTAGAGCCCTTTAGA	PCR-2, (+) primer	RT-A2
**6**	TATATCATTGACAGTCCAGCTTTCC	PCR-2, (-) primer	RT-A2
**7**	TTCTTGGTACTACCTTTATTTCGTTATTGTCTTG	Primer extension, (-) primer	Int-A3
**8**	AGAATTTGGAATTCCCTACAATCCC	PCR-2, (+) primer	Int-A3
**9**	TCTGCTGTCCCTGTAATAAACCC	PCR-2, (-) primer	Int-A3
**10**	CTAGAATCATTTCTTGTGGGTTGGGGTCTGTG	Primer extension, (-) primer	Vpu-A4
**11**	AGTAGGACTAATAGTAGCATTCATAG	PCR-2, (+) primer	Vpu-A4
**12**	TAATAGACTGTGACCCACAAGTTATTT	PCR-2, (-) primer	Vpu-A4
**13**	ACTACTGGCCTAATTCCATGTGTACATTGT	Primer extension, (-) primer	Gp120-A5
**14**	GACCCAACAACAATACAAGAAAAAGT	PCR-2, (+) primer	Gp120-A5
**15**	GTTGTATTGCAATAGAAAAATTCTCCT	PCR-2, (-) primer	Gp120-A5
**16**	TCCTAGGTGATATGGCCTGGTGTACCATTTG	Primer extension, (-) primer	P17-A6
**17**	TTGACTAGCGGAGGCTAGAAGG	PCR-2, (+) primer	P17-A6
**18**	ATTATGTAATGATTTAAGTTCTTCTGTT	PCR-2, (-) primer	P17-A6

Sequences were selected using the corresponding regions in AF316544 sequence. PCR primers are shown only for PCR-2.

Table S3. HIV BLAST results for target A1.

https://www.hiv.lanl.gov/content/sequence/BASIC_BLAST/basic_blast.html

HIV sequence database

HIV BLAST Results

BLAST Summary

200 sequences –13 different subtypes: A1, B, D, 02_AG, 02G, BF1, 01_AE, 08_BC, A1D, 35_AD, BC, 10_CD, C.

Query= seq1=A1 19 bp core sequence

Query	Acc	Description	Identity(%)
**seq1**	KY035111	HIV-1|name|seid 743558|-|US|2001|def	100
**seq1**	KY035251	HIV-1|name|seid 743418|-|US|2000|def	100
**seq1**	KY035957	HIV-1|name|seid 742712|-|US|2006|def	100
**seq1**	KY036939	HIV-1|name|seid 741730|-|US|2011|def	100
**seq1**	KY037212	HIV-1|name|seid 741457|-|US|2010|def	100
**seq1**	KY037546	HIV-1|name|seid 741123|-|US|2008|def	100
**seq1**	KY037705	HIV-1|name|seid 740964|-|US|2001|def	100
**seq1**	KX139396	HIV-1|name|seid 739853|-|NG|2012|def	100
**seq1**	KX944684	HIV-1|name|seid 739044|D|UG|2013|def	100
**seq1**	KX926891	HIV-1|name|seid 738677|B|US|2003|def	100
**seq1**	KX926901	HIV-1|name|seid 738667|B|US|2007|def	100
**seq1**	KX927025	HIV-1|name|seid 738543|B|US|2002|def	100
**seq1**	KY235817	HIV-1|name|seid 736976|-|UZ|2015|def	100
**seq1**	KY235854	HIV-1|name|seid 736939|A1|UZ|2015|def	100
**seq1**	KX790972	HIV-1|name|seid 728827|A1|KE|2012|def	100
**seq1**	KX791026	HIV-1|name|seid 728773|A1|KE|2012|def	100
**seq1**	KX661499	HIV-1|name|seid 728656|B|GB|2012|def	100
**seq1**	KX661727	HIV-1|name|seid 728428|B|GB|2010|def	100
**seq1**	KX662059	HIV-1|name|seid 728096|B|GB|2011|def	100

**seq1**	KX662388	HIV-1|name|seid 727767|A1|GB|2010|def	100
**seq1**	KX662716	HIV-1|name|seid 727439|B|GB|2008|def	100
**seq1**	KX887886	HIV-1|name|seid 725293|B|BR|2015|def	100
**seq1**	KX888007	HIV-1|name|seid 725172|B|BR|2015|def	100
**seq1**	KX888678	HIV-1|name|seid 724501|BF1|BR|2015|def	100
**seq1**	KX888825	HIV-1|name|seid 724354|B|BR|2015|def	100
**seq1**	KX782011	HIV-1|name|seid 721841|B|KR|2011|def	100
**seq1**	KU954716	HIV-1|name|seid 720993|BC|CN|2012|def	100
**seq1**	KX302419	HIV-1|name|seid 719272|A1|KE|2000|def	100
**seq1**	KX302561	HIV-1|name|seid 719236|A1|KE|2002|def	100
**seq1**	KU678028	HIV-1|name|seid 720075|B|US|2014|def	100
**seq1**	KU678034	HIV-1|name|seid 720069|B|US|2014|def	100
**seq1**	KT379823	HIV-1|name|seid 715951|B|CN|2008|def	100
**seq1**	KX018204	HIV-1|name|seid 714260|B|US|-|def	100
**seq1**	KX465662	HIV-1|name|seid 709717|B|DE|2007|def	100
**seq1**	KX466879	HIV-1|name|seid 708500|B|DE|2010|def	100
**seq1**	KX467092	HIV-1|name|seid 708287|02_AG|DE|2011|def	100
**seq1**	KU645853	HIV-1|name|seid 706221|A1|RU|2013|def	100
**seq1**	KU645876	HIV-1|name|seid 706198|A1|RU|2013|def	100
**seq1**	KU645878	HIV-1|name|seid 706196|A1|RU|2013|def	100
**seq1**	KU670329	HIV-1|name|seid 706182|A1|RU|2013|def	100
**seq1**	KX306421	HIV-1|name|seid 705386|A1|KE|2010|def	100
**seq1**	KX306437	HIV-1|name|seid 705370|A1|KE|2010|def	100
**seq1**	KU684966	HIV-1|name|seid 705032|02G|CM|2010|def	100
**seq1**	KJ474071	HIV-1|name|seid 701536|B|PA|2009|def	100
**seq1**	KJ474082	HIV-1|name|seid 701525|B|PA|2009|def	100
**seq1**	KJ474638	HIV-1|name|seid 700969|B|PA|2013|def	100
**seq1**	KT998288	HIV-1|name|seid	100

		700679|B|GF|2006|def	
**seq1**	KX128987	HIV-1|name|seid 698006|A1|-|-|def	100
**seq1**	KT713247	HIV-1|name|seid 693755|B|ES|1999|def	100
**seq1**	KR677204	HIV-1|name|seid 692773|02G|TG|2012|def	100
**seq1**	KU574443	HIV-1|name|seid 692197|B|AT|-|def	100
**seq1**	KU248505	HIV-1|name|seid 691230|A1|KE|2004|def	100
**seq1**	KU248535	HIV-1|name|seid 691200|A1|KE|2004|def	100
**seq1**	KU248537	HIV-1|name|seid 691198|A1|KE|2004|def	100
**seq1**	KU248573	HIV-1|name|seid 691162|A1|KE|2004|def	100
**seq1**	KU248656	HIV-1|name|seid 691079|A1|KE|2005|def	100
**seq1**	KU248678	HIV-1|name|seid 691057|D|KE|2005|def	100
**seq1**	KU248685	HIV-1|name|seid 691050|A1|KE|2005|def	100
**seq1**	KU248714	HIV-1|name|seid 691021|A1|KE|2005|def	100
**seq1**	KU644957	HIV-1|name|seid 690152|B|US|2000|def	100
**seq1**	KT863987	HIV-1|name|seid 688930|A1|BE|-|def	100
**seq1**	KT864319	HIV-1|name|seid 688598|02_AG|BE|-|def	100
**seq1**	KT869041	HIV-1|name|seid 688542|B|MX|2013|def	100
**seq1**	KT869070	HIV-1|name|seid 688513|B|MX|2014|def	100
**seq1**	KP877908	HIV-1|name|seid 683521|A1|KE|2011|def	100
**seq1**	KP877945	HIV-1|name|seid 683484|A1|KE|2011|def	100
**seq1**	KT347996	HIV-1|name|seid 685469|A1|UG|2010|def	100
**seq1**	KT348010	HIV-1|name|seid 685455|A1|UG|2010|def	100
**seq1**	KT348112	HIV-1|name|seid 685353|D|UG|2010|def	100
**seq1**	KT348170	HIV-1|name|seid 685295|D|UG|2010|def	100
**seq1**	KT348172	HIV-1|name|seid 685293|A1D|UG|2010|def	100
**seq1**	KU498429	HIV-1|name|seid 685128|A1|GB|2008|def	100
**seq1**	KU499327	HIV-1|name|seid 684230|D|GB|2008|def	100
**seq1**	KT427720	HIV-1|name|seid 676707|B|BR|2010|def	100

**seq1**	KJ396414	HIV-1|name|seid 673997|B|FR|2006|def	100
**seq1**	KT315997	HIV-1|name|seid 672788|A1|CD|2012|def	100
**seq1**	KT121452	HIV-1|name|seid 672226|A1|RU|2015|def	100
**seq1**	KT741420	HIV-1|name|seid 671822|B|BR|2009|def	100
**seq1**	KT746713	HIV-1|name|seid 666529|BF1|BR|2008|def	100
**seq1**	KT747846	HIV-1|name|seid 665396|BF1|BR|2009|def	100
**seq1**	KT748494	HIV-1|name|seid 664748|B|BR|2007|def	100
**seq1**	KT228902	HIV-1|name|seid 662873|A1|AU|2012|def	100
**seq1**	KT229271	HIV-1|name|seid 662504|B|AU|2004|def	100
**seq1**	KT370918	HIV-1|name|seid 660500|A1|KE|2009|def	100
**seq1**	KM985254	HIV-1|name|seid 658669|B|IL|2011|def	100
**seq1**	KR233334	HIV-1|name|seid 658545|A1|RU|2008|def	100
**seq1**	KR233337	HIV-1|name|seid 658542|A1|RU|2008|def	100
**seq1**	KT340116	HIV-1|name|seid 658309|B|PL|2014|def	100
**seq1**	KT340203	HIV-1|name|seid 658222|B|PL|2014|def	100
**seq1**	KM358093	HIV-1|name|seid 657667|B|US|2010|def	100
**seq1**	KT022364	HIV-1|name|seid 657563|A1|KE|2004|def	100
**seq1**	KF267601	HIV-1|name|seid 653027|01_AE|CN|2010|def	100
**seq1**	HG421598	HIV-1|name|seid 643451|08_BC|CN|2010|def	100
**seq1**	KP688157	HIV-1|name|seid 642702|B|CU|2008|def	100
**seq1**	KR188035	HIV-1|name|seid 641704|01_AE|CN|2013|def	100
**seq1**	KP738951	HIV-1|name|seid 640665|D|UG|2005|def	100
**seq1**	KP739008	HIV-1|name|seid 640608|D|UG|2005|def	100
**seq1**	KP739041	HIV-1|name|seid 640575|D|UG|2006|def	100
**seq1**	KP739061	HIV-1|name|seid 640555|D|UG|2006|def	100
**seq1**	KP739064	HIV-1|name|seid 640552|D|UG|2006|def	100
**seq1**	KP739159	HIV-1|name|seid 640457|D|UG|2005|def	100
**seq1**	KP739163	HIV-1|name|seid	100

		640453|D|UG|2005|def	
**seq1**	KP121155	HIV-1|name|seid 634664|BF1|TR|2014|def	100
**seq1**	KP121156	HIV-1|name|seid 634663|BF1|TR|2014|def	100
**seq1**	KP065817	HIV-1|name|seid 634520|B|US|2011|def	100
**seq1**	KP066341	HIV-1|name|seid 633996|B|US|2011|def	100
**seq1**	KP066658	HIV-1|name|seid 633679|B|US|2011|def	100
**seq1**	KP066826	HIV-1|name|seid 633511|B|US|2011|def	100
**seq1**	KP066871	HIV-1|name|seid 633466|B|US|2011|def	100
**seq1**	KP066888	HIV-1|name|seid 633449|B|US|2011|def	100
**seq1**	KP066892	HIV-1|name|seid 633445|B|US|2011|def	100
**seq1**	KP067002	HIV-1|name|seid 633335|B|US|2011|def	100
**seq1**	KP067016	HIV-1|name|seid 633321|B|US|2011|def	100
**seq1**	KP067017	HIV-1|name|seid 633320|B|US|2011|def	100
**seq1**	KP113072	HIV-1|name|seid 633238|B|US|2002|def	100
**seq1**	KP113084	HIV-1|name|seid 633226|B|US|2002|def	100
**seq1**	KP113095	HIV-1|name|seid 633215|B|US|2002|def	100
**seq1**	KP113101	HIV-1|name|seid 633209|B|US|2002|def	100
**seq1**	KP113362	HIV-1|name|seid 632948|B|US|2002|def	100
**seq1**	KP113371	HIV-1|name|seid 632939|B|US|2002|def	100
**seq1**	KP067025	HIV-1|name|seid 633312|B|US|2011|def	100
**seq1**	KP223814	HIV-1|name|seid 626395|A1|RW|2007|def	100
**seq1**	KP681733	HIV-1|name|seid 624614|A1D|TZ|-|def	100
**seq1**	KM851060	HIV-1|name|seid 621802|B|BR|2012|def	100
**seq1**	KF984060	HIV-1|name|seid 621504|C|IN|2012|def	100
**seq1**	KF544044	HIV-1|name|seid 621391|35_AD|IR|2011|def	100
**seq1**	KF544054	HIV-1|name|seid 621381|35_AD|IR|2011|def	100
**seq1**	KP235159	HIV-1|name|seid 620638|BC|CN|2009|def	100
**seq1**	KJ636003	HIV-1|name|seid 620524|A1|CY|2012|def	100

**seq1**	EU342764	HIV-1|name|seid 27683|10_CD|ES|2003|def	100
**seq1**	KF444838	HIV-1|name|seid 564447|-|GR|-|def	100
**seq1**	JX300509	HIV-1|name|seid 502888|B|HR|2006|def	100
**seq1**	JQ259171	HIV-1|name|seid 533260|01_AE|BG|2009|def	100
**seq1**	GQ268493	HIV-1|name|seid 370115|A1|GE|2007|def	100
**seq1**	JF929071	HIV-1|name|seid 443581|A1|ES|2007|def	100
**seq1**	GQ268440	HIV-1|name|seid 370168|A1|GE|2006|def	100
**seq1**	KC184386	HIV-1|name|seid 517927|A1|IL|2001|def	100
**seq1**	KP013647	HIV-1|name|seid 619050|B|SI|2013|def	100
**seq1**	KP013743	HIV-1|name|seid 618954|A1|SI|2000|def	100
**seq1**	KM395730	HIV-1|name|seid 617219|C|CN|2010|def	100
**seq1**	KM438266	HIV-1|name|seid 616652|A1D|TZ|2008|def	100
**seq1**	KM588946	HIV-1|name|seid 613836|01_AE|KW|2013|def	100
**seq1**	KM247296	HIV-1|name|seid 613632|A1|RU|1999|def	100
**seq1**	KM247299	HIV-1|name|seid 613629|A1|RU|1999|def	100
**seq1**	KJ870273	HIV-1|name|seid 613208|A1|RU|2010|def	100
**seq1**	KJ870346	HIV-1|name|seid 613135|A1|RU|2010|def	100
**seq1**	KJ870365	HIV-1|name|seid 613116|A1|RU|2011|def	100
**seq1**	KJ870435	HIV-1|name|seid 613046|A1|RU|2011|def	100
**seq1**	KJ870580	HIV-1|name|seid 612901|A1|RU|2012|def	100
**seq1**	KJ870603	HIV-1|name|seid 612878|A1|RU|2012|def	100
**seq1**	KJ870632	HIV-1|name|seid 612849|A1|RU|2013|def	100
**seq1**	KJ870677	HIV-1|name|seid 612804|A1|RU|2012|def	100
**seq1**	KJ870682	HIV-1|name|seid 612799|A1|RU|2013|def	100
**seq1**	KJ769488	HIV-1|name|seid 609954|B|US|2008|def	100
**seq1**	KF544096	HIV-1|name|seid 607114|A1|KE|2007|def	100
**seq1**	KF544137	HIV-1|name|seid 607073|A1|KE|2006|def	100
**seq1**	KF544200	HIV-1|name|seid	100

		607010|A1|KE|2006|def	
**seq1**	KJ769707	HIV-1|name|seid 606811|B|DE|2001|def	100
**seq1**	KJ769933	HIV-1|name|seid 606585|B|DE|2004|def	100
**seq1**	KJ770061	HIV-1|name|seid 606457|B|DE|2005|def	100
**seq1**	KJ770081	HIV-1|name|seid 606437|B|DE|2005|def	100
**seq1**	KJ770176	HIV-1|name|seid 606342|B|DE|2006|def	100
**seq1**	KJ770841	HIV-1|name|seid 605677|B|DE|2008|def	100
**seq1**	KJ771004	HIV-1|name|seid 605514|B|DE|2009|def	100
**seq1**	KJ771214	HIV-1|name|seid 605304|B|DE|2010|def	100
**seq1**	KJ771247	HIV-1|name|seid 605271|02_AG|DE|2010|def	100
**seq1**	KJ722815	HIV-1|name|seid 604627|B|US|2006|def	100
**seq1**	KJ722844	HIV-1|name|seid 604598|B|US|2001|def	100
**seq1**	KJ723386	HIV-1|name|seid 604056|B|US|2005|def	100
**seq1**	KJ722071	HIV-1|name|seid 603103|A1|RU|2011|def	100
**seq1**	KJ722084	HIV-1|name|seid 603090|A1|RU|2012|def	100
**seq1**	KJ906694	HIV-1|name|seid 601271|D|UG|2002|def	100
**seq1**	KJ906797	HIV-1|name|seid 601168|A1|UG|2006|def	100
**seq1**	KJ906853	HIV-1|name|seid 601112|A1|UG|2006|def	100
**seq1**	KJ906907	HIV-1|name|seid 601058|A1|UG|2007|def	100
**seq1**	KJ906911	HIV-1|name|seid 601054|A1|UG|2007|def	100
**seq1**	KJ907072	HIV-1|name|seid 600893|A1C|UG|2008|def	100
**seq1**	KJ907123	HIV-1|name|seid 600842|A1D|UG|2008|def	100
**seq1**	KJ907142	HIV-1|name|seid 600823|D|UG|2008|def	100
**seq1**	KJ502161	HIV-1|name|seid 597707|A1|KE|2013|def	100
**seq1**	KJ185225	HIV-1|name|seid 596817|C|IN|2012|def	100
**seq1**	KJ778897	HIV-1|name|seid 596143|0107|CN|2012|def	100
**seq1**	KC900666	HIV-1|name|seid 593064|A1|UG|2010|def	100
**seq1**	KC900683	HIV-1|name|seid 593047|A1|KE|2010|def	100

**seq1**	KC900692	HIV-1|name|seid 593038|A1|UG|2010|def	100
**seq1**	KC900699	HIV-1|name|seid 593031|A1C|KE|2009|def	100
**seq1**	KC900707	HIV-1|name|seid 593023|A1|UG|2010|def	100
**seq1**	KJ499570	HIV-1|name|seid 592525|A1|RU|2012|def	100
**seq1**	KF531125	HIV-1|name|seid 589772|A1|TZ|2005|def	100
**seq1**	KF531248	HIV-1|name|seid 589649|A1|TZ|2005|def	100
**seq1**	KF531275	HIV-1|name|seid 589622|A1|TZ|2005|def	100
**seq1**	KC681848	HIV-1|name|seid 592265|A1|RU|-|def	100
**seq1**	KF531361	HIV-1|name|seid 589536|A1|TZ|2005|def	100
**seq1**	KF531448	HIV-1|name|seid 589449|A1|TZ|2005|def	100
**seq1**	KF531484	HIV-1|name|seid 589413|A1|TZ|2005|def	100
**seq1**	KC560241	HIV-1|name|seid 587354|01_AE|TW|2012|def	100
**seq1**	KF135126	HIV-1|name|seid 587142|A1|IL|2009|def	100
**seq1**	KF720945	HIV-1|name|seid 583855|A1|AM|2009|def	100
**seq1**	KF576525	HIV-1|name|seid 582335|F2|CM|-|def	100
**seq1**	KF576588	HIV-1|name|seid 582272|A1G|CM|-|def	100

Table S4. HIV BLAST results for target A2.

https://www.hiv.lanl.gov/content/sequence/BASIC_BLAST/basic_blast.html

HIV sequence database

HIV BLAST Results

BLAST Summary

200 sequences – 16 different subtypes – D, B, A1, BF1, BC, 02_AG, 02G, A1D, 01AE, 35_AD, 10_CD, 01_AE , C, 0107, F2, A1G.

Query= seq1=A2 19 bp core sequence

Query	Acc	Description	Identity(%)
**seq1**	KY035111	HIV-1|name|seid 743558|-|US|2001|def	100

**seq1**	KY035251	HIV-1|name|seid 743418|-|US|2000|def	100
**seq1**	KY035957	HIV-1|name|seid 742712|-|US|2006|def	100
**seq1**	KY036939	HIV-1|name|seid 741730|-|US|2011|def	100
**seq1**	KY037212	HIV-1|name|seid 741457|-|US|2010|def	100
**seq1**	KY037546	HIV-1|name|seid 741123|-|US|2008|def	100
**seq1**	KY037705	HIV-1|name|seid 740964|-|US|2001|def	100
**seq1**	KX139396	HIV-1|name|seid 739853|-|NG|2012|def	100
**seq1**	KX944684	HIV-1|name|seid 739044|D|UG|2013|def	100
**seq1**	KX926891	HIV-1|name|seid 738677|B|US|2003|def	100
**seq1**	KX926901	HIV-1|name|seid 738667|B|US|2007|def	100
**seq1**	KX927025	HIV-1|name|seid 738543|B|US|2002|def	100
**seq1**	KY235817	HIV-1|name|seid 736976|-|UZ|2015|def	100
**seq1**	KY235854	HIV-1|name|seid 736939|A1|UZ|2015|def	100
**seq1**	KX790972	HIV-1|name|seid 728827|A1|KE|2012|def	100
**seq1**	KX791026	HIV-1|name|seid 728773|A1|KE|2012|def	100
**seq1**	KX661499	HIV-1|name|seid 728656|B|GB|2012|def	100
**seq1**	KX661727	HIV-1|name|seid 728428|B|GB|2010|def	100
**seq1**	KX662059	HIV-1|name|seid 728096|B|GB|2011|def	100
**seq1**	KX662388	HIV-1|name|seid 727767|A1|GB|2010|def	100
**seq1**	KX662716	HIV-1|name|seid 727439|B|GB|2008|def	100
**seq1**	KX887886	HIV-1|name|seid 725293|B|BR|2015|def	100
**seq1**	KX888007	HIV-1|name|seid 725172|B|BR|2015|def	100
**seq1**	KX888678	HIV-1|name|seid 724501|BF1|BR|2015|def	100
**seq1**	KX888825	HIV-1|name|seid 724354|B|BR|2015|def	100
**seq1**	KX782011	HIV-1|name|seid 721841|B|KR|2011|def	100
**seq1**	KU954716	HIV-1|name|seid 720993|BC|CN|2012|def	100
**seq1**	KX302419	HIV-1|name|seid 719272|A1|KE|2000|def	100
**seq1**	KX302561	HIV-1|name|seid	100

		719236|A1|KE|2002|def	
**seq1**	KU678028	HIV-1|name|seid 720075|B|US|2014|def	100
**seq1**	KU678034	HIV-1|name|seid 720069|B|US|2014|def	100
**seq1**	KT379823	HIV-1|name|seid 715951|B|CN|2008|def	100
**seq1**	KX018204	HIV-1|name|seid 714260|B|US|-|def	100
**seq1**	KX465662	HIV-1|name|seid 709717|B|DE|2007|def	100
**seq1**	KX466879	HIV-1|name|seid 708500|B|DE|2010|def	100
**seq1**	KX467092	HIV-1|name|seid 708287|02_AG|DE|2011|def	100
**seq1**	KU645853	HIV-1|name|seid 706221|A1|RU|2013|def	100
**seq1**	KU645876	HIV-1|name|seid 706198|A1|RU|2013|def	100
**seq1**	KU645878	HIV-1|name|seid 706196|A1|RU|2013|def	100
**seq1**	KU670329	HIV-1|name|seid 706182|A1|RU|2013|def	100
**seq1**	KX306421	HIV-1|name|seid 705386|A1|KE|2010|def	100
**seq1**	KX306437	HIV-1|name|seid 705370|A1|KE|2010|def	100
**seq1**	KU684966	HIV-1|name|seid 705032|02G|CM|2010|def	100
**seq1**	KJ474071	HIV-1|name|seid 701536|B|PA|2009|def	100
**seq1**	KJ474082	HIV-1|name|seid 701525|B|PA|2009|def	100
**seq1**	KJ474638	HIV-1|name|seid 700969|B|PA|2013|def	100
**seq1**	KT998288	HIV-1|name|seid 700679|B|GF|2006|def	100
**seq1**	KX128987	HIV-1|name|seid 698006|A1|-|-|def	100
**seq1**	KT713247	HIV-1|name|seid 693755|B|ES|1999|def	100
**seq1**	KR677204	HIV-1|name|seid 692773|02G|TG|2012|def	100
**seq1**	KU574443	HIV-1|name|seid 692197|B|AT|-|def	100
**seq1**	KU248505	HIV-1|name|seid 691230|A1|KE|2004|def	100
**seq1**	KU248535	HIV-1|name|seid 691200|A1|KE|2004|def	100
**seq1**	KU248537	HIV-1|name|seid 691198|A1|KE|2004|def	100
**seq1**	KU248573	HIV-1|name|seid 691162|A1|KE|2004|def	100
**seq1**	KU248656	HIV-1|name|seid 691079|A1|KE|2005|def	100

**seq1**	KU248678	HIV-1|name|seid 691057|D|KE|2005|def	100
**seq1**	KU248685	HIV-1|name|seid 691050|A1|KE|2005|def	100
**seq1**	KU248714	HIV-1|name|seid 691021|A1|KE|2005|def	100
**seq1**	KU644957	HIV-1|name|seid 690152|B|US|2000|def	100
**seq1**	KT863987	HIV-1|name|seid 688930|A1|BE|-|def	100
**seq1**	KT864319	HIV-1|name|seid 688598|02_AG|BE|-|def	100
**seq1**	KT869041	HIV-1|name|seid 688542|B|MX|2013|def	100
**seq1**	KT869070	HIV-1|name|seid 688513|B|MX|2014|def	100
**seq1**	KP877908	HIV-1|name|seid 683521|A1|KE|2011|def	100
**seq1**	KP877945	HIV-1|name|seid 683484|A1|KE|2011|def	100
**seq1**	KT347996	HIV-1|name|seid 685469|A1|UG|2010|def	100
**seq1**	KT348010	HIV-1|name|seid 685455|A1|UG|2010|def	100
**seq1**	KT348112	HIV-1|name|seid 685353|D|UG|2010|def	100
**seq1**	KT348170	HIV-1|name|seid 685295|D|UG|2010|def	100
**seq1**	KT348172	HIV-1|name|seid 685293|A1D|UG|2010|def	100
**seq1**	KU498429	HIV-1|name|seid 685128|A1|GB|2008|def	100
**seq1**	KU499327	HIV-1|name|seid 684230|D|GB|2008|def	100
**seq1**	KT427720	HIV-1|name|seid 676707|B|BR|2010|def	100
**seq1**	KJ396414	HIV-1|name|seid 673997|B|FR|2006|def	100
**seq1**	KT315997	HIV-1|name|seid 672788|A1|CD|2012|def	100
**seq1**	KT121452	HIV-1|name|seid 672226|A1|RU|2015|def	100
**seq1**	KT741420	HIV-1|name|seid 671822|B|BR|2009|def	100
**seq1**	KT746713	HIV-1|name|seid 666529|BF1|BR|2008|def	100
**seq1**	KT747846	HIV-1|name|seid 665396|BF1|BR|2009|def	100
**seq1**	KT748494	HIV-1|name|seid 664748|B|BR|2007|def	100
**seq1**	KT228902	HIV-1|name|seid 662873|A1|AU|2012|def	100
**seq1**	KT229271	HIV-1|name|seid 662504|B|AU|2004|def	100
**seq1**	KT370918	HIV-1|name|seid	100

		660500|A1|KE|2009|def	
**seq1**	KM985254	HIV-1|name|seid 658669|B|IL|2011|def	100
**seq1**	KR233334	HIV-1|name|seid 658545|A1|RU|2008|def	100
**seq1**	KR233337	HIV-1|name|seid 658542|A1|RU|2008|def	100
**seq1**	KT340116	HIV-1|name|seid 658309|B|PL|2014|def	100
**seq1**	KT340203	HIV-1|name|seid 658222|B|PL|2014|def	100
**seq1**	KM358093	HIV-1|name|seid 657667|B|US|2010|def	100
**seq1**	KT022364	HIV-1|name|seid 657563|A1|KE|2004|def	100
**seq1**	KF267601	HIV-1|name|seid 653027|01_AE|CN|2010|def	100
**seq1**	HG421598	HIV-1|name|seid 643451|08_BC|CN|2010|def	100
**seq1**	KP688157	HIV-1|name|seid 642702|B|CU|2008|def	100
**seq1**	KR188035	HIV-1|name|seid 641704|01_AE|CN|2013|def	100
**seq1**	KP738951	HIV-1|name|seid 640665|D|UG|2005|def	100
**seq1**	KP739008	HIV-1|name|seid 640608|D|UG|2005|def	100
**seq1**	KP739041	HIV-1|name|seid 640575|D|UG|2006|def	100
**seq1**	KP739061	HIV-1|name|seid 640555|D|UG|2006|def	100
**seq1**	KP739064	HIV-1|name|seid 640552|D|UG|2006|def	100
**seq1**	KP739159	HIV-1|name|seid 640457|D|UG|2005|def	100
**seq1**	KP739163	HIV-1|name|seid 640453|D|UG|2005|def	100
**seq1**	KP121155	HIV-1|name|seid 634664|BF1|TR|2014|def	100
**seq1**	KP121156	HIV-1|name|seid 634663|BF1|TR|2014|def	100
**seq1**	KP065817	HIV-1|name|seid 634520|B|US|2011|def	100
**seq1**	KP066341	HIV-1|name|seid 633996|B|US|2011|def	100
**seq1**	KP066658	HIV-1|name|seid 633679|B|US|2011|def	100
**seq1**	KP066826	HIV-1|name|seid 633511|B|US|2011|def	100
**seq1**	KP066871	HIV-1|name|seid 633466|B|US|2011|def	100
**seq1**	KP066888	HIV-1|name|seid 633449|B|US|2011|def	100
**seq1**	KP066892	HIV-1|name|seid 633445|B|US|2011|def	100

**seq1**	KP067002	HIV-1|name|seid 633335|B|US|2011|def	100
**seq1**	KP067016	HIV-1|name|seid 633321|B|US|2011|def	100
**seq1**	KP067017	HIV-1|name|seid 633320|B|US|2011|def	100
**seq1**	KP113072	HIV-1|name|seid 633238|B|US|2002|def	100
**seq1**	KP113084	HIV-1|name|seid 633226|B|US|2002|def	100
**seq1**	KP113095	HIV-1|name|seid 633215|B|US|2002|def	100
**seq1**	KP113101	HIV-1|name|seid 633209|B|US|2002|def	100
**seq1**	KP113362	HIV-1|name|seid 632948|B|US|2002|def	100
**seq1**	KP113371	HIV-1|name|seid 632939|B|US|2002|def	100
**seq1**	KP067025	HIV-1|name|seid 633312|B|US|2011|def	100
**seq1**	KP223814	HIV-1|name|seid 626395|A1|RW|2007|def	100
**seq1**	KP681733	HIV-1|name|seid 624614|A1D|TZ|-|def	100
**seq1**	KM851060	HIV-1|name|seid 621802|B|BR|2012|def	100
**seq1**	KF984060	HIV-1|name|seid 621504|C|IN|2012|def	100
**seq1**	KF544044	HIV-1|name|seid 621391|35_AD|IR|2011|def	100
**seq1**	KF544054	HIV-1|name|seid 621381|35_AD|IR|2011|def	100
**seq1**	KP235159	HIV-1|name|seid 620638|BC|CN|2009|def	100
**seq1**	KJ636003	HIV-1|name|seid 620524|A1|CY|2012|def	100
**seq1**	EU342764	HIV-1|name|seid 27683|10_CD|ES|2003|def	100
**seq1**	KF444838	HIV-1|name|seid 564447|-|GR|-|def	100
**seq1**	JX300509	HIV-1|name|seid 502888|B|HR|2006|def	100
**seq1**	JQ259171	HIV-1|name|seid 533260|01_AE|BG|2009|def	100
**seq1**	GQ268493	HIV-1|name|seid 370115|A1|GE|2007|def	100
**seq1**	JF929071	HIV-1|name|seid 443581|A1|ES|2007|def	100
**seq1**	GQ268440	HIV-1|name|seid 370168|A1|GE|2006|def	100
**seq1**	KC184386	HIV-1|name|seid 517927|A1|IL|2001|def	100
**seq1**	KP013647	HIV-1|name|seid 619050|B|SI|2013|def	100
**seq1**	KP013743	HIV-1|name|seid	100

		618954|A1|SI|2000|def	
**seq1**	KM395730	HIV-1|name|seid<br>617219|C|CN|2010|def	100
**seq1**	KM438266	HIV-1|name|seid<br>616652|A1D|TZ|2008|def	100
**seq1**	KM588946	HIV-1|name|seid<br>613836|01_AE|KW|2013|def	100
**seq1**	KM247296	HIV-1|name|seid<br>613632|A1|RU|1999|def	100
**seq1**	KM247299	HIV-1|name|seid<br>613629|A1|RU|1999|def	100
**seq1**	KJ870273	HIV-1|name|seid<br>613208|A1|RU|2010|def	100
**seq1**	KJ870346	HIV-1|name|seid<br>613135|A1|RU|2010|def	100
**seq1**	KJ870365	HIV-1|name|seid<br>613116|A1|RU|2011|def	100
**seq1**	KJ870435	HIV-1|name|seid<br>613046|A1|RU|2011|def	100
**seq1**	KJ870580	HIV-1|name|seid<br>612901|A1|RU|2012|def	100
**seq1**	KJ870603	HIV-1|name|seid<br>612878|A1|RU|2012|def	100
**seq1**	KJ870632	HIV-1|name|seid<br>612849|A1|RU|2013|def	100
**seq1**	KJ870677	HIV-1|name|seid<br>612804|A1|RU|2012|def	100
**seq1**	KJ870682	HIV-1|name|seid<br>612799|A1|RU|2013|def	100
**seq1**	KJ769488	HIV-1|name|seid<br>609954|B|US|2008|def	100
**seq1**	KF544096	HIV-1|name|seid<br>607114|A1|KE|2007|def	100
**seq1**	KF544137	HIV-1|name|seid<br>607073|A1|KE|2006|def	100
**seq1**	KF544200	HIV-1|name|seid<br>607010|A1|KE|2006|def	100
**seq1**	KJ769707	HIV-1|name|seid<br>606811|B|DE|2001|def	100
**seq1**	KJ769933	HIV-1|name|seid<br>606585|B|DE|2004|def	100
**seq1**	KJ770061	HIV-1|name|seid<br>606457|B|DE|2005|def	100
**seq1**	KJ770081	HIV-1|name|seid<br>606437|B|DE|2005|def	100
**seq1**	KJ770176	HIV-1|name|seid<br>606342|B|DE|2006|def	100
**seq1**	KJ770841	HIV-1|name|seid<br>605677|B|DE|2008|def	100
**seq1**	KJ771004	HIV-1|name|seid<br>605514|B|DE|2009|def	100
**seq1**	KJ771214	HIV-1|name|seid<br>605304|B|DE|2010|def	100
**seq1**	KJ771247	HIV-1|name|seid<br>605271|02_AG|DE|2010|def	100

**seq1**	KJ722815	HIV-1|name|seid 604627|B|US|2006|def	100
**seq1**	KJ722844	HIV-1|name|seid 604598|B|US|2001|def	100
**seq1**	KJ723386	HIV-1|name|seid 604056|B|US|2005|def	100
**seq1**	KJ722071	HIV-1|name|seid 603103|A1|RU|2011|def	100
**seq1**	KJ722084	HIV-1|name|seid 603090|A1|RU|2012|def	100
**seq1**	KJ906694	HIV-1|name|seid 601271|D|UG|2002|def	100
**seq1**	KJ906797	HIV-1|name|seid 601168|A1|UG|2006|def	100
**seq1**	KJ906853	HIV-1|name|seid 601112|A1|UG|2006|def	100
**seq1**	KJ906907	HIV-1|name|seid 601058|A1|UG|2007|def	100
**seq1**	KJ906911	HIV-1|name|seid 601054|A1|UG|2007|def	100
**seq1**	KJ907072	HIV-1|name|seid 600893|A1C|UG|2008|def	100
**seq1**	KJ907123	HIV-1|name|seid 600842|A1D|UG|2008|def	100
**seq1**	KJ907142	HIV-1|name|seid 600823|D|UG|2008|def	100
**seq1**	KJ502161	HIV-1|name|seid 597707|A1|KE|2013|def	100
**seq1**	KJ185225	HIV-1|name|seid 596817|C|IN|2012|def	100
**seq1**	KJ778897	HIV-1|name|seid 596143|0107|CN|2012|def	100
**seq1**	KC900666	HIV-1|name|seid 593064|A1|UG|2010|def	100
**seq1**	KC900683	HIV-1|name|seid 593047|A1|KE|2010|def	100
**seq1**	KC900692	HIV-1|name|seid 593038|A1|UG|2010|def	100
**seq1**	KC900699	HIV-1|name|seid 593031|A1C|KE|2009|def	100
**seq1**	KC900707	HIV-1|name|seid 593023|A1|UG|2010|def	100
**seq1**	KJ499570	HIV-1|name|seid 592525|A1|RU|2012|def	100
**seq1**	KF531125	HIV-1|name|seid 589772|A1|TZ|2005|def	100
**seq1**	KF531248	HIV-1|name|seid 589649|A1|TZ|2005|def	100
**seq1**	KF531275	HIV-1|name|seid 589622|A1|TZ|2005|def	100
**seq1**	KC681848	HIV-1|name|seid 592265|A1|RU|-|def	100
**seq1**	KF531361	HIV-1|name|seid 589536|A1|TZ|2005|def	100
**seq1**	KF531448	HIV-1|name|seid	100

		589449|A1|TZ|2005|def	
**seq1**	KF531484	HIV-1|name|seid 589413|A1|TZ|2005|def	100
**seq1**	KC560241	HIV-1|name|seid 587354|01_AE|TW|2012|def	100
**seq1**	KF135126	HIV-1|name|seid 587142|A1|IL|2009|def	100
**seq1**	KF720945	HIV-1|name|seid 583855|A1|AM|2009|def	100
**seq1**	KF576525	HIV-1|name|seid 582335|F2|CM|-|def	100
**seq1**	KF576588	HIV-1|name|seid 582272|A1G|CM|-|def	100

Table S5. HIV BLAST results for target A3.

https://www.hiv.lanl.gov/content/sequence/BASIC_BLAST/basic_blast.html

HIV sequence database

HIV BLAST Results

BLAST Summary

200 sequences – 12 different subtypes A1, 63_02A1, 63A1, D, 01_AE, BG, 02_AG, 14_BG, G, U, 37_cpx, A1BC, and SIV.
Query= seq1=A3 19 bp core sequence

Query	Acc	Description	Identity(%)
**seq1**	KU749400	HIV-1|name|seid 739023|A1|UA|2011|def	100
**seq1**	KX574417	HIV-1|name|seid 723997|63_02A1|RU|2015|def	100
**seq1**	KX574448	HIV-1|name|seid 723966|63A1|RU|2015|def	100
**seq1**	KX640210	HIV-1|name|seid 722783|A1|RU|2015|def	100
**seq1**	KX640233	HIV-1|name|seid 722760|A1|RU|2015|def	100
**seq1**	KF716491	HIV-1|name|seid 561754|A1|RU|2008|def	100
**seq1**	KJ415771	HIV-1|name|seid 593225|A1|AM|2009|def	100
**seq1**	AB870417	HIV-1|name|seid 571650|A1|JP|2012|def	100
**seq1**	HQ709849	HIV-1|name|seid 411685|A1|UG|2009|def	100
**seq1**	HQ709981	HIV-1|name|seid 411553|A1|UG|2009|def	100
**seq1**	HQ709994	HIV-1|name|seid 411540|A1|UG|2009|def	100
**seq1**	HQ710071	HIV-1|name|seid	100

		411463|A1|UG|2008|def	
**seq1**	HQ710187	HIV-1|name|seid 411347|D|UG|2008|def	100
**seq1**	AB253680	HIV-1|name|seid 102579|01_AE|JP|-|def	100
**seq1**	U57602	SIV|name|seid 238125|SAB|SN|1991|def	100
**seq1**	AB253651	HIV-1|name|seid 102608|01_AE|JP|-|def	100
**seq1**	AB253647	HIV-1|name|seid 102612|01_AE|JP|-|def	100
**seq1**	EF589042	HIV-1|name|seid 65712|A1|KZ|2002|def	100
**seq1**	EF589043	HIV-1|name|seid 65711|A1|KZ|2002|def	100
**seq1**	EU074774	HIV-1|name|seid 42123|BG|ES|2002|def	100
**seq1**	KX574407	HIV-1|name|seid 724007|63_02A1|RU|2015|def	100
**seq1**	KX574445	HIV-1|name|seid 723969|63_02A1|RU|2015|def	100
**seq1**	KC215175	HIV-1|name|seid 544754|A1|KZ|2009|def	100
**seq1**	JX202786	HIV-1|name|seid 522750|A1|UG|2007|def	100
**seq1**	HQ709985	HIV-1|name|seid 411549|A1|UG|2009|def	100
**seq1**	HQ710182	HIV-1|name|seid 411352|D|UG|2008|def	100
**seq1**	HM466988	HIV-1|name|seid 368941|A1|RU|2008|def	100
**seq1**	AF423755	HIV-1|name|seid 89445|BG|ES|1999|def	100
**seq1**	AF458242	HIV-1|name|seid 149881|A1|RW|1992|def	100
**seq1**	EU618551	HIV-1|name|seid 263944|02_AG|CM|1996|def	100
**seq1**	EU618518	HIV-1|name|seid 263977|02_AG|CM|1996|def	100
**seq1**	FJ670517	HIV-1|name|seid 333749|14_BG|ES|2004|def	100
**seq1**	GU217258	HIV-1|name|seid 336815|A1|GB|-|def	100
**seq1**	GU217004	HIV-1|name|seid 337069|A1|ES|2006|def	100
**seq1**	KY235915	HIV-1|name|seid 736878|A1|UZ|2013|def	100
**seq1**	KX574426	HIV-1|name|seid 723988|63_02A1|RU|2015|def	100
**seq1**	KX640200	HIV-1|name|seid 722793|A1|RU|2015|def	100
**seq1**	KX640217	HIV-1|name|seid 722776|A1|RU|2015|def	100
**seq1**	KC312920	HIV-1|name|seid 531946|A1|RU|2012|def	100

**seq1**	JX236669	HIV-1|name|seid 494843|A1|UG|2007|def	100
**seq1**	AF423759	HIV-1|name|seid 89441|14_BG|ES|2000|def	100
**seq1**	AY612637	HIV-1|name|seid 88145|G|PT|-|def	100
**seq1**	AB253421	HIV-1|name|seid 102981|A1|RW|1992|def	100
**seq1**	AB253673	HIV-1|name|seid 102586|01_AE|JP|-|def	100
**seq1**	AB253657	HIV-1|name|seid 102602|01_AE|JP|-|def	100
**seq1**	FJ465107	HIV-1|name|seid 258252|G|RU|2005|def	100
**seq1**	FJ388932	HIV-1|name|seid 283159|A1|CY|2005|def	100
**seq1**	GU362884	HIV-1|name|seid 368148|G|ES|2009|def	100
**seq1**	FJ670518	HIV-1|name|seid 333761|14_BG|ES|2004|def	100
**seq1**	FJ670527	HIV-1|name|seid 333754|DF1G|ES|2004|def	100
**seq1**	GU216925	HIV-1|name|seid 337148|G|ES|2005|def	100
**seq1**	DQ823356	HIV-1|name|seid 104698|A1|UA|2001|def	100
**seq1**	KU749402	HIV-1|name|seid 739021|A1|UA|2012|def	100
**seq1**	KX640229	HIV-1|name|seid 722764|A1|RU|2015|def	100
**seq1**	KX640235	HIV-1|name|seid 722758|A1|RU|2015|def	100
**seq1**	KJ415752	HIV-1|name|seid 593244|A1|AM|2009|def	100
**seq1**	KJ415754	HIV-1|name|seid 593242|A1|AM|2009|def	100
**seq1**	KF612672	HIV-1|name|seid 578014|A1|RU|2012|def	100
**seq1**	AB870695	HIV-1|name|seid 571368|B|JP|2011|def	100
**seq1**	KF716500	HIV-1|name|seid 561745|A1|UG|2003|def	100
**seq1**	JX202790	HIV-1|name|seid 522748|A1|UG|2007|def	100
**seq1**	JQ821358	HIV-1|name|seid 484432|A1|KG|2009|def	100
**seq1**	JQ292900	HIV-1|name|seid 462829|A1|RU|2006|def	100
**seq1**	HM466994	HIV-1|name|seid 368935|A1|UA|2008|def	100
**seq1**	AF413970	HIV-1|name|seid 142101|A1|UA|2000|def	100
**seq1**	AB253674	HIV-1|name|seid 102585|01_AE|JP|-|def	100
**seq1**	FJ465122	HIV-1|name|seid	100

		258237|G|RU|2005|def	
**seq1**	FJ388950	HIV-1|name|seid 283141|A1|CY|2006|def	100
**seq1**	FJ388951	HIV-1|name|seid 283140|A1|CY|2006|def	100
**seq1**	AY829203	HIV-1|name|seid 114155|A1|UZ|2002|def	100
**seq1**	DQ823358	HIV-1|name|seid 104696|A1|UA|2001|def	100
**seq1**	EU074780	HIV-1|name|seid 42117|BG|ES|2005|def	100
**seq1**	KY235909	HIV-1|name|seid 736884|A1|UZ|2013|def	100
**seq1**	KX574449	HIV-1|name|seid 723965|63A1|RU|2015|def	100
**seq1**	KJ197201	HIV-1|name|seid 610471|63_02A1|RU|2013|def	100
**seq1**	KJ415770	HIV-1|name|seid 593226|A1|AM|2009|def	100
**seq1**	KF612693	HIV-1|name|seid 577993|A1|RU|2011|def	100
**seq1**	KF648734	HIV-1|name|seid 564908|A1|KG|2010|def	100
**seq1**	KC215172	HIV-1|name|seid 544757|02_AG|KZ|2009|def	100
**seq1**	HQ710075	HIV-1|name|seid 411459|A1|UG|2008|def	100
**seq1**	HM215250	HIV-1|name|seid 398693|U|CA|2001|def	100
**seq1**	HM466985	HIV-1|name|seid 368944|A1|RU|2008|def	100
**seq1**	AF413972	HIV-1|name|seid 142099|A1|UA|2000|def	100
**seq1**	AB253677	HIV-1|name|seid 102582|01_AE|JP|-|def	100
**seq1**	AF450098	HIV-1|name|seid 89446|G|ES|1999|def	100
**seq1**	FJ465099	HIV-1|name|seid 258260|G|RU|2005|def	100
**seq1**	FJ670522	HIV-1|name|seid 333758|14_BG|ES|2005|def	100
**seq1**	GU217042	HIV-1|name|seid 337031|G|ES|2007|def	100
**seq1**	FJ183543	HIV-1|name|seid 2785|A1|DE|2007|def	100
**seq1**	KX574421	HIV-1|name|seid 723993|63_02A1|RU|2015|def	100
**seq1**	KX574440	HIV-1|name|seid 723974|63_02A1|RU|2015|def	100
**seq1**	KX574452	HIV-1|name|seid 723962|63A1|RU|2015|def	100
**seq1**	KX640198	HIV-1|name|seid 722795|A1|RU|2015|def	100
**seq1**	KX228801	HIV-1|name|seid 713516|G|ES|2009|def	100

**seq1**	KJ415756	HIV-1|name|seid 593240|A1|AM|2009|def	100
**seq1**	KJ415761	HIV-1|name|seid 593235|A1|AM|2009|def	100
**seq1**	KJ415767	HIV-1|name|seid 593229|A1|AM|2009|def	100
**seq1**	KJ415777	HIV-1|name|seid 593219|A1|AM|2009|def	100
**seq1**	KC681871	HIV-1|name|seid 592242|A1|RU|-|def	100
**seq1**	KF612661	HIV-1|name|seid 578025|A1|RU|2012|def	100
**seq1**	JX425832	HIV-1|name|seid 500733|A1G|FR|2008|def	100
**seq1**	JQ846266	HIV-1|name|seid 486199|A1|KG|2009|def	100
**seq1**	HQ714910	HIV-1|name|seid 415467|02_AG|CM|-|def	100
**seq1**	AY829211	HIV-1|name|seid 114147|A1|UZ|2002|def	100
**seq1**	FJ388892	HIV-1|name|seid 283199|A1|CY|2005|def	100
**seq1**	FJ465126	HIV-1|name|seid 258233|G|RU|2005|def	100
**seq1**	KU749399	HIV-1|name|seid 739024|A1|UA|2011|def	100
**seq1**	KU749404	HIV-1|name|seid 739019|A1|UA|2012|def	100
**seq1**	KX574406	HIV-1|name|seid 724008|63_02A1|RU|2015|def	100
**seq1**	KX640236	HIV-1|name|seid 722757|A1|RU|2015|def	100
**seq1**	AB870674	HIV-1|name|seid 571389|B|JP|2011|def	100
**seq1**	HM467005	HIV-1|name|seid 368924|A1|RU|2009|def	100
**seq1**	DQ823366	HIV-1|name|seid 104691|A1|UA|2001|def	100
**seq1**	FJ465119	HIV-1|name|seid 258240|G|RU|2005|def	100
**seq1**	FJ465105	HIV-1|name|seid 258254|G|RU|2005|def	100
**seq1**	EU618547	HIV-1|name|seid 263948|G|CM|1996|def	100
**seq1**	KY235916	HIV-1|name|seid 736877|A1|UZ|2013|def	100
**seq1**	KF747732	HIV-1|name|seid 614004|A1|RU|-|def	100
**seq1**	KF612690	HIV-1|name|seid 577996|A1|RU|2011|def	100
**seq1**	KC473837	HIV-1|name|seid 527351|14_BG|ES|2005|def	100
**seq1**	JX202785	HIV-1|name|seid 523017|A1|UG|2007|def	100
**seq1**	JX202795	HIV-1|name|seid	100

		523012|A1|UG|2007|def	
**seq1**	JX451923	HIV-1|name|seid 510265|14_BG|CH|2008|def	100
**seq1**	HQ709996	HIV-1|name|seid 411538|A1|UG|2009|def	100
**seq1**	HQ710069	HIV-1|name|seid 411465|A1|UG|2008|def	100
**seq1**	DQ460696	HIV-1|name|seid 116033|A2|KE|-|def	100
**seq1**	AB253422	HIV-1|name|seid 102980|A1|RW|1992|def	100
**seq1**	AB253671	HIV-1|name|seid 102588|01_AE|JP|-|def	100
**seq1**	AF457080	HIV-1|name|seid 189746|A1|KE|2000|def	100
**seq1**	FJ473401	HIV-1|name|seid 258430|G|RU|2005|def	100
**seq1**	FJ388903	HIV-1|name|seid 283188|A1|CY|2005|def	100
**seq1**	GU358383	HIV-1|name|seid 333723|G|FR|2009|def	100
**seq1**	DQ823360	HIV-1|name|seid 104694|A1|UA|2001|def	100
**seq1**	KY235920	HIV-1|name|seid 736873|-|UZ|2013|def	100
**seq1**	KX574418	HIV-1|name|seid 723996|63_02A1|RU|2015|def	100
**seq1**	KX574422	HIV-1|name|seid 723992|63_02A1|RU|2015|def	100
**seq1**	KX574430	HIV-1|name|seid 723984|63_02A1|RU|2015|def	100
**seq1**	KX640239	HIV-1|name|seid 722754|A1|RU|2015|def	100
**seq1**	KX640249	HIV-1|name|seid 722744|A1|RU|2015|def	100
**seq1**	KJ415732	HIV-1|name|seid 593264|A1|AM|2009|def	100
**seq1**	KJ415742	HIV-1|name|seid 593254|A1|AM|2009|def	100
**seq1**	KC681877	HIV-1|name|seid 592236|A1|RU|-|def	100
**seq1**	KF612669	HIV-1|name|seid 578017|A1|RU|2012|def	100
**seq1**	FJ183641	HIV-1|name|seid 2687|A1|DE|2007|def	100
**seq1**	HQ714911	HIV-1|name|seid 415466|02_AG|CM|-|def	100
**seq1**	HQ709993	HIV-1|name|seid 411541|A1|UG|2009|def	100
**seq1**	HM467012	HIV-1|name|seid 368917|A1|RU|2009|def	100
**seq1**	GU230137	HIV-1|name|seid 371432|14_BG|PT|2000|def	100
**seq1**	AF450096	HIV-1|name|seid 89448|14_BG|ES|2000|def	100

**seq1**	AB253676	HIV-1|name|seid 102583|01_AE|JP|-|def	100
**seq1**	AB253655	HIV-1|name|seid 102604|01_AE|JP|-|def	100
**seq1**	FV536612	synthetic DNA|name|seid 343931|-|-|-|def	100
**seq1**	FJ480285	HIV-1|name|seid 281559|G|CM|1996|def	100
**seq1**	GU216919	HIV-1|name|seid 337154|G|ES|2005|def	100
**seq1**	EU074775	HIV-1|name|seid 42122|BG|ES|2002|def	100
**seq1**	FJ388907	HIV-1|name|seid 283184|A1|CY|2005|def	100
**seq1**	EF589040	HIV-1|name|seid 65714|A1|KZ|2002|def	100
**seq1**	KX574432	HIV-1|name|seid 723982|63_02A1|RU|2015|def	100
**seq1**	KT022379	HIV-1|name|seid 657548|G|KE|2006|def	100
**seq1**	KJ415736	HIV-1|name|seid 593260|A1|AM|2009|def	100
**seq1**	KC681878	HIV-1|name|seid 592235|A1|RU|-|def	100
**seq1**	KC681865	HIV-1|name|seid 592248|A1|RU|-|def	100
**seq1**	AB869898	HIV-1|name|seid 572172|02_AG|JP|2012|def	100
**seq1**	KC215174	HIV-1|name|seid 544755|A1|KZ|2009|def	100
**seq1**	KC312894	HIV-1|name|seid 531959|A1|RU|2012|def	100
**seq1**	JX500699	HIV-1|name|seid 510190|63_02A1|RU|2011|def	100
**seq1**	JX425446	HIV-1|name|seid 500926|14_BG|FR|2008|def	100
**seq1**	JQ250602	HIV-1|name|seid 462122|02_AG|FR|2007|def	100
**seq1**	HQ714921	HIV-1|name|seid 415456|37_cpx|CM|-|def	100
**seq1**	HQ710073	HIV-1|name|seid 411461|A1|UG|2008|def	100
**seq1**	JF683780	HIV-1|name|seid 400209|A1|CY|2008|def	100
**seq1**	HM466979	HIV-1|name|seid 368950|A1|RU|2008|def	100
**seq1**	FJ465104	HIV-1|name|seid 258255|G|RU|2005|def	100
**seq1**	KU749405	HIV-1|name|seid 739018|A1|UA|2012|def	100
**seq1**	KX574415	HIV-1|name|seid 723999|63_02A1|RU|2015|def	100
**seq1**	KX574442	HIV-1|name|seid 723972|63_02A1|RU|2015|def	100
**seq1**	KF747731	HIV-1|name|seid	100

		614005|A1|RU|-|def	
**seq1**	KF747733	HIV-1|name|seid 614003|A1|RU|-|def	100
**seq1**	KJ415731	HIV-1|name|seid 593265|A1|AM|2009|def	100
**seq1**	KJ415764	HIV-1|name|seid 593232|A1|AM|2009|def	100
**seq1**	JQ292892	HIV-1|name|seid 462833|A1|RU|2002|def	100
**seq1**	HQ710078	HIV-1|name|seid 411456|A1|UG|2008|def	100
**seq1**	HQ710184	HIV-1|name|seid 411350|D|UG|2008|def	100
**seq1**	AB253678	HIV-1|name|seid 102581|01_AE|JP|-|def	100
**seq1**	DQ460695	HIV-1|name|seid 116034|A2|KE|-|def	100
**seq1**	AY829208	HIV-1|name|seid 114150|A1|UZ|2002|def	100
**seq1**	AF457077	HIV-1|name|seid 189749|A1|KE|2000|def	100
**seq1**	EU618229	HIV-1|name|seid 264266|G|CM|2002|def	100
**seq1**	EU618079	HIV-1|name|seid 264416|02_AG|CM|2003|def	100
**seq1**	KX640192	HIV-1|name|seid 722801|A1|RU|2015|def	100
**seq1**	KJ415768	HIV-1|name|seid 593228|A1|AM|2009|def	100
**seq1**	KC681875	HIV-1|name|seid 592238|A1|RU|-|def	100
**seq1**	AB869602	HIV-1|name|seid 572468|A1|JP|2010|def	100
**seq1**	KF716477	HIV-1|name|seid 561768|G|KE|2009|def	100
**seq1**	FR846409	HIV-1|name|seid 501646|G|PT|-|def	100
**seq1**	FJ183545	HIV-1|name|seid 2783|A1|DE|2006|def	100
**seq1**	HQ710183	HIV-1|name|seid 411351|D|UG|2008|def	100
**seq1**	HQ710189	HIV-1|name|seid 411345|D|UG|2008|def	100
**seq1**	AY857145	HIV-1|name|seid 79539|A1BC|AU|-|def	100
**seq1**	AF423756	HIV-1|name|seid 89444|14_BG|ES|1999|def	100

Table S6. HIV BLAST results for target A4.

https://www.hiv.lanl.gov/content/sequence/BASIC_BLAST/basic_blast.html

HIV sequence database

HIV BLAST Results

BLAST Summary

200 sequences – 11 different subtypes – A1, A1D, 01_AE, 35_AD, C, A1CD, 01A1, B, 11_cpx, 67_01B , 01B.

Query= seq1=A4 19 bp core sequence

Query	Acc	Description	Identity(%)
**seq1**	**KU749400**	**HIV-1|name|seid 739023|A1|UA|2011|def**	**100**
**seq1**	KT022364	HIV-1|name|seid 657563|A1|KE|2004|def	100
**seq1**	KT022399	HIV-1|name|seid 657528|A1D|KE|2005|def	100
**seq1**	KP223805	HIV-1|name|seid 626404|A1|RW|2007|def	100
**seq1**	JX203053	HIV-1|name|seid 522883|A1|UG|2007|def	100
**seq1**	JX203060	HIV-1|name|seid 522613|A1|UG|2007|def	100
**seq1**	JX447239	HIV-1|name|seid 498578|01_AE|TH|2009|def	100
**seq1**	AB703608	HIV-1|name|seid 496762|35_AD|IR|2010|def	100
**seq1**	HM027823	HIV-1|name|seid 363845|A1|UG|2007|def	100
**seq1**	GQ477442	HIV-1|name|seid 350590|35_AD|AF|2006|def	100
**seq1**	FJ623482	HIV-1|name|seid 336170|A1|KE|2006|def	100
**seq1**	FJ185243	HIV-1|name|seid 297884|01_AE|VN|1997|def	100
**seq1**	EU875277	HIV-1|name|seid 7122|A1|KE|1995|def	100
**seq1**	EU875216	HIV-1|name|seid 7183|A1|KE|2002|def	100
**seq1**	EU836515	HIV-1|name|seid 8283|A1|KE|2002|def	100
**seq1**	EU836462	HIV-1|name|seid 8336|A1|KE|1999|def	100
**seq1**	EU836448	HIV-1|name|seid 8350|A1|KE|1995|def	100
**seq1**	EU836335	HIV-1|name|seid 8463|A1|KE|1987|def	100
**seq1**	EU836339	HIV-1|name|seid 8459|A1|KE|1987|def	100
**seq1**	EU875237	HIV-1|name|seid 7162|A1|KE|2002|def	100
**seq1**	EF589042	HIV-1|name|seid 65712|A1|KZ|2002|def	100
**seq1**	KT012691	HIV-1|name|seid 653643|A1|IN|2012|def	100

**seq1**	KT152841	HIV-1|name|seid 643407|A1|IN|1999|def	100
**seq1**	KP223802	HIV-1|name|seid 626407|A1|RW|2007|def	100
**seq1**	KC681881	HIV-1|name|seid 592232|A1|RU|-|def	100
**seq1**	JX203056	HIV-1|name|seid 522615|A1|UG|2007|def	100
**seq1**	JX447073	HIV-1|name|seid 498661|01_AE|TH|2008|def	100
**seq1**	JN860764	HIV-1|name|seid 481249|01_AE|TH|2007|def	100
**seq1**	HM027871	HIV-1|name|seid 363797|A1D|UG|2003|def	100
**seq1**	AF069671	HIV-1|name|seid 223867|A1|SE|1994|def	100
**seq1**	FJ623483	HIV-1|name|seid 336169|A1|KE|2006|def	100
**seq1**	FJ185235	HIV-1|name|seid 297892|01_AE|VN|1998|def	100
**seq1**	AY358044	HIV-1|name|seid 163486|01_AE|TH|1999|def	100
**seq1**	EU875346	HIV-1|name|seid 7053|A1|KE|2002|def	100
**seq1**	EU875276	HIV-1|name|seid 7123|A1|KE|1995|def	100
**seq1**	EU875186	HIV-1|name|seid 7213|C|KE|1997|def	100
**seq1**	EU875108	HIV-1|name|seid 7252|A1|KE|1988|def	100
**seq1**	EU836329	HIV-1|name|seid 8469|A1|KE|1990|def	100
**seq1**	EU836357	HIV-1|name|seid 8441|A1|KE|1987|def	100
**seq1**	EF158040	HIV-1|name|seid 66098|35_AD|AF|2005|def	100
**seq1**	KJ190269	HIV-1|name|seid 585838|A1|RW|2007|def	100
**seq1**	KJ190273	HIV-1|name|seid 585834|A1|RW|2007|def	100
**seq1**	KF716470	HIV-1|name|seid 561775|A1CD|KE|2010|def	100
**seq1**	JX112800	HIV-1|name|seid 553671|01_AE|CN|2010|def	100
**seq1**	JX203210	HIV-1|name|seid 522754|A1|UG|2007|def	100
**seq1**	JX203052	HIV-1|name|seid 522617|A1|UG|2007|def	100
**seq1**	JX447056	HIV-1|name|seid 499595|01_AE|TH|2007|def	100
**seq1**	JX447076	HIV-1|name|seid 499585|01_AE|TH|2008|def	100
**seq1**	JX447311	HIV-1|name|seid 498542|01_AE|TH|2006|def	100
**seq1**	JX447285	HIV-1|name|seid	100

		498555|01_AE|TH|2007|def	
**seq1**	JN248355	HIV-1|name|seid 484374|01_AE|TH|2005|def	100
**seq1**	JF430899	HIV-1|name|seid 461794|A1|RU|2008|def	100
**seq1**	HQ616083	HIV-1|name|seid 456299|A1|RU|2008|def	100
**seq1**	HM027853	HIV-1|name|seid 363815|A1D|UG|2000|def	100
**seq1**	HM027877	HIV-1|name|seid 363791|A1D|UG|2003|def	100
**seq1**	AB253421	HIV-1|name|seid 102981|A1|RW|1992|def	100
**seq1**	AY905588	HIV-1|name|seid 122494|A1|KE|1987|def	100
**seq1**	AF457073	HIV-1|name|seid 189753|A1D|KE|1999|def	100
**seq1**	AF143901	HIV-1|name|seid 224230|A|TW|-|def	100
**seq1**	AF457070	HIV-1|name|seid 189756|A1|KE|2000|def	100
**seq1**	FJ623484	HIV-1|name|seid 336168|A1|KE|2006|def	100
**seq1**	FJ388953	HIV-1|name|seid 283138|01A1|CY|2006|def	100
**seq1**	FJ388932	HIV-1|name|seid 283159|A1|CY|2005|def	100
**seq1**	FJ866115	HIV-1|name|seid 300537|A1|KE|2005|def	100
**seq1**	FV536588	synthetic DNA|name|seid 343955|-|-|-|def	100
**seq1**	EU875305	HIV-1|name|seid 7094|A1D|KE|1996|def	100
**seq1**	EU875218	HIV-1|name|seid 7181|A1|KE|2002|def	100
**seq1**	EU836479	HIV-1|name|seid 8319|A1|KE|1997|def	100
**seq1**	EU836411	HIV-1|name|seid 8387|A1|KE|1996|def	100
**seq1**	DQ823356	HIV-1|name|seid 104698|A1|UA|2001|def	100
**seq1**	KM217988	HIV-1|name|seid 635602|01_AE|CN|2007|def	100
**seq1**	KP223849	HIV-1|name|seid 626360|A1|RW|2007|def	100
**seq1**	KC913687	HIV-1|name|seid 625159|01C|MM|2009|def	100
**seq1**	KJ190274	HIV-1|name|seid 585833|A1|RW|2007|def	100
**seq1**	KF716499	HIV-1|name|seid 561746|A1|RW|2003|def	100
**seq1**	JX203059	HIV-1|name|seid 522880|A1|UG|2007|def	100
**seq1**	JX203216	HIV-1|name|seid 522751|A1|UG|2007|def	100

**seq1**	JX203050	HIV-1|name|seid 522618|A1|UG|2007|def	100
**seq1**	JX203054	HIV-1|name|seid 522616|A1|UG|2007|def	100
**seq1**	JX203142	HIV-1|name|seid 522572|A1|UG|2007|def	100
**seq1**	JX447082	HIV-1|name|seid 499582|01_AE|TH|2005|def	100
**seq1**	JX447728	HIV-1|name|seid 499259|01_AE|TH|2009|def	100
**seq1**	JF430896	HIV-1|name|seid 461791|A1|RU|2008|def	100
**seq1**	HM027872	HIV-1|name|seid 363796|A1D|UG|2003|def	100
**seq1**	AF457086	HIV-1|name|seid 189740|A1|KE|2000|def	100
**seq1**	FJ388951	HIV-1|name|seid 283140|A1|CY|2006|def	100
**seq1**	FJ185250	HIV-1|name|seid 297877|01_AE|VN|1997|def	100
**seq1**	FJ866123	HIV-1|name|seid 300529|A1|KE|2005|def	100
**seq1**	AB485632	HIV-1|name|seid 312775|A1|UG|-|def	100
**seq1**	GU367592	HIV-1|name|seid 342455|B|US|-|def	100
**seq1**	EU875353	HIV-1|name|seid 7046|A1|KE|2002|def	100
**seq1**	EU875211	HIV-1|name|seid 7188|A1|KE|2002|def	100
**seq1**	EU836521	HIV-1|name|seid 8277|A1|KE|2002|def	100
**seq1**	EU836483	HIV-1|name|seid 8315|A1|KE|1995|def	100
**seq1**	EU836422	HIV-1|name|seid 8376|A1|KE|2000|def	100
**seq1**	EU836570	HIV-1|name|seid 9310|A1D|KE|1996|def	100
**seq1**	AY829203	HIV-1|name|seid 114155|A1|UZ|2002|def	100
**seq1**	DQ823358	HIV-1|name|seid 104696|A1|UA|2001|def	100
**seq1**	KP223801	HIV-1|name|seid 626408|A1|RW|2007|def	100
**seq1**	KP223822	HIV-1|name|seid 626387|A1|RW|2007|def	100
**seq1**	KP223840	HIV-1|name|seid 626369|A1|RW|2007|def	100
**seq1**	KP223848	HIV-1|name|seid 626361|A1|RW|2007|def	100
**seq1**	KC913647	HIV-1|name|seid 625199|01_AE|MM|2008|def	100
**seq1**	JX203057	HIV-1|name|seid 522881|A1|UG|2007|def	100
**seq1**	AF069670	HIV-1|name|seid	100

		223868|A1|SE|1994|def	
**seq1**	FJ623476	HIV-1|name|seid 336176|A1|KE|2006|def	100
**seq1**	EU875345	HIV-1|name|seid 7054|A1|KE|2002|def	100
**seq1**	EU875297	HIV-1|name|seid 7102|A1D|KE|1996|def	100
**seq1**	EU875298	HIV-1|name|seid 7101|A1D|KE|1996|def	100
**seq1**	EU836480	HIV-1|name|seid 8318|A1|KE|1994|def	100
**seq1**	EU836453	HIV-1|name|seid 8345|A1|KE|2002|def	100
**seq1**	EU836376	HIV-1|name|seid 8422|A1|KE|1995|def	100
**seq1**	EU110085	HIV-1|name|seid 14870|A1|KE|2001|def	100
**seq1**	KY042007	HIV-1|name|seid 720849|35_AD|IR|2012|def	100
**seq1**	KP109490	HIV-1|name|seid 626344|A1|UG|2009|def	100
**seq1**	JX447061	HIV-1|name|seid 498667|01_AE|TH|2007|def	100
**seq1**	HQ616082	HIV-1|name|seid 456290|A1|RU|-|def	100
**seq1**	AF413973	HIV-1|name|seid 142098|A1|UA|2000|def	100
**seq1**	AY829211	HIV-1|name|seid 114147|A1|UZ|2002|def	100
**seq1**	FJ388892	HIV-1|name|seid 283199|A1|CY|2005|def	100
**seq1**	AF127555	HIV-1|name|seid 211622|11_cpx|CM|1995|def	100
**seq1**	M62320	HIV-1|name|seid 252812|A1|UG|1985|def	100
**seq1**	FJ388925	HIV-1|name|seid 283166|A1|CY|2005|def	100
**seq1**	DQ083238	HIV-1|name|seid 119770|A1C|IN|2001|def	100
**seq1**	EU875352	HIV-1|name|seid 7047|A1|KE|2002|def	100
**seq1**	EU875300	HIV-1|name|seid 7099|A1D|KE|1996|def	100
**seq1**	EU836539	HIV-1|name|seid 8259|A1|KE|2004|def	100
**seq1**	EU836519	HIV-1|name|seid 8279|A1|KE|2002|def	100
**seq1**	EU836451	HIV-1|name|seid 8347|A1|KE|1995|def	100
**seq1**	EU836555	HIV-1|name|seid 8243|A1|KE|1989|def	100
**seq1**	KU749399	HIV-1|name|seid 739024|A1|UA|2011|def	100
**seq1**	KU749404	HIV-1|name|seid 739019|A1|UA|2012|def	100

**seq1**	KT022383	HIV-1|name|seid 657544|A1|KE|2006|def	100
**seq1**	KT152839	HIV-1|name|seid 643409|A1|IN|2009|def	100
**seq1**	KM217993	HIV-1|name|seid 635597|01_AE|CN|2007|def	100
**seq1**	KP223777	HIV-1|name|seid 626417|A1|RW|2007|def	100
**seq1**	KP223804	HIV-1|name|seid 626405|A1|RW|2007|def	100
**seq1**	KP223842	HIV-1|name|seid 626367|A1|RW|2007|def	100
**seq1**	KC913646	HIV-1|name|seid 625200|01_AE|MM|2008|def	100
**seq1**	JX203058	HIV-1|name|seid 522614|A1|UG|2007|def	100
**seq1**	JX203145	HIV-1|name|seid 522571|A1|UG|2007|def	100
**seq1**	JX447756	HIV-1|name|seid 499245|01_AE|TH|2007|def	100
**seq1**	JX448028	HIV-1|name|seid 499109|01_AE|TH|2008|def	100
**seq1**	JX447133	HIV-1|name|seid 498631|01_AE|TH|2006|def	100
**seq1**	HQ616093	HIV-1|name|seid 456295|A1|RU|2008|def	100
**seq1**	JF683779	HIV-1|name|seid 400210|A1|CY|2008|def	100
**seq1**	AB253428	HIV-1|name|seid 102974|A1|UG|1992|def	100
**seq1**	DQ823366	HIV-1|name|seid 104691|A1|UA|2001|def	100
**seq1**	AY945731	HIV-1|name|seid 106213|01_AE|TH|1999|def	100
**seq1**	AY284977	HIV-1|name|seid 110961|A|AR|1998|def	100
**seq1**	AY734554	HIV-1|name|seid 118541|A1C|TZ|2002|def	100
**seq1**	AF457087	HIV-1|name|seid 189739|A1C|KE|2000|def	100
**seq1**	AB098333	HIV-1|name|seid 176667|A1|UG|1992|def	100
**seq1**	FJ623485	HIV-1|name|seid 336167|A1|KE|2006|def	100
**seq1**	FJ623486	HIV-1|name|seid 336166|A1|KE|2006|def	100
**seq1**	FJ623477	HIV-1|name|seid 336175|A1|KE|2006|def	100
**seq1**	FJ185238	HIV-1|name|seid 297889|01_AE|VN|1997|def	100
**seq1**	EU875363	HIV-1|name|seid 7036|A1|KE|2002|def	100
**seq1**	EU875354	HIV-1|name|seid 7045|A1|KE|2002|def	100
**seq1**	EU875293	HIV-1|name|seid	100

		7106|A1D|KE|1996|def	
**seq1**	EU875240	HIV-1|name|seid 7159|A1|KE|2002|def	100
**seq1**	EU875235	HIV-1|name|seid 7164|A1|KE|2002|def	100
**seq1**	EU875212	HIV-1|name|seid 7187|A1|KE|2002|def	100
**seq1**	EU836527	HIV-1|name|seid 8271|A1|KE|2002|def	100
**seq1**	EU836345	HIV-1|name|seid 8453|A1D|KE|1987|def	100
**seq1**	EU875306	HIV-1|name|seid 7093|A1D|KE|1996|def	100
**seq1**	AF484512	HIV-1|name|seid 151375|A1|UG|1998|def	100
**seq1**	KU958484	SHIV|name|seid 697863|A1|-|-|def	100
**seq1**	KJ190263	HIV-1|name|seid 585844|A1|RW|2007|def	100
**seq1**	KJ190268	HIV-1|name|seid 585839|A1|RW|2007|def	100
**seq1**	KC183780	HIV-1|name|seid 527328|67_01B|CN|2011|def	100
**seq1**	JX203204	HIV-1|name|seid 522757|A1|UG|2007|def	100
**seq1**	JX203214	HIV-1|name|seid 522752|A1|UG|2007|def	100
**seq1**	JX447754	HIV-1|name|seid 499246|01_AE|TH|2007|def	100
**seq1**	JX448018	HIV-1|name|seid 499114|01B|TH|2005|def	100
**seq1**	JX447055	HIV-1|name|seid 498670|01_AE|TH|2007|def	100
**seq1**	JF683761	HIV-1|name|seid 400228|A1|CY|2007|def	100
**seq1**	HQ691074	HIV-1|name|seid 376832|01_AE|TH|2007|def	100
**seq1**	HQ691078	HIV-1|name|seid 376828|01_AE|TH|2007|def	100
**seq1**	HQ691079	HIV-1|name|seid 376827|01_AE|TH|2007|def	100
**seq1**	AF457052	HIV-1|name|seid 189774|A1|KE|2000|def	100
**seq1**	AB253422	HIV-1|name|seid 102980|A1|RW|1992|def	100
**seq1**	AY905603	HIV-1|name|seid 122479|A1D|KE|1987|def	100
**seq1**	AF457080	HIV-1|name|seid 189746|A1|KE|2000|def	100
**seq1**	FJ388903	HIV-1|name|seid 283188|A1|CY|2005|def	100
**seq1**	FJ647148	HIV-1|name|seid 282689|A1|ZA|2001|def	100
**seq1**	EU875349	HIV-1|name|seid 7050|A1|KE|2002|def	100

**seq1**	EU875351	HIV-1|name|seid 7048|A1|KE|2002|def	100
**seq1**	EU836424	HIV-1|name|seid 8374|A1|KE|1996|def	100
**seq1**	EU836338	HIV-1|name|seid 8460|A1|KE|1987|def	100
**seq1**	DQ823360	HIV-1|name|seid 104694|A1|UA|2001|def	100
**seq1**	EU836394	HIV-1|name|seid 8404|A1|KE|1996|def	100
**seq1**	EU875109	HIV-1|name|seid 7251|A1|KE|1988|def	100
**seq1**	DQ912822	HIV-1|name|seid 39195|A1D|DK|1996|def	100
**seq1**	KP223800	HIV-1|name|seid 626409|A1|RW|2007|def	100
**seq1**	KP223836	HIV-1|name|seid 626373|A1|RW|2007|def	100
**seq1**	KC913633	HIV-1|name|seid 625213|01_AE|MM|2008|def	100
**seq1**	JX203209	HIV-1|name|seid 523021|A1|UG|2007|def	100
**seq1**	JX203206	HIV-1|name|seid 522756|A1|UG|2007|def	100
**seq1**	JX203134	HIV-1|name|seid 522576|A1|UG|2007|def	100

Table S7. HIV BLAST results for target A6.

https://www.hiv.lanl.gov/content/sequence/BASIC_BLAST/basic_blast.html

HIV sequence database

HIV BLAST Results

BLAST Summary

200 sequences – 20 different subtypes - A1, A1D, C, B, G, 01_AE, F1, A1CD, A1C, 69_01B, 70_BF1, 58_01B, 56_cpx, 35_AD, 65_cpx, 01B, 01BC, BF1, 28_BF, 29_BF.

Query= seq1=A6 19 bp core sequence

**Query**	**Acc**	**Description**	**Identity(%)**
**seq1**	KU749400	HIV-1|name|seid 739023|A1|UA|2011|def	100
**seq1**	KU749413	HIV-1|name|seid 739010|-|PK|2014|def	100
**seq1**	KU921867	HIV-1|name|seid 721374|A1|KE|-|def	100
**seq1**	KU921873	HIV-1|name|seid	100

		721368|A1D|KE|-|def	
**seq1**	KU921923	HIV-1|name|seid 721318|A1CD|KE|-|def	100
**seq1**	KU921958	HIV-1|name|seid 721283|A1|KE|-|def	100
**seq1**	KU921991	HIV-1|name|seid 721250|A1|KE|-|def	100
**seq1**	KU922009	HIV-1|name|seid 721232|A1|KE|-|def	100
**seq1**	KU922059	HIV-1|name|seid 721182|C|KE|-|def	100
**seq1**	KU678028	HIV-1|name|seid 720075|B|US|2014|def	100
**seq1**	KU678034	HIV-1|name|seid 720069|B|US|2014|def	100
**seq1**	KX505399	HIV-1|name|seid 715720|B|US|2014|def	100
**seq1**	KX505580	HIV-1|name|seid 715539|B|US|2015|def	100
**seq1**	KU142283	HIV-1|name|seid 707613|B|GB|2007|def	100
**seq1**	KU142287	HIV-1|name|seid 707609|B|GB|2006|def	100
**seq1**	KU142301	HIV-1|name|seid 707595|B|GB|2005|def	100
**seq1**	KU142371	HIV-1|name|seid 707525|B|GB|2007|def	100
**seq1**	KU142448	HIV-1|name|seid 707448|B|GB|2006|def	100
**seq1**	KU142520	HIV-1|name|seid 707376|B|GB|2006|def	100
**seq1**	KU501257	HIV-1|name|seid 703551|01BC|CN|2013|def	100
**seq1**	KT124748	HIV-1|name|seid 703412|B|US|2001|def	100
**seq1**	KT124780	HIV-1|name|seid 703380|B|DE|2010|def	100
**seq1**	KT124787	HIV-1|name|seid 703373|B|DE|2012|def	100
**seq1**	KU685583	HIV-1|name|seid 693262|B|ES|2014|def	100
**seq1**	KT736703	HIV-1|name|seid 688254|C|ZA|2006|def	100
**seq1**	KT123093	HIV-1|name|seid 686827|G|PT|2007|def	100
**seq1**	KR811243	HIV-1|name|seid 684134|01_AE|CN|-|def	100
**seq1**	KR811265	HIV-1|name|seid 684112|01_AE|CN|-|def	100
**seq1**	KR811279	HIV-1|name|seid 684098|01_AE|CN|-|def	100
**seq1**	KU141042	HIV-1|name|seid 683733|B|US|-|def	100
**seq1**	KU168264	HIV-1|name|seid 685623|01_AE|-|2001|def	100

**seq1**	KT427659	HIV-1|name|seid 676768|B|BR|2010|def	100
**seq1**	KT427663	HIV-1|name|seid 676764|F1|BR|2010|def	100
**seq1**	KT427713	HIV-1|name|seid 676714|B|BR|2010|def	100
**seq1**	KT427720	HIV-1|name|seid 676707|B|BR|2010|def	100
**seq1**	KT427735	HIV-1|name|seid 676692|B|BR|2010|def	100
**seq1**	KT427760	HIV-1|name|seid 676667|BC|BR|2010|def	100
**seq1**	KT427789	HIV-1|name|seid 676638|BF1|BR|2010|def	100
**seq1**	KT339951	HIV-1|name|seid 674377|B|US|2008|def	100
**seq1**	KT339954	HIV-1|name|seid 674374|B|US|2005|def	100
**seq1**	LC100171	HIV-1|name|seid 662405|01_AE|VN|-|def	100
**seq1**	LC100194	HIV-1|name|seid 662382|01_AE|VN|-|def	100
**seq1**	LC100370	HIV-1|name|seid 662206|01_AE|VN|-|def	100
**seq1**	LC100442	HIV-1|name|seid 662134|01_AE|VN|-|def	100
**seq1**	LC100497	HIV-1|name|seid 662079|01_AE|VN|-|def	100
**seq1**	KT022364	HIV-1|name|seid 657563|A1|KE|2004|def	100
**seq1**	KT022399	HIV-1|name|seid 657528|A1D|KE|2005|def	100
**seq1**	KR020074	HIV-1|name|seid 656623|A1|KE|2008|def	100
**seq1**	KT191818	HIV-1|name|seid 656234|B|CN|2009|def	100
**seq1**	KT183246	HIV-1|name|seid 650893|C|ZA|2008|def	100
**seq1**	KT183260	HIV-1|name|seid 650879|C|ZA|2008|def	100
**seq1**	KT200355	HIV-1|name|seid 650790|B|ES|2010|def	100
**seq1**	KT200349	HIV-1|name|seid 650796|B|ES|2008|def	100
**seq1**	KR781619	HIV-1|name|seid 645081|A1|KE|-|def	100
**seq1**	KR781652	HIV-1|name|seid 645048|D|KE|1995|def	100
**seq1**	KR781722	HIV-1|name|seid 644978|A1|KE|1995|def	100
**seq1**	KR781738	HIV-1|name|seid 644962|A1|KE|1997|def	100
**seq1**	KR781778	HIV-1|name|seid 644922|D|KE|1994|def	100
**seq1**	KR781900	HIV-1|name|seid	100

		644800|A1|KE|2002|def	
**seq1**	KR781901	HIV-1|name|seid 644799|A1|KE|1995|def	100
**seq1**	KR781938	HIV-1|name|seid 644762|A1C|KE|1996|def	100
**seq1**	KR781973	HIV-1|name|seid 644727|A1D|KE|1997|def	100
**seq1**	KR782137	HIV-1|name|seid 644563|A1|KE|1995|def	100
**seq1**	KP223729	HIV-1|name|seid 626465|A1|RW|2007|def	100
**seq1**	KP223742	HIV-1|name|seid 626452|A1|RW|2007|def	100
**seq1**	KP223814	HIV-1|name|seid 626395|A1|RW|2007|def	100
**seq1**	KM192420	HIV-1|name|seid 626251|C|ZA|2008|def	100
**seq1**	KM192485	HIV-1|name|seid 626186|C|ZA|2008|def	100
**seq1**	KM192488	HIV-1|name|seid 626183|C|ZA|2008|def	100
**seq1**	KM192611	HIV-1|name|seid 626060|C|ZA|2009|def	100
**seq1**	KM192792	HIV-1|name|seid 625879|A1C|ZA|2010|def	100
**seq1**	KM192815	HIV-1|name|seid 625856|C|ZA|2010|def	100
**seq1**	KP056202	HIV-1|name|seid 625341|C|ZA|2008|def	100
**seq1**	LC027100	HIV-1|name|seid 625223|69_01B|JP|2005|def	100
**seq1**	KP208184	HIV-1|name|seid 624183|C|ZA|2012|def	100
**seq1**	KM484714	HIV-1|name|seid 617237|01_AE|CN|2013|def	100
**seq1**	KJ869481	HIV-1|name|seid 615984|B|DE|2004|def	100
**seq1**	KM243681	HIV-1|name|seid 615367|A1|IR|2012|def	100
**seq1**	KF818791	HIV-1|name|seid 613346|01_AE|CN|2010|def	100
**seq1**	KC913948	HIV-1|name|seid 611552|01_AE|MM|2008|def	100
**seq1**	KF701649	HIV-1|name|seid 608047|B|US|1985|def	100
**seq1**	KF701661	HIV-1|name|seid 608035|B|US|1985|def	100
**seq1**	KF701686	HIV-1|name|seid 608010|B|US|1985|def	100
**seq1**	KF701742	HIV-1|name|seid 607954|B|US|1987|def	100
**seq1**	KF701803	HIV-1|name|seid 607893|B|US|1988|def	100
**seq1**	KF701810	HIV-1|name|seid 607886|B|US|1989|def	100

**seq1**	KF701843	HIV-1|name|seid 607853|B|US|1988|def	100
**seq1**	KF701891	HIV-1|name|seid 607805|B|US|1984|def	100
**seq1**	KJ849801	HIV-1|name|seid 607375|B|BR|2010|def	100
**seq1**	KJ849809	HIV-1|name|seid 607367|70_BF1|BR|2010|def	100
**seq1**	KJ669905	HIV-1|name|seid 603539|B|NL|1991|def	100
**seq1**	KM048486	HIV-1|name|seid 600518|C|ZM|2008|def	100
**seq1**	KM048555	HIV-1|name|seid 600449|C|ZM|2006|def	100
**seq1**	KJ778897	HIV-1|name|seid 596143|0107|CN|2012|def	100
**seq1**	KF905959	HIV-1|name|seid 589068|C|ZA|2008|def	100
**seq1**	KF905968	HIV-1|name|seid 589059|C|ZA|2008|def	100
**seq1**	KF425293	HIV-1|name|seid 586799|58_01B|MY|2010|def	100
**seq1**	KJ190262	HIV-1|name|seid 585845|A1|RW|2007|def	100
**seq1**	AB873386	HIV-1|name|seid 573976|B|JP|-|def	100
**seq1**	AB873529	HIV-1|name|seid 573836|B|JP|-|def	100
**seq1**	AB873545	HIV-1|name|seid 573820|B|JP|-|def	100
**seq1**	KC852174	HIV-1|name|seid 564448|56_cpx|FR|2010|def	100
**seq1**	KF716480	HIV-1|name|seid 561765|D|UG|2011|def	100
**seq1**	KF526178	HIV-1|name|seid 561588|B|US|2011|def	100
**seq1**	KF208785	HIV-1|name|seid 559393|C|ZA|2010|def	100
**seq1**	JX112796	HIV-1|name|seid 553675|01_AE|CN|2010|def	100
**seq1**	JX112816	HIV-1|name|seid 553655|01_AE|CN|2007|def	100
**seq1**	KC797225	HIV-1|name|seid 552856|B|CH|2008|def	100
**seq1**	JX112856	HIV-1|name|seid 553615|01_AE|CN|2007|def	100
**seq1**	KC899333	HIV-1|name|seid 551375|B|FR|2012|def	100
**seq1**	KC149452	HIV-1|name|seid 543293|C|MW|2007|def	100
**seq1**	JQ302435	HIV-1|name|seid 542833|01_AE|CN|2009|def	100
**seq1**	JQ302372	HIV-1|name|seid 542642|01_AE|CN|2009|def	100
**seq1**	AB716197	HIV-1|name|seid	100

		539509|35_AD|IR|2010|def	
**seq1**	KC312514	HIV-1|name|seid 537345|B|US|2003|def	100
**seq1**	KC312532	HIV-1|name|seid 537336|B|US|2003|def	100
**seq1**	JX973394	HIV-1|name|seid 536646|B|US|2008|def	100
**seq1**	JX973448	HIV-1|name|seid 536619|B|US|2008|def	100
**seq1**	JX973480	HIV-1|name|seid 536603|B|US|2009|def	100
**seq1**	JX973492	HIV-1|name|seid 536597|B|US|2008|def	100
**seq1**	JX974044	HIV-1|name|seid 536321|C|ZA|2007|def	100
**seq1**	JX974114	HIV-1|name|seid 536286|C|ZA|2007|def	100
**seq1**	JX973347	HIV-1|name|seid 535614|B|US|2009|def	100
**seq1**	JX973433	HIV-1|name|seid 535571|B|US|2008|def	100
**seq1**	JX973493	HIV-1|name|seid 535541|B|US|2008|def	100
**seq1**	KC149259	HIV-1|name|seid 533919|C|MW|2008|def	100
**seq1**	HW060663	-|name|seid 533907|-|-|-|def	100
**seq1**	KC596063	HIV-1|name|seid 527345|01_AE|CN|2010|def	100
**seq1**	KC183778	HIV-1|name|seid 527329|65_cpx|CN|2011|def	100
**seq1**	JX203155	HIV-1|name|seid 523048|A1|UG|2007|def	100
**seq1**	JX202859	HIV-1|name|seid 522980|D|UG|2007|def	100
**seq1**	JX203156	HIV-1|name|seid 522781|A1|UG|2007|def	100
**seq1**	JX202904	HIV-1|name|seid 522691|D|UG|2007|def	100
**seq1**	JX202942	HIV-1|name|seid 522672|A1|UG|2007|def	100
**seq1**	JX202944	HIV-1|name|seid 522671|A1|UG|2007|def	100
**seq1**	JX446989	HIV-1|name|seid 498703|01_AE|TH|2007|def	100
**seq1**	JQ900864	HIV-1|name|seid 517273|01_AE|CN|2010|def	100
**seq1**	JQ900871	HIV-1|name|seid 517143|01_AE|CN|2009|def	100
**seq1**	JQ900891	HIV-1|name|seid 517133|B|CN|2008|def	100
**seq1**	JX960631	HIV-1|name|seid 510146|01_AE|CN|2009|def	100
**seq1**	JX960598	HIV-1|name|seid 510184|B|CN|2009|def	100
**seq1**	JX446646	HIV-1|name|seid	100

		499800|01_AE|TH|2008|def	
**seq1**	JX446850	HIV-1|name|seid 499698|01_AE|TH|2008|def	100
**seq1**	JX446852	HIV-1|name|seid 499697|01_AE|TH|2008|def	100
**seq1**	JX446872	HIV-1|name|seid 499687|01_AE|TH|2007|def	100
**seq1**	JX447018	HIV-1|name|seid 499614|01_AE|TH|2004|def	100
**seq1**	JX447170	HIV-1|name|seid 499538|01_AE|TH|2007|def	100
**seq1**	JX447422	HIV-1|name|seid 499412|01_AE|TH|2007|def	100
**seq1**	JX447442	HIV-1|name|seid 499402|01_AE|TH|2005|def	100
**seq1**	JX447352	HIV-1|name|seid 499447|01_AE|TH|2006|def	100
**seq1**	JX447460	HIV-1|name|seid 499393|01_AE|TH|2008|def	100
**seq1**	JX447628	HIV-1|name|seid 499309|01_AE|TH|2005|def	100
**seq1**	JX447676	HIV-1|name|seid 499285|01B|TH|2006|def	100
**seq1**	JX447762	HIV-1|name|seid 499242|01_AE|TH|2007|def	100
**seq1**	JX447768	HIV-1|name|seid 499239|01_AE|TH|2007|def	100
**seq1**	JX447866	HIV-1|name|seid 499190|01_AE|TH|2007|def	100
**seq1**	JX448012	HIV-1|name|seid 499117|01B|TH|2005|def	100
**seq1**	JX448236	HIV-1|name|seid 499005|01_AE|TH|2006|def	100
**seq1**	JX446763	HIV-1|name|seid 498816|01_AE|TH|2008|def	100
**seq1**	JX447573	HIV-1|name|seid 498411|01_AE|TH|2005|def	100
**seq1**	JX447763	HIV-1|name|seid 498316|01_AE|TH|2007|def	100
**seq1**	JX447793	HIV-1|name|seid 498301|B|TH|2006|def	100
**seq1**	JX447351	HIV-1|name|seid 498522|01_AE|TH|2006|def	100
**seq1**	JX448159	HIV-1|name|seid 498118|01_AE|TH|2005|def	100
**seq1**	JX448233	HIV-1|name|seid 498081|01_AE|TH|2006|def	100
**seq1**	JX448269	HIV-1|name|seid 498063|01_AE|TH|2005|def	100
**seq1**	AB703608	HIV-1|name|seid 496762|35_AD|IR|2010|def	100
**seq1**	JF932500	HIV-1|name|seid 496736|B|CN|2007|def	100
**seq1**	JF932477	HIV-1|name|seid 496731|B|CN|2007|def	100

**seq1**	JF932487	HIV-1|name|seid 496726|B|CN|2007|def	100
**seq1**	JX264248	HIV-1|name|seid 495195|01_AE|JP|1992|def	100
**seq1**	JX264372	HIV-1|name|seid 495133|B|JP|2007|def	100
**seq1**	JX264327	HIV-1|name|seid 494997|B|JP|2004|def	100
**seq1**	JX264345	HIV-1|name|seid 494988|B|JP|2005|def	100
**seq1**	JX264353	HIV-1|name|seid 494984|B|JP|2005|def	100
**seq1**	JX264359	HIV-1|name|seid 494981|B|JP|2006|def	100
**seq1**	JX264371	HIV-1|name|seid 494975|B|JP|2007|def	100
**seq1**	JX140678	HIV-1|name|seid 494863|A1B|ES|2009|def	100
**seq1**	JX244929	HIV-1|name|seid 494782|G|CM|2007|def	100
**seq1**	JQ229725	HIV-1|name|seid 494245|B|SE|2009|def	100
**seq1**	JQ229756	HIV-1|name|seid 492190|B|SE|2010|def	100
**seq1**	JN248347	HIV-1|name|seid 484378|B|TH|2005|def	100
**seq1**	JN248349	HIV-1|name|seid 484377|01B|TH|2005|def	100
**seq1**	JN685355	HIV-1|name|seid 479536|B|US|2007|def	100
**seq1**	JN685364	HIV-1|name|seid 479115|B|US|2006|def	100
**seq1**	JQ846108	HIV-1|name|seid 478300|B|ES|2007|def	100
**seq1**	JF905590	HIV-1|name|seid 477581|B|US|2005|def	100
**seq1**	JF905592	HIV-1|name|seid 477580|B|US|2006|def	100
**seq1**	JQ316134	HIV-1|name|seid 477599|B|KR|2005|def	100
**seq1**	JN014135	HIV-1|name|seid 473478|C|ZM|1998|def	100
**seq1**	JN014279	HIV-1|name|seid 473406|C|ZM|2002|def	100
**seq1**	JN014266	HIV-1|name|seid 472922|C|ZM|2002|def	100
**seq1**	JN014350	HIV-1|name|seid 472880|C|ZM|2000|def	100
**seq1**	JN014360	HIV-1|name|seid 472875|C|ZM|2000|def	100
**seq1**	JQ927640	HIV-1|name|seid 472584|B|US|2005|def	100
**seq1**	JF804812	HIV-1|name|seid 476613|28_BF|BR|2005|def	100
**seq1**	JF804807	HIV-1|name|seid	100

		476588|29_BF|BR|2005|def	
**seq1**	GQ430655	HIV-1|name|seid 319506|B|KE|1995|def	100
**seq1**	GQ430668	HIV-1|name|seid 319493|B|KE|1995|def	100
**seq1**	GQ432541	HIV-1|name|seid 317620|B|KE|1996|def	100

Figure S1. Complete alignment of deep sequencing reads for A1 (cohort 1).

The reference sequence is shown at the top (GenBank AF316544). The number and percentage of identical reads are indicated.

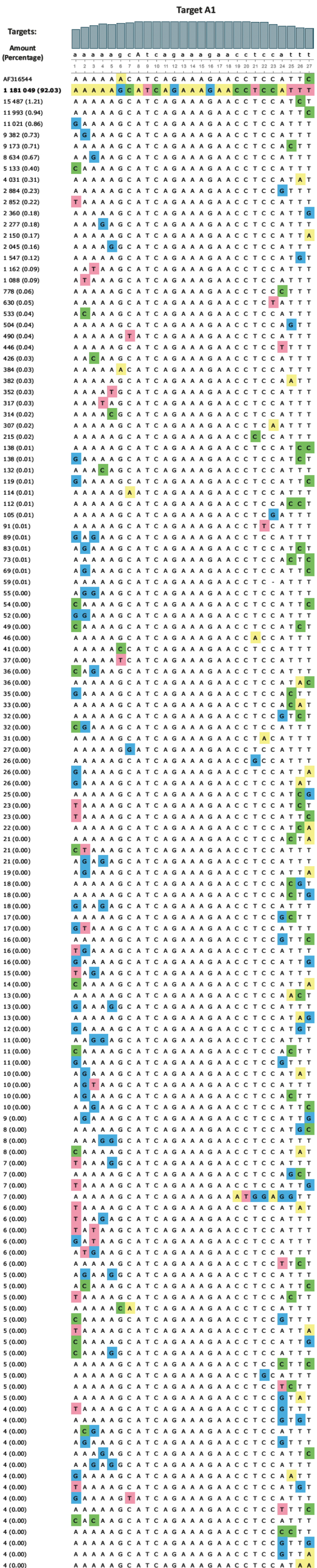

Target A1
Targets:
Amount (Percentage)
a a a a a g c A t c a g a a a g a a c c t c c a t t t
AF316544
1 181 049 (92.03)

Figure S2. Complete alignment of deep sequencing reads for A2 (cohort 1).

The reference sequence is shown at the top (GenBank AF316544). The number and percentage of identical reads are indicated.

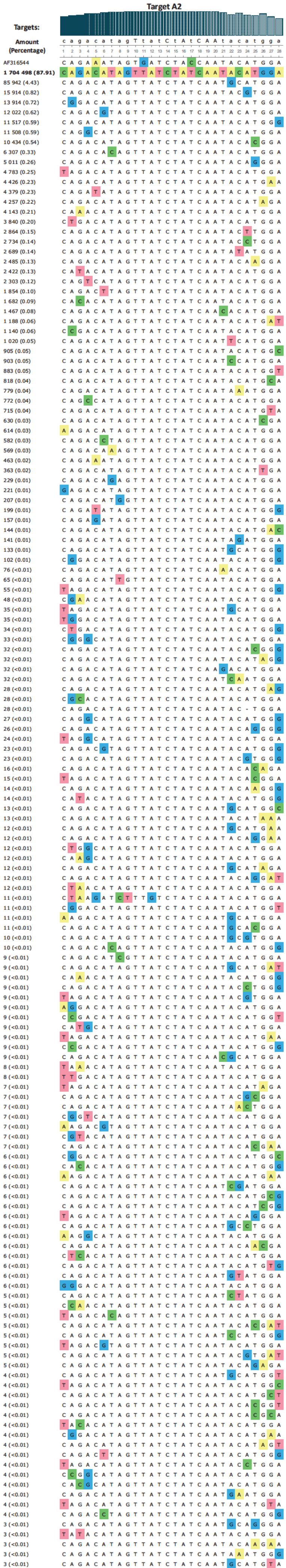

Target A2
Targets:
Amount
(Percentage)
AF316544

Figure S3. Complete alignment of deep sequencing reads for A3 (cohort 1).

The reference sequence is shown at the top (GenBank AF316544). The number and percentage of identical reads are indicated.

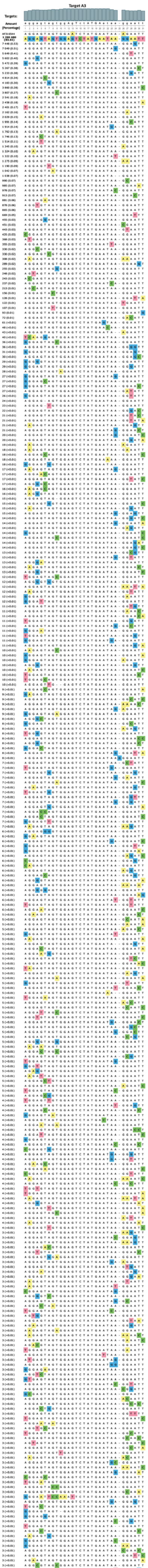
Target A3
Targets:
Amount
(Percentage)
AF316544
1 290 680
(92.61)

Figure S4. Complete alignment of deep sequencing reads for A4 (cohort 1).

The reference sequence is shown at the top (GenBank AF316544). The number and percentage of identical reads are indicated.

Target A4

Targets:

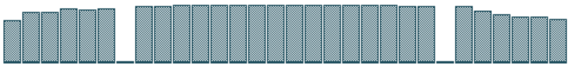

Amount (Percentage)	t g t g t g - g a C t A T a G t a g g T A t a - g a a t a t (positions 1–30)
AF316544	AGTGTG-GACCATAGTGTATATA-GAATAT
**138 085 (92.44)**	TGTGTG-GACTATAGTAGGTATA-GAATAT
1 165 (0.78)	TGTGTG-GACTATAGTAGGTATA-GGATAT
1 078 (0.72)	TGCGTG-GACTATAGTAGGTATA-GAATAT
1 050 (0.70)	TGTGTG-GACTATAGTAGGTATA-GAGTAT
1 031 (0.69)	CGTGTG-GACTATAGTAGGTATA-GAATAT
1 001 (0.67)	TGTGTG-GACTATAGTAGGTATA-GAATGT
902 (0.60)	TGTGTG-GACTATAGTAGGTATA-GAACAT
839 (0.56)	TGTGTG-GACTATAGTAGGTATA-GAATAC
357 (0.24)	TGTGTG-GACTATAGTAGGTATA-GAATAA
338 (0.23)	TATGTG-GACTATAGTAGGTATA-GAATAT
331 (0.22)	TGTGCG-GACTATAGTAGGTATA-GAATAT
324 (0.22)	TGAGTG-GACTATAGTAGGTATA-GAATAT
304 (0.20)	TGTGTG-GACTATAGTAGGTATA-GAAAAT
190 (0.13)	AGTGTG-GACTATAGTAGGTATA-GAATAT
188 (0.13)	TGTGTG-GACTATAGTAGGTATA-GAAGAT
173 (0.12)	TGTGTG-GACTATAGTAGGTATA-GTATAT
166 (0.11)	TGTGTG-GACTATAGTAGGTATA-GAATCT
151 (0.10)	TGTGTG-GACTATAGTAGGTATA-GATTAT
134 (0.09)	TGTGTG-GACTATAGTAGGTATA-GAATTT
119 (0.08)	TGTGAG-GACTATAGTAGGTATA-GAATAT
115 (0.08)	GTTGTG-GACTATAGTAGGTATA-GAATAT
104 (0.07)	TGTGTG-GACTATAGTAGGTATA-GACTAT
102 (0.07)	TGTGT--GACTATAGTAGGTATA-GAATAT
92 (0.06)	TGGGTG-GACTATAGTAGGTATA-GAATAT
90 (0.06)	TGTGTG-GACTATAGTAGGTATA-GAATAG
84 (0.06)	TGTGTG-GACTATAGTAGGTATA-GCATAT
57 (0.04)	TGTATG-GACTATAGTAGGTATA-GAATAT
48 (0.03)	TGTGTG-AACTATAGTAGGTATA-GAATAT
48 (0.03)	TGTGTA-GACTATAGTAGGTATA-GAATAT
46 (0.03)	TGTGTG-GACTATAGTAGGTATA-AAATAT
46 (0.03)	TGTGTG-GACTATAGTAGGTATG-GAATAT
39 (0.03)	TGTGGG-GACTATAGTAGGTATA-GAATAT
36 (0.02)	GGTGTG-GACTATAGTAGGTATA-GAATAT
35 (0.02)	TCTGTG-GACTATAGTAGGTATA-GAATAT
32 (0.02)	TGTGTG-GGCTATAGTAGGTATA-GAATAT
29 (0.02)	TGTGTG-GACTATAGTAGGTATA-GAAATT
25 (0.02)	TGTGTG-GACTATAGTAGGTACA-GAATAT
18 (0.01)	TGTGTG-GACTATAGTAGGTATA-GAACGT
18 (0.01)	TTTGTG-GACTATAGTAGGTATA-GAATAT
16 (0.01)	TGTGTG-CACTATAGTAGGTATA-GAATAT
14 (0.01)	TGTGTG-GACTATAGTAGGTATA-CAATAT
12 (0.01)	TGTGTG-GACTATAGTAGGTATT-GAATAT
12 (0.01)	TGTGTG-GACTATAGTAGGTATA-GAATCA
12 (0.01)	TGTGTG-GACTATAGTAGGTATA-GATATA
11 (0.01)	TGTGTG-TACTATAGTAGGTATA-GAATAT
11 (0.01)	TGTGTT-GACTATAGTAGGTATA-GAATAT
11 (0.01)	TGTGTG-GACTATAGTAGGTATA-TAATAT
11 (0.01)	TGTGTG-GACTATAGTAGGTATA-GAGTAA
11 (0.01)	TGTGTG-GACTATAGTAGGTATC-GAATAT
10 (0.01)	TGTGTG-GACTATAGTAGGTATA-GAAACT
10 (0.01)	TGTCTG-GACTATAGTAGGTATA-GAATAT
9 (0.01)	TGTGTG-GACTATAGTAGGTATA-GAGTGT
9 (0.01)	CGTGTG-GACTATAGTAGGTATA-GAATAC
9 (0.01)	TGTGTC-GACTATAGTAGGTATA-GAATAT
8 (0.01)	CGTGTG-GACTATAGTAGGTATA-GAGTAT
7 (0.01)	TGTGTG-GACTATAGTAGGTATA-GAGTAC
7 (0.01)	TGTGTG-GACTATAGTAGGTATA-GAGAAT
6 (<0.01)	CGTGTG-GACTATAGTAGGTATA-GAATGT
6 (<0.01)	CGCGTG-GACTATAGTAGGTATA-GAATAT
5 (<0.01)	TGTGTG-GACTATAGTAGGTATA-GAACTA
5 (<0.01)	TGTGTG-GACTATAGTAGGTATA-GGATGT
5 (<0.01)	TGTGTG-GACTATAGTAGGTATA-GAATGC
5 (<0.01)	TGTGTG-GCCTATAGTAGGTATA-GAATAT
5 (<0.01)	TGTGTG-GACTATAGTAGGTATA-GAGCAT
5 (<0.01)	TGTGTGTGACTATAGTAGGTATA-GAATAT
5 (<0.01)	TGTGTG-GACTATAGTAGGTATA-GAACTT
5 (<0.01)	CGTGTG-GACTATAGTAGGTATA-GAACAT
5 (<0.01)	TGTTTG-GACTATAGTAGGTATA-GAATAT
4 (<0.01)	TGCGTG-GACTATAGTAGGTATA-GAATAC
4 (<0.01)	TGTGTG-GACTATAGTAGGTATA-GAACAC
3 (<0.01)	TGTGTG-GACTATAGTAGGTATA-GGACAT
3 (<0.01)	TGTGTG-GTCTATAGTAGGTATA-GAATAT
3 (<0.01)	CGTGTG-GACTATAGTAGGTATA-GAATAA
3 (<0.01)	CGTGTG-GACTATAGTAGGTATA-GGATAT
3 (<0.01)	AGTGTG-GACTATAGTAGGTATA-GAATAC
3 (<0.01)	TGTGG--GACTATAGTAGGTATA-GAATAT
3 (<0.01)	TGTGTG-GACTATAGTAGGTATA-GATCAT
2 (<0.01)	AGCGTG-GACTATAGTAGGTATA-GAATAT
2 (<0.01)	TGTGTG-GACTATAGTAGGTATA-GAAAAT
2 (<0.01)	TGTGTG-GACTATAGTAGGTATA-GGATAC
2 (<0.01)	CATGTG-GACTATAGTAGGTATA-GAATAT
2 (<0.01)	TGTGTG-GACTATAGTAGGTATA-GCATGT

Figure S5. Complete alignment of deep sequencing reads for A5 (cohort 1).

The reference sequence is shown at the top (GenBank AF316544). The number and percentage of identical reads are indicated.

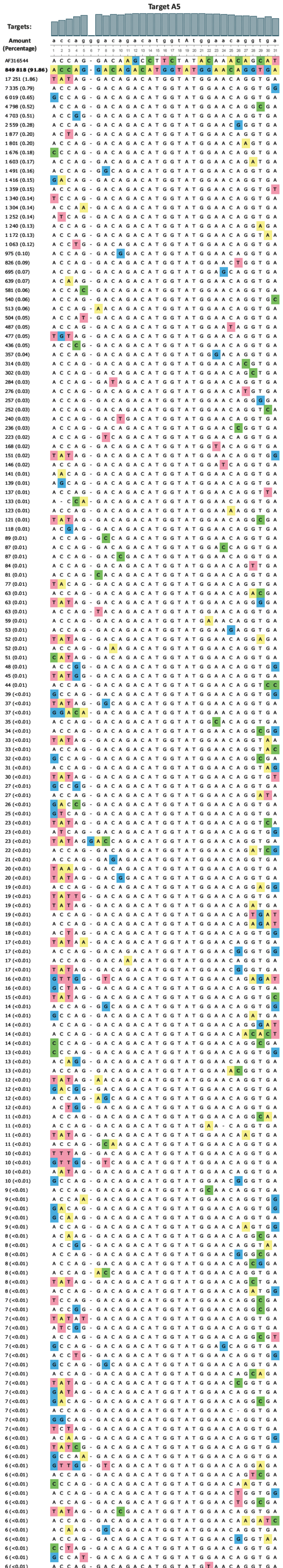

Target A5
Targets:
Amount (Percentage)
AF316544
849 818 (91.86)

Figure S6. Complete alignment of deep sequencing reads for A6 (cohort 1).

The reference sequence is shown at the top (GenBank AF316544). The number and percentage of identical reads are indicated.

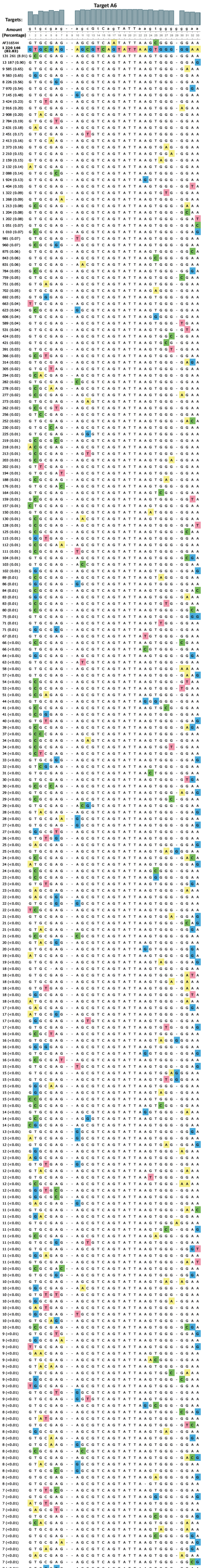
Target A6
Targets:
Amount
(Percentage)
g t g c g a g - - a g c G t C a g T A T T a a g t g g g g g g a a
AF316544

Figure S7. Complete alignment of deep sequencing reads for A1 (cohort 2).

The reference sequence is shown at the top (GenBank AF316544). The number and percentage of identical reads are indicated.

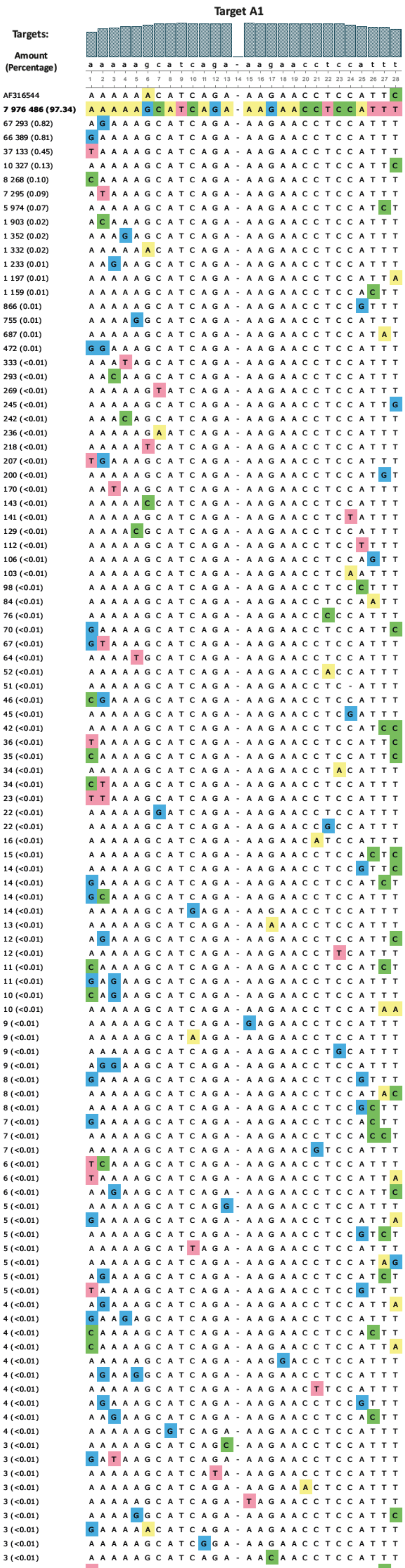

Target A1
Targets:
Amount (Percentage)
a a a a a g c a t c a g a - a a g a a c c t c c a t t t
AF316544
7 976 486 (97.34)

Figure S8. Complete alignment of deep sequencing reads for A2 (cohort 2).

The reference sequence is shown at the top (GenBank AF316544). The number and percentage of identical reads are indicated.

Target A2

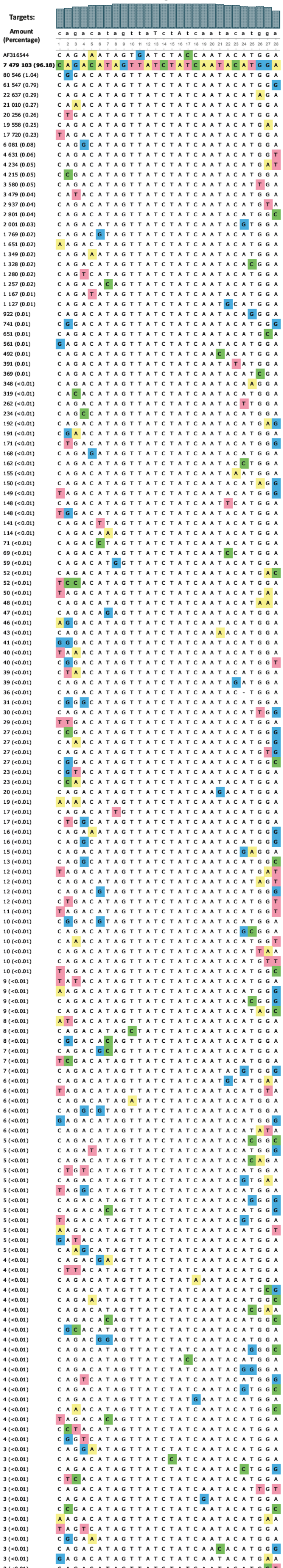

Figure S9. Complete alignment of deep sequencing reads for A3 (cohort 2).

The reference sequence is shown at the top (GenBank AF316544). The number and percentage of identical reads are indicated.

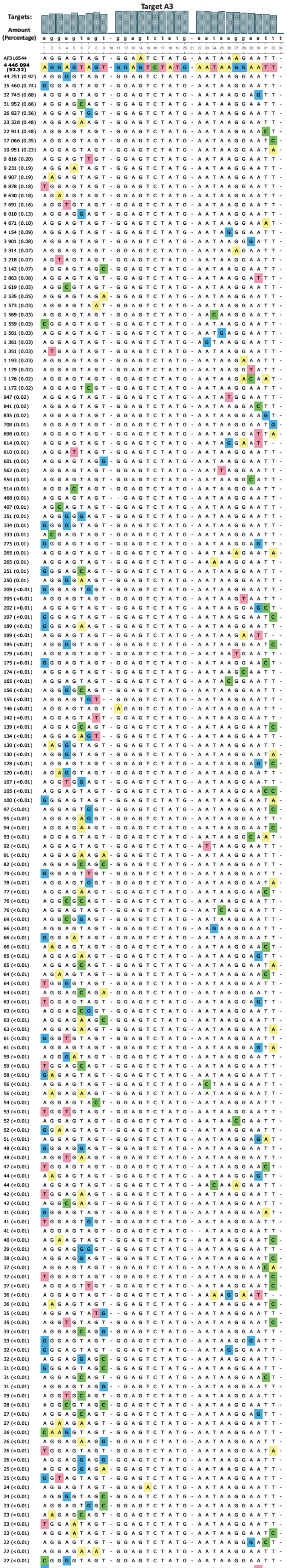
Target A3
Targets:
Amount
(Percentage)
AF316544

Figure S10. Complete alignment of deep sequencing reads for A4 (cohort 2).

The reference sequence is shown at the top (GenBank AF316544). The number and percentage of identical reads are indicated.

Target A4

Targets:

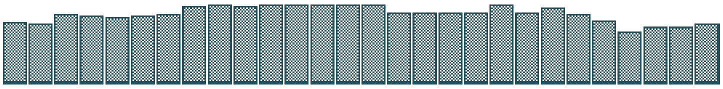

Amount (Percentage)	t	g	t	g	t	g	g	a	C	t	A	T	A	G	t	a	g	g	t	a	t	a	g	a	a	t	a	t
	1	2	3	4	5	6	7	8	9	10	11	12	13	14	15	16	17	18	19	20	21	22	23	24	25	26	27	28
AF316544	A	G	T	G	T	G	G	A	C	C	A	T	A	G	T	G	T	A	T	A	T	A	G	A	A	T	A	T
**73 906 (95.28)**	T	G	T	G	T	G	G	A	C	T	A	T	A	G	T	A	G	G	T	A	T	A	G	A	A	T	A	T
656 (0.85)	C	G	T	G	T	G	G	A	C	T	A	T	A	G	T	A	G	G	T	A	T	A	G	A	A	T	A	T
596 (0.77)	T	G	T	G	T	G	G	A	C	T	A	T	A	G	T	A	G	G	T	A	T	A	G	A	G	T	A	T
497 (0.64)	T	A	T	G	T	G	G	A	C	T	A	T	A	G	T	A	G	G	T	A	T	A	G	A	A	T	A	T
418 (0.54)	T	G	C	G	T	G	G	A	C	T	A	T	A	G	T	A	G	G	T	A	T	A	G	A	A	T	A	T
369 (0.48)	T	G	T	G	T	G	G	A	C	T	A	T	A	G	T	A	G	G	T	A	T	A	G	A	A	T	A	C
137 (0.18)	A	G	T	G	T	G	G	A	C	T	A	T	A	G	T	A	G	G	T	A	T	A	G	A	A	T	A	T
121 (0.16)	T	G	A	G	T	G	G	A	C	T	A	T	A	G	T	A	G	G	T	A	T	A	G	A	A	T	A	T
108 (0.14)	T	G	T	G	T	G	G	A	C	T	A	T	A	G	T	A	G	G	T	A	T	A	G	A	A	C	A	T
70 (0.09)	T	G	T	G	T	G	G	A	C	T	A	T	A	G	T	A	G	G	T	A	T	A	G	A	A	T	G	T
49 (0.06)	T	G	T	G	T	G	G	A	C	T	A	T	A	G	T	A	G	G	T	A	T	A	G	A	A	T	A	A
43 (0.06)	-	G	T	G	T	G	G	A	C	T	A	T	A	G	T	A	G	G	T	A	T	A	G	A	A	T	A	T
29 (0.04)	T	G	T	G	T	G	G	A	C	T	A	T	A	G	T	A	G	G	T	A	T	A	G	-	-	-	-	-
27 (0.04)	T	A	T	G	T	G	G	A	C	T	A	T	A	G	T	A	G	G	T	A	T	A	G	A	G	T	A	T
27 (0.04)	-	-	T	G	T	G	G	A	C	T	A	T	A	G	T	A	G	G	T	A	T	A	G	A	A	T	A	T
26 (0.03)	G	G	T	G	T	G	G	A	C	T	A	T	A	G	T	A	G	G	T	A	T	A	G	A	A	T	A	T
26 (0.03)	T	G	G	G	T	G	G	A	C	T	A	T	A	G	T	A	G	G	T	A	T	A	G	A	A	T	A	T
26 (0.03)	T	G	T	G	T	G	G	A	C	T	A	T	A	G	T	A	G	G	T	A	T	G	G	A	A	T	A	T
25 (0.03)	T	A	T	A	C	C	T	A	C	T	A	T	A	G	T	C	C	A	C	A	C	A	A	C	T	A	C	T
25 (0.03)	T	G	T	G	A	G	G	A	C	T	A	T	A	G	T	A	G	G	T	A	T	A	G	A	A	T	A	T
24 (0.03)	T	G	T	G	C	G	G	A	C	T	A	T	A	G	T	A	G	G	T	A	T	A	G	A	A	T	A	T
23 (0.03)	T	G	T	A	T	G	G	A	C	T	A	T	A	G	T	A	G	G	T	A	T	A	G	A	A	T	A	T
21 (0.03)	T	G	T	G	T	G	G	A	C	T	A	T	A	G	T	A	G	G	T	A	T	A	G	G	A	T	A	T
19 (0.02)	T	G	T	G	T	G	G	A	C	T	A	T	A	G	T	A	G	G	T	A	T	A	G	A	A	T	T	T
15 (0.02)	-	-	-	G	T	G	G	A	C	T	A	T	A	G	T	A	G	G	T	A	T	A	G	A	A	T	A	T
15 (0.02)	T	G	T	G	T	G	G	G	C	T	A	T	A	G	T	A	G	G	T	A	T	A	G	A	A	T	A	T
15 (0.02)	T	A	T	A	C	C	T	A	C	T	A	T	A	G	T	C	C	A	C	A	C	A	A	C	T	A	T	T
14 (0.02)	T	G	T	G	T	A	G	A	C	T	A	T	A	G	T	A	G	G	T	A	T	A	G	A	A	T	A	T
12 (0.01)	T	G	T	G	T	G	G	A	C	T	A	T	A	G	T	A	G	G	T	A	T	A	G	A	A	T	-	-
11 (0.01)	T	G	T	G	T	G	G	A	C	T	A	T	A	G	T	A	G	G	T	A	T	A	G	A	-	-	-	-
11 (0.01)	G	T	T	G	T	G	G	A	C	T	A	T	A	G	T	A	G	G	T	A	T	A	G	A	A	T	A	T
11 (0.01)	T	T	T	G	T	G	G	A	C	T	A	T	A	G	T	A	G	G	T	A	T	A	G	A	A	T	A	T
10 (0.01)	T	G	T	G	T	G	G	A	C	T	A	T	A	G	T	A	G	G	T	A	T	A	G	A	A	A	A	T
9 (0.01)	T	G	T	G	T	G	G	A	C	T	A	T	A	G	T	A	G	G	T	A	T	A	G	A	A	T	A	-
9 (0.01)	T	G	T	G	T	G	G	A	C	T	A	T	A	G	T	A	G	G	T	A	T	A	G	A	A	T	A	G
8 (0.01)	C	G	T	G	T	G	G	A	C	T	A	T	A	G	T	A	G	G	T	A	T	A	G	A	A	T	G	T
8 (0.01)	T	G	T	G	T	G	A	A	C	T	A	T	A	G	T	A	G	G	T	A	T	A	G	A	A	T	A	T
7 (0.01)	T	G	T	G	T	G	G	A	C	T	A	T	A	G	T	A	G	G	T	A	T	A	G	A	A	A	T	A
6 (0.01)	T	G	T	G	T	G	G	A	C	T	A	T	A	G	T	A	G	G	T	A	T	A	G	A	G	-	-	-
6 (0.01)	T	G	T	G	T	G	G	A	C	T	A	T	A	G	T	A	G	G	T	A	T	A	G	A	G	C	A	T
6 (0.01)	T	G	T	G	T	G	G	A	C	T	A	T	A	G	T	A	G	G	T	A	T	A	A	A	A	T	A	T
6 (0.01)	T	G	T	G	T	G	G	A	C	T	A	T	A	G	T	A	G	G	T	A	T	A	G	C	A	T	A	T
5 (0.01)	T	G	T	G	T	G	G	A	C	T	A	T	A	G	T	A	G	G	T	A	T	A	G	T	A	T	A	T
5 (0.01)	T	G	T	G	T	G	G	A	C	T	A	T	A	G	T	A	G	G	T	A	T	A	G	A	G	A	A	T
5 (0.01)	T	G	T	G	T	G	G	A	C	T	A	T	A	G	T	A	G	G	T	A	T	A	-	-	-	-	-	-
5 (0.01)	T	G	T	G	T	G	G	A	C	T	A	T	A	G	T	A	G	G	T	A	T	A	G	A	A	T	C	T
5 (0.01)	T	G	T	G	T	G	G	A	C	T	A	T	A	G	T	A	G	G	T	A	T	A	G	A	A	-	-	-
4 (0.01)	T	G	T	G	G	G	G	A	C	T	A	T	A	G	T	A	G	G	T	A	T	A	G	A	A	T	A	T
4 (0.01)	C	G	T	G	A	G	G	A	C	T	A	T	A	G	T	A	G	G	T	A	T	A	G	A	A	T	A	T
3 (0.00)	T	G	T	G	T	G	G	A	C	T	A	T	A	G	T	A	G	G	T	A	T	A	G	C	G	T	A	T
3 (0.00)	C	G	C	G	T	G	G	A	C	T	A	T	A	G	T	A	G	G	T	A	T	A	G	A	A	T	A	T
3 (0.00)	C	G	T	G	T	G	G	A	C	T	A	T	A	G	T	A	G	G	T	A	T	A	G	A	A	T	A	C
3 (0.00)	T	G	T	G	T	G	G	A	C	T	A	T	A	G	T	A	G	G	T	A	T	A	G	A	C	T	A	T
3 (0.00)	T	A	T	G	T	G	G	A	C	T	A	T	A	G	T	A	G	G	T	A	T	A	G	A	G	-	-	-
3 (0.00)	T	G	T	G	T	G	C	A	C	T	A	T	A	G	T	A	G	G	T	A	T	A	G	A	A	T	A	T
2 (0.00)	T	G	T	G	T	G	G	A	C	T	A	T	A	G	T	A	G	G	T	A	T	A	G	A	T	T	A	T
2 (0.00)	T	G	T	G	T	G	G	A	C	T	A	T	A	G	T	A	G	G	T	A	T	A	G	A	G	T	-	-
2 (0.00)	T	G	T	G	T	G	G	T	C	T	A	T	A	G	T	A	G	G	T	A	T	A	G	A	A	T	A	T

Figure S11. Complete alignment of deep sequencing reads for A5 (cohort 2).

The reference sequence is shown at the top (GenBank AF316544). The number and percentage of identical reads are indicated.

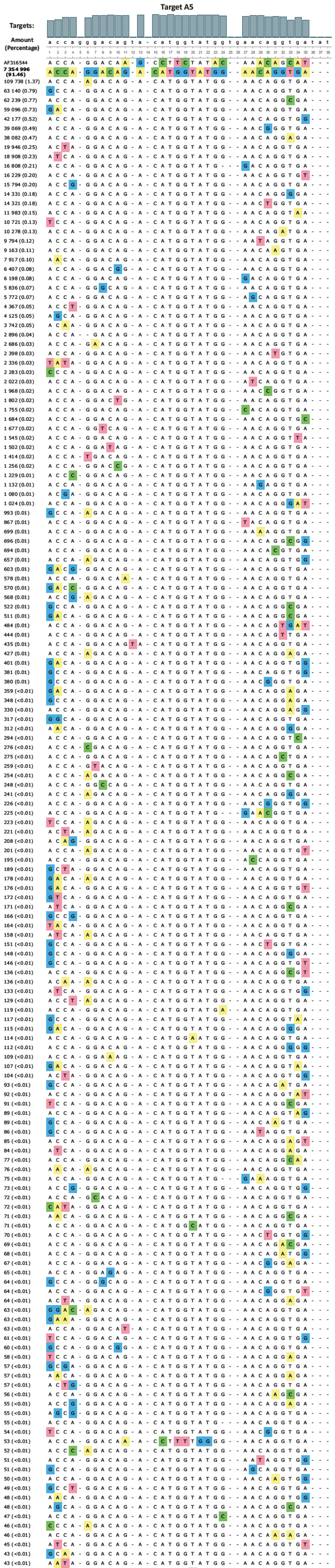
Target A5
Targets:
Amount
(Percentage)
AF316544

Figure S12. Complete alignment of deep sequencing reads for A6 (cohort 2).

The reference sequence is shown at the top (GenBank AF316544). The number and percentage of identical reads are indicated.

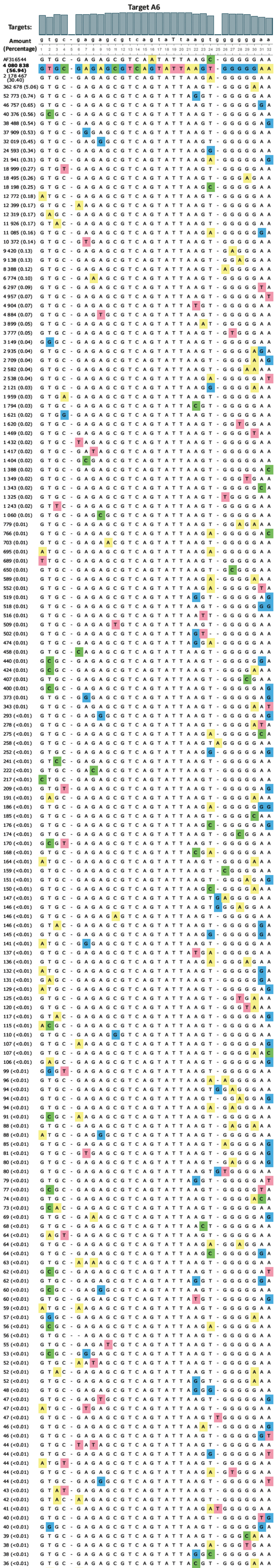
Target A6
Targets:
Amount
(Percentage)

Figure S13. Conserved Domains Database (CDD) and Resources – NCBI – for the targets A1–A6.

The regions corresponding to the targets are highlighted in yellow.

Conserved Domains Database (CDD) and Resources - NCBI

A1, A2

RT_Rtv: Reverse transcriptases (RTs) from retroviruses (Rtvs). RTs catalyze the conversion of single-stranded RNA into double-stranded viral DNA for integration into host chromosomes.

Pssm-ID: 238823 [Multi-domain] **Cd Length:** 213 **Bit Score:** 350.43 **E-value:** 3.09e-113

```
                          10        20        30        40        50        60        70        80
                  ....*....|....*....|....*....|....*....|....*....|....*....|....*....|....*....|
gi 14530264   173 GPKVKQWPLTEEKIKALTEICKEMEKEGKISKIGpeNPYNTPIFAIKKKDStKWRKLVDFRELNKRTQDFWEVQLGIPHP 252
Cdd:cd01645     1 PVWIKQWPLTEEKLEALTELVTEQLKEGHIEPST--SPWNTPVFVIKKKSG-KWRLLHDLRAVNAQTQDMGALQPGLPHP 77
                          90       100       110       120       130       140       150       160
                  ....*....|....*....|....*....|....*....|....*....|....*....|....*....|....*....|
gi 14530264   253 AGLKKKKSVTVLDVGDAYFSVPLDEGFRKYTAFTIPSINNETPGVRYQYNVLPQGWKGSPAIFQSSMTKILEPFRADNPE 332
Cdd:cd01645    78 AALPKGWPLIVLDLKDCFFSIPLHPDDRERFAFTVPSINNKGPAKRYQWKVLPQGMKNSPTICQSFVAQALEPFRKQYPD 157
                         170       180       190       200       210
                  ....*....|....*....|....*....|....*....|....*....|....*..
gi 14530264   333 IVIYQYMDDLYVGSDLEiGQHRAKIEELREHLLRWGFTTPDKKHQKEPPFLWMGYEL 389
Cdd:cd01645   158 IVIYHYMDDILIASDLE-GQLREIYEELRQTLLRWGLTIPPEKVQKEPPFQYLGYEL 213
                     A2                                      A1
```

A3

Integrase core domain

Integrase mediates integration of a DNA copy of the viral genome into the host chromosome. Integrase is composed of three domains. The amino-terminal domain is a zinc binding domain pfam02022. This domain is the central catalytic domain. The carboxyl terminal domain that is a non-specific DNA binding domain pfam00552. The catalytic domain acts as an endonuclease when two nucleotides are removed from the 3' ends of the blunt-ended viral DNA made by reverse transcription. This domain also catalyzes the DNA strand transfer reaction of the 3' ends of the viral DNA to the 5' ends of the integration site.

```
                             10        20        30        40        50        60        70        80
                     ....*....|....*....|....*....|....*....|....*....|....*....|....*....|....*....|
gi 14530264      773 PGVWQLDCTHLEGKI------ILVAVHVASGYIEAEVIPAETGQETAYFILKLA----GGWPVKVIHTDNGSNFTSAAVK 842
Cdd:pfam00665      2 NELWQTDFTYVRVPGgggklyLAVAVDDFSREIVAWALSSEMDAELVIDALKRAiefrGPPGPKIIHSDNGSEYTSKAFQ 81
                             90       100       110
                     ....*....|....*....|....*....|..
gi 14530264      843 AACWWANVTQEFGIPYNPQSQGVVESMNKELK 874
Cdd:pfam00665     82 EFLAHYGITHSFSRPGNPQDNGKVERFNGTLK 113
```

A4

Vpu protein; The Vpu protein contains an N-terminal transmembrane spanning region and a C-terminal cytoplasmic region. The HIV-1 Vpu protein stimulates virus production by enhancing the release of viral particles from infected cells. The VPU protein binds specifically to CD4.

```
                         10        20        30        40        50        60        70
                 ....*....|....*....|....*....|....*....|....*....|....*....|....*....|....*
gi 14530268    1 MQALEISAIVGLIVAFIAATVVWTIVYIEYRKIRKQKRIERLLDRIGERAEDSGNESEGDAEE-LAKLVEMGGFD 74
Cdd:pfam00558  1 MLLLEIIGLIALIVALIINIVVWTIVYREYRKIKKQREILRLIKRIRERAEDSGNESNGDEEEeLADLVHSHGFD 75
```

A5

Envelope glycoprotein GP120; The entry of HIV requires interaction of viral GP120 with CD4 and a chemokine receptor on the cell surface.

Pssm-ID: 278917 **Cd Length:** 525 **Bit Score:** 36.68 **E-value:** 2.62e-05

```
                           10        20        30
                   ....*....|....*....|....*....|..
Query_22997      7 VEITCIRPNNNTRKSIRF--GPGQAFYTNSII 36
Cdd:pfam00516 295 LEINCKRPGNKTRKPIRImrGPGRAFVFHGKI 326
```

A6

gag gene protein p17 (matrix protein); The matrix protein forms an icosahedral shell associated with the inner membrane of the mature immunodeficiency virus.

Pssm-ID: 249943 **Cd Length:** 140 **Bit Score:** 195.93 **E-value:** 5.21e-60

```
                           10        20        30        40        50        60        70        80
                   ....*....|....*....|....*....|....*....|....*....|....*....|....*....|....*....|
gi_14530263      1 ARASILSGGKLEAWEKIRLRPGGKKKYRLKHLVWASRELEKFSINPGLLETAAGCRQILGQLQPALQTGTEELRSLYNTV 80
Cdd:pfam00540    2 ARASVLSGGELDKWEKIRLRPGGKKKYKLKHLVWASRELERFAVNPGLLETSEGCRKILGQLQPSLQTGSEGLRSLYNTV 81
                           90       100       110       120       130       140
                   ....*....|....*....|....*....|....*....|....*....|....*....|.
gi_14530263     81 AVLYCVHQKIEVKDTKEALDKIEEEQNTCKQRTQhaaADTGSSRSQDYRGSSSQNYPIVQN 141
Cdd:pfam00540   82 AVLYCVHQRIDVKDTKEALEKIEEEQNKSKKKKT---AVPPGAQQAANTGGTGNSSGVSQN 139
```
